# A Method for Assessing the Robustness of Protein Structures by Randomizing Packing Interactions

**DOI:** 10.3389/fmolb.2022.849272

**Published:** 2022-06-27

**Authors:** Shilpa Yadahalli, Lakshmi P. Jayanthi, Shachi Gosavi

**Affiliations:** Simons Centre for the Study of Living Machines, National Centre for Biological Sciences, Tata Institute of Fundamental Research, Bangalore, India

**Keywords:** packing perturbations, protein scaffold, structure-based models, molecular dynamics simulations, sequence permutations, robustness of protein structure, protein folding

## Abstract

Many single-domain proteins are not only stable and water-soluble, but they also populate few to no intermediates during folding. This reduces interactions between partially folded proteins, misfolding, and aggregation, and makes the proteins tractable in biotechnological applications. Natural proteins fold thus, not necessarily only because their structures are well-suited for folding, but because their sequences optimize packing and fit their structures well. In contrast, folding experiments on the *de novo* designed Top7 suggest that it populates several intermediates. Additionally, in *de novo* protein design, where sequences are designed for natural and new non-natural structures, tens of sequences still need to be tested before success is achieved. Both these issues may be caused by the specific scaffolds used in design, i.e., some protein scaffolds may be more tolerant to packing perturbations and varied sequences. Here, we report a computational method for assessing the response of protein structures to packing perturbations. We then benchmark this method using designed proteins and find that it can identify scaffolds whose folding gets disrupted upon perturbing packing, leading to the population of intermediates. The method can also isolate regions of both natural and designed scaffolds that are sensitive to such perturbations and identify contacts which when present can rescue folding. Overall, this method can be used to identify protein scaffolds that are more amenable to whole protein design as well as to identify protein regions which are sensitive to perturbations and where further mutations should be avoided during protein engineering.

## Introduction

With advances in protein design methods, whole protein design using both naturally occurring and computationally designed protein scaffolds has become common (Pokala and Handel, 2001; Kuhlman and Bradley, 2019). These design methods usually choose sequences that minimize the energy of the target structure but do not optimize the entire folding energy landscape (Pokala and Handel, 2001; Chikenji et al., 2006; Kuhlman and Bradley, 2019; Pan et al., 2020). However, natural selection acts on both the stability and the foldability of proteins, creating funnel-shaped energy landscapes. Non-native interactions (interactions not present in the folded state of the protein), which could otherwise have created traps or misfolded ensembles on such landscapes, interfere little with the productive folding of natural proteins (Bryngelson et al., 1995; Onuchic et al., 1997; Noel et al., 2010a). Additionally, natural proteins also fold cooperatively, in an almost all or nothing manner, populating few intermediates during folding (Jackson and Fersht, 1991). This folding cooperativity reduces the interactions between partially folded proteins, interactions which could lead to protein aggregation, disruption of protein function and disease (Dobson, 1999).

Since designed proteins are expressed and purified from cells, they must also be able to fold. However, folding experiments on Top7 (Kuhlman et al., 2003), the first protein to be designed with a fold not found in nature, show that it populates several intermediates during folding (Dantas et al., 2006). Based on these experiments, it was hypothesized that the designed non-natural topology of Top7 led to its complex folding (Scalley-Kim and Baker, 2004; Watters et al., 2007). Subsequent folding simulations using coarse-grained structure-based models suggested that the reason for the non-cooperative folding of Top7 may be that the packing of its sequence onto its structure creates defects which stall folding and lead to the population of intermediates (Zhang and Chan, 2009; Yadahalli and Gosavi, 2014). In other words, the sequence-structure fit for Top7 is not optimal. These simulations also suggested that the Top7 structure was sensitive to packing perturbations and a more nuanced approach to packing would be required to find a good sequence-structure fit. Thus, “ideal” protein structures like Top7 (Koga and Koga, 2019) may not always be good scaffolds for the design of proteins whose folding is similar to that of natural proteins.

A fold is said to be “designable” if it can accommodate many sequences (Helling et al., 2001; England and Shakhnovich, 2003; Ben-David et al., 2019) and a diversity of sequences are known to fold into the naturally occurring ‘‘superfolds’’ (Orengo et al., 1994; Magner et al., 2015). However, the structures of proteins which fold to a given superfold are marginally different from each other, containing protein-specific local features such as loops, kinks and bulges which have evolved to accommodate their individual functions (Finkelstein et al., 1993; Helling et al., 2001). It is also known both from experiments and folding simulations that these protein specific functional features can affect folding with one protein from the same fold populating an intermediate, while another protein folds cooperatively (Chavez et al., 2006; Gosavi et al., 2008; Hills and Brooks, 2009; Gosavi, 2013; Giri Rao and Gosavi, 2016). Thus, just structural information such as fold classification is not sufficient to select a natural protein scaffold for protein redesign. Additionally, simulations of a structure-based model of the naturally occurring *E. coli* RNase-H (ecoRNase-H), which recapitulate key experimental folding results, show that its structure is sensitive to packing perturbations (Yadahalli and Gosavi, 2016). However, evolution has been able to counteract the effect of this sensitivity by selecting a sequence whose packing onto the ecoRNase-H structure preserves cooperative folding. Consequently, even information derived from folding experiments may not be enough to choose a natural scaffold for protein redesign.

**FIGURE 1:**
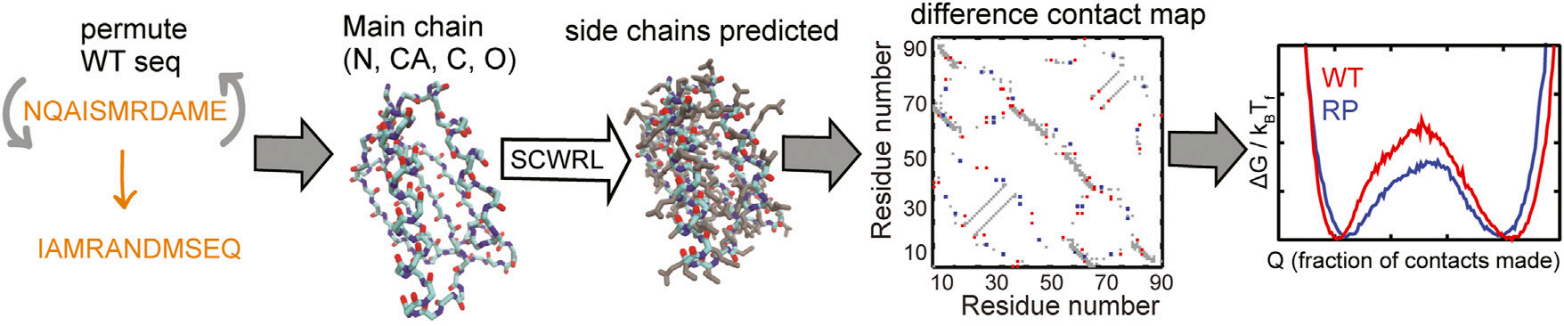
A flowchart of the RP method. The selected WT protein sequence (shown in orange) is randomly permuted. The side-chains are removed from the structure of the WT protein but the backbone atoms N (shown in blue), CA, C (both in cyan) and O (red) are retained. The randomly permuted sequence is put back onto this backbone using the program SCWRL. SCWRL predicts sidechain rotamers given the backbone structure and a sequence. The predicted side chains are shown in grey. Contact maps are calculated from both the WT and the randomly permuted protein structures. The difference contact map is shown here. The X and Y axes are indices on the residue number. A square marked at (x, y) implies that the residues x and y are in contact. This contact map is symmetric with the same information present on both sides of the x = y line, i.e., (x, y) and (y, x) represent the same contact. The grey contacts are common to both the WT and the random permutant (RP). Red contacts are present only in the WT and blue contacts are present only in the RP. Cα-SBMs (see Methods) are constructed using the structure of the protein and these contact maps. Folding simulations of these SBMs are performed using molecular dynamics simulations and free energy profiles (negative logarithm of the population distributions vs. a measure of foldedness) are calculated and plotted. A comparison of the WT and RP free energy profiles can be used to assess if the protein structure is robust to changes in sequence. Multiple RP constructs are made for each WT protein.

Here, we devise a computational method, the random permutant (RP) method, for assessing the response of protein structures to packing perturbations. The RP method (Figure 1) repacks random permutations of the protein sequence onto the protein backbone. Thus, the backbone structure of the RP protein remains the same as that of the original protein but large side-chains may be replaced by small side-chains or vice versa and the packing within the protein is perturbed. This perturbed packing is then assessed for robustness using folding simulations of coarse-grained structure-based models (SBMs) (Noel and Onuchic, 2012) of both the original (wild-type or WT) and the RP proteins.

SBMs (Noel and Onuchic, 2012) have funneled energy landscapes (Bryngelson et al., 1995; Onuchic et al., 1997; Nymeyer et al., 1998) because they encode the protein structure in their potential energy functions. They have been successfully used to reproduce folding routes, free energy barriers and intermediate structures and populations in diverse proteins (Chavez et al., 2004; Hills and Brooks, 2009; Zhang and Chan, 2010). A random permutation of the sequence is not likely to create a foldable protein if all physical interactions are encoded in the potential energy function. The SBM used here encodes the folded or native protein structure by including attractive interactions between all atoms that are close in this structure irrespective of their chemical nature. Additionally, no attractive non-native interactions are encoded in the model. The SBM can also be used to enforce folding to the same backbone structure as the WT protein. This ensures that the only differences between the WT and the RP SBMs are the number and the position of attractive interactions (or contacts) present in the folded state, which are determined by the positioning of the side-chains in the specific RP. Thus, the perturbed packing created by a random permutation results in a reorganization and change in the number of contacts within the WT protein structure. It is the effect of this reorganization that is probed using folding simulations of SBMs. The SBM used here is also coarse-grained to a single Cα bead per residue (Clementi et al., 2000) making it computationally efficient for performing multiple folding simulations.

**FIGURE 2:**
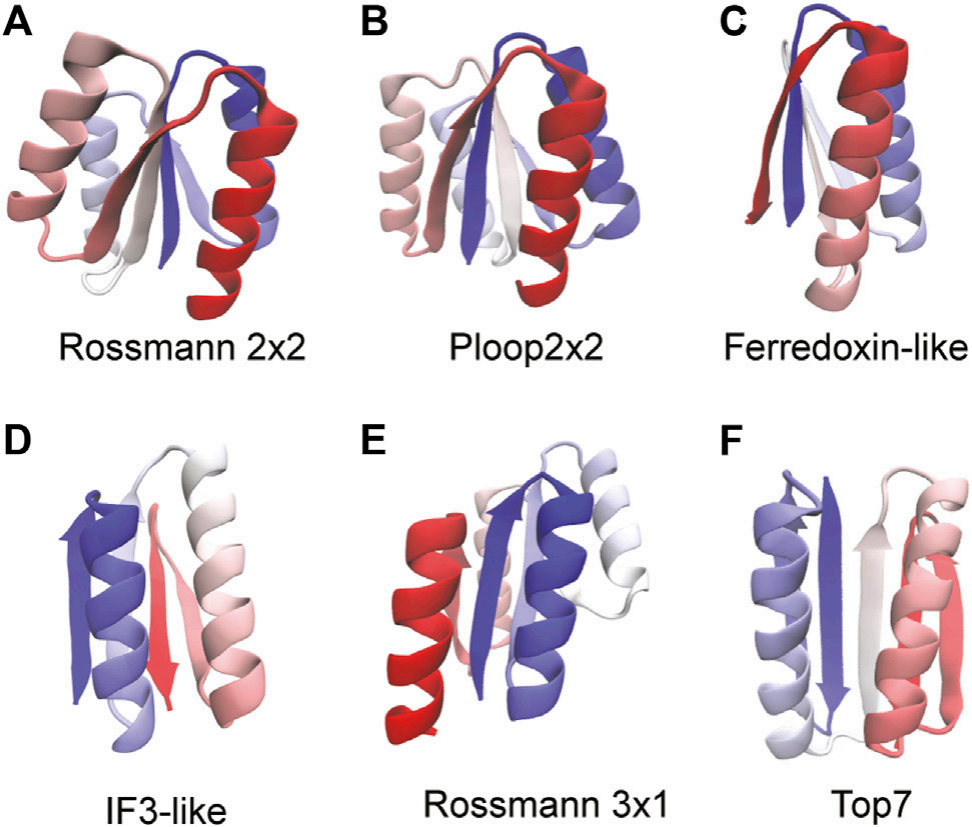
Proteins used to benchmark the RP method are shown. The proteins are colored from blue (N-terminus) through white to red (C-terminus). The folds of the proteins are stated below each structure. We mention here the sequence of secondary structural elements for each fold. **(A)** Rossmann 2x2: β-α-β-α-β-α-β-α. **(B)** P-loop 2x2: β-α-β-α-β-α-β-α **(C)** Ferredoxin: β-α-β-β-α-β **(D)** IF3: β-α-β-α-β-β **(E)** Rossmann 3x1: β-α-β-α-β-α-β-α **(F)** Top7: β-β-α-β-α-β-β. All protein structures are drawn using the program VMD (Humphrey et al., 1996). Proteins that have the same sequence of secondary structural elements fold to different tertiary structures and this can be seen by comparing **(A)**, **(B)**, and **(E)**.

Five ideal proteins, not containing functional “defects” in their structures and belonging to various super folds, have been synthesized (Koga et al., 2012). These proteins and their core folds have either two or four α-helices interspersed between a four stranded β-sheet with neighboring strands arranged in either parallel or anti-parallel orientations. The α/β Rossmann fold, often described as a doubly-wound three-layer sandwich (Medvedev et al., 2019), is an extremely common fold observed predominantly in metabolic proteins (Medvedev et al., 2021). The classical Rossmann fold has two pseudosymmetric units making a six stranded parallel β-sheet with a characteristic crossover between β strands 3 and 4. However, the Rossmann structures studied here have a four stranded parallel β-sheet with Rossmann 2X2 (Figure 2A) having two pseudosymmetric units of β-α-β with a crossover between strands 2 and 3. The Rossmann 3X1 protein (Figure 2E) has one three-stranded unit and another one-stranded unit with a crossover between strands 3 and 4. Despite the difference in their tertiary structures, both proteins have the same order of secondary structural elements (β-α-β-α-β-α-β-α) and, coincidentally, also the same number (99) of amino acids. The P-loop or phosphate-binding loop is a common motif in proteins that are associated with phosphate binding (Romero Romero et al., 2018). This protein family is possibly the most ancient and abundant enzyme family (Longo et al., 2020). The P-loop proteins are α/β three layer sandwiches nominally similar to the Rossmann proteins but with entirely different tertiary structures (Koga et al., 2012). The designed P-loop 2X2 protein (Figure 2B) has a β-α-β-α-β-α-β-α secondary structure with 101 amino acids. The Ferredoxin fold from the α+β protein class is a common fold with around 60 superfamilies which function in translation (Fox et al., 2014), electron transfer reactions (Krishna et al., 2006), and also as structural proteins (Krishna et al., 2006). The Ferredoxin protein (Figure 2C; 76 amino acids) has a signature β-α-β-β-α-β secondary structure with a four stranded antiparallel β-sheet covered on one side by two α-helices (Koga et al., 2012). IF3 is an α+β fold made of about eight superfamilies (Fox et al., 2014) with a majority of its proteins functioning as translation initiation factors. The IF3 protein (Figure 2D; 72 amino acids) has the following order of secondary structural elements: β-α-β-α-β-β with the strands arranged in a mixed β-sheet. Finally, we study Top7 (Kuhlman et al., 2003) (Figure 2F; 92 amino acids) which has a non-natural β-α-β-β-β-α-β secondary structure with the five strands arranged in an anti-parallel β-sheet. We apply the RP method to these proteins in order to understand if their ideal structures (Koga and Koga, 2019) imply a robustness to packing perturbations and natural protein-like folding.

The RP method has been devised for assessing the sensitivity of protein structures or backbones to packing perturbations. In the RP method, these packing perturbations are brought about by changes in protein contact maps that occur upon a random permutation of the sequence. As stated earlier, random permutation of sequences is unlikely to produce foldable proteins. Consequently, the RP method cannot be used to examine sequences for either stability or foldability. It is also not intended to improve sequences. Other methods that use evolutionary information (Goldenzweig et al., 2016) or directed evolution (Ben-David et al., 2019) can be used to improve the sequences of designed proteins (Listov et al., 2021).

The rest of the content is organized as follows: The steps required to implement the RP method, the details of proteins, SBM and simulation parameters as well as a description of the simulation analyses are given in the *Methods* section. The application of the RP method to two natural proteins: S6 of the Ferredoxin fold and ecoRNase-H are given in the first subsection of the *Results and Discussion* section. The next three subsections describe results obtained from the application of the RP method to the six *de novo* designed proteins. The final subsection discusses potential applications of the RP method when folding simulations are not possible. A summary of the method, salient results, and contexts in which the method is likely to be useful are listed in the Conclusions section.

## Methods

### An Overview of the Method

The random permutant (RP) method (Figure 1) repacks a randomly permuted WT sequence onto the WT backbone. This ensures that the backbone structure of the RP protein is preserved while redistributing the amino acid side-chains. Thus, protein packing may be perturbed through the replacement of larger side-chains by smaller side-chains or vice versa. This perturbed packing is then probed using folding derived parameters such as contact maps, barrier heights, folding routes, the population of intermediates, etc. We define robust protein backbones or scaffolds as those in which random permutations do not significantly affect folding. In proteins whose folding is perturbed by random permutations, the method can be used to find regions within the protein which are sensitive to changes in packing. The specific pattern of residues in a protein sequence is such that it makes the protein foldable, stable and soluble. Thus, the RPs as folding proteins are only theoretical objects constructed to probe their packing. None of the RPs are likely to fold in an experiment if at all they are soluble.

To implement the method, a protein structure (PDB file) is selected. The positions of the backbone atoms are preserved while the side-chains (all other atoms) are removed from this PDB file. The amino acid sequence of the protein is then randomly permuted. This randomized sequence is then rebuilt onto the original or wild-type (WT) protein backbone using a side-chain conformation prediction program, SCWRL4 (Krivov et al., 2009), which ignores specific attractive and repulsive interactions between sidechains. Several such RPs are constructed for each protein. The PDB files of these RPs generated by SCWRL4 are then used to perform folding molecular dynamics (MD) simulations using structure-based models (SBMs) coarse-grained to one Cα bead per residue (Clementi et al., 2000). Cα coarse-grained models are particularly appropriate for use with the RP method because some random permutations create clashes between atoms in the interior of the proteins while other permutations might create loosely packed protein cores. The coarse-graining filters out the detailed effects of this over- or under-packing such that the effects of only the strongest packing perturbations remain.

### Proteins Used in This Study

The RP method was first applied to two natural proteins, namely, S6 (PDB ID: 1RIS) and ribonuclease H (RNAse-H; PDB ID: 2RN2). To provide a contrast to these proteins and to avoid functional features such as loosely packed or strained loops, kinked α-helices and bulged β-strands that may introduce additional sensitivity to packing perturbations, we then chose to benchmark the RP method using six designed proteins (Kuhlman et al., 2003; Koga et al., 2012). These proteins were designed with only the basic architecture of the fold and do not possess additional functional elements (Figure 2). NMR structures (PDB IDs: 2KL8, 2LV8, 2LN3, 2LVB, 2LTA) of five of these proteins exist, but SBMs derived from a single NMR MODEL do not always accurately represent folding dynamics (Jiang and Hansmann, 2012). So, we used the original computationally designed coordinates of the proteins, available at http://psvs-1_4-dev.nesg.org/ideal_proteins/ (Koga et al., 2012) (Figures 2A–E) to perform the SBM MD simulations. These coordinates are now also given in the Supplementary Material. These model coordinates are similar to those in the experimentally derived NMR structures (Koga et al., 2012). The crystal structure of Top7 (Figure 2F; PDB ID: 1QYS) was used after fixing missing atoms (49 in number) using the program spdbv (Guex and Peitsch, 1997).

### Details of Random Permutant Construction

The sequence of the WT protein was randomly permuted. After deleting the side chain atoms (all atoms except the backbone atoms: N, Cα, C, and O) of the WT residues from the PDB file, the permuted sequence was assigned to the WT backbone atoms by editing the PDB file. This PDB file was then used as an input to SCWRL4, which determines the best orientation of the new sidechains and outputs a PDB file with the preserved coordinates of the backbone atoms and the generated coordinates of the permuted side-chain atoms. SCWRL4 uses a backbone-dependent rotamer library for its predictions (Krivov et al., 2009). Further, the SCWRL4 energy function has a simple repulsive steric energy term to reduce overlaps between predicted sidechain atoms but does not include terms that account for either attractive interactions between similar atoms (either hydrophobic or polar or charges of the opposite sign) or additional repulsive interactions between unlike atoms or like charges (Krivov et al., 2009). Randomizing the sequence is likely to bring together at least some residues whose physico-chemical interactions are not favorable. Since SCWRL4 ignores such interactions while repacking the protein, the RP structures can be constructed without under-packing “chemically” destabilized regions. We found that SCWRL4 was able to repack all generated sequences onto the protein backbones. The secondary structure of the RPs remains the same as the WT protein because the positions of the backbone atoms are preserved. Random permutation also preserves the amino acid composition and thus the average side-chain size also remains constant. Because of these properties, the RPs can be used to study the effect of packing perturbations on protein folding.

### Structure Based Models

As the name suggests, the potentials of SBMs (Nymeyer et al., 1998; Noel and Onuchic, 2012) of proteins encode the protein structure (as present in the PDB file). As stated earlier, a Cα-SBM is used here for performing the simulations. The details of the energy function of this commonly used SBM can be found elsewhere (Clementi et al., 2000). Here we summarize the various terms that encode the structure and allow it to fold and unfold. The minima of all potential energy terms are at folded distances (or angles) and these values can be calculated using the coordinates of the Cα atoms in the PDB file. Backbone bonding interactions are represented through strong harmonic bond and angle terms between adjacent Cα atoms. Secondary structure is encoded through the cosine of the dihedral angle between four adjacent Cα atoms. Cα atoms which are close (see the next section for definition of “closeness”) in the protein structure are defined to be in contact. Contacting Cα atoms have a 10–12 Lennard-Jones-like interaction between them while all other non-interacting Cα atoms interact through the repulsive part of the Lennard-Jones interaction. The strengths of the dihedral, contact and non-contact interactions are similar, far lower than those of the backbone interactions and define the basic energy scale of the potential energy function. Electrostatic interactions can be part of the contact map but are not explicitly encoded. Overall, SBM folding simulations can account for effects that arise from chain connectivity (backbone interactions and to some extent the dihedral term), excluded volume (the non-contact term) and heterogeneity in packing (the contact interactions).

### Contact Calculations

The list of pairs of Cα atoms in contact is calculated by first removing all the hydrogen atoms from the PDB file (of WT or RPs). If at least one of the remaining non-hydrogen or heavy atoms from residue ‘‘i” is within a cutoff distance of 4.5 Å of at least one heavy atom of residue “j” and i and j are separated by at least three residues in sequence, then the Cα atoms of the residues i and j are defined to be in contact. The total numbers of residues and contacts for the natural proteins are given in Supplementary Table S1 and those for the designed proteins are given in Supplementary Table S2. Randomly permuting residues is likely to create or delete contacts. For instance, a contact between two large side chains (say TRP) could get deleted in an RP in which one or both of these large side chains are replaced by smaller side chains (such as GLY or ALA). Similarly, a region packed with small amino acids might gain contacts when these small amino acids are replaced by large amino acids. Regions where many contacts are lost or gained across several RPs, are regions which are sensitive to packing perturbations. It should be noted that the contact map used here defines only one contact between two Cα atoms even when many atomic contacts are present between the two residues that they represent. Thus, the effect of small to large mutations is reduced when such mutations only lead to an increase in the number of contacts (and not to the creation of a first contact) between two amino acids. Repacking non-WT sequences onto the protein backbone can create clashes between atoms and short contacts (Supplementary Figure S1). The contact map used here reduces the effect of this overpacking upon folding by not weighting Cα-Cα contacts and assigning only one contact between two Cα atoms even when many atomic contacts are present.

The RP method was tested with contacts calculated using two other cutoff distances of 5.5 Å and 6 Å. At higher cutoff values there are more contacts and the changes in contacts created by the side chain perturbations are fewer. Thus, these contact maps are not as sensitive to changes in packing upon random permutation. Other types of cutoff, screened and weighted contact maps have previously been used to simulate proteins and these could also be used with the RP method (Cho et al., 2009; Noel et al., 2012; Reddy and Thirumalai, 2015; Sinner et al., 2015; Yadahalli and Gosavi, 2016). The last subsection of the *Results and Discussion* section illustrates one such use. However, the same kind of contact calculations should be used for both the WT and the RPs and across proteins when a comparison is being made.

### Contact Maps

The contact list can be easily visualized by plotting a contact map, whose X and Y axes represent the residue index. A colored square plotted at (i, j) and (j, i) means that a contact exists between residues i and j in the protein. In some contact maps, the upper left triangle and the lower right triangle represent different types of contact maps. A difference contact map between the WT and a given RP shows the contacts gained and lost upon that specific random permutation. In such maps contacts common to the WT and the RP are colored in grey while contacts specific to the WT and the RP are colored in different colors. Such contact maps can be used to visually detect sensitive regions which gain and lose contacts. We also plot composite contact maps which pool contacts from several RPs. The color of each contact gives the number of RPs that it is present in. Here, such composite maps are plotted with a color scale going from white/yellow (contact present in zero to a few RPs) through red to blue (contact present in almost all to all RPs). Since the backbone hydrogen bonding interactions within and between the secondary structural elements are preserved across RPs, contacts which represent such interactions are blue while easily perturbed contacts are yellow. Thus, composite contact maps can also be used to visually detect regions which are variably packed across the RPs.

### Molecular Dynamics Simulations of the Structure-Based Models

The folding simulations of the SBMs were performed using the GROMACS v4.0.7 program suite (Hess et al., 2008). The basic energy and length scales of the SBM can be set to any convenient value. Since we simulate the SBM using GROMACS, the internal units of this package, namely 1 kJ/mol and 1 nm are used in the SBM. The SMOG webserver (Noel et al., 2010b) was used to generate the input (.top and .gro) files for the simulations using the PDB files and the contact lists. A stochastic dynamics integrator was used to run the simulations with a time step of 0.0005 ps. The folding temperature, T_f_, is that temperature at which the folded and the unfolded ensembles are equally populated, multiple transitions occur between these ensembles and the transition region is sufficiently sampled. All simulations were performed close to T_f_ until at least 15 (un)folding transitions were observed. The values of the T_f_ for the WT and the RPs of the designed proteins are given in Supplementary Table S1.

### Simulation Analyses

SBMs encode structure through attractive interactions between contacting residues. These contacts form and break during folding transitions and so, the fraction of formed native contacts (Q) is often used as an order parameter to study folding (Chavez et al., 2004; Cho et al., 2006; Jackson et al., 2015). Since the only difference between the WT and the RPs is the number and the position of native contacts, we also use Q as an order parameter to understand changes in folding barrier heights, the presence or absence of folding intermediates, etc. A contact between residues i and j is said to be formed (*q*_*ij*_ = 1) in a given snapshot of the simulation trajectory when the distance between the Cα atoms, r_ij_, is less than 1.2 × d_ij_. Here, d_ij_ is the distance between the Cα atoms in the folded state. Unformed contacts have *q*_*ij*_ = 0. For a given snap shot, *Q* = ∑q_ij_/M, where M is the total number of contacts in the contact map of the protein. When the protein is unfolded, most *q*_*ij*_ = 0 and Q is close to 0. When the protein is folded, most *q*_*ij*_ = 1 and Q is close to 1. Snapshots with the same Q are collected and averaged over to calculate quantities such as the scaled free energy (ΔG/k_B_T_f_), average contact maps, etc. These quantities are functions of Q and they can be used to understand the populations (scaled free energy) and the structural details (average contact maps) of the partially folded ensembles. The scaled free energy profile (FEP) is calculated by creating a histogram of the number of protein snapshots that have a given Q. The FEP is the negative logarithm of this population histogram. The folded and the unfolded ensembles have the same scaled free energy in the FEPs because the simulations are performed close to T_f_ and are reweighted using single histogram reweighting (Ferrenberg and Swendsen, 1988) to T_f_. The binned snapshots at each Q are also used to construct and plot average contact maps at a given Q (or amount of foldedness of the protein). In such maps each contact (q_ij_) is colored according to how formed it is in a given Q-ensemble, i.e., the color is a function of *C*_*ij*_ = ∑q_ij_/N_Q_ where, as before, *q*_*ij*_ = 1 if the contact is formed in a given snapshot and *q*_*ij*_ = 0 if it is not formed. The summation is over all snapshots at a given Q (and not over all contacts) and N_Q_ is the total number of snapshots at a given Q.

### Identifying Robust Proteins and Sensitive Regions

The robustness or sensitivity of the structure and packing of a given protein is assessed by simulating multiple RPs and comparing their FEPs and average partial contact maps to each other. A structurally robust protein has the following three features: 1) The folded and the unfolded ensembles of both the RPs and the WT are located at similar Q values in the FEP. 2) The barrier heights of the RPs and the WT are within 2 k_B_T_f_ of each other. This empirical cutoff free energy was chosen after observing the variability between WT and RP FEPs of several proteins. However, similar numbers have previously been used to determine when differences in free energy barriers calculated using the same SBM are significant enough to imply that functional residues affect folding (Gosavi, 2013). FEPs of proteins with sensitive regions not only show large fluctuations in barrier height but a varying population and number of intermediate ensembles. 3) The folding routes of the WT and the RPs are similar. A change in folding route does not necessarily indicate that a protein is non-robust from the standpoint of folding, i.e., an RP can fold by a different route without a change in the position of the folded and unfolded ensembles, a large change in barrier height or the population of intermediates. However, a change in folding route usually indicates that one part of the protein has lost several contacts while another has gained contacts and thus the protein is likely to have regions that are sensitive to random permutation. Finally, the folding of non-robust proteins do not meet one or more of the above criteria and are thus sensitive to structural perturbations caused by random permutation.

### How Many Random Permutants Should to Be Simulated?

More information can be obtained about the protein structure if more RPs are simulated. However, there are N! permutations of an N residue protein sequence, each with a different contact map, and computational and time constraints do not permit many simulations. We have some evidence from previous contact map analysis of the RNAse-H proteins that contact patterns that emerge at five RP contact maps did not change up to 200 RP contact maps (Yadahalli and Gosavi, 2017). Since the main application of the RP method that is explored here is choosing designed protein structures, we make the following argument: for a given protein redesign about 5–10 protein sequences may be synthesized and tested (Koga et al., 2012). Each of these sequences can be thought of as having a different contact map. So, a structure should be insensitive to at least 5–10 contact map perturbations. That being said, a bad sequence to structure fit, which for the RP method means an unbalanced contact map pattern, may make even a marginally robust structure sensitive to random permutation. On the other hand, the SBM simulations performed for the RP method are coarse-grained and use unweighted contact maps, thus retaining only the largest perturbations in the simulations. In this background, the RP method is likely to be most predictive for rejecting protein scaffolds for redesign. Here, we chose to simulate five RPs because this number is computationally feasible and because any structures that are non-robust with only five RPs should be rejected.

## Results and Discussion

### A Summary of the Random Permutant Method and Criteria for Structural Robustness

An RP is constructed by repacking a randomly permuted WT sequence onto the WT backbone (Figure 1). This structural perturbation preserves the WT backbone and the volume of the WT protein chain while perturbing packing through the shuffling of large and small side-chains. The first five RPs that are output from a random permutation generator are then used to perform folding simulations using coarse-grained Cα-SBMs. The coarse-graining filters out the details of the packing perturbations, retaining only the strongest effects. A robust protein backbone is defined to have the following three features: 1) The folded and the unfolded ensembles of the RPs have the same level of “foldedness” as the WT. 2) The folding barriers of the RPs are similar in height and shape to those of the WT. 3) The folding routes of the RPs are similar to those of the WT. The folding of proteins whose structures are sensitive to packing perturbations do not meet one or more of the above criteria.

### Natural Proteins Can Fold Cooperatively Despite Having Non-Robust Scaffolds

**FIGURE 3:**
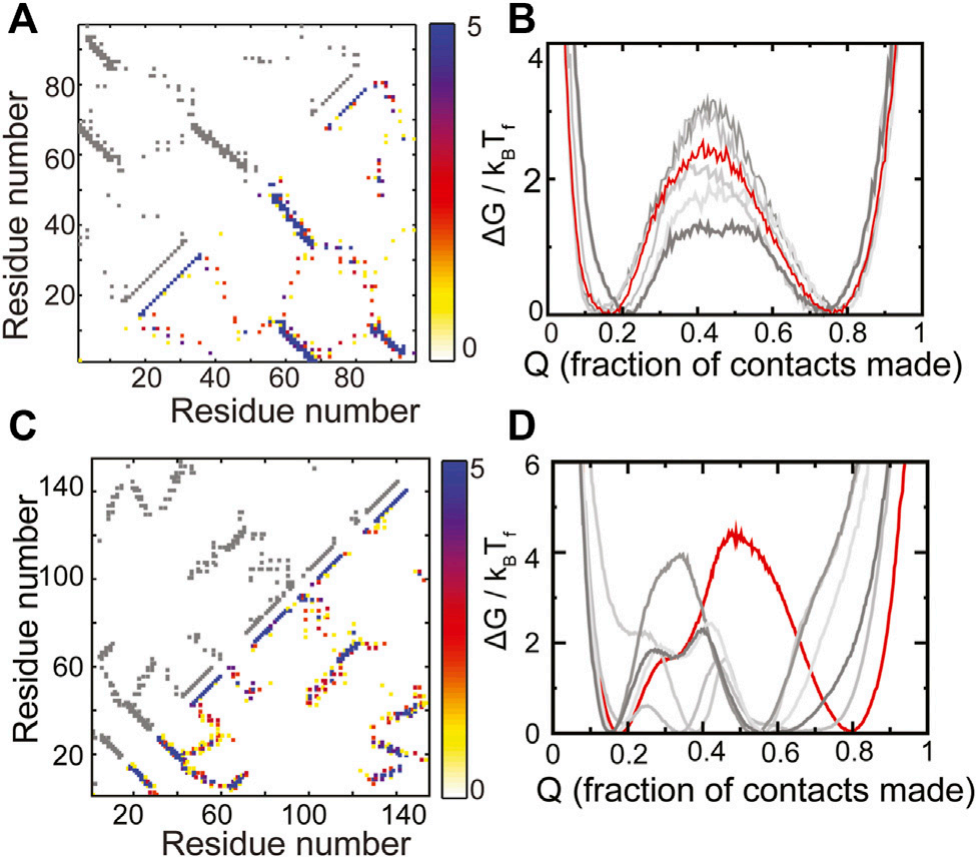
RPs of evolved proteins show diverse properties. **(A,B)** S6 **(C,D)** ecoRNase-H. **(A,C)** The contact map of the WT is shown in grey in the upper left triangle. The composite contact map of five RPs of the protein is shown in the lower right triangle. The color represents the number of RPs that a given contact is present in with the color scale being given on the right. **(B,D)** Scaled free energies of the WT (red) and the five RPs (different shades of grey) are plotted as a function of the fraction of native contacts of the individual proteins. **(B)** S6 folds like the Ferredoxin protein (Figures 2C, 4F). No intermediate (a dip in the barrier) is populated and the unfolded and the folded ensembles are similar across the WT and the RPs. **(D)** ecoRNase-H behaves differently from S6. The FEP of the WT is mostly cooperative with only two populated minima, none of the RPs fold completely and several intermediates are populated. Thus, although nature seems to have evolved an optimal packing, this packing is special and not reproducible in an RP. Panels **(A)** and **(B)** contain replotted data from our previous work (Yadahalli and Gosavi, 2014; Copyright 2013 by John Wiley and Sons, Adapted with permission).

The RP method was previously applied ad hoc to two natural proteins, S6 (Yadahalli and Gosavi, 2014) and *E. coli* ribonuclease-H (Yadahalli and Gosavi, 2017) (ecoRNAse-H). Here, we extend those results and place them in the context of choosing scaffolds for protein design. The protein S6 belongs to the Ferredoxin fold (Fox et al., 2014) and has the following sequence of secondary structural elements present along its chain: β-α-β-β-α-β. The contact map of WT S6 and a composite contact map (see *Methods* for a definition) of five RPs are similar and show no major loss or gain of contacts (Figure 3A). As expected from this composite contact map, the folding of the RPs of S6 is similar to that of WT S6 (Figure 3B). Specifically, the unfolded and the folded minima of the RPs and WT S6 are at similar values of Q or the “foldedness of the protein”, all the free energy barriers are within 2k_B_T_F_ of each other and no apparent intermediate is visible. Thus, by our criteria, S6 has a robust structure and sequences which have diverse patterns of amino acid sizes will be able to fold to the S6 backbone.

ecoRNase-H is also composed of α-helices and β-strands (contact map in Figure 3C). However, previous analyses showed that a group of contiguous helices within the protein (called the CORE) lose several contacts upon random permutation (Yadahalli and Gosavi, 2017). This is because several tryptophans pack against each other in the CORE and random sequence permutations leads to the placement of at least some of these tryptophans in other regions of the protein. This loss in CORE contacts is expected to lead to at least some difference between the folding of WT ecoRNase-H and its RPs. In agreement, we find that the ecoRNase-H RPs do not fold completely (the folded minimum of the RPs is at a much lower Q than the folded minimum of the WT), that the barrier to folding is lower than that of the WT across RPs and that several intermediates are also populated in the RPs (Figure 3D). Taken together these differences between the folding of the WT and the RPs indicate that the scaffold of ecoRNase-H is sensitive to packing perturbations.

Natural single domain-proteins often fold cooperatively, i.e., in an all or nothing manner populating only the folded and unfolded states and no intermediates (Jackson and Fersht, 1991). It has been hypothesized that this folding cooperativity is important because it reduces the interaction between partially folded proteins and in turn, protein aggregation (Dobson, 1999). Despite the difference in the behavior of the RPs of S6 and ecoRNase-H (Figure 3) it can be seen that both WT sequences can reach a fully folded state and fold cooperatively with a substantial barrier to folding. Thus, natural proteins can have scaffolds such as that of S6 which are not sensitive to changes in packing or scaffolds such as that of ecoRNase-H which can only accommodate specific patterns of amino acid size. ecoRNase-H possesses regions which upon being mutated can perturb folding substantially. Thus, “optimal” folding in a natural protein does not automatically imply the presence of a robust backbone that can be used as a starting point for protein redesign.

We note in passing that the present SBM of ecoRNase-H does not reproduce the experimentally determined order of folding events without the incorporation of contact weighting (Yadahalli and Gosavi, 2016). However, the height of the folding barrier in the weighted model is similar to that shown here (Figure 3).

### The Folding of Some Designed Proteins Is Insensitive to Packing Perturbations

In order to highlight the usefulness of the RP method in choosing protein scaffolds for protein redesign, we decided to apply the method to six previously *de novo* designed proteins (Kuhlman et al., 2003; Koga et al., 2012) and test the quality of their scaffolds. Similar to S6 and ecoRNAse-H, all these proteins are composed of both α-helices and β-strands (Figure 2). However, the backbones of these proteins have been designed such that they have near ideal secondary structural elements with no functional distortions which could make them sensitive to packing perturbations (Koga and Koga, 2019).

**FIGURE 4:**
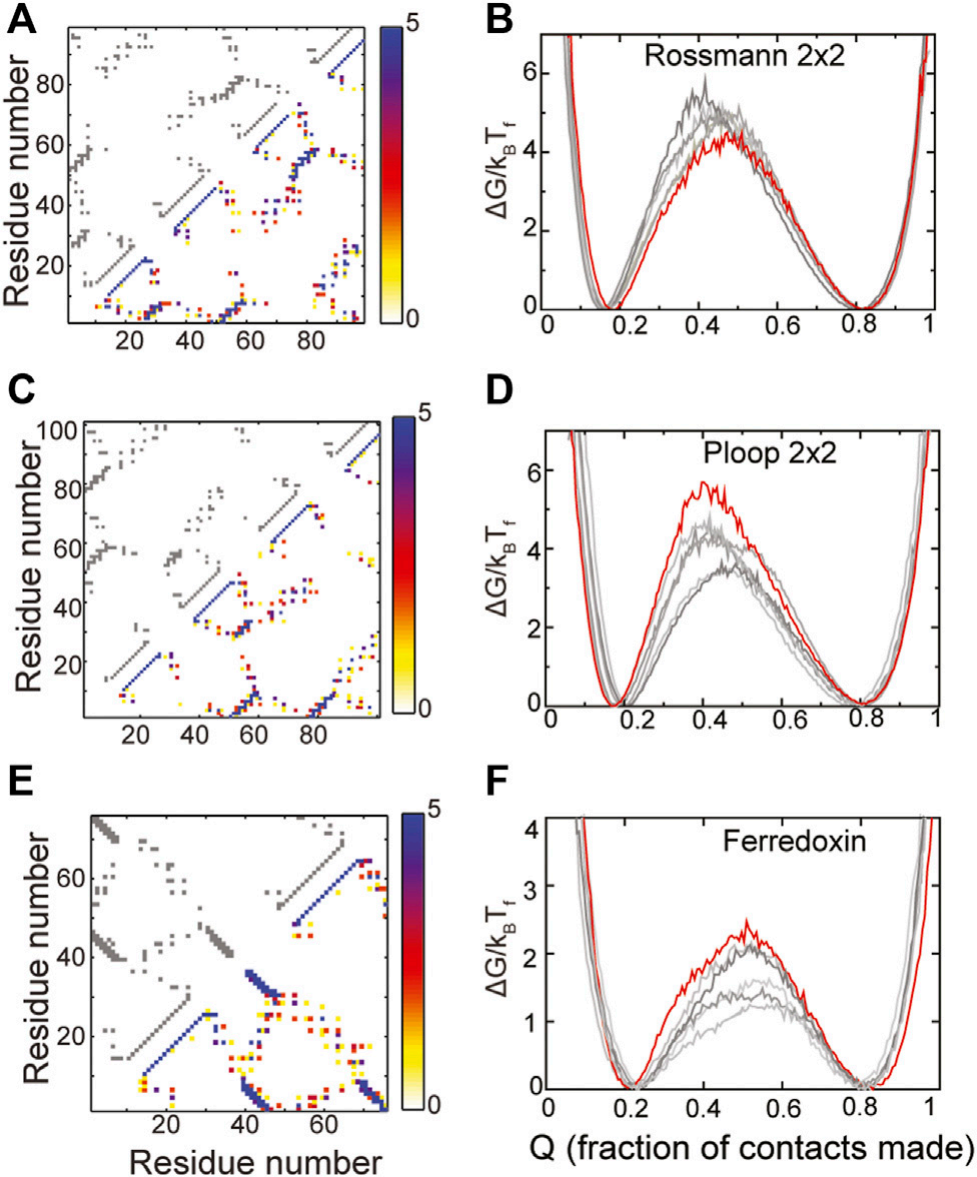
Robustly packed proteins. **(A,B)** Rossmann 2X2 **(C,D)** Ploop 2X2 **(E,F)** Ferredoxin-like. **(A,C,E)** The X and Y axes represent the residue number. A square present at (x, y) implies that the residues x and y are in contact. The contact map of the WT is shown in grey in the upper left triangle. A composite contact map (see *Methods*) of five RPs of the protein is shown in the lower right triangle. The color represents the number of RPs that a given contact is present in with the color scale being given on the right. As an example, a blue contact is present in all five RPs. Each of the RPs has the same backbone as the WT and thus most intra-α-helical contacts and inter-β-strand contacts are preserved across RPs and are blue. However, other long-ranged contacts depend on the specific RP and are thus red or yellow. **(B,D,F)** Scaled free energies of the WT (red) and the five RPs (different shades of grey) are plotted as a function of the fraction of native contacts of the individual proteins. The position of the unfolded (low Q) and the folded (high Q) minima and the barrier heights are similar. No clear intermediate, seen as a dip in the barrier, is populated.

The composite contact maps from five RPs of three of these designed proteins, namely those belonging to the Rossmann 2X2, P-loop 2X2 and the Ferredoxin folds are shown in Figures 4A,C,E. Although there is variability in regions of the contact map which depict helix-strand packing as well as other non-hydrogen bonding contacts, no clear loss or gain of contacts in a specific region of the protein can be seen. In agreement with these observations, the folded and the unfolded basins of the RPs of these proteins are similar to those of the WT, the RPs do not populate intermediate states and their barriers are within 2k_B_T_F_ of the WT barrier. Additionally, the folding routes of the RPs are also similar to those of the WT. Thus, these “ideal” proteins are indeed robust to packing perturbations.

The RPs of the Rossmann 2X2 protein have higher barriers to folding than the WT though by a small margin (∼1.5 k_B_T_F_) indicating that the packing in this fold could be improved. However, given the small increase in barrier height, we did not analyze the RP contact maps further to identify contacts whose presence increases the barrier. The P-loop 2X2 and Ferredoxin WT proteins also have the highest barriers among the RPs indicating that they are well designed and their packing is better than at least the five randomly chosen RPs shown in Figures 4D,F. The natural protein S6 (Figures 3A,B), which is robust to packing perturbations, also folds to the Ferredoxin fold (compare contact maps in Figures 3A, 4E). Thus, the Ferredoxin fold is able to stay robust despite the addition of functional features. In fact, the Rossmann, Ferredoxin and P-loop folds are superfolds (Orengo et al., 1994; Magner et al., 2015) because they can accommodate more sequences than many other natural folds (Longo et al., 2020) and are more tolerant to amino acid mutations. Thus, these folds are also termed highly designable (Helling et al., 2001; England and Shakhnovich, 2003). We next examine the IF3 fold.

### Alternative Folding Routes Can Be Detected Using the Random Permutant Method

**FIGURE 5:**
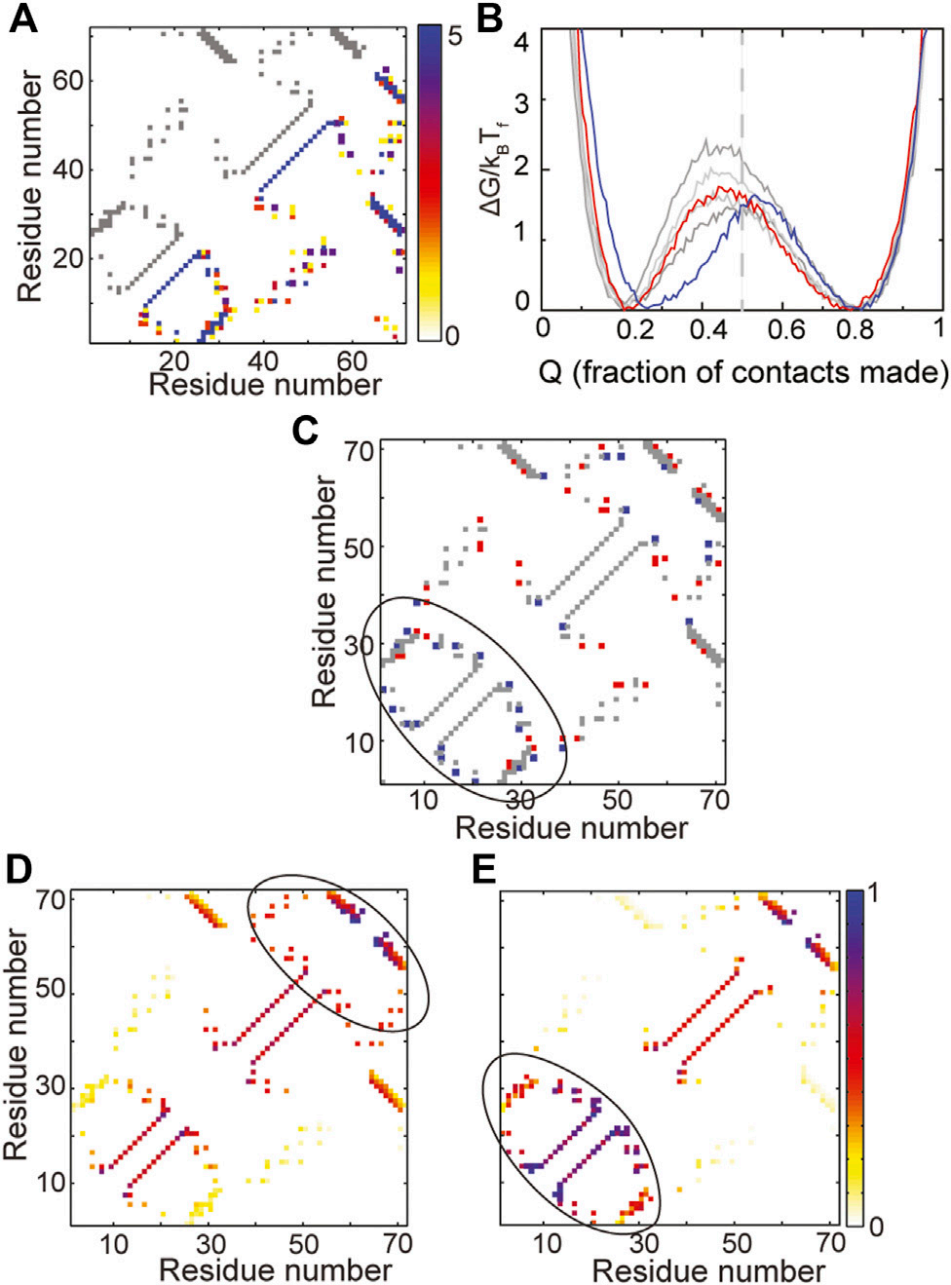
A change in folding route is observed in IF3. **(A)** The contact map of the WT is shown in grey in the upper left triangle. The composite contact map of five RPs of the protein is shown in the lower right triangle. The color represents the number of RPs that a given contact is present in with the color scale being given on the right. **(B)** Scaled free energies of the WT (red) and four RPs (different shades of grey) are plotted as a function of the fraction of native contacts of the individual proteins. Although the free energy barriers are large across RPs, the primary folding route of one RP (FEP shown in blue) changes. The unfolded basin of this RP is also more folded than the unfolded basins of the other RPs and the WT. **(C)** A difference contact map of the WT and the RP with the changed folding route is shown to determine regions which are differently packed in the RP. Grey contacts are common to both the WT and the RP. Red contacts are present only in the WT and blue contacts are present only in the RP. The RP has many more contacts in the N-terminal region (marked by the black ellipse). **(D,E)** Average contact maps of the WT **(D)** and the RP with changed folding route **(E)** are shown at Q ∼ 0.5. The colors depict the probability of contact formation and the color scale is given on the right. A darker (e.g., blue) color implies a more formed contact while a lighter (e.g., yellow) color implies a less formed contact. The WT folds through a C-terminal route while the RP folds *via* an N-terminal route.

The WT contact map as well as the composite contact map of five RPs of the IF3 fold are shown in Figure 5A. As with the previous three robust folds, there is variability in the contact map but no clear loss or gain of contacts is seen in any given region. For the most part, the folded and the unfolded basins of the RPs are similar to those of the WT, no intermediates are populated and the folding barriers are within 2k_B_T_F_ of each other. Thus, IF3 is also likely to be robust to packing perturbations.

However, a study of the average folding routes of the RPs shows that one of the RPs (blue free energy profile in Figure 5B) folds by a different folding route than the WT. The unfolded basin of this RP is also slightly more folded than that of the WT. The IF3 protein (Figures 2D, 5A) is made of the following secondary structural elements β-α-β-α-β-β. It is apparent from both the contact map (Figure 5A) and order of secondary structural elements that IF3 is not symmetric. Multiple accessible folding routes are often seen in proteins with repeating units or some other symmetries (Klimov and Thirumalai, 2005; Chavez et al., 2006). So, it was surprising to see a second folding route in IF3 and we decided to investigate its origins by identifying regions of the RP which were differently packed than the WT. A difference contact map of the WT and the RP (Figure 5C) shows that the RP gains contacts in the N-terminal region while losing contacts in the rest of the protein. In agreement with this observation, it can be seen that the WT folds C-terminus first (Figure 5D) while the RP folds N-terminal first (Figure 5E). This implies that two different folding routes can be present at the average amino acid size and distribution present in WT IF3. When the N-terminus is better packed, the protein folds by the RP route while when the C-terminus is better packed, the protein folds by the WT route. It may be possible to switch between the two routes in the WT by making a few small to large hydrophobic mutations in the N-terminal region while concomitantly making large to small mutations in the C-terminal region. It may also be possible to change the amino acid size distribution, increase the average amino acid size and repack the protein such that it folds more homogeneously from both the N- and C-terminal regions. This could lead to higher folding barriers and a more cooperative protein. However, whether this is possible within the limitations of the chemistry and sizes of the twenty amino acid alphabet is unclear.

Functional residues usually destabilize the protein in order for there to be a driving force for function (such as protein-ligand binding) (Giri Rao and Gosavi, 2016). An understanding of all accessible folding routes (Udgaonkar, 2008) and of ways to direct folding proteins between them will be useful when *de novo* designed proteins such as the IF3 protein need to be functionalized. Specifically, introducing destabilizing residues in the C-terminus may direct folding via the N-terminus but with smaller barriers to folding. On the other hand destabilizing N-terminal residues may lead to the population of an intermediate with only the C-terminus folded. In fact, such effects are already seen in the Rossmann 3X1 and Top7 proteins and we discuss these in the next section.

### The Random Permutant Method Can Be Used to Optimize the Packing of a Protein and Allow it to Fold Cooperatively

**FIGURE 6:**
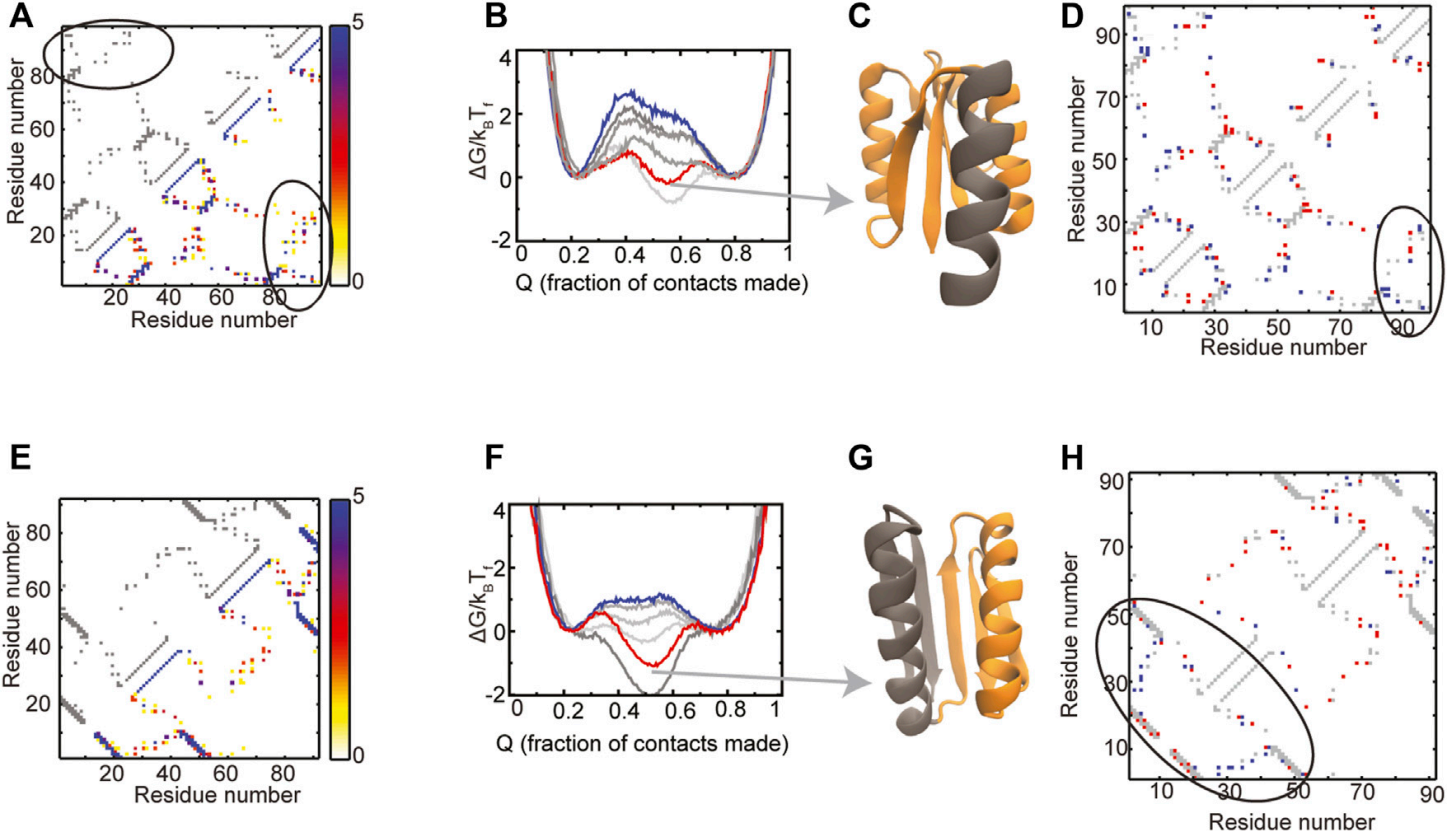
Proteins with populated intermediates. **(A–D)** Rossmann 3X1 **(E–H)** Top7. The contact map of the WT is shown in grey in the upper left triangle. The composite contact map of five RPs of the protein is shown in the lower right triangle. The color represents the number of RPs that a given contact is present in with the color scale being given on the right. The ellipses in **(A)** mark the contacts between the C-terminal helix and the rest of the Rossmann 3X1 protein. **(B,F)** Scaled free energies of the WT (red) and four RPs (different shades of grey) are plotted as a function of the fraction of native contacts of the individual proteins. The WT proteins have intermediate ensembles which are populated as much or more than the unfolded and the folded ensembles. Random permutation creates proteins which fold cooperatively with a single free energy barrier. The scaled free energy of the RP with the largest free energy barrier is shown in blue. **(C,G)** A representative structure from the intermediate ensemble is shown with the folded regions colored in orange and the unfolded regions colored in grey. **(D,H)** A difference contact map of the WT and the RP with the highest barrier is shown to determine regions which are better packed in the RP. Grey contacts are common to both the WT and the RP. Red contacts are present only in the WT and blue contacts are present only in the RP. **(D)** There is a variation between the number and position of contacts present between the C-terminal helix and the rest of the protein (enclosed by the black ellipse). **(H)** The N-terminal region (black ellipse) gains several blue contacts in the Top7 RP and earlier studies have shown that these contacts increase the free energy barrier in Top7 (Yadahalli and Gosavi, 2014). Panels **(E)** and **(F)** contain replotted data from our previous work (Yadahalli and Gosavi, 2014; Copyright 2013 by John Wiley and Sons, Adapted with permission).

The composite contact map of the RPs of the Rossmann 3X1 protein (Figure 6A) indicates that there is variability in the packing contacts of the C-terminal helix. A comparison of the composite map and the WT contact map (Figure 6A) indicates that only a few of these helix packing contacts exist in the WT. Folding free energy profiles (Figure 6B) show that the unfolded and the folded ensembles of the WT and the RPs are at similar positions. However, an intermediate is populated in the WT and one of the RPs. The WT has negligible barriers between the unfolded, intermediate and folded ensembles while the RPs have barriers of variable heights. Most of the protein is structured in the intermediate ensemble of the WT except for the C-terminal helix (Figure 6C). The difference contact map of the WT and the RP with the highest barrier (Figure 6D) shows both a small increase in the number as well as a repositioning of the packing contacts between the C-terminal helix and the rest of the protein. It is likely that these changes drive protein folding and C-terminal helix packing to occur concomitantly and promote folding cooperativity (Also, see Supplementary Figure 2 for average contact maps of WT and the RP at ∼55% “foldedness”). Based on these observations, we suggest two pairs of mutations which may increase the packing between the C-terminal helix and the rest of the protein, mimic the effect of the contacts gained in the RP and increase folding cooperativity. The first mutation, Ala88Ile, converts a small hydrophobic residue in the C-terminal helix to a large one and may increase the contacts of the C-terminal helix with the rest of the protein. However, an Ala to Ile mutation is also likely to reduce the local helical propensity. A second mutation, similar in nature to Ala88Ile, could be Leu92Phe. An alternative to this mutation is a Leu92Lys, which could increase helix-protein interactions if a salt bridge is formed between Lys92 and the spatially proximal Glu24. We next summarize our previous results from Top7 (Yadahalli and Gosavi, 2014).

There is variability in the helix-strand packing contacts among the RPs of Top7 in both the N-terminal and C-terminal regions (Figure 6E). However, many of these contacts exist in the C-terminal region of the WT, while only a few are present in its N-terminal region (Figure 6E). The folding free energy profiles of Top7 (Figure 6F) are similar to those of Rossmann 3X1 and show that an intermediate is populated in the WT but not in all RPs. As expected, the WT intermediate has a formed C-terminal region and an unformed N-terminal region (Figure 6G). The difference contact map of the WT and the RP with the highest barrier (Figure 6H) shows that the RP has lost contacts in the C-terminal region but gained them in the N-terminal region. This balancing of the packing allows both halves of the protein to form together and leads to cooperative folding. In previous work (Yadahalli and Gosavi, 2014), we had identified mutations that could promote such packing in Top7 by a different method and we list them here: V6I-L29F-I40F-F71V-I79V-F83V.

Overall, folding simulations can be used to identify if a given protein (WT) is cooperatively folding. When an intermediate is populated in the WT, the RP method can be used to understand if protein repacking will increase folding cooperativity. The method can also help identify contacts, the addition of which can reduce intermediate population and increase folding cooperativity.

### Experimental Folding Studies of *De Novo* Designed Proteins

The folding of Top7 has been characterized using varied experimental techniques (Scalley-Kim and Baker, 2004; Sharma et al., 2007; Watters et al., 2007; Li et al., 2021). It was shown that Top7 exhibits non-cooperative multiphasic folding involving multiple intermediate states. Recent single molecule studies have also shown that Top7 exhibits considerable mechanical stability and the intermediate states on the unfolding pathway likely involve nonnative structure (Li et al., 2021). To the best of our knowledge, there have been no published mutants of Top7 which show cooperative intermediate free folding. Single molecule mechanical unfolding of the Rossmann 2X2 protein using optical tweezers showed that the protein undergoes a slow transition between folded and unfolded states. This slowness was ascribed to the presence of high energy intermediates (Mehlich et al., 2020). In a separate study, the robustness of the Rossmann 2X2 protein was also examined by altering the packing of its core (Koga et al., 2020). It was shown that mutating up to ten larger hydrophobic residues to Valine still does not alter the protein’s foldability or stability. This suggested that in proteins designed using the Rosetta pipeline the local backbone structure may have a larger role in determining thermal stability than hydrophobic packing. The folding of the IF3-like protein was examined using a combination of experimental methods (Basak et al., 2019). The protein exhibits cooperative two state folding when denatured using GdnHCl, a salt. However, the protein remained folded in upto 9.25 M urea, an uncharged denaturant. Changing the sign of surface charges through mutation restored urea induced denaturation. In contrast to the absence of intermediates detected in denaturation by GdnHcl, several high energy states which exchange over long periods of time were detected using hydrogen-deuterium exchange NMR in water. Reducing the density of hydrophobic packing by mutating two large hydrophobic amino acids to smaller ones eliminates these high energy intermediates. Together these experiments suggested that the IF3-like protein had a dense network of electrostatic and hydrophobic interactions not usually present in natural proteins.

Overall, of the three designed proteins studied experimentally, all seemed to show the population of folding intermediates. It is known from several simulation studies that designed proteins tend to be more frustrated than natural proteins (Chavez et al., 2004; Zhang and Chan, 2010; Best et al., 2013; Jackson et al., 2015; Neelamraju et al., 2018), i.e., they populate several partially folded intermediates enroute to their final folded states. Frustration can either be energetic (sequence driven) or topological (structure driven). Sequence driven frustration can occur because the sequence-structure fit is not optimal (e.g., like charges interact in the folded state) or because non-native interactions are stabilized during folding. Topological frustration can be caused by structural defects: regions of the protein that do not have sufficient interactions or are overpacked. The RP method can only identify structural defects and is blind to sequence driven frustration. Experimentally, it is difficult to differentiate between sequence-driven and structure-driven intermediates. However, the population of intermediates can be reduced in the IF3 fold by mutations while the same has not been achieved thus far for Top7. This is in overall agreement with our conclusions about Top7 and IF3. Although different RPs of IF3 fold through one of two folding routes (Figure 5), they all fold cooperatively with a single free energy barrier and without the population of intermediates. However, the structure of Top7 is not robust (Figure 6) and it is likely that only carefully designed sequences will allow it to fold cooperatively and such a sequence has not been experimentally designed thus far. Finally, the structure of the Rossmann 2X2 protein is robust because multiple sequences or contact patterns still allow it to fold, albeit with the population of intermediates. A prediction of the RP method is that the Rossmann 3X1 protein will behave similar to Top7 and we hope experimental data on this protein will become available soon.

### The Random Permutant Method and *De Novo* Designed Backbones

The overall goal of the RP method is to understand the structural robustness of protein backbones. Recently machine learning (ML) methods have been used to design protein backbones (Anand et al., 2019) as well as proteins (Anishchenko et al., 2021). The RP method can be used to understand if the structures of such ML designed backbones are more robust to structural perturbation than those of previously designed backbones which were designed mostly using geometric principles (Koga et al., 2012).

As stated in the previous section, topological frustration can be caused by structural defects in the final folded structure. By encoding the native structure, SBMs reduce energetic frustration and can be used to isolate the effect of topological frustration on the folding of proteins. It was recently shown using a frustration density parameter that the SBM of Top7 was more frustrated than the SBM of S6 (Neelamraju et al., 2018). However, a similar analysis has not yet been performed on the other designed proteins simulated in this study. It would be interesting to understand if the designed proteins that are classified as robust according to the RP method also have lower values of the frustration density parameter.

### Role of Non-Native interactions in the Folding of Designed Proteins

Sequence driven frustration can cause non-native interactions to contribute significantly to the folding of designed proteins (Chavez et al., 2004; Zhang and Chan, 2010; Best et al., 2013; Yadahalli et al., 2014; Jackson et al., 2015) promoting the population of intermediates. In order to structurally characterize such intermediates, it is important to understand if a specific pattern of residues present in the WT protein or in the RPs is likely to promote trapping beyond that seen in simulations which encode only native interactions. The present version of the RP method ignores such non-native trapping and focuses on assessing the robustness of only the final folded structures. However, some intermediates such as those seen in Top7 (Scalley-Kim and Baker, 2004; Watters et al., 2007) may be caused by the non-robustness of the folded structure (Zhang and Chan, 2009; Yadahalli and Gosavi, 2014). Thus, by allowing the selection of a robust scaffold, the RP method may promote designed protein foldability by reducing the population of some intermediates.

Native interactions play a dominant role in the folding of natural proteins and thus, Q, the fraction of native contacts, is a good reaction coordinate for understanding protein folding not only in SBM simulations, but also in atomistic simulations (Best et al., 2013; Noel et al., 2016). However, unlike in natural proteins, non-native interactions play a significant role in the folding of the designed protein α_3_D in atomistic simulations (Best et al., 2013) and may do so in other proteins as well. Since the only difference between the WT and the RPs is the number and position of native contacts, the fraction of native contacts, Q, is used here for analyzing the folding simulations. However, given the potential role of non-native interactions, the analysis prescribed here, based on Q, should be treated as an ad hoc recipe for assessing the robustness of designed protein structures without direct correlation to observable quantities. Since SBM folding simulations even without non-native interactions are computationally intensive we next discuss ways of analyzing repacked protein contact maps.

### Understanding Protein Packing Without Folding Simulations

The same procedure used in the RP method can also be used to repack protein backbones with non-RP sequences and we first discuss such protein repacking. Since alanine is the smallest residue with a side-chain, repacking with a poly-alanine sequence provides information about the minimal set of contacts that are present given a backbone no matter the residue identity (assuming few to no glycine residues). Arginine and tryptophan are large residues with different shapes and flexibilities, so poly-arginine and poly-tryptophan repacking will likely give all possible contacts that can be made given a backbone. It should be noted that such contact maps need not always promote folding cooperativity: they may over-pack some protein regions, allowing them to fold earlier than the rest of the protein and populate folding intermediates. Protein repacking can also be performed with random sequences. This ensures that there is sufficient amino acid size diversity to find diverse packing defects. For the proteins simulated here, results from pilot simulations performed with such repacking did not differ substantially from results using random permutations. We chose random permutations for protein repacking because random permutation preserves the average amino acid size of a sequence. However, for protein sequences with low amino acid diversity, repacking with random sequences may be preferable. Packing the protein with a single side chain (such as leucine or isoleucine) whose size is closest to the average amino acid size of the WT sequence will preserve backbone volume but with homogeneous packing. A comparison of such a contact map to the WT contact map can be used to identify regions which gain or lose contacts in the WT. Regions which gain contacts in the WT have larger side chains, which may be lost upon random permutation. Such regions are well packed in the WT but are likely to be sensitive to packing perturbations. Sensitive regions can also be identified by a comparison of the composite RP and the WT contact maps. Regions of the composite contact map in which many contacts are present, each of which exists in only a few RPs, are regions whose packing depends on the specific sizes of the amino acids or the sequence. The packing in such regions is likely to be perturbed easily. If the WT has few contacts in such regions, then it is likely that folding will stall at such regions and intermediates will be populated.

Sensitive regions which have few and variable contacts upon random permutation can breathe, breaking the few contacts, allowing other parts of the protein chain to thread through the resulting cavity and causing topological frustration and misfolding (Neelamraju et al., 2018). A possible way to detect such regions without calculating contact maps is to detect protein cavities (Tan et al., 2013; Benkaidali et al., 2014). However, a detailed analysis of how cavity sizes change and correlate with contacts and contact numbers upon random permutation is needed before they can be used as a readout of sensitive regions.

We next summarize previous analysis of ecoRNase-H and its homologs (Yadahalli and Gosavi, 2017) performed using only RP contacts. This analysis was performed using weighted contacts (Yadahalli and Gosavi, 2017), but the procedure should be applicable to other types of contact maps. As can be seen from Figures 3C,D, the RPs of ecoRNase-H fold differently from each other and populate several intermediates indicating that the protein has regions whose packing is perturbed upon random permutation. In particular, an ecoRNase-H region termed CORE has several tryptophans packed against each other. These tryptophans get replaced by smaller residues upon random permutation leading to imperfect packing and loss of contacts in CORE. Difference contact maps such as those shown in Figures 1, 5C, 6D,H were created for over a hundred RPs in order to identify contacts gained and lost upon random permutation. These contacts were then partitioned into contacts gained or lost in the CORE and those gained or lost in the rest of the protein (termed the periphery). The normalized number of contacts lost in each of the regions (CORE or periphery) for each RP was then plotted versus the normalized number of contacts gained in the same region. If the two regions, CORE and periphery, have different packing properties, then the CORE points form a distinct cluster from the periphery points. However, if they have similar properties, then the clusters of the two regions overlap. As expected, the two clusters are distinct in ecoRNAse-H but overlap in other homologs (Figure 5 from Yadahalli and Gosavi, 2017). Such an analysis, which partitions the protein into different regions, can be used for comparing the packing in designed proteins with that of their natural or designed structural homologs. Since none of the designed proteins analyzed here that also have sensitive regions have obvious structural homologs, we do not perform such analysis here.

### Natural Sequence Landscapes and Protein Design

The RP method uses “foldability” criteria to help identify protein scaffolds which are resistant to packing perturbations. It is expected that many real sequences with diverse packing will be foldable to such scaffolds. In nature, there exist superfolds (Orengo et al., 1994; Magner et al., 2015), folds to which many diverse sequences fold. It has been hypothesized that multiple sequences can fold to superfolds because they optimize the thermodynamic stability of the protein (Orengo et al., 1994).

It was also shown that the mutational stability of a sequence (the number of mutant sequences which fold to the same structure) was correlated with its thermodynamic stability (Bornberg-Bauer and Chan, 1999; Sikosek and Chan, 2014). A landscape constructed with sequences which fold to the same structure, connected through single mutations, was funnel shaped, with the sequence with the maximum thermodynamic and mutational stability being at the bottom of the funnel. In this context, the RP method can be thought of as a way of probing the foldability landscape of a particular structure. The points on this landscape are given by all possible mutant sequences, with the distance between any two being given by the number of mutations. Random permutations are a subset of this landscape. If even a full random permutation allows complete folding to the native structure without getting stuck in a partially folded state and preserves folding cooperativity, then sequences with diverse packing signatures can be accommodated on that structure. Such structures are likely to be more designable (Helling et al., 2001; England and Shakhnovich, 2003) and good topologies for protein redesign.

## Conclusion

The RP method, reported here, can be used to assess the response of protein structures to packing perturbations. To apply this method, random permutations of the protein sequence are fit back onto the protein backbone. This procedure preserves the protein backbone structure but scrambles the position of large and small side-chains, perturbing packing. Folding simulations of these RP structures are then performed using coarse-grained structure based models and the perturbed packing is assessed using folding derived parameters such as barriers to folding, folding routes and the population of intermediates along these routes. We apply the method to six previously experimentally characterized *de novo* designed proteins and find that random permutation does not significantly affect barriers to folding, positions of the unfolded and folded basins or folding routes in three of the designed proteins. Thus, the method predicts that these proteins, namely, the Rossmann 2X2, the P-loop 2X2, and the Ferredoxin-like protein, should be able to accommodate mutations in all parts of their structures. Random permutation allows the discovery of an alternative folding route in the IF3 protein. So, the method can be used to identify patterns of packing which promote a specific folding route in a given scaffold. Finally, our folding simulations show that Top7 and the Rossmann 3X1 proteins populate folding intermediates. However, random permutation can be used to isolate specific contacts and in turn suggest mutations which can destabilize the folding intermediates and make folding more cooperative in both proteins. In summary, the RP method can be used to choose protein scaffolds for whole protein design as well as to identify protein regions which are sensitive to perturbations where further mutations should be avoided during protein engineering.

## Data Availability

Data used to support the findings of this study are available from the corresponding authors upon reasonable request.
