# Supplementary material for: A Method for Assessing the Robustness of Protein Structures by Randomizing Packing Interactions
Source: Front Mol Biosci. 2022 Jun 27;9:849272. doi: 10.3389/fmolb.2022.849272 (PMC9271847; doi:10.3389/fmolb.2022.849272)
Supplement: Supplementary file 1 [file DataSheet1.DOCX]

Supplementary Material

A method for assessing the robustness of protein structures by randomizing packing interactions

Shilpa Yadahalli^1,*,†^, Lakshmi P. Jayanthi^1^, Shachi Gosavi^1,*^

^1^Simons Centre for the Study of Living Machines, National Centre for Biological Sciences, Tata Institute of Fundamental Research, Bangalore 560065, India

**^†^**Present address:

ProteinQure,119 Spadina Avenue, Toronto, ON M5V 2L1

*** Correspondence:**

Shachi Gosavi

shachi@ncbs.res.in

or

Shilpa Yadahalli

shilpa.yadahalli@gmail.com

Keywords: Packing perturbation, Protein scaffold, Structure-based models, Molecular dynamics simulations, Sequence permutations, Robustness of protein structure, Protein folding.

**Supplementary Table 1.** Folding temperature and contact data for the WT and RPs of the natural proteins.

| **Protein Name** | **Number of contacts** | **T_f_** | **T_f_/120** | **Number of residues** |
| --- | --- | --- | --- | --- |
| **S6 WT** | 211 | 125 | 1.04 | 97 |
| RP1 | 193 | 117 | 0.98 |  |
| RP2 | 190 | 119 | 0.99 |  |
| RP3 | 199 | 121 | 1.01 |  |
| RP4 | 187 | 117 | 0.98 |  |
| RP5 | 204 | 122 | 1.02 |  |
| **ecoRNase-H WT** | 352 | 124 | 1.03 | 155 |
| RP1 | 317 | 120 | 1.00 |  |
| RP2 | 327 | 123 | 1.03 |  |
| RP3 | 326 | 122 | 1.02 |  |
| RP4 | 327 | 123 | 1.03 |  |
| RP5 | 328 | 122 | 1.02 |  |

**Supplementary Table 2.** Folding temperature and contact data for the WT and RPs of the designed proteins.

| **Protein Name** | **Number of contacts** | **T_f_** | **T_f_/120** | **Number of residues** |
| --- | --- | --- | --- | --- |
| Rossmann 2X2 WT | 216 | 122 | 1.02 | 99 |
| RP1 | 220 | 123 | 1.03 |  |
| RP2 | 233 | 127 | 1.06 |  |
| RP3 | 226 | 126 | 1.05 |  |
| RP4 | 224 | 125 | 1.04 |  |
| RP5 | 216 | 122 | 1.02 |  |
| P-loop 2X2 WT | 209 | 116 | 0.97 | 101 |
| RP1 | 197 | 113 | 0.94 |  |
| RP2 | 199 | 114 | 0.95 |  |
| RP3 | 182 | 107 | 0.89 |  |
| RP4 | 205 | 117 | 0.98 |  |
| RP5 | 208 | 115 | 0.96 |  |
| Ferredoxin WT | 159 | 122 | 1.02 | 76 |
| RP1 | 155 | 119 | 0.99 |  |
| RP2 | 150 | 116 | 0.97 |  |
| RP3 | 160 | 121 | 1.01 |  |
| RP4 | 166 | 123 | 1.03 |  |
| RP5 | 171 | 127 | 1.06 |  |
| IF3 WT | 145 | 119 | 0.99 | 72 |
| RP1 | 143 | 115 | 0.96 |  |
| RP2 | 144 | 117 | 1.2 |  |
| RP3 | 136 | 113 | 1.13 |  |
| RP4 | 147 | 119 | 0.99 |  |
| RP5 | 140 | 114 | 1.17 |  |
| Rossmann 3X1 WT | 205 | 118 | 0.98 | 99 |
| RP1 | 201 | 117 | 0.98 |  |
| RP2 | 204 | 118 | 0.98 |  |
| RP3 | 200 | 116 | 0.97 |  |
| RP4 | 194 | 114 | 0.95 |  |
| RP5 | 205 | 119 | 0.99 |  |
| Top7 WT | 202 | 122 | 1.02 | 92 |
| RP1 | 183 | 118 | 0.98 |  |
| RP2 | 169 | 111 | 0.93 |  |
| RP3 | 179 | 115 | 0.96 |  |
| RP4 | 187 | 118 | 0.98 |  |
| RP5 | 189 | 117 | 0.98 |  |


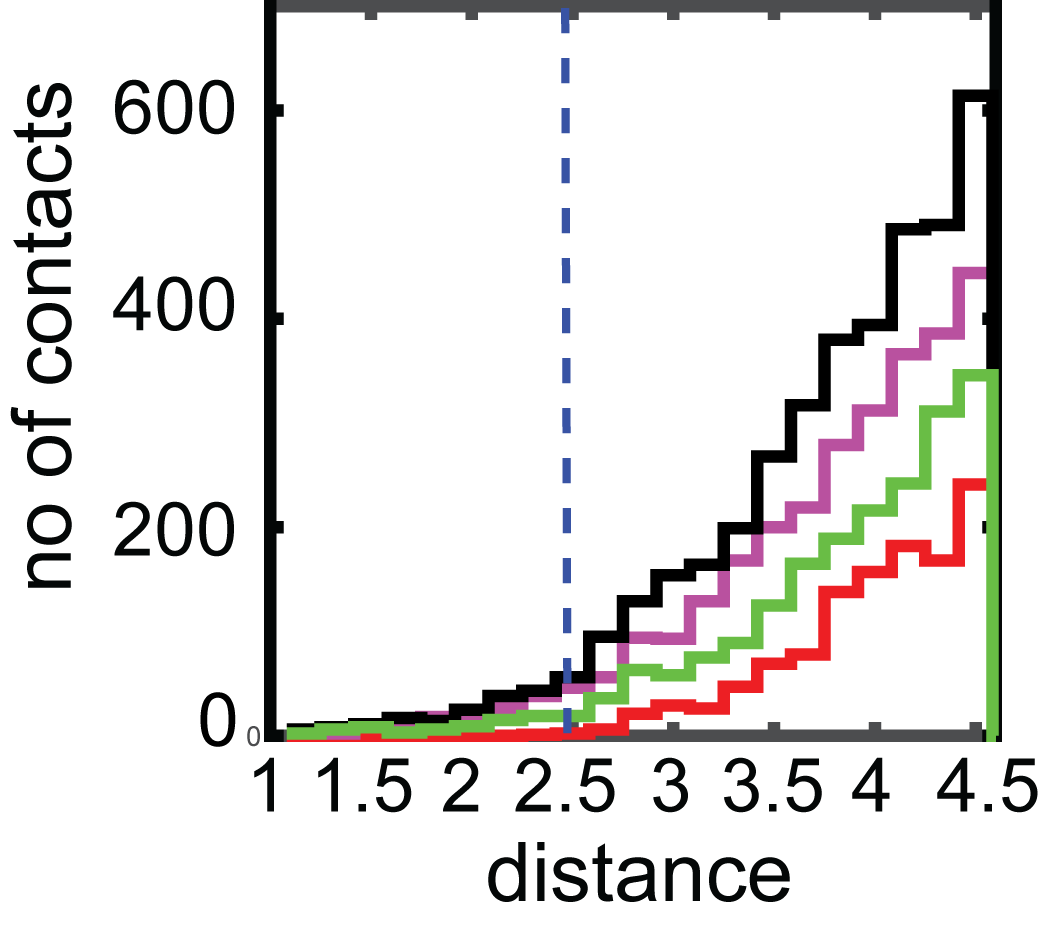


**Supplementary Figure 1**. Histogram of the number of atomic contacts as a function of the distance (in Å) between the two atoms that are in contact for WT (red), poly-tyrosine (magenta), poly-arginine (green) and poly-tryptophan (black) sequences packed onto the structure of Top7. The contacts were calculated using a 4.5 Å cutoff contact map. The amino acids that are used are large and in the case of arginine, charged. Despite the size and the charge of the amino acid, SCWRL4 is able to pack the sequences onto the structure. However, this packing generates many more short contacts than the WT sequence. The dashed blue line indicates the distance at which the short contacts of the WT start to form. Histograms for all the 8 simulated proteins are similar to the histograms shown here.


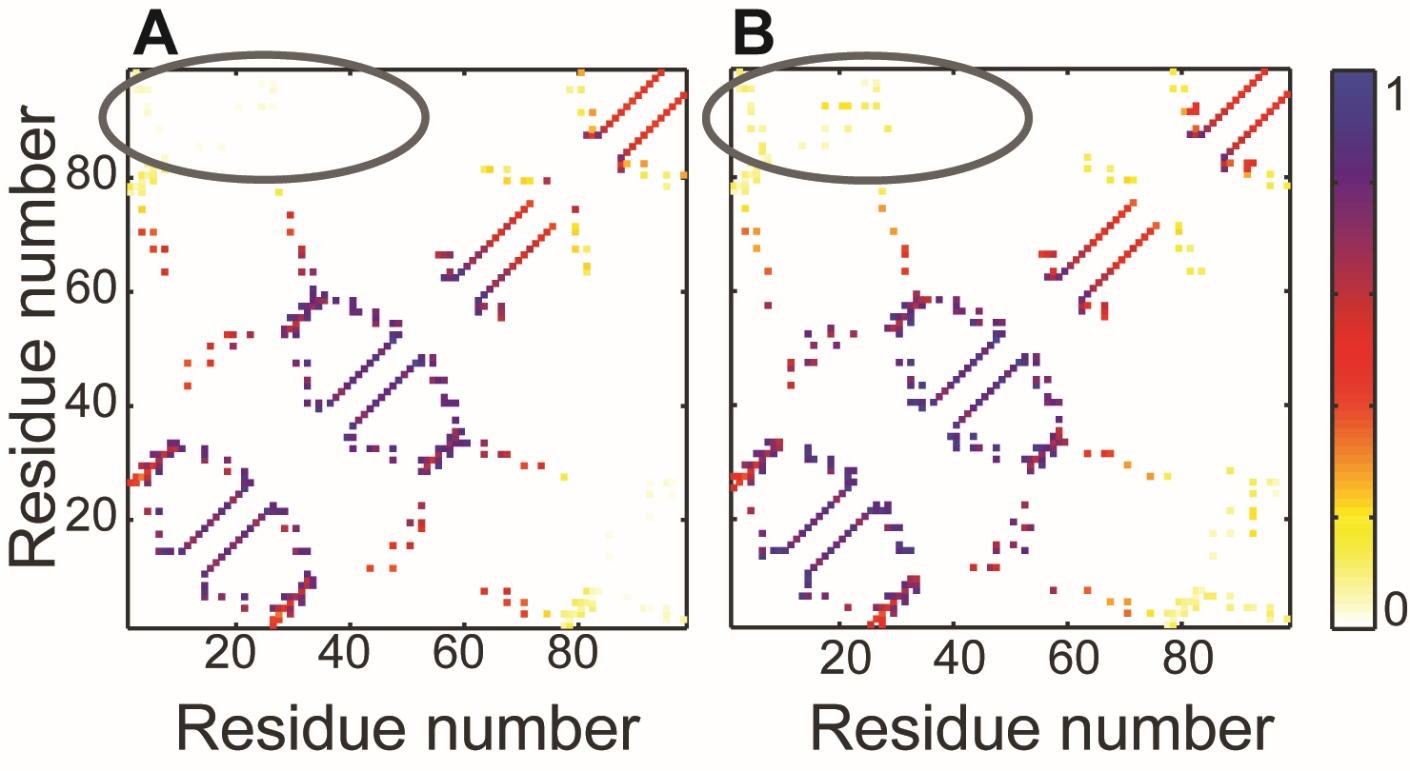


**Supplementary Figure 2**. Average contact maps of proteins from the Rossmann 3X1 fold at about 55% foldedness or Q~0.55. (A) WT and (B) the RP with the highest barrier. The colors depict the probability of contact formation and the color scale is given on the right. A darker (e.g. blue) color implies a more formed contact while a lighter (e.g. yellow) color implies a less formed contact. The contacts between the C-terminal helix and the rest of the protein (enclosed in the ellipse) are more formed in the RP where a large barrier to folding is present. The WT populates an intermediate in which everything except the C-terminal helix is mostly folded.

**Supplementary Data 1a. Sequences of the WT (bold) and RPs**

**S6:**

**MRRYEVNIVLNPNLDQSQLALEKEIIQRALENYGARVEKVEELGLRRLAYPIAKDPQGYFLWYQVEMPEDRVNDLARELRIRDNVRRVMVVKSQEPF**

MPFRVRYRERQERLARPVNVILAYPNWVNPNVFLMVLRIREDQYRDYRLNEDLLKVAKEILIDIVLQVEQMQARVRDPQAESSGLEEAGLKKEGENY

EYNELGVIRLPRQKRAEKIMLDQVAANRRRRNVPNLLWFVDPVLAKINVGVSEEQEEQLQERLDPYMRQYAAVYNRDYLIRKRSMIVDEVGFEPLLE

GAEKVVEDERDYYRLPQIELRAARVNNQLDLREPSNLVVYEKELYVMKLNEELLIYAPFGVSKERDDMVQMWQEGRQNPIIIARNRARRLVVQRLFP

REQALRLLVYDEEDNPRMQSNMRIDLGADEMVERLRRVYNAKQVYLNRLEGIYNELPKVWLLGPPIVRAQSVVKVQRIPLFAEIVDYAQENKEFRER

REQYQDDRFKRELAEYLRFAWVLRRNDAEKAQLPAPLIYENNVVPIERMQEVEERAGMRVIVVYSKVSGLQEDDYLINLLLPRNRRNEPIVVKQGML

**ecoRNase-H:**

**MLKQVEIFTDGSCLGNPGPGGYGAILRYRGREKTFSAGYTRTTNNRMELMAAIVALEALKEHCEVILSTDSQYVRQGITQWIHNWKKRGWKTADKKPVKNVDLWQRLDAALGQHQIKWEWVKGHAGHPENERCDELARAAAMNPTLEDTGYQVEV**

QHMAAQKLPNVAMTTEMIPVIGNAPFVETSLYPLRRRAGVITRKNHDQSKWYLHKVIKCFEAGKGKLDTTQGGLDAAGVTEIEAGRLVCGWNLARAIDYTRYWRRDGRKLLEKWQEEMADQQTEPCGEHALWSWYQLHIGVTNNGAVKNKGSEDE

AAMGLVREVQSNCARFLETEAGAQHVMTIMNRNDNDWECLHWDDIGTKNPGTATGGPKIKLEEPNHHQVKQLQQRRAGKNSGWIAEQIAIKPKKTYPYKRVRDHRRKTALRQWGEGDEKSFDAVVWIGYCLLEVLETMGYWLAAYTVLATGLESG

IVQGRADLENSRCLKPAYVECGEAELDKVGYGMTGPVQRDLITAWEVRWWENLTQRKVMEDVAGWTTLKDAWNAETMSCIHTHVGAQYKMRKTHGAPSQHNAVNNGPAYTPLQLERWGSAEAETGALLRIFGEYIGKDKKLQLKGRRNQDFIIHK

KPVVLWLTCVGRKAGQPRGKCMGALLADALKHTALGLEGDKLEGETVGHHGDMKERIEEQEGYSWLKFTAARPAAWDAQRINDLQESEMQLPDNNTRDYNVHALTREGWQNTMKAGRCWNQTYTRYGPAHNEISVKFTSQEAGIIVKIRVWVKIY

TTDWLTLYGINVEDRKLKVGEVKLSGHTPYVRRILQKMLEKDGIKKAEELAWAGKNVWTRFISGKMARIENAQEEGACVGQRYWYDMFCDKSRQLNAIAEVPHHQLNMRDGHPGLAAGPHTEATEDLWALVRGVPIQGQTRTWNKCTYQNEGSAA

**Rossmann 2X2**

**LLYVLIISNDKKLIEEARKMAEKANLELRTVKTEDELKKYLEEFRKESQNIKVLILVSNDEELDKAKELAQKMEIDVRTRKVTSPDEAKRWIKEFSEEG**

YTLAEWEAKLRKVTEKRKAEEELKENYLEVDLDVDKIGMPEELVNALILKKEKISENSDRIAEITELFQSVSLIEKRIEKKLMAETQKFVDLDSKKRNR

KSLLKNKVKEQREFEVREEKLTADAIVDSSNIRYNTKRIKKKEMVEKENMEEIIWKDAVELIDKLLDPEIAEEQLKLGKVSDFASLLRERLTAEKYETL

EKKEPNKITKSKQSIVERKVLVRIMSVKIAMAESKKETLWRRGTALKNFILLLREKFELKQEIVLLDILEEYEDRKNKELEDDYEVDSDNAETAALEEK

DAEKFNEKDAKSESYVLELEATQVEEEDSAPLRFIMKELRDIKLEKGWKTNQRAIRNIEVKISLKLIVDKTRNLYVKKLLISMEVTLEALDKREKKEEE

FRLLKRRDELLLKTSEAIKTGEQKIAYIVDKSKSEEREDPSAKDKYERLVNDEIALNMLAEKLIDQVKTTEKVEEESEIALKWMNLKRNVIKLEEEVKF

**Ploop 2x2:**

**GKVLLVISTDTNIISSVQERAKHNYPGREIRTATSSQDIRDIIKSMKDNGKPLVVFVNGASQNDVNEFQNEAKKEGVSYDVLKSTDPEELTQRVREFLKTA**

PEVNMLRAVDQELESIDAKELKQNRKDEVATAQSNFSFVKILRLITGIVSDRKADVTNRERTSKKGNVQFVDTEEVGTIHSSSNYDKLKPVINYSQITPGG

RSERSNIADKLAVIALQSKYVDQKDSLTSENKTSAKTRDGVIKFIFLITKGEPEKRPVIVFDTGNNKREDQNVYNITLEEQSTSHEDMVVPSVANQRLVGG

EINDTNEQAVVNVRLASKILLFDINREGDESSETTKRSVDLDVVIAELMSYDNGSVIIPTKSIRSVTGLVEGTSFQATDKVPQKYKKRNRKNHGPKEAQFQ

SESKQAGATVDNVLTPRVNRITNSEIEASILNKGGLQKTRKRIALVVVSKPNMISKVHIVDTDSVLPDVIFNQGEKERKETTNRLAFEDKFSQEDYGSQDY

VDREVFQSTQEFKIANLGLKKATRPEVSARVPTDDLIRDMYIIATQESNKVAGVEYNQKEFLKSVRGLGIDSDKSDISRNKVTLVTVSNNPNITESEHQGK

**Ferredoxin-like:**

**EMDIRFRGDDLEAFEKALKEMIRQARKFAGTVTYTLDGNDLEIRITGVPEQVRKELAKEAERLAKEFNITVTYTIR**

ILRRRDTALIALPGRTIEEEIRFKTRQYVKEEDITEFEKKRDKARLNGTDEAQFVMGFTLMINEKAVGDEALAVYT

ENKAYIIFRENTEVGYTEDERDRLITTTDAAEKVFRRGDQKLEVLVFIIDKLAMFTGEAAQTRGRMEIERALKPKL

DTRFEDDKAVAILRNLAMMLEGYQVIDREKPINIVIKKTRARTLRFIERGTDAFEGTEELQEETKKTREGFLVAYA

LEDPMTIIDIKLVLRTFMGEGTAEIRNIAAGTFKREFVDYQADVEEKVQEARIDTRLRERRTNTKGELFAEAKYKL

RLTATKDLIAFTGKAQYREVYLAENQNKITVRKRILTVILMDEPKIGGRDVRLAEKREGFDETREAMEFFETDIAE

**IF3-like:**

**LTRTITSQNKEELLEIALKFISQGLDLEVEFDSTDDKEIEEFERDMEDLAKKTGVQIQKQWQGNKLRIRLKG**

ITENIKRDIGKSFQLRIETKALEQEETSEQLVEMGNDKILLQIKKDGFLSDRFVGEDAKQKQLETLEWTDLR

EEKNIESEAKLSLIFITKNQQKGIVWRGVEELETQDGDLDIGLKQLMKLRDLKEQRLASTEERDIDKQFFTT

TKSMRLEDNEIRTDFLELQLADRLFDLKTLIQVLWKEGEKKREIDDQKQEGSNKESQIIETLGQEIVKATGF

WDITDTGRKTTKLQLDINLQIFKAIEEGKDLQIEEEESEQFEKLRLRASSGVTLQNKDVQFKDGLEMEILKR

AISLKKGWSVRTKEQTLTGVLTNLEEMTIKEDEFGLRNFEEDKEDAELDDIRLLIDSQIKQLQIKEQQKRFG

**Rossmann 3X1**

**SKIIVIISSDDTTLEELARKIKDEGLEVYILLKDKDEKRLEEKIQKLKSQGFEVRKVKDDDDIDKWIDKIKKERPQLEVRKVTDEDQAKQILEDLKKKG**

WARKLDDGDRIDSIGIESREQKEKKVEPDLDAETGQVEDDKKELKYFVERKDIQKLDKKVIVIVKSDKKLLLDEDQIKIKSIEKKLIETRDQILTEKKL

QISLKTEVIIDEGWVKLRVIEDEEDILKVSKEDRLQDATDEDDGEVELFYIKDKKVQRLQKIEIKKDDKLEAIKGKLQDKEDSPKRKLKKKDSILKTIR

VPEDLKKTGIRKDIVREKKKLSEEGIIDSLKKSIDDKEFGRQLDLKAKEEDKVVREQDYKIAITILDWLKLDSDDEIQKKIEERVKVLKKKQLQEDDTI

DTLKDADKRKKQDWKDRIDKIEEKRVLKEDLSIIRKKVKKDDESLQKVKEDPVLEEFDIEESGKIKGIIEVIQDTRGKDLDYLALQKKKVESLQIEILT

ILVDEKDEKDKLTKRQDKTGGALRIKAEQKKDVKILEEDKEIVQQDQKDDDESIPGEKLIFKLIIRYVSVKEIEDELWDLSRDRKKKVSDKIELKLIKT

**Top7:**

**DIQVQVNIDDNGKNFDYTYTVTTESELQKVLNELMDYIKKQGAKRVRISITARTKKEAEKFAAILIKVFAELGYNDINVTFDGDTVTVEGQL**

NAIVTFSNEMKDIFTTTNIEGQQDTQYDTIDFYDVVLEAYKIIVVLDNLQVNRLKLNKEEAKVKTRSGLKKVTTGDGAVAREKYIFAGIDQE

DKYTNIEDTTALVQTINLFVVLTIGNRIYDQEIKEARKVQEFIDVRTVIKIVDKQDLTAEGVFAQLYKNMGGFKATDETAEKLNDNKSYSVG

IDAFIIENYKVAIMASKLNDAQNGQYEFKVTNVKEREIEKTRLVGQRDKDGDNDTTLIYTGIVFFQGADTANSEDQTKILYEVTVKVKLVTL

QNAKVKYLVENSQGEQAGTRIRRIQTLKINGFDFKIDDVELADTTETVTKDIEATGKGEQYFNALSYAVVNIVDVNDTEMKIYLLTVDIKFK

VITAKAEGDLNVAEANVGDIGITIEQTNKKEQKQNTVDLVDRMLTLGTIIYLKKITAVVEQKATRRVDEDKQYSNNFDEYFLDIKFVTGFYS

**Supplementary Data 1b.** Sequences of the WT and the 5 RPs. Amino acids are colored as follows: aliphatic (GAVLMI):grey-green; aromatic (FYW): yellow; positively charged (KRH): blue, negatively charged (DE): red and polar (STCPNQ): cyan. The order of secondary structural elements is shown above the WT sequence of each protein with rectangles depicting α-helices and arrows depicting β-sheets

**S6 (PDB: 1RIS)**


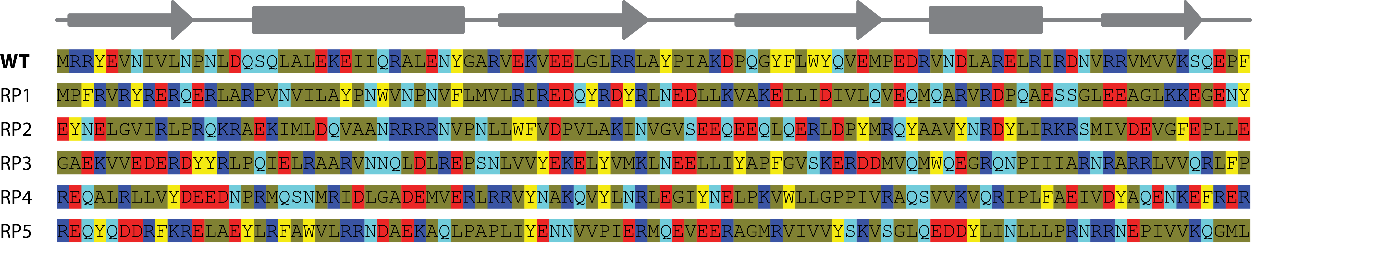


**ecoRNase-H (PDB: 2RN2)**


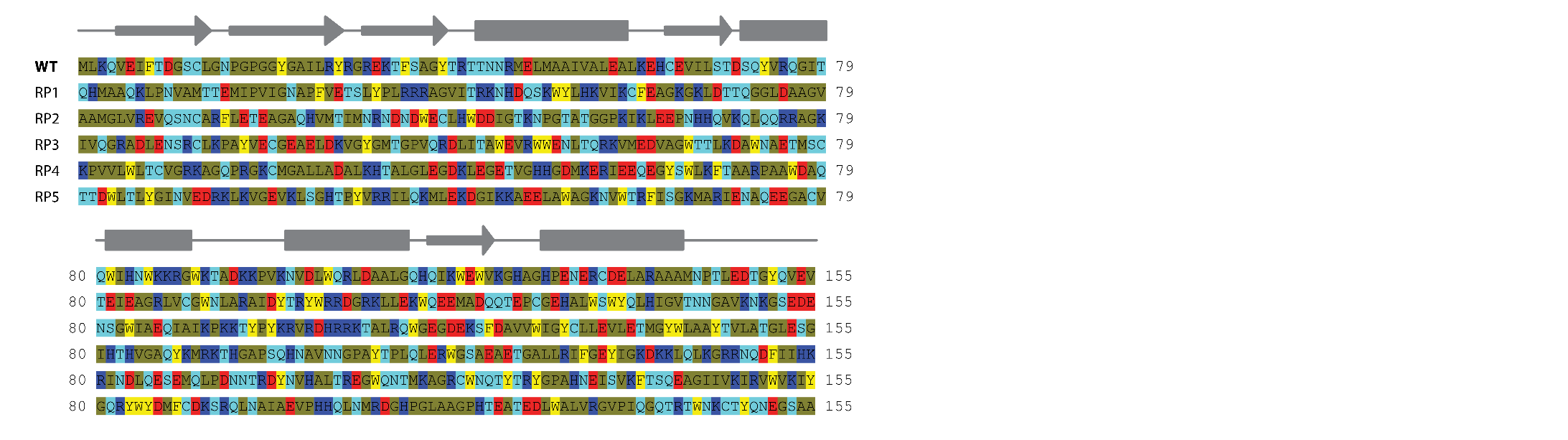


**Rossmann 2X2 (PDB ID: 2LV8)**


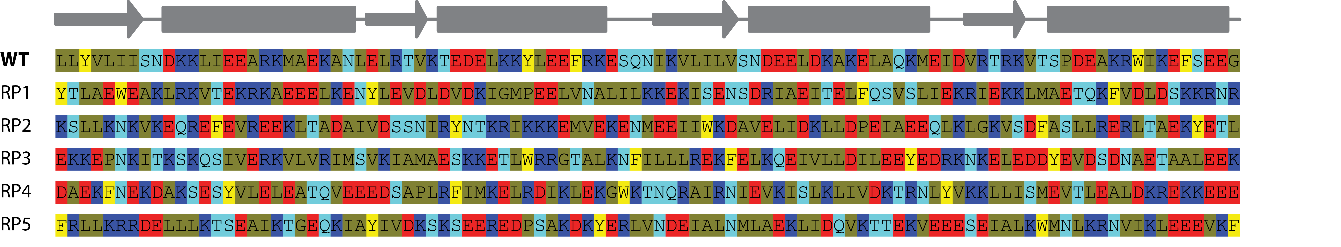


**Ploop 2x2 (PDB Id: 2LVB)**


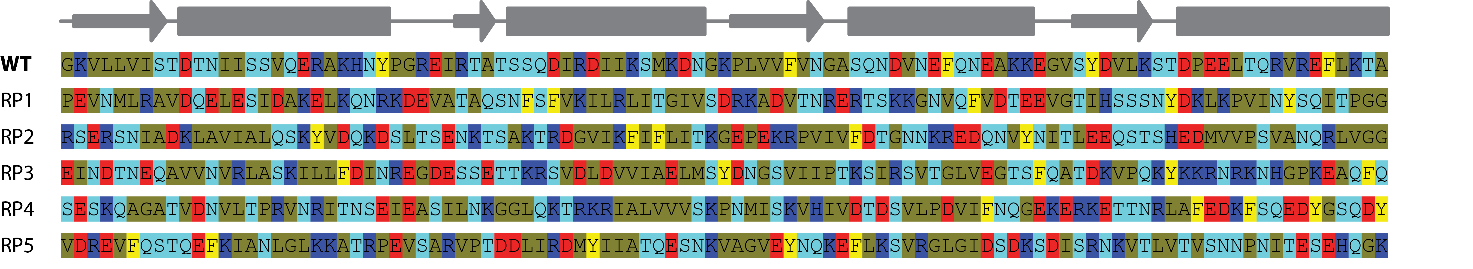


**Ferredoxin-like fold (PDB: 2KL8)**


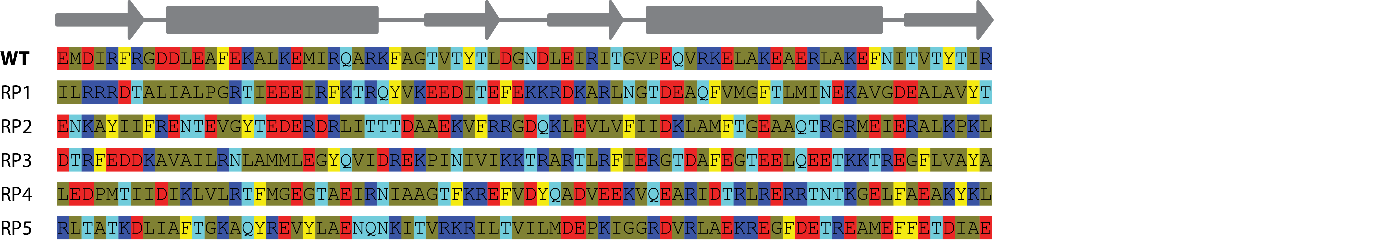


**IF3-like (PDB: 2LN3)**


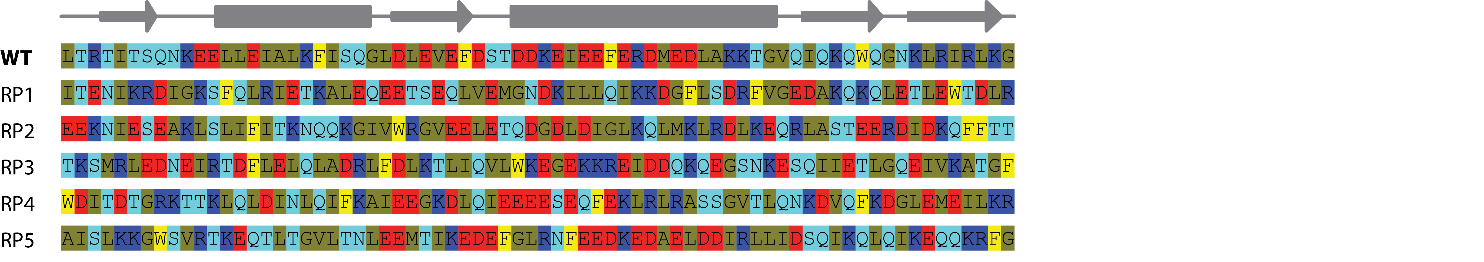


**Rossmann 3X1 (PDB: 2LTA)**


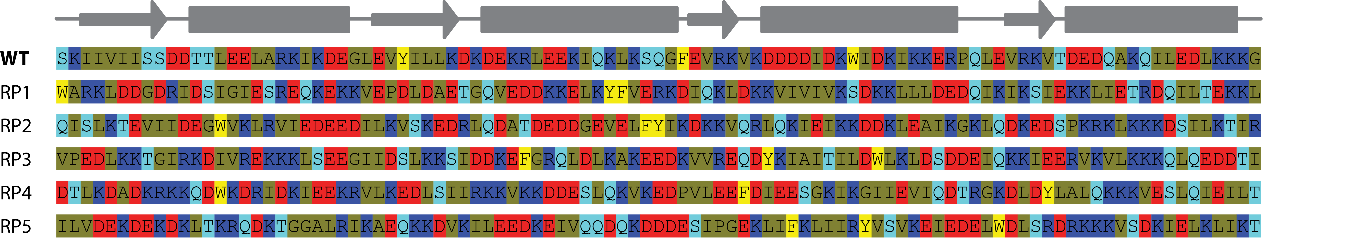


**Top7 (PDB: 1QYS)**


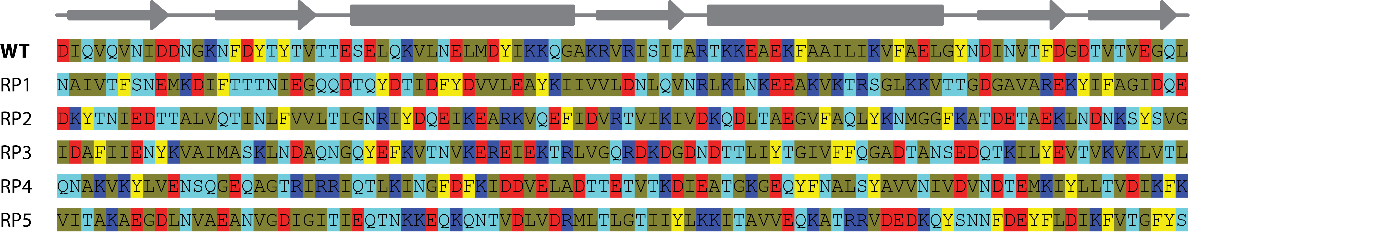


**Supplementary Data 2. Model structures of five computationally designed proteins with hydrogen atoms removed.**

**PDB ID 2KL8**

ATOM 1 N GLU 1 8.643 0.242 -3.765 1.00 0.00

ATOM 2 CA GLU 1 9.931 0.835 -3.428 1.00 0.00

ATOM 3 C GLU 1 10.118 2.179 -4.120 1.00 0.00

ATOM 4 O GLU 1 10.369 2.238 -5.324 1.00 0.00

ATOM 5 CB GLU 1 11.072 -0.113 -3.804 1.00 0.00

ATOM 6 CG GLU 1 12.457 0.387 -3.420 1.00 0.00

ATOM 7 CD GLU 1 13.522 -0.597 -3.815 1.00 0.00

ATOM 8 OE1 GLU 1 13.190 -1.602 -4.396 1.00 0.00

ATOM 9 OE2 GLU 1 14.679 -0.294 -3.637 1.00 0.00

ATOM 10 N MET 2 9.995 3.257 -3.354 1.00 0.00

ATOM 11 CA MET 2 10.237 4.598 -3.872 1.00 0.00

ATOM 12 C MET 2 11.695 5.001 -3.697 1.00 0.00

ATOM 13 O MET 2 12.111 5.408 -2.612 1.00 0.00

ATOM 14 CB MET 2 9.323 5.604 -3.175 1.00 0.00

ATOM 15 CG MET 2 9.443 7.029 -3.697 1.00 0.00

ATOM 16 SD MET 2 8.677 7.244 -5.316 1.00 0.00

ATOM 17 CE MET 2 10.106 7.119 -6.386 1.00 0.00

ATOM 18 N ASP 3 12.467 4.885 -4.772 1.00 0.00

ATOM 19 CA ASP 3 13.865 5.297 -4.759 1.00 0.00

ATOM 20 C ASP 3 14.044 6.674 -5.383 1.00 0.00

ATOM 21 O ASP 3 13.977 6.826 -6.602 1.00 0.00

ATOM 22 CB ASP 3 14.733 4.272 -5.495 1.00 0.00

ATOM 23 CG ASP 3 16.220 4.599 -5.504 1.00 0.00

ATOM 24 OD1 ASP 3 16.580 5.650 -5.032 1.00 0.00

ATOM 25 OD2 ASP 3 16.995 3.734 -5.838 1.00 0.00

ATOM 26 N ILE 4 14.266 7.676 -4.539 1.00 0.00

ATOM 27 CA ILE 4 14.512 9.034 -5.010 1.00 0.00

ATOM 28 C ILE 4 15.969 9.430 -4.812 1.00 0.00

ATOM 29 O ILE 4 16.474 9.432 -3.688 1.00 0.00

ATOM 30 CB ILE 4 13.610 10.053 -4.291 1.00 0.00

ATOM 31 CG1 ILE 4 12.137 9.661 -4.440 1.00 0.00

ATOM 32 CG2 ILE 4 13.849 11.453 -4.836 1.00 0.00

ATOM 33 CD1 ILE 4 11.226 10.318 -3.428 1.00 0.00

ATOM 34 N ARG 5 16.640 9.767 -5.907 1.00 0.00

ATOM 35 CA ARG 5 18.030 10.201 -5.851 1.00 0.00

ATOM 36 C ARG 5 18.147 11.704 -6.071 1.00 0.00

ATOM 37 O ARG 5 17.716 12.227 -7.099 1.00 0.00

ATOM 38 CB ARG 5 18.913 9.428 -6.819 1.00 0.00

ATOM 39 CG ARG 5 19.075 7.951 -6.496 1.00 0.00

ATOM 40 CD ARG 5 19.873 7.190 -7.491 1.00 0.00

ATOM 41 NE ARG 5 20.067 5.787 -7.162 1.00 0.00

ATOM 42 CZ ARG 5 20.783 4.919 -7.903 1.00 0.00

ATOM 43 NH1 ARG 5 21.343 5.294 -9.033 1.00 0.00

ATOM 44 NH2 ARG 5 20.889 3.673 -7.477 1.00 0.00

ATOM 45 N PHE 6 18.733 12.396 -5.099 1.00 0.00

ATOM 46 CA PHE 6 18.960 13.830 -5.209 1.00 0.00

ATOM 47 C PHE 6 20.409 14.133 -5.574 1.00 0.00

ATOM 48 O PHE 6 21.326 13.826 -4.814 1.00 0.00

ATOM 49 CB PHE 6 18.589 14.534 -3.902 1.00 0.00

ATOM 50 CG PHE 6 17.120 14.505 -3.595 1.00 0.00

ATOM 51 CD1 PHE 6 16.578 13.490 -2.819 1.00 0.00

ATOM 52 CD2 PHE 6 16.274 15.493 -4.080 1.00 0.00

ATOM 53 CE1 PHE 6 15.227 13.462 -2.536 1.00 0.00

ATOM 54 CE2 PHE 6 14.924 15.467 -3.797 1.00 0.00

ATOM 55 CZ PHE 6 14.398 14.452 -3.025 1.00 0.00

ATOM 56 N ARG 7 20.605 14.735 -6.743 1.00 0.00

ATOM 57 CA ARG 7 21.943 15.065 -7.218 1.00 0.00

ATOM 58 C ARG 7 22.167 16.570 -7.231 1.00 0.00

ATOM 59 O ARG 7 21.249 17.343 -7.509 1.00 0.00

ATOM 60 CB ARG 7 22.239 14.446 -8.576 1.00 0.00

ATOM 61 CG ARG 7 22.311 12.928 -8.585 1.00 0.00

ATOM 62 CD ARG 7 22.657 12.339 -9.904 1.00 0.00

ATOM 63 NE ARG 7 22.744 10.887 -9.911 1.00 0.00

ATOM 64 CZ ARG 7 22.981 10.141 -11.008 1.00 0.00

ATOM 65 NH1 ARG 7 23.118 10.698 -12.190 1.00 0.00

ATOM 66 NH2 ARG 7 23.049 8.828 -10.867 1.00 0.00

ATOM 67 N GLY 8 23.393 16.983 -6.933 1.00 0.00

ATOM 68 CA GLY 8 23.730 18.400 -6.868 1.00 0.00

ATOM 69 C GLY 8 24.762 18.672 -5.780 1.00 0.00

ATOM 70 O GLY 8 24.833 17.953 -4.784 1.00 0.00

ATOM 71 N ASP 9 25.563 19.716 -5.978 1.00 0.00

ATOM 72 CA ASP 9 26.581 20.095 -5.006 1.00 0.00

ATOM 73 C ASP 9 25.997 20.985 -3.916 1.00 0.00

ATOM 74 O ASP 9 26.303 22.176 -3.846 1.00 0.00

ATOM 75 CB ASP 9 27.744 20.807 -5.700 1.00 0.00

ATOM 76 CG ASP 9 28.949 21.063 -4.803 1.00 0.00

ATOM 77 OD1 ASP 9 28.951 20.586 -3.693 1.00 0.00

ATOM 78 OD2 ASP 9 29.919 21.597 -5.284 1.00 0.00

ATOM 79 N ASP 10 25.157 20.402 -3.069 1.00 0.00

ATOM 80 CA ASP 10 24.572 21.128 -1.947 1.00 0.00

ATOM 81 C ASP 10 24.127 20.175 -0.846 1.00 0.00

ATOM 82 O ASP 10 23.017 19.644 -0.883 1.00 0.00

ATOM 83 CB ASP 10 23.389 21.977 -2.418 1.00 0.00

ATOM 84 CG ASP 10 22.791 22.873 -1.341 1.00 0.00

ATOM 85 OD1 ASP 10 23.177 22.741 -0.204 1.00 0.00

ATOM 86 OD2 ASP 10 22.076 23.785 -1.684 1.00 0.00

ATOM 87 N LEU 11 24.999 19.963 0.132 1.00 0.00

ATOM 88 CA LEU 11 24.707 19.055 1.236 1.00 0.00

ATOM 89 C LEU 11 23.549 19.571 2.080 1.00 0.00

ATOM 90 O LEU 11 22.748 18.792 2.599 1.00 0.00

ATOM 91 CB LEU 11 25.954 18.860 2.106 1.00 0.00

ATOM 92 CG LEU 11 27.098 18.081 1.442 1.00 0.00

ATOM 93 CD1 LEU 11 28.331 18.102 2.334 1.00 0.00

ATOM 94 CD2 LEU 11 26.651 16.651 1.172 1.00 0.00

ATOM 95 N GLU 12 23.463 20.892 2.215 1.00 0.00

ATOM 96 CA GLU 12 22.387 21.514 2.975 1.00 0.00

ATOM 97 C GLU 12 21.024 21.185 2.376 1.00 0.00

ATOM 98 O GLU 12 20.097 20.807 3.092 1.00 0.00

ATOM 99 CB GLU 12 22.581 23.032 3.033 1.00 0.00

ATOM 100 CG GLU 12 21.531 23.768 3.853 1.00 0.00

ATOM 101 CD GLU 12 21.811 25.244 3.899 1.00 0.00

ATOM 102 OE1 GLU 12 22.789 25.663 3.327 1.00 0.00

ATOM 103 OE2 GLU 12 20.990 25.969 4.410 1.00 0.00

ATOM 104 N ALA 13 20.914 21.329 1.061 1.00 0.00

ATOM 105 CA ALA 13 19.703 20.934 0.349 1.00 0.00

ATOM 106 C ALA 13 19.396 19.456 0.560 1.00 0.00

ATOM 107 O ALA 13 18.245 19.076 0.770 1.00 0.00

ATOM 108 CB ALA 13 19.840 21.243 -1.135 1.00 0.00

ATOM 109 N PHE 14 20.433 18.628 0.503 1.00 0.00

ATOM 110 CA PHE 14 20.277 17.191 0.696 1.00 0.00

ATOM 111 C PHE 14 19.665 16.881 2.055 1.00 0.00

ATOM 112 O PHE 14 18.762 16.052 2.165 1.00 0.00

ATOM 113 CB PHE 14 21.625 16.483 0.552 1.00 0.00

ATOM 114 CG PHE 14 22.092 16.352 -0.870 1.00 0.00

ATOM 115 CD1 PHE 14 21.373 16.926 -1.907 1.00 0.00

ATOM 116 CD2 PHE 14 23.252 15.654 -1.173 1.00 0.00

ATOM 117 CE1 PHE 14 21.803 16.806 -3.215 1.00 0.00

ATOM 118 CE2 PHE 14 23.684 15.534 -2.480 1.00 0.00

ATOM 119 CZ PHE 14 22.958 16.110 -3.502 1.00 0.00

ATOM 120 N GLU 15 20.160 17.553 3.089 1.00 0.00

ATOM 121 CA GLU 15 19.651 17.360 4.442 1.00 0.00

ATOM 122 C GLU 15 18.186 17.759 4.543 1.00 0.00

ATOM 123 O GLU 15 17.384 17.059 5.162 1.00 0.00

ATOM 124 CB GLU 15 20.484 18.160 5.447 1.00 0.00

ATOM 125 CG GLU 15 21.890 17.622 5.668 1.00 0.00

ATOM 126 CD GLU 15 22.687 18.528 6.565 1.00 0.00

ATOM 127 OE1 GLU 15 22.183 19.562 6.932 1.00 0.00

ATOM 128 OE2 GLU 15 23.754 18.136 6.976 1.00 0.00

ATOM 129 N LYS 16 17.840 18.888 3.931 1.00 0.00

ATOM 130 CA LYS 16 16.466 19.371 3.936 1.00 0.00

ATOM 131 C LYS 16 15.533 18.385 3.245 1.00 0.00

ATOM 132 O LYS 16 14.442 18.098 3.740 1.00 0.00

ATOM 133 CB LYS 16 16.379 20.741 3.259 1.00 0.00

ATOM 134 CG LYS 16 17.019 21.875 4.049 1.00 0.00

ATOM 135 CD LYS 16 16.892 23.202 3.316 1.00 0.00

ATOM 136 CE LYS 16 17.551 24.330 4.092 1.00 0.00

ATOM 137 NZ LYS 16 17.467 25.627 3.369 1.00 0.00

ATOM 138 N ALA 17 15.967 17.870 2.102 1.00 0.00

ATOM 139 CA ALA 17 15.180 16.896 1.352 1.00 0.00

ATOM 140 C ALA 17 15.011 15.602 2.139 1.00 0.00

ATOM 141 O ALA 17 13.942 14.991 2.126 1.00 0.00

ATOM 142 CB ALA 17 15.826 16.620 0.003 1.00 0.00

ATOM 143 N LEU 18 16.075 15.187 2.820 1.00 0.00

ATOM 144 CA LEU 18 16.036 13.982 3.639 1.00 0.00

ATOM 145 C LEU 18 15.045 14.124 4.786 1.00 0.00

ATOM 146 O LEU 18 14.265 13.211 5.061 1.00 0.00

ATOM 147 CB LEU 18 17.435 13.664 4.181 1.00 0.00

ATOM 148 CG LEU 18 17.523 12.414 5.067 1.00 0.00

ATOM 149 CD1 LEU 18 17.113 11.182 4.273 1.00 0.00

ATOM 150 CD2 LEU 18 18.941 12.266 5.597 1.00 0.00

ATOM 151 N LYS 19 15.076 15.272 5.451 1.00 0.00

ATOM 152 CA LYS 19 14.162 15.548 6.551 1.00 0.00

ATOM 153 C LYS 19 12.711 15.461 6.094 1.00 0.00

ATOM 154 O LYS 19 11.863 14.905 6.791 1.00 0.00

ATOM 155 CB LYS 19 14.445 16.928 7.148 1.00 0.00

ATOM 156 CG LYS 19 15.753 17.022 7.923 1.00 0.00

ATOM 157 CD LYS 19 15.961 18.418 8.489 1.00 0.00

ATOM 158 CE LYS 19 17.278 18.519 9.245 1.00 0.00

ATOM 159 NZ LYS 19 17.512 19.890 9.774 1.00 0.00

ATOM 160 N GLU 20 12.431 16.015 4.919 1.00 0.00

ATOM 161 CA GLU 20 11.092 15.966 4.348 1.00 0.00

ATOM 162 C GLU 20 10.682 14.534 4.030 1.00 0.00

ATOM 163 O GLU 20 9.561 14.119 4.324 1.00 0.00

ATOM 164 CB GLU 20 11.015 16.828 3.086 1.00 0.00

ATOM 165 CG GLU 20 9.616 16.966 2.502 1.00 0.00

ATOM 166 CD GLU 20 8.686 17.646 3.468 1.00 0.00

ATOM 167 OE1 GLU 20 9.059 18.659 4.009 1.00 0.00

ATOM 168 OE2 GLU 20 7.562 17.217 3.581 1.00 0.00

ATOM 169 N MET 21 11.597 13.782 3.427 1.00 0.00

ATOM 170 CA MET 21 11.334 12.392 3.071 1.00 0.00

ATOM 171 C MET 21 11.057 11.550 4.307 1.00 0.00

ATOM 172 O MET 21 10.186 10.680 4.296 1.00 0.00

ATOM 173 CB MET 21 12.513 11.814 2.291 1.00 0.00

ATOM 174 CG MET 21 12.602 12.282 0.846 1.00 0.00

ATOM 175 SD MET 21 11.160 11.807 -0.129 1.00 0.00

ATOM 176 CE MET 21 10.200 13.318 -0.071 1.00 0.00

ATOM 177 N ILE 22 11.802 11.814 5.377 1.00 0.00

ATOM 178 CA ILE 22 11.615 11.099 6.635 1.00 0.00

ATOM 179 C ILE 22 10.254 11.403 7.246 1.00 0.00

ATOM 180 O ILE 22 9.577 10.509 7.757 1.00 0.00

ATOM 181 CB ILE 22 12.716 11.452 7.652 1.00 0.00

ATOM 182 CG1 ILE 22 14.070 10.918 7.182 1.00 0.00

ATOM 183 CG2 ILE 22 12.368 10.897 9.026 1.00 0.00

ATOM 184 CD1 ILE 22 15.243 11.441 7.980 1.00 0.00

ATOM 185 N ARG 23 9.856 12.669 7.189 1.00 0.00

ATOM 186 CA ARG 23 8.540 13.082 7.668 1.00 0.00

ATOM 187 C ARG 23 7.430 12.409 6.870 1.00 0.00

ATOM 188 O ARG 23 6.444 11.936 7.436 1.00 0.00

ATOM 189 CB ARG 23 8.382 14.595 7.678 1.00 0.00

ATOM 190 CG ARG 23 9.177 15.314 8.756 1.00 0.00

ATOM 191 CD ARG 23 8.807 16.741 8.941 1.00 0.00

ATOM 192 NE ARG 23 9.082 17.588 7.792 1.00 0.00

ATOM 193 CZ ARG 23 10.261 18.195 7.553 1.00 0.00

ATOM 194 NH1 ARG 23 11.263 18.083 8.396 1.00 0.00

ATOM 195 NH2 ARG 23 10.376 18.927 6.458 1.00 0.00

ATOM 196 N GLN 24 7.597 12.369 5.552 1.00 0.00

ATOM 197 CA GLN 24 6.596 11.780 4.671 1.00 0.00

ATOM 198 C GLN 24 6.452 10.283 4.922 1.00 0.00

ATOM 199 O GLN 24 5.356 9.729 4.827 1.00 0.00

ATOM 200 CB GLN 24 6.961 12.025 3.205 1.00 0.00

ATOM 201 CG GLN 24 6.895 13.482 2.782 1.00 0.00

ATOM 202 CD GLN 24 5.503 14.066 2.929 1.00 0.00

ATOM 203 OE1 GLN 24 4.509 13.441 2.547 1.00 0.00

ATOM 204 NE2 GLN 24 5.422 15.271 3.481 1.00 0.00

ATOM 205 N ALA 25 7.565 9.633 5.244 1.00 0.00

ATOM 206 CA ALA 25 7.566 8.201 5.511 1.00 0.00

ATOM 207 C ALA 25 6.914 7.890 6.853 1.00 0.00

ATOM 208 O ALA 25 6.119 6.959 6.968 1.00 0.00

ATOM 209 CB ALA 25 8.986 7.654 5.469 1.00 0.00

ATOM 210 N ARG 26 7.255 8.680 7.866 1.00 0.00

ATOM 211 CA ARG 26 6.682 8.508 9.197 1.00 0.00

ATOM 212 C ARG 26 5.180 8.749 9.185 1.00 0.00

ATOM 213 O ARG 26 4.435 8.140 9.954 1.00 0.00

ATOM 214 CB ARG 26 7.377 9.373 10.238 1.00 0.00

ATOM 215 CG ARG 26 8.789 8.936 10.593 1.00 0.00

ATOM 216 CD ARG 26 9.485 9.833 11.551 1.00 0.00

ATOM 217 NE ARG 26 10.840 9.423 11.885 1.00 0.00

ATOM 218 CZ ARG 26 11.691 10.141 12.643 1.00 0.00

ATOM 219 NH1 ARG 26 11.348 11.318 13.118 1.00 0.00

ATOM 220 NH2 ARG 26 12.892 9.640 12.879 1.00 0.00

ATOM 221 N LYS 27 4.736 9.644 8.307 1.00 0.00

ATOM 222 CA LYS 27 3.314 9.931 8.160 1.00 0.00

ATOM 223 C LYS 27 2.514 8.654 7.935 1.00 0.00

ATOM 224 O LYS 27 1.369 8.542 8.373 1.00 0.00

ATOM 225 CB LYS 27 3.081 10.907 7.004 1.00 0.00

ATOM 226 CG LYS 27 1.623 11.286 6.785 1.00 0.00

ATOM 227 CD LYS 27 1.480 12.314 5.672 1.00 0.00

ATOM 228 CE LYS 27 0.023 12.693 5.451 1.00 0.00

ATOM 229 NZ LYS 27 -0.133 13.692 4.358 1.00 0.00

ATOM 230 N PHE 28 3.124 7.694 7.250 1.00 0.00

ATOM 231 CA PHE 28 2.450 6.443 6.919 1.00 0.00

ATOM 232 C PHE 28 3.091 5.264 7.640 1.00 0.00

ATOM 233 O PHE 28 2.928 4.114 7.236 1.00 0.00

ATOM 234 CB PHE 28 2.470 6.209 5.407 1.00 0.00

ATOM 235 CG PHE 28 1.745 7.264 4.621 1.00 0.00

ATOM 236 CD1 PHE 28 2.445 8.274 3.978 1.00 0.00

ATOM 237 CD2 PHE 28 0.362 7.250 4.524 1.00 0.00

ATOM 238 CE1 PHE 28 1.779 9.246 3.255 1.00 0.00

ATOM 239 CE2 PHE 28 -0.307 8.218 3.801 1.00 0.00

ATOM 240 CZ PHE 28 0.403 9.218 3.166 1.00 0.00

ATOM 241 N ALA 29 3.821 5.558 8.711 1.00 0.00

ATOM 242 CA ALA 29 4.516 4.526 9.472 1.00 0.00

ATOM 243 C ALA 29 5.287 3.588 8.553 1.00 0.00

ATOM 244 O ALA 29 5.240 2.368 8.714 1.00 0.00

ATOM 245 CB ALA 29 3.528 3.745 10.325 1.00 0.00

ATOM 246 N GLY 30 5.997 4.165 7.587 1.00 0.00

ATOM 247 CA GLY 30 6.806 3.384 6.661 1.00 0.00

ATOM 248 C GLY 30 8.243 3.264 7.149 1.00 0.00

ATOM 249 O GLY 30 8.541 3.550 8.309 1.00 0.00

ATOM 250 N THR 31 9.132 2.840 6.259 1.00 0.00

ATOM 251 CA THR 31 10.561 2.817 6.551 1.00 0.00

ATOM 252 C THR 31 11.344 3.645 5.539 1.00 0.00

ATOM 253 O THR 31 11.150 3.513 4.330 1.00 0.00

ATOM 254 CB THR 31 11.112 1.379 6.560 1.00 0.00

ATOM 255 OG1 THR 31 10.437 0.612 7.567 1.00 0.00

ATOM 256 CG2 THR 31 12.605 1.382 6.848 1.00 0.00

ATOM 257 N VAL 32 12.230 4.499 6.038 1.00 0.00

ATOM 258 CA VAL 32 13.007 5.385 5.181 1.00 0.00

ATOM 259 C VAL 32 14.502 5.202 5.408 1.00 0.00

ATOM 260 O VAL 32 14.984 5.296 6.536 1.00 0.00

ATOM 261 CB VAL 32 12.639 6.863 5.415 1.00 0.00

ATOM 262 CG1 VAL 32 12.871 7.244 6.869 1.00 0.00

ATOM 263 CG2 VAL 32 13.446 7.764 4.494 1.00 0.00

ATOM 264 N THR 33 15.231 4.938 4.328 1.00 0.00

ATOM 265 CA THR 33 16.679 4.792 4.399 1.00 0.00

ATOM 266 C THR 33 17.372 5.686 3.378 1.00 0.00

ATOM 267 O THR 33 16.817 5.981 2.319 1.00 0.00

ATOM 268 CB THR 33 17.112 3.333 4.167 1.00 0.00

ATOM 269 OG1 THR 33 16.706 2.914 2.856 1.00 0.00

ATOM 270 CG2 THR 33 16.481 2.418 5.204 1.00 0.00

ATOM 271 N TYR 34 18.588 6.115 3.703 1.00 0.00

ATOM 272 CA TYR 34 19.319 7.056 2.861 1.00 0.00

ATOM 273 C TYR 34 20.798 6.702 2.798 1.00 0.00

ATOM 274 O TYR 34 21.368 6.198 3.767 1.00 0.00

ATOM 275 CB TYR 34 19.140 8.485 3.377 1.00 0.00

ATOM 276 CG TYR 34 19.766 8.730 4.733 1.00 0.00

ATOM 277 CD1 TYR 34 21.067 9.196 4.845 1.00 0.00

ATOM 278 CD2 TYR 34 19.051 8.495 5.898 1.00 0.00

ATOM 279 CE1 TYR 34 21.643 9.419 6.080 1.00 0.00

ATOM 280 CE2 TYR 34 19.616 8.716 7.139 1.00 0.00

ATOM 281 CZ TYR 34 20.913 9.178 7.226 1.00 0.00

ATOM 282 OH TYR 34 21.480 9.402 8.460 1.00 0.00

ATOM 283 N THR 35 21.418 6.973 1.654 1.00 0.00

ATOM 284 CA THR 35 22.850 6.752 1.487 1.00 0.00

ATOM 285 C THR 35 23.475 7.827 0.609 1.00 0.00

ATOM 286 O THR 35 23.093 7.998 -0.548 1.00 0.00

ATOM 287 CB THR 35 23.140 5.370 0.872 1.00 0.00

ATOM 288 OG1 THR 35 22.585 4.345 1.708 1.00 0.00

ATOM 289 CG2 THR 35 24.639 5.150 0.735 1.00 0.00

ATOM 290 N LEU 36 24.431 8.550 1.169 1.00 0.00

ATOM 291 CA LEU 36 25.077 9.646 0.454 1.00 0.00

ATOM 292 C LEU 36 26.330 9.170 -0.267 1.00 0.00

ATOM 293 O LEU 36 27.245 8.624 0.352 1.00 0.00

ATOM 294 CB LEU 36 25.418 10.784 1.424 1.00 0.00

ATOM 295 CG LEU 36 26.136 11.985 0.794 1.00 0.00

ATOM 296 CD1 LEU 36 25.223 12.669 -0.213 1.00 0.00

ATOM 297 CD2 LEU 36 26.557 12.956 1.888 1.00 0.00

ATOM 298 N ASP 37 26.368 9.384 -1.579 1.00 0.00

ATOM 299 CA ASP 37 27.531 9.018 -2.380 1.00 0.00

ATOM 300 C ASP 37 28.036 10.205 -3.188 1.00 0.00

ATOM 301 O ASP 37 27.536 10.486 -4.280 1.00 0.00

ATOM 302 CB ASP 37 27.194 7.851 -3.311 1.00 0.00

ATOM 303 CG ASP 37 28.368 7.350 -4.141 1.00 0.00

ATOM 304 OD1 ASP 37 29.422 7.935 -4.059 1.00 0.00

ATOM 305 OD2 ASP 37 28.250 6.300 -4.726 1.00 0.00

ATOM 306 N GLY 38 29.029 10.905 -2.648 1.00 0.00

ATOM 307 CA GLY 38 29.526 12.131 -3.261 1.00 0.00

ATOM 308 C GLY 38 28.454 13.214 -3.272 1.00 0.00

ATOM 309 O GLY 38 28.024 13.686 -2.219 1.00 0.00

ATOM 310 N ASN 39 28.028 13.604 -4.466 1.00 0.00

ATOM 311 CA ASN 39 26.981 14.609 -4.615 1.00 0.00

ATOM 312 C ASN 39 25.658 13.967 -5.020 1.00 0.00

ATOM 313 O ASN 39 24.870 14.565 -5.755 1.00 0.00

ATOM 314 CB ASN 39 27.375 15.678 -5.617 1.00 0.00

ATOM 315 CG ASN 39 27.580 15.154 -7.011 1.00 0.00

ATOM 316 OD1 ASN 39 27.598 13.940 -7.244 1.00 0.00

ATOM 317 ND2 ASN 39 27.816 16.060 -7.926 1.00 0.00

ATOM 318 N ASP 40 25.419 12.757 -4.532 1.00 0.00

ATOM 319 CA ASP 40 24.205 12.022 -4.867 1.00 0.00

ATOM 320 C ASP 40 23.633 11.317 -3.643 1.00 0.00

ATOM 321 O ASP 40 24.155 10.290 -3.208 1.00 0.00

ATOM 322 CB ASP 40 24.483 11.006 -5.978 1.00 0.00

ATOM 323 CG ASP 40 23.256 10.232 -6.443 1.00 0.00

ATOM 324 OD1 ASP 40 22.214 10.399 -5.855 1.00 0.00

ATOM 325 OD2 ASP 40 23.330 9.604 -7.472 1.00 0.00

ATOM 326 N LEU 41 22.556 11.872 -3.096 1.00 0.00

ATOM 327 CA LEU 41 21.886 11.275 -1.949 1.00 0.00

ATOM 328 C LEU 41 20.741 10.372 -2.388 1.00 0.00

ATOM 329 O LEU 41 19.735 10.845 -2.917 1.00 0.00

ATOM 330 CB LEU 41 21.371 12.370 -1.007 1.00 0.00

ATOM 331 CG LEU 41 20.620 11.865 0.232 1.00 0.00

ATOM 332 CD1 LEU 41 21.554 11.045 1.111 1.00 0.00

ATOM 333 CD2 LEU 41 20.055 13.049 1.002 1.00 0.00

ATOM 334 N GLU 42 20.898 9.071 -2.166 1.00 0.00

ATOM 335 CA GLU 42 19.859 8.105 -2.502 1.00 0.00

ATOM 336 C GLU 42 18.936 7.853 -1.318 1.00 0.00

ATOM 337 O GLU 42 19.371 7.370 -0.271 1.00 0.00

ATOM 338 CB GLU 42 20.482 6.789 -2.972 1.00 0.00

ATOM 339 CG GLU 42 19.474 5.733 -3.402 1.00 0.00

ATOM 340 CD GLU 42 20.159 4.473 -3.850 1.00 0.00

ATOM 341 OE1 GLU 42 20.915 3.925 -3.083 1.00 0.00

ATOM 342 OE2 GLU 42 19.844 3.995 -4.914 1.00 0.00

ATOM 343 N ILE 43 17.661 8.182 -1.487 1.00 0.00

ATOM 344 CA ILE 43 16.670 7.983 -0.433 1.00 0.00

ATOM 345 C ILE 43 15.593 6.999 -0.866 1.00 0.00

ATOM 346 O ILE 43 14.820 7.275 -1.785 1.00 0.00

ATOM 347 CB ILE 43 16.005 9.311 -0.026 1.00 0.00

ATOM 348 CG1 ILE 43 17.054 10.299 0.491 1.00 0.00

ATOM 349 CG2 ILE 43 14.933 9.070 1.025 1.00 0.00

ATOM 350 CD1 ILE 43 16.521 11.694 0.719 1.00 0.00

ATOM 351 N ARG 44 15.548 5.850 -0.202 1.00 0.00

ATOM 352 CA ARG 44 14.543 4.834 -0.495 1.00 0.00

ATOM 353 C ARG 44 13.500 4.755 0.610 1.00 0.00

ATOM 354 O ARG 44 13.829 4.536 1.773 1.00 0.00

ATOM 355 CB ARG 44 15.166 3.474 -0.769 1.00 0.00

ATOM 356 CG ARG 44 16.012 3.400 -2.031 1.00 0.00

ATOM 357 CD ARG 44 16.700 2.099 -2.232 1.00 0.00

ATOM 358 NE ARG 44 17.491 2.018 -3.449 1.00 0.00

ATOM 359 CZ ARG 44 18.153 0.918 -3.860 1.00 0.00

ATOM 360 NH1 ARG 44 18.152 -0.181 -3.139 1.00 0.00

ATOM 361 NH2 ARG 44 18.823 0.981 -4.997 1.00 0.00

ATOM 362 N ILE 45 12.235 4.938 0.237 1.00 0.00

ATOM 363 CA ILE 45 11.129 4.766 1.172 1.00 0.00

ATOM 364 C ILE 45 10.271 3.567 0.793 1.00 0.00

ATOM 365 O ILE 45 9.765 3.481 -0.324 1.00 0.00

ATOM 366 CB ILE 45 10.241 6.023 1.233 1.00 0.00

ATOM 367 CG1 ILE 45 11.056 7.229 1.707 1.00 0.00

ATOM 368 CG2 ILE 45 9.050 5.788 2.149 1.00 0.00

ATOM 369 CD1 ILE 45 10.322 8.546 1.590 1.00 0.00

ATOM 370 N THR 46 10.113 2.639 1.734 1.00 0.00

ATOM 371 CA THR 46 9.249 1.481 1.529 1.00 0.00

ATOM 372 C THR 46 8.207 1.371 2.637 1.00 0.00

ATOM 373 O THR 46 8.230 2.133 3.602 1.00 0.00

ATOM 374 CB THR 46 10.060 0.174 1.468 1.00 0.00

ATOM 375 OG1 THR 46 10.682 -0.065 2.738 1.00 0.00

ATOM 376 CG2 THR 46 11.131 0.261 0.392 1.00 0.00

ATOM 377 N GLY 47 7.298 0.414 2.491 1.00 0.00

ATOM 378 CA GLY 47 6.220 0.227 3.454 1.00 0.00

ATOM 379 C GLY 47 5.056 1.165 3.168 1.00 0.00

ATOM 380 O GLY 47 4.185 1.367 4.015 1.00 0.00

ATOM 381 N VAL 48 5.041 1.734 1.968 1.00 0.00

ATOM 382 CA VAL 48 3.946 2.594 1.537 1.00 0.00

ATOM 383 C VAL 48 3.352 2.110 0.220 1.00 0.00

ATOM 384 O VAL 48 4.014 1.419 -0.555 1.00 0.00

ATOM 385 CB VAL 48 4.404 4.056 1.377 1.00 0.00

ATOM 386 CG1 VAL 48 4.879 4.614 2.710 1.00 0.00

ATOM 387 CG2 VAL 48 5.506 4.158 0.334 1.00 0.00

ATOM 388 N PRO 49 2.100 2.478 -0.028 1.00 0.00

ATOM 389 CA PRO 49 1.412 2.075 -1.249 1.00 0.00

ATOM 390 C PRO 49 1.809 2.962 -2.422 1.00 0.00

ATOM 391 O PRO 49 2.370 4.043 -2.235 1.00 0.00

ATOM 392 CB PRO 49 -0.080 2.208 -0.902 1.00 0.00

ATOM 393 CG PRO 49 -0.149 3.337 0.090 1.00 0.00

ATOM 394 CD PRO 49 1.165 2.926 1.013 1.00 0.00

ATOM 395 N GLU 50 1.497 2.566 -3.639 1.00 0.00

ATOM 396 CA GLU 50 1.771 3.230 -4.909 1.00 0.00

ATOM 397 C GLU 50 1.338 4.690 -4.871 1.00 0.00

ATOM 398 O GLU 50 2.084 5.576 -5.288 1.00 0.00

ATOM 399 CB GLU 50 1.068 2.502 -6.056 1.00 0.00

ATOM 400 CG GLU 50 1.325 3.101 -7.432 1.00 0.00

ATOM 401 CD GLU 50 0.642 2.306 -8.510 1.00 0.00

ATOM 402 OE1 GLU 50 0.025 1.317 -8.192 1.00 0.00

ATOM 403 OE2 GLU 50 0.642 2.746 -9.635 1.00 0.00

ATOM 404 N GLN 51 0.132 4.933 -4.371 1.00 0.00

ATOM 405 CA GLN 51 -0.390 6.288 -4.253 1.00 0.00

ATOM 406 C GLN 51 0.571 7.182 -3.478 1.00 0.00

ATOM 407 O GLN 51 0.795 8.335 -3.847 1.00 0.00

ATOM 408 CB GLN 51 -1.756 6.278 -3.563 1.00 0.00

ATOM 409 CG GLN 51 -2.422 7.641 -3.480 1.00 0.00

ATOM 410 CD GLN 51 -2.803 8.183 -4.844 1.00 0.00

ATOM 411 OE1 GLN 51 -3.364 7.466 -5.678 1.00 0.00

ATOM 412 NE2 GLN 51 -2.497 9.453 -5.082 1.00 0.00

ATOM 413 N VAL 52 1.135 6.643 -2.404 1.00 0.00

ATOM 414 CA VAL 52 2.058 7.398 -1.564 1.00 0.00

ATOM 415 C VAL 52 3.393 7.612 -2.268 1.00 0.00

ATOM 416 O VAL 52 3.990 8.683 -2.173 1.00 0.00

ATOM 417 CB VAL 52 2.306 6.690 -0.219 1.00 0.00

ATOM 418 CG1 VAL 52 3.403 7.399 0.562 1.00 0.00

ATOM 419 CG2 VAL 52 1.023 6.635 0.598 1.00 0.00

ATOM 420 N ARG 53 3.856 6.584 -2.972 1.00 0.00

ATOM 421 CA ARG 53 5.096 6.674 -3.733 1.00 0.00

ATOM 422 C ARG 53 5.016 7.769 -4.789 1.00 0.00

ATOM 423 O ARG 53 6.001 8.454 -5.064 1.00 0.00

ATOM 424 CB ARG 53 5.493 5.338 -4.343 1.00 0.00

ATOM 425 CG ARG 53 5.771 4.233 -3.338 1.00 0.00

ATOM 426 CD ARG 53 6.425 3.031 -3.915 1.00 0.00

ATOM 427 NE ARG 53 5.616 2.311 -4.884 1.00 0.00

ATOM 428 CZ ARG 53 4.721 1.351 -4.573 1.00 0.00

ATOM 429 NH1 ARG 53 4.547 0.967 -3.328 1.00 0.00

ATOM 430 NH2 ARG 53 4.042 0.790 -5.557 1.00 0.00

ATOM 431 N LYS 54 3.837 7.927 -5.379 1.00 0.00

ATOM 432 CA LYS 54 3.596 9.007 -6.331 1.00 0.00

ATOM 433 C LYS 54 3.680 10.368 -5.652 1.00 0.00

ATOM 434 O LYS 54 4.263 11.307 -6.195 1.00 0.00

ATOM 435 CB LYS 54 2.231 8.837 -7.000 1.00 0.00

ATOM 436 CG LYS 54 2.148 7.666 -7.969 1.00 0.00

ATOM 437 CD LYS 54 0.765 7.561 -8.594 1.00 0.00

ATOM 438 CE LYS 54 0.671 6.377 -9.543 1.00 0.00

ATOM 439 NZ LYS 54 -0.686 6.243 -10.136 1.00 0.00

ATOM 440 N GLU 55 3.097 10.467 -4.463 1.00 0.00

ATOM 441 CA GLU 55 3.125 11.709 -3.698 1.00 0.00

ATOM 442 C GLU 55 4.540 12.046 -3.244 1.00 0.00

ATOM 443 O GLU 55 4.918 13.214 -3.167 1.00 0.00

ATOM 444 CB GLU 55 2.194 11.613 -2.487 1.00 0.00

ATOM 445 CG GLU 55 0.712 11.592 -2.834 1.00 0.00

ATOM 446 CD GLU 55 0.312 12.823 -3.599 1.00 0.00

ATOM 447 OE1 GLU 55 0.596 13.903 -3.141 1.00 0.00

ATOM 448 OE2 GLU 55 -0.177 12.683 -4.696 1.00 0.00

ATOM 449 N LEU 56 5.321 11.012 -2.945 1.00 0.00

ATOM 450 CA LEU 56 6.733 11.188 -2.619 1.00 0.00

ATOM 451 C LEU 56 7.501 11.771 -3.796 1.00 0.00

ATOM 452 O LEU 56 8.344 12.653 -3.623 1.00 0.00

ATOM 453 CB LEU 56 7.348 9.850 -2.192 1.00 0.00

ATOM 454 CG LEU 56 6.869 9.312 -0.838 1.00 0.00

ATOM 455 CD1 LEU 56 7.405 7.905 -0.615 1.00 0.00

ATOM 456 CD2 LEU 56 7.332 10.248 0.271 1.00 0.00

ATOM 457 N ALA 57 7.206 11.280 -4.994 1.00 0.00

ATOM 458 CA ALA 57 7.831 11.790 -6.208 1.00 0.00

ATOM 459 C ALA 57 7.469 13.249 -6.444 1.00 0.00

ATOM 460 O ALA 57 8.308 14.050 -6.859 1.00 0.00

ATOM 461 CB ALA 57 7.432 10.941 -7.407 1.00 0.00

ATOM 462 N LYS 58 6.212 13.591 -6.178 1.00 0.00

ATOM 463 CA LYS 58 5.756 14.972 -6.284 1.00 0.00

ATOM 464 C LYS 58 6.521 15.882 -5.334 1.00 0.00

ATOM 465 O LYS 58 6.948 16.975 -5.712 1.00 0.00

ATOM 466 CB LYS 58 4.255 15.063 -6.003 1.00 0.00

ATOM 467 CG LYS 58 3.374 14.454 -7.086 1.00 0.00

ATOM 468 CD LYS 58 1.899 14.582 -6.736 1.00 0.00

ATOM 469 CE LYS 58 1.019 13.950 -7.805 1.00 0.00

ATOM 470 NZ LYS 58 -0.426 14.039 -7.460 1.00 0.00

ATOM 471 N GLU 59 6.692 15.428 -4.098 1.00 0.00

ATOM 472 CA GLU 59 7.436 16.185 -3.098 1.00 0.00

ATOM 473 C GLU 59 8.910 16.292 -3.474 1.00 0.00

ATOM 474 O GLU 59 9.552 17.310 -3.214 1.00 0.00

ATOM 475 CB GLU 59 7.290 15.538 -1.719 1.00 0.00

ATOM 476 CG GLU 59 5.901 15.664 -1.109 1.00 0.00

ATOM 477 CD GLU 59 5.497 17.104 -0.963 1.00 0.00

ATOM 478 OE1 GLU 59 6.249 17.858 -0.393 1.00 0.00

ATOM 479 OE2 GLU 59 4.490 17.477 -1.517 1.00 0.00

ATOM 480 N ALA 60 9.438 15.240 -4.089 1.00 0.00

ATOM 481 CA ALA 60 10.825 15.231 -4.534 1.00 0.00

ATOM 482 C ALA 60 11.080 16.323 -5.566 1.00 0.00

ATOM 483 O ALA 60 12.087 17.027 -5.503 1.00 0.00

ATOM 484 CB ALA 60 11.191 13.866 -5.101 1.00 0.00

ATOM 485 N GLU 61 10.160 16.457 -6.516 1.00 0.00

ATOM 486 CA GLU 61 10.267 17.485 -7.545 1.00 0.00

ATOM 487 C GLU 61 10.118 18.879 -6.950 1.00 0.00

ATOM 488 O GLU 61 10.860 19.798 -7.302 1.00 0.00

ATOM 489 CB GLU 61 9.216 17.264 -8.635 1.00 0.00

ATOM 490 CG GLU 61 9.467 16.045 -9.512 1.00 0.00

ATOM 491 CD GLU 61 8.330 15.818 -10.469 1.00 0.00

ATOM 492 OE1 GLU 61 7.362 16.538 -10.395 1.00 0.00

ATOM 493 OE2 GLU 61 8.473 15.001 -11.347 1.00 0.00

ATOM 494 N ARG 62 9.157 19.031 -6.046 1.00 0.00

ATOM 495 CA ARG 62 9.007 20.267 -5.288 1.00 0.00

ATOM 496 C ARG 62 10.313 20.661 -4.610 1.00 0.00

ATOM 497 O ARG 62 10.748 21.809 -4.700 1.00 0.00

ATOM 498 CB ARG 62 7.862 20.192 -4.288 1.00 0.00

ATOM 499 CG ARG 62 7.591 21.480 -3.529 1.00 0.00

ATOM 500 CD ARG 62 6.540 21.366 -2.485 1.00 0.00

ATOM 501 NE ARG 62 6.888 20.495 -1.373 1.00 0.00

ATOM 502 CZ ARG 62 7.706 20.838 -0.359 1.00 0.00

ATOM 503 NH1 ARG 62 8.292 22.015 -0.330 1.00 0.00

ATOM 504 NH2 ARG 62 7.924 19.951 0.595 1.00 0.00

ATOM 505 N LEU 63 10.935 19.702 -3.935 1.00 0.00

ATOM 506 CA LEU 63 12.157 19.962 -3.180 1.00 0.00

ATOM 507 C LEU 63 13.299 20.360 -4.107 1.00 0.00

ATOM 508 O LEU 63 14.036 21.307 -3.829 1.00 0.00

ATOM 509 CB LEU 63 12.542 18.729 -2.353 1.00 0.00

ATOM 510 CG LEU 63 11.606 18.411 -1.180 1.00 0.00

ATOM 511 CD1 LEU 63 11.851 16.991 -0.687 1.00 0.00

ATOM 512 CD2 LEU 63 11.832 19.417 -0.062 1.00 0.00

ATOM 513 N ALA 64 13.443 19.631 -5.209 1.00 0.00

ATOM 514 CA ALA 64 14.493 19.911 -6.181 1.00 0.00

ATOM 515 C ALA 64 14.352 21.315 -6.752 1.00 0.00

ATOM 516 O ALA 64 15.340 22.030 -6.919 1.00 0.00

ATOM 517 CB ALA 64 14.470 18.876 -7.296 1.00 0.00

ATOM 518 N LYS 65 13.119 21.707 -7.052 1.00 0.00

ATOM 519 CA LYS 65 12.847 23.024 -7.615 1.00 0.00

ATOM 520 C LYS 65 13.163 24.127 -6.612 1.00 0.00

ATOM 521 O LYS 65 13.706 25.171 -6.975 1.00 0.00

ATOM 522 CB LYS 65 11.389 23.125 -8.065 1.00 0.00

ATOM 523 CG LYS 65 11.050 22.288 -9.292 1.00 0.00

ATOM 524 CD LYS 65 9.589 22.442 -9.680 1.00 0.00

ATOM 525 CE LYS 65 9.242 21.582 -10.887 1.00 0.00

ATOM 526 NZ LYS 65 7.808 21.702 -11.263 1.00 0.00

ATOM 527 N GLU 66 12.822 23.889 -5.350 1.00 0.00

ATOM 528 CA GLU 66 13.103 24.847 -4.287 1.00 0.00

ATOM 529 C GLU 66 14.595 24.913 -3.988 1.00 0.00

ATOM 530 O GLU 66 15.115 25.958 -3.593 1.00 0.00

ATOM 531 CB GLU 66 12.327 24.483 -3.019 1.00 0.00

ATOM 532 CG GLU 66 10.823 24.689 -3.122 1.00 0.00

ATOM 533 CD GLU 66 10.122 24.226 -1.875 1.00 0.00

ATOM 534 OE1 GLU 66 10.780 23.714 -1.001 1.00 0.00

ATOM 535 OE2 GLU 66 8.948 24.484 -1.746 1.00 0.00

ATOM 536 N PHE 67 15.284 23.793 -4.181 1.00 0.00

ATOM 537 CA PHE 67 16.707 23.706 -3.881 1.00 0.00

ATOM 538 C PHE 67 17.550 23.956 -5.124 1.00 0.00

ATOM 539 O PHE 67 18.773 24.085 -5.042 1.00 0.00

ATOM 540 CB PHE 67 17.046 22.338 -3.283 1.00 0.00

ATOM 541 CG PHE 67 16.301 22.031 -2.015 1.00 0.00

ATOM 542 CD1 PHE 67 15.755 23.050 -1.250 1.00 0.00

ATOM 543 CD2 PHE 67 16.145 20.721 -1.585 1.00 0.00

ATOM 544 CE1 PHE 67 15.071 22.769 -0.082 1.00 0.00

ATOM 545 CE2 PHE 67 15.461 20.437 -0.419 1.00 0.00

ATOM 546 CZ PHE 67 14.923 21.461 0.333 1.00 0.00

ATOM 547 N ASN 68 16.892 24.023 -6.276 1.00 0.00

ATOM 548 CA ASN 68 17.580 24.241 -7.541 1.00 0.00

ATOM 549 C ASN 68 18.586 23.131 -7.819 1.00 0.00

ATOM 550 O ASN 68 19.724 23.392 -8.209 1.00 0.00

ATOM 551 CB ASN 68 18.270 25.592 -7.573 1.00 0.00

ATOM 552 CG ASN 68 18.604 26.068 -8.960 1.00 0.00

ATOM 553 OD1 ASN 68 17.935 25.717 -9.936 1.00 0.00

ATOM 554 ND2 ASN 68 19.687 26.798 -9.060 1.00 0.00

ATOM 555 N ILE 69 18.159 21.890 -7.616 1.00 0.00

ATOM 556 CA ILE 69 19.020 20.734 -7.851 1.00 0.00

ATOM 557 C ILE 69 18.368 19.750 -8.812 1.00 0.00

ATOM 558 O ILE 69 17.269 19.988 -9.310 1.00 0.00

ATOM 559 CB ILE 69 19.361 20.008 -6.537 1.00 0.00

ATOM 560 CG1 ILE 69 18.085 19.494 -5.864 1.00 0.00

ATOM 561 CG2 ILE 69 20.126 20.930 -5.601 1.00 0.00

ATOM 562 CD1 ILE 69 18.340 18.589 -4.682 1.00 0.00

ATOM 563 N THR 70 19.054 18.640 -9.068 1.00 0.00

ATOM 564 CA THR 70 18.531 17.611 -9.959 1.00 0.00

ATOM 565 C THR 70 17.931 16.453 -9.172 1.00 0.00

ATOM 566 O THR 70 18.519 15.981 -8.200 1.00 0.00

ATOM 567 CB THR 70 19.624 17.067 -10.898 1.00 0.00

ATOM 568 OG1 THR 70 20.126 18.131 -11.718 1.00 0.00

ATOM 569 CG2 THR 70 19.065 15.968 -11.787 1.00 0.00

ATOM 570 N VAL 71 16.758 16.001 -9.600 1.00 0.00

ATOM 571 CA VAL 71 16.081 14.886 -8.946 1.00 0.00

ATOM 572 C VAL 71 15.757 13.777 -9.938 1.00 0.00

ATOM 573 O VAL 71 15.114 14.015 -10.961 1.00 0.00

ATOM 574 CB VAL 71 14.780 15.339 -8.256 1.00 0.00

ATOM 575 CG1 VAL 71 13.830 15.960 -9.270 1.00 0.00

ATOM 576 CG2 VAL 71 14.115 14.167 -7.552 1.00 0.00

ATOM 577 N THR 72 16.210 12.566 -9.633 1.00 0.00

ATOM 578 CA THR 72 15.834 11.389 -10.408 1.00 0.00

ATOM 579 C THR 72 15.231 10.309 -9.516 1.00 0.00

ATOM 580 O THR 72 15.900 9.780 -8.630 1.00 0.00

ATOM 581 CB THR 72 17.040 10.802 -11.164 1.00 0.00

ATOM 582 OG1 THR 72 17.577 11.789 -12.054 1.00 0.00

ATOM 583 CG2 THR 72 16.623 9.577 -11.964 1.00 0.00

ATOM 584 N TYR 73 13.965 9.990 -9.756 1.00 0.00

ATOM 585 CA TYR 73 13.225 9.086 -8.884 1.00 0.00

ATOM 586 C TYR 73 12.648 7.914 -9.666 1.00 0.00

ATOM 587 O TYR 73 12.282 8.055 -10.834 1.00 0.00

ATOM 588 CB TYR 73 12.104 9.837 -8.162 1.00 0.00

ATOM 589 CG TYR 73 11.095 10.475 -9.091 1.00 0.00

ATOM 590 CD1 TYR 73 10.035 9.741 -9.604 1.00 0.00

ATOM 591 CD2 TYR 73 11.206 11.810 -9.452 1.00 0.00

ATOM 592 CE1 TYR 73 9.111 10.320 -10.453 1.00 0.00

ATOM 593 CE2 TYR 73 10.288 12.398 -10.300 1.00 0.00

ATOM 594 CZ TYR 73 9.241 11.649 -10.799 1.00 0.00

ATOM 595 OH TYR 73 8.324 12.230 -11.643 1.00 0.00

ATOM 596 N THR 74 12.571 6.758 -9.020 1.00 0.00

ATOM 597 CA THR 74 12.017 5.561 -9.646 1.00 0.00

ATOM 598 C THR 74 11.217 4.739 -8.647 1.00 0.00

ATOM 599 O THR 74 11.728 4.343 -7.601 1.00 0.00

ATOM 600 CB THR 74 13.123 4.678 -10.254 1.00 0.00

ATOM 601 OG1 THR 74 13.862 5.433 -11.224 1.00 0.00

ATOM 602 CG2 THR 74 12.520 3.453 -10.923 1.00 0.00

ATOM 603 N ILE 75 9.955 4.479 -8.978 1.00 0.00

ATOM 604 CA ILE 75 9.221 3.365 -8.387 1.00 0.00

ATOM 605 C ILE 75 9.718 2.031 -8.930 1.00 0.00

ATOM 606 O ILE 75 9.415 1.660 -10.064 1.00 0.00

ATOM 607 CB ILE 75 7.708 3.485 -8.647 1.00 0.00

ATOM 608 CG1 ILE 75 7.154 4.756 -7.999 1.00 0.00

ATOM 609 CG2 ILE 75 6.980 2.256 -8.123 1.00 0.00

ATOM 610 CD1 ILE 75 5.719 5.054 -8.368 1.00 0.00

ATOM 611 N ARG 76 10.479 1.313 -8.111 1.00 0.00

ATOM 612 CA ARG 76 11.191 0.128 -8.572 1.00 0.00

ATOM 613 C ARG 76 10.275 -1.087 -8.604 1.00 0.00

ATOM 614 O ARG 76 10.421 -1.967 -9.456 1.00 0.00

ATOM 615 CB ARG 76 12.446 -0.141 -7.755 1.00 0.00

ATOM 616 CG ARG 76 13.562 0.874 -7.943 1.00 0.00

ATOM 617 CD ARG 76 14.849 0.495 -7.304 1.00 0.00

ATOM 618 NE ARG 76 15.888 1.508 -7.396 1.00 0.00

ATOM 619 CZ ARG 76 16.675 1.702 -8.471 1.00 0.00

ATOM 620 NH1 ARG 76 16.572 0.933 -9.532 1.00 0.00

ATOM 621 NH2 ARG 76 17.572 2.672 -8.423 1.00 0.00

TER

ENDMDL

**PDB ID 2LV8**

ATOM 1 N LEU 1 0.000 0.000 0.000 1.00 0.00

ATOM 2 CA LEU 1 0.766 1.240 0.000 1.00 0.00

ATOM 3 C LEU 1 0.943 1.779 -1.414 1.00 0.00

ATOM 4 O LEU 1 1.995 2.318 -1.756 1.00 0.00

ATOM 5 CB LEU 1 2.132 1.019 0.662 1.00 0.00

ATOM 6 CG LEU 1 2.082 0.532 2.116 1.00 0.00

ATOM 7 CD1 LEU 1 3.490 0.250 2.621 1.00 0.00

ATOM 8 CD2 LEU 1 1.399 1.581 2.980 1.00 0.00

ATOM 9 N LEU 2 -0.093 1.630 -2.232 1.00 0.00

ATOM 10 CA LEU 2 -0.050 2.089 -3.616 1.00 0.00

ATOM 11 C LEU 2 -1.151 3.103 -3.893 1.00 0.00

ATOM 12 O LEU 2 -2.336 2.769 -3.879 1.00 0.00

ATOM 13 CB LEU 2 -0.168 0.898 -4.574 1.00 0.00

ATOM 14 CG LEU 2 0.940 -0.156 -4.446 1.00 0.00

ATOM 15 CD1 LEU 2 0.633 -1.347 -5.343 1.00 0.00

ATOM 16 CD2 LEU 2 2.278 0.466 -4.814 1.00 0.00

ATOM 17 N TYR 3 -0.754 4.346 -4.147 1.00 0.00

ATOM 18 CA TYR 3 -1.706 5.410 -4.441 1.00 0.00

ATOM 19 C TYR 3 -1.356 6.120 -5.742 1.00 0.00

ATOM 20 O TYR 3 -0.183 6.345 -6.041 1.00 0.00

ATOM 21 CB TYR 3 -1.755 6.418 -3.290 1.00 0.00

ATOM 22 CG TYR 3 -2.439 5.895 -2.046 1.00 0.00

ATOM 23 CD1 TYR 3 -1.783 5.029 -1.183 1.00 0.00

ATOM 24 CD2 TYR 3 -3.738 6.271 -1.738 1.00 0.00

ATOM 25 CE1 TYR 3 -2.403 4.549 -0.046 1.00 0.00

ATOM 26 CE2 TYR 3 -4.367 5.798 -0.603 1.00 0.00

ATOM 27 CZ TYR 3 -3.697 4.936 0.241 1.00 0.00

ATOM 28 OH TYR 3 -4.319 4.462 1.373 1.00 0.00

ATOM 29 N VAL 4 -2.380 6.470 -6.515 1.00 0.00

ATOM 30 CA VAL 4 -2.185 7.201 -7.760 1.00 0.00

ATOM 31 C VAL 4 -3.029 8.469 -7.792 1.00 0.00

ATOM 32 O VAL 4 -4.237 8.427 -7.560 1.00 0.00

ATOM 33 CB VAL 4 -2.533 6.334 -8.985 1.00 0.00

ATOM 34 CG1 VAL 4 -2.357 7.131 -10.269 1.00 0.00

ATOM 35 CG2 VAL 4 -1.669 5.082 -9.015 1.00 0.00

ATOM 36 N LEU 5 -2.386 9.595 -8.080 1.00 0.00

ATOM 37 CA LEU 5 -3.077 10.877 -8.145 1.00 0.00

ATOM 38 C LEU 5 -3.161 11.386 -9.579 1.00 0.00

ATOM 39 O LEU 5 -2.150 11.466 -10.279 1.00 0.00

ATOM 40 CB LEU 5 -2.370 11.906 -7.253 1.00 0.00

ATOM 41 CG LEU 5 -2.090 11.443 -5.818 1.00 0.00

ATOM 42 CD1 LEU 5 -1.315 12.517 -5.065 1.00 0.00

ATOM 43 CD2 LEU 5 -3.405 11.136 -5.117 1.00 0.00

ATOM 44 N ILE 6 -4.369 11.728 -10.011 1.00 0.00

ATOM 45 CA ILE 6 -4.587 12.221 -11.366 1.00 0.00

ATOM 46 C ILE 6 -4.786 13.731 -11.377 1.00 0.00

ATOM 47 O ILE 6 -5.626 14.262 -10.651 1.00 0.00

ATOM 48 CB ILE 6 -5.804 11.546 -12.023 1.00 0.00

ATOM 49 CG1 ILE 6 -5.639 10.024 -12.019 1.00 0.00

ATOM 50 CG2 ILE 6 -5.995 12.060 -13.442 1.00 0.00

ATOM 51 CD1 ILE 6 -4.417 9.541 -12.765 1.00 0.00

ATOM 52 N ILE 7 -4.007 14.418 -12.207 1.00 0.00

ATOM 53 CA ILE 7 -4.118 15.866 -12.338 1.00 0.00

ATOM 54 C ILE 7 -4.347 16.271 -13.789 1.00 0.00

ATOM 55 O ILE 7 -3.396 16.491 -14.539 1.00 0.00

ATOM 56 CB ILE 7 -2.862 16.579 -11.806 1.00 0.00

ATOM 57 CG1 ILE 7 -2.498 16.054 -10.415 1.00 0.00

ATOM 58 CG2 ILE 7 -3.079 18.084 -11.771 1.00 0.00

ATOM 59 CD1 ILE 7 -1.316 16.757 -9.787 1.00 0.00

ATOM 60 N SER 8 -5.613 16.369 -14.178 1.00 0.00

ATOM 61 CA SER 8 -5.968 16.708 -15.551 1.00 0.00

ATOM 62 C SER 8 -7.465 16.952 -15.687 1.00 0.00

ATOM 63 O SER 8 -8.269 16.361 -14.967 1.00 0.00

ATOM 64 CB SER 8 -5.526 15.605 -16.493 1.00 0.00

ATOM 65 OG SER 8 -5.936 15.842 -17.811 1.00 0.00

ATOM 66 N ASN 9 -7.834 17.828 -16.616 1.00 0.00

ATOM 67 CA ASN 9 -9.238 18.131 -16.868 1.00 0.00

ATOM 68 C ASN 9 -9.855 17.126 -17.832 1.00 0.00

ATOM 69 O ASN 9 -11.050 17.180 -18.119 1.00 0.00

ATOM 70 CB ASN 9 -9.414 19.543 -17.398 1.00 0.00

ATOM 71 CG ASN 9 -9.154 20.611 -16.373 1.00 0.00

ATOM 72 OD1 ASN 9 -9.273 20.382 -15.164 1.00 0.00

ATOM 73 ND2 ASN 9 -8.880 21.798 -16.852 1.00 0.00

ATOM 74 N ASP 10 -9.031 16.209 -18.329 1.00 0.00

ATOM 75 CA ASP 10 -9.494 15.190 -19.262 1.00 0.00

ATOM 76 C ASP 10 -10.322 14.127 -18.551 1.00 0.00

ATOM 77 O ASP 10 -9.834 13.034 -18.264 1.00 0.00

ATOM 78 CB ASP 10 -8.308 14.540 -19.979 1.00 0.00

ATOM 79 CG ASP 10 -8.697 13.573 -21.089 1.00 0.00

ATOM 80 OD1 ASP 10 -9.864 13.475 -21.383 1.00 0.00

ATOM 81 OD2 ASP 10 -7.816 13.060 -21.738 1.00 0.00

ATOM 82 N LYS 11 -11.578 14.454 -18.267 1.00 0.00

ATOM 83 CA LYS 11 -12.484 13.520 -17.610 1.00 0.00

ATOM 84 C LYS 11 -12.238 12.091 -18.079 1.00 0.00

ATOM 85 O LYS 11 -11.899 11.217 -17.283 1.00 0.00

ATOM 86 CB LYS 11 -13.939 13.914 -17.868 1.00 0.00

ATOM 87 CG LYS 11 -14.963 13.043 -17.153 1.00 0.00

ATOM 88 CD LYS 11 -16.379 13.553 -17.380 1.00 0.00

ATOM 89 CE LYS 11 -17.409 12.623 -16.756 1.00 0.00

ATOM 90 NZ LYS 11 -18.791 13.163 -16.877 1.00 0.00

ATOM 91 N LYS 12 -12.412 11.862 -19.377 1.00 0.00

ATOM 92 CA LYS 12 -12.217 10.538 -19.953 1.00 0.00

ATOM 93 C LYS 12 -10.976 9.865 -19.380 1.00 0.00

ATOM 94 O LYS 12 -11.006 8.687 -19.024 1.00 0.00

ATOM 95 CB LYS 12 -12.112 10.627 -21.476 1.00 0.00

ATOM 96 CG LYS 12 -11.993 9.281 -22.178 1.00 0.00

ATOM 97 CD LYS 12 -11.966 9.445 -23.691 1.00 0.00

ATOM 98 CE LYS 12 -11.773 8.108 -24.391 1.00 0.00

ATOM 99 NZ LYS 12 -11.758 8.253 -25.872 1.00 0.00

ATOM 100 N LEU 13 -9.886 10.620 -19.296 1.00 0.00

ATOM 101 CA LEU 13 -8.632 10.097 -18.768 1.00 0.00

ATOM 102 C LEU 13 -8.768 9.715 -17.300 1.00 0.00

ATOM 103 O LEU 13 -8.219 8.707 -16.858 1.00 0.00

ATOM 104 CB LEU 13 -7.510 11.127 -18.950 1.00 0.00

ATOM 105 CG LEU 13 -6.134 10.693 -18.428 1.00 0.00

ATOM 106 CD1 LEU 13 -5.038 11.225 -19.342 1.00 0.00

ATOM 107 CD2 LEU 13 -5.946 11.202 -17.007 1.00 0.00

ATOM 108 N ILE 14 -9.503 10.528 -16.548 1.00 0.00

ATOM 109 CA ILE 14 -9.728 10.266 -15.131 1.00 0.00

ATOM 110 C ILE 14 -10.489 8.962 -14.925 1.00 0.00

ATOM 111 O ILE 14 -10.135 8.154 -14.068 1.00 0.00

ATOM 112 CB ILE 14 -10.504 11.415 -14.461 1.00 0.00

ATOM 113 CG1 ILE 14 -9.647 12.682 -14.415 1.00 0.00

ATOM 114 CG2 ILE 14 -10.943 11.014 -13.061 1.00 0.00

ATOM 115 CD1 ILE 14 -10.413 13.924 -14.020 1.00 0.00

ATOM 116 N GLU 15 -11.537 8.764 -15.718 1.00 0.00

ATOM 117 CA GLU 15 -12.354 7.560 -15.620 1.00 0.00

ATOM 118 C GLU 15 -11.538 6.314 -15.933 1.00 0.00

ATOM 119 O GLU 15 -11.576 5.332 -15.192 1.00 0.00

ATOM 120 CB GLU 15 -13.557 7.651 -16.562 1.00 0.00

ATOM 121 CG GLU 15 -14.613 8.661 -16.138 1.00 0.00

ATOM 122 CD GLU 15 -15.692 8.792 -17.176 1.00 0.00

ATOM 123 OE1 GLU 15 -15.573 8.180 -18.210 1.00 0.00

ATOM 124 OE2 GLU 15 -16.689 9.414 -16.893 1.00 0.00

ATOM 125 N GLU 16 -10.797 6.359 -17.036 1.00 0.00

ATOM 126 CA GLU 16 -9.975 5.230 -17.455 1.00 0.00

ATOM 127 C GLU 16 -8.889 4.929 -16.430 1.00 0.00

ATOM 128 O GLU 16 -8.604 3.768 -16.135 1.00 0.00

ATOM 129 CB GLU 16 -9.347 5.502 -18.823 1.00 0.00

ATOM 130 CG GLU 16 -10.332 5.481 -19.983 1.00 0.00

ATOM 131 CD GLU 16 -11.029 4.153 -20.083 1.00 0.00

ATOM 132 OE1 GLU 16 -10.356 3.151 -20.119 1.00 0.00

ATOM 133 OE2 GLU 16 -12.235 4.132 -20.010 1.00 0.00

ATOM 134 N ALA 17 -8.286 5.982 -15.889 1.00 0.00

ATOM 135 CA ALA 17 -7.263 5.836 -14.861 1.00 0.00

ATOM 136 C ALA 17 -7.821 5.141 -13.624 1.00 0.00

ATOM 137 O ALA 17 -7.178 4.261 -13.052 1.00 0.00

ATOM 138 CB ALA 17 -6.682 7.193 -14.494 1.00 0.00

ATOM 139 N ARG 18 -9.020 5.543 -13.218 1.00 0.00

ATOM 140 CA ARG 18 -9.688 4.927 -12.077 1.00 0.00

ATOM 141 C ARG 18 -9.950 3.448 -12.325 1.00 0.00

ATOM 142 O ARG 18 -9.746 2.615 -11.441 1.00 0.00

ATOM 143 CB ARG 18 -10.964 5.662 -11.692 1.00 0.00

ATOM 144 CG ARG 18 -11.716 5.063 -10.515 1.00 0.00

ATOM 145 CD ARG 18 -10.964 5.081 -9.234 1.00 0.00

ATOM 146 NE ARG 18 -11.707 4.572 -8.092 1.00 0.00

ATOM 147 CZ ARG 18 -11.168 4.307 -6.886 1.00 0.00

ATOM 148 NH1 ARG 18 -9.881 4.464 -6.669 1.00 0.00

ATOM 149 NH2 ARG 18 -11.965 3.861 -5.930 1.00 0.00

ATOM 150 N LYS 19 -10.403 3.126 -13.532 1.00 0.00

ATOM 151 CA LYS 19 -10.651 1.741 -13.913 1.00 0.00

ATOM 152 C LYS 19 -9.396 0.892 -13.751 1.00 0.00

ATOM 153 O LYS 19 -9.443 -0.201 -13.187 1.00 0.00

ATOM 154 CB LYS 19 -11.155 1.665 -15.355 1.00 0.00

ATOM 155 CG LYS 19 -12.564 2.206 -15.558 1.00 0.00

ATOM 156 CD LYS 19 -12.997 2.090 -17.012 1.00 0.00

ATOM 157 CE LYS 19 -14.400 2.641 -17.218 1.00 0.00

ATOM 158 NZ LYS 19 -14.822 2.571 -18.644 1.00 0.00

ATOM 159 N MET 20 -8.275 1.402 -14.248 1.00 0.00

ATOM 160 CA MET 20 -6.999 0.705 -14.133 1.00 0.00

ATOM 161 C MET 20 -6.575 0.568 -12.677 1.00 0.00

ATOM 162 O MET 20 -6.084 -0.480 -12.258 1.00 0.00

ATOM 163 CB MET 20 -5.922 1.440 -14.930 1.00 0.00

ATOM 164 CG MET 20 -4.601 0.692 -15.042 1.00 0.00

ATOM 165 SD MET 20 -4.690 -0.712 -16.171 1.00 0.00

ATOM 166 CE MET 20 -4.801 -2.072 -15.011 1.00 0.00

ATOM 167 N ALA 21 -6.767 1.635 -11.908 1.00 0.00

ATOM 168 CA ALA 21 -6.437 1.625 -10.488 1.00 0.00

ATOM 169 C ALA 21 -7.136 0.479 -9.768 1.00 0.00

ATOM 170 O ALA 21 -6.524 -0.234 -8.973 1.00 0.00

ATOM 171 CB ALA 21 -6.802 2.957 -9.849 1.00 0.00

ATOM 172 N GLU 22 -8.423 0.308 -10.051 1.00 0.00

ATOM 173 CA GLU 22 -9.197 -0.782 -9.467 1.00 0.00

ATOM 174 C GLU 22 -8.649 -2.137 -9.894 1.00 0.00

ATOM 175 O GLU 22 -8.415 -3.013 -9.061 1.00 0.00

ATOM 176 CB GLU 22 -10.671 -0.663 -9.860 1.00 0.00

ATOM 177 CG GLU 22 -11.406 0.497 -9.203 1.00 0.00

ATOM 178 CD GLU 22 -12.803 0.634 -9.741 1.00 0.00

ATOM 179 OE1 GLU 22 -13.147 -0.096 -10.640 1.00 0.00

ATOM 180 OE2 GLU 22 -13.563 1.388 -9.180 1.00 0.00

ATOM 181 N LYS 23 -8.445 -2.304 -11.196 1.00 0.00

ATOM 182 CA LYS 23 -7.913 -3.550 -11.734 1.00 0.00

ATOM 183 C LYS 23 -6.488 -3.793 -11.257 1.00 0.00

ATOM 184 O LYS 23 -6.003 -4.925 -11.266 1.00 0.00

ATOM 185 CB LYS 23 -7.961 -3.537 -13.263 1.00 0.00

ATOM 186 CG LYS 23 -9.365 -3.587 -13.850 1.00 0.00

ATOM 187 CD LYS 23 -9.332 -3.542 -15.370 1.00 0.00

ATOM 188 CE LYS 23 -10.733 -3.626 -15.958 1.00 0.00

ATOM 189 NZ LYS 23 -10.717 -3.560 -17.444 1.00 0.00

ATOM 190 N ALA 24 -5.819 -2.724 -10.838 1.00 0.00

ATOM 191 CA ALA 24 -4.443 -2.817 -10.365 1.00 0.00

ATOM 192 C ALA 24 -4.392 -3.016 -8.856 1.00 0.00

ATOM 193 O ALA 24 -3.316 -3.155 -8.274 1.00 0.00

ATOM 194 CB ALA 24 -3.660 -1.576 -10.766 1.00 0.00

ATOM 195 N ASN 25 -5.562 -3.031 -8.227 1.00 0.00

ATOM 196 CA ASN 25 -5.655 -3.244 -6.787 1.00 0.00

ATOM 197 C ASN 25 -4.932 -2.144 -6.020 1.00 0.00

ATOM 198 O ASN 25 -4.168 -2.419 -5.095 1.00 0.00

ATOM 199 CB ASN 25 -5.112 -4.605 -6.390 1.00 0.00

ATOM 200 CG ASN 25 -5.544 -5.053 -5.022 1.00 0.00

ATOM 201 OD1 ASN 25 -6.605 -4.659 -4.523 1.00 0.00

ATOM 202 ND2 ASN 25 -4.691 -5.809 -4.380 1.00 0.00

ATOM 203 N LEU 26 -5.176 -0.898 -6.410 1.00 0.00

ATOM 204 CA LEU 26 -4.590 0.248 -5.726 1.00 0.00

ATOM 205 C LEU 26 -5.586 1.398 -5.625 1.00 0.00

ATOM 206 O LEU 26 -6.641 1.374 -6.259 1.00 0.00

ATOM 207 CB LEU 26 -3.319 0.706 -6.452 1.00 0.00

ATOM 208 CG LEU 26 -3.501 1.048 -7.936 1.00 0.00

ATOM 209 CD1 LEU 26 -4.054 2.460 -8.081 1.00 0.00

ATOM 210 CD2 LEU 26 -2.168 0.914 -8.657 1.00 0.00

ATOM 211 N GLU 27 -5.243 2.402 -4.826 1.00 0.00

ATOM 212 CA GLU 27 -6.131 3.536 -4.596 1.00 0.00

ATOM 213 C GLU 27 -5.786 4.700 -5.517 1.00 0.00

ATOM 214 O GLU 27 -4.614 5.009 -5.732 1.00 0.00

ATOM 215 CB GLU 27 -6.061 3.983 -3.134 1.00 0.00

ATOM 216 CG GLU 27 -6.647 2.986 -2.144 1.00 0.00

ATOM 217 CD GLU 27 -8.118 2.785 -2.377 1.00 0.00

ATOM 218 OE1 GLU 27 -8.836 3.755 -2.393 1.00 0.00

ATOM 219 OE2 GLU 27 -8.509 1.674 -2.651 1.00 0.00

ATOM 220 N LEU 28 -6.814 5.344 -6.058 1.00 0.00

ATOM 221 CA LEU 28 -6.622 6.466 -6.969 1.00 0.00

ATOM 222 C LEU 28 -7.483 7.657 -6.567 1.00 0.00

ATOM 223 O LEU 28 -8.672 7.507 -6.283 1.00 0.00

ATOM 224 CB LEU 28 -6.939 6.041 -8.408 1.00 0.00

ATOM 225 CG LEU 28 -6.673 7.108 -9.477 1.00 0.00

ATOM 226 CD1 LEU 28 -6.049 6.468 -10.710 1.00 0.00

ATOM 227 CD2 LEU 28 -7.978 7.806 -9.834 1.00 0.00

ATOM 228 N ARG 29 -6.877 8.838 -6.544 1.00 0.00

ATOM 229 CA ARG 29 -7.590 10.058 -6.185 1.00 0.00

ATOM 230 C ARG 29 -7.366 11.152 -7.221 1.00 0.00

ATOM 231 O ARG 29 -6.337 11.179 -7.896 1.00 0.00

ATOM 232 CB ARG 29 -7.238 10.538 -4.785 1.00 0.00

ATOM 233 CG ARG 29 -7.453 9.511 -3.684 1.00 0.00

ATOM 234 CD ARG 29 -8.878 9.193 -3.414 1.00 0.00

ATOM 235 NE ARG 29 -9.091 8.273 -2.308 1.00 0.00

ATOM 236 CZ ARG 29 -9.110 6.930 -2.422 1.00 0.00

ATOM 237 NH1 ARG 29 -8.968 6.348 -3.592 1.00 0.00

ATOM 238 NH2 ARG 29 -9.300 6.214 -1.328 1.00 0.00

ATOM 239 N THR 30 -8.335 12.053 -7.342 1.00 0.00

ATOM 240 CA THR 30 -8.244 13.154 -8.294 1.00 0.00

ATOM 241 C THR 30 -7.911 14.464 -7.591 1.00 0.00

ATOM 242 O THR 30 -8.531 14.817 -6.589 1.00 0.00

ATOM 243 CB THR 30 -9.554 13.327 -9.085 1.00 0.00

ATOM 244 OG1 THR 30 -9.828 12.132 -9.828 1.00 0.00

ATOM 245 CG2 THR 30 -9.446 14.503 -10.044 1.00 0.00

ATOM 246 N VAL 31 -6.928 15.182 -8.124 1.00 0.00

ATOM 247 CA VAL 31 -6.502 16.449 -7.542 1.00 0.00

ATOM 248 C VAL 31 -7.308 17.612 -8.105 1.00 0.00

ATOM 249 O VAL 31 -7.322 17.841 -9.315 1.00 0.00

ATOM 250 CB VAL 31 -5.004 16.708 -7.786 1.00 0.00

ATOM 251 CG1 VAL 31 -4.599 18.062 -7.222 1.00 0.00

ATOM 252 CG2 VAL 31 -4.163 15.602 -7.168 1.00 0.00

ATOM 253 N LYS 32 -7.977 18.345 -7.221 1.00 0.00

ATOM 254 CA LYS 32 -8.754 19.511 -7.624 1.00 0.00

ATOM 255 C LYS 32 -7.886 20.762 -7.669 1.00 0.00

ATOM 256 O LYS 32 -8.151 21.685 -8.439 1.00 0.00

ATOM 257 CB LYS 32 -9.934 19.723 -6.674 1.00 0.00

ATOM 258 CG LYS 32 -10.986 18.624 -6.724 1.00 0.00

ATOM 259 CD LYS 32 -12.142 18.921 -5.780 1.00 0.00

ATOM 260 CE LYS 32 -13.189 17.818 -5.821 1.00 0.00

ATOM 261 NZ LYS 32 -14.321 18.091 -4.893 1.00 0.00

ATOM 262 N THR 33 -6.848 20.786 -6.840 1.00 0.00

ATOM 263 CA THR 33 -5.935 21.922 -6.788 1.00 0.00

ATOM 264 C THR 33 -4.649 21.561 -6.055 1.00 0.00

ATOM 265 O THR 33 -4.577 20.540 -5.372 1.00 0.00

ATOM 266 CB THR 33 -6.584 23.136 -6.099 1.00 0.00

ATOM 267 OG1 THR 33 -5.722 24.275 -6.221 1.00 0.00

ATOM 268 CG2 THR 33 -6.828 22.846 -4.626 1.00 0.00

ATOM 269 N GLU 34 -3.634 22.406 -6.203 1.00 0.00

ATOM 270 CA GLU 34 -2.344 22.173 -5.564 1.00 0.00

ATOM 271 C GLU 34 -2.513 21.826 -4.091 1.00 0.00

ATOM 272 O GLU 34 -1.789 20.988 -3.553 1.00 0.00

ATOM 273 CB GLU 34 -1.442 23.400 -5.714 1.00 0.00

ATOM 274 CG GLU 34 -0.040 23.222 -5.148 1.00 0.00

ATOM 275 CD GLU 34 0.807 24.439 -5.396 1.00 0.00

ATOM 276 OE1 GLU 34 0.316 25.372 -5.985 1.00 0.00

ATOM 277 OE2 GLU 34 1.908 24.483 -4.900 1.00 0.00

ATOM 278 N ASP 35 -3.474 22.474 -3.442 1.00 0.00

ATOM 279 CA ASP 35 -3.763 22.212 -2.036 1.00 0.00

ATOM 280 C ASP 35 -4.183 20.764 -1.821 1.00 0.00

ATOM 281 O ASP 35 -3.812 20.141 -0.826 1.00 0.00

ATOM 282 CB ASP 35 -4.854 23.157 -1.527 1.00 0.00

ATOM 283 CG ASP 35 -4.400 24.598 -1.336 1.00 0.00

ATOM 284 OD1 ASP 35 -3.216 24.836 -1.367 1.00 0.00

ATOM 285 OD2 ASP 35 -5.239 25.466 -1.319 1.00 0.00

ATOM 286 N GLU 36 -4.958 20.233 -2.761 1.00 0.00

ATOM 287 CA GLU 36 -5.426 18.855 -2.678 1.00 0.00

ATOM 288 C GLU 36 -4.282 17.870 -2.885 1.00 0.00

ATOM 289 O GLU 36 -4.193 16.853 -2.197 1.00 0.00

ATOM 290 CB GLU 36 -6.531 18.601 -3.706 1.00 0.00

ATOM 291 CG GLU 36 -7.850 19.294 -3.396 1.00 0.00

ATOM 292 CD GLU 36 -8.389 18.868 -2.059 1.00 0.00

ATOM 293 OE1 GLU 36 -8.503 17.687 -1.835 1.00 0.00

ATOM 294 OE2 GLU 36 -8.583 19.717 -1.221 1.00 0.00

ATOM 295 N LEU 37 -3.408 18.179 -3.837 1.00 0.00

ATOM 296 CA LEU 37 -2.233 17.355 -4.095 1.00 0.00

ATOM 297 C LEU 37 -1.342 17.263 -2.862 1.00 0.00

ATOM 298 O LEU 37 -0.882 16.183 -2.496 1.00 0.00

ATOM 299 CB LEU 37 -1.443 17.917 -5.284 1.00 0.00

ATOM 300 CG LEU 37 -0.071 17.272 -5.519 1.00 0.00

ATOM 301 CD1 LEU 37 -0.218 15.761 -5.633 1.00 0.00

ATOM 302 CD2 LEU 37 0.553 17.850 -6.781 1.00 0.00

ATOM 303 N LYS 38 -1.104 18.406 -2.226 1.00 0.00

ATOM 304 CA LYS 38 -0.275 18.456 -1.026 1.00 0.00

ATOM 305 C LYS 38 -0.904 17.660 0.110 1.00 0.00

ATOM 306 O LYS 38 -0.226 16.892 0.792 1.00 0.00

ATOM 307 CB LYS 38 -0.049 19.904 -0.591 1.00 0.00

ATOM 308 CG LYS 38 0.883 20.695 -1.499 1.00 0.00

ATOM 309 CD LYS 38 1.160 22.082 -0.936 1.00 0.00

ATOM 310 CE LYS 38 -0.039 23.001 -1.115 1.00 0.00

ATOM 311 NZ LYS 38 0.237 24.377 -0.620 1.00 0.00

ATOM 312 N LYS 39 -2.204 17.849 0.310 1.00 0.00

ATOM 313 CA LYS 39 -2.934 17.123 1.342 1.00 0.00

ATOM 314 C LYS 39 -2.836 15.617 1.130 1.00 0.00

ATOM 315 O LYS 39 -2.621 14.860 2.077 1.00 0.00

ATOM 316 CB LYS 39 -4.401 17.556 1.365 1.00 0.00

ATOM 317 CG LYS 39 -5.239 16.881 2.443 1.00 0.00

ATOM 318 CD LYS 39 -6.663 17.417 2.452 1.00 0.00

ATOM 319 CE LYS 39 -7.512 16.717 3.504 1.00 0.00

ATOM 320 NZ LYS 39 -8.912 17.221 3.515 1.00 0.00

ATOM 321 N TYR 40 -2.994 15.188 -0.117 1.00 0.00

ATOM 322 CA TYR 40 -2.912 13.773 -0.458 1.00 0.00

ATOM 323 C TYR 40 -1.557 13.192 -0.072 1.00 0.00

ATOM 324 O TYR 40 -1.481 12.161 0.596 1.00 0.00

ATOM 325 CB TYR 40 -3.165 13.566 -1.953 1.00 0.00

ATOM 326 CG TYR 40 -4.592 13.835 -2.376 1.00 0.00

ATOM 327 CD1 TYR 40 -5.608 13.929 -1.436 1.00 0.00

ATOM 328 CD2 TYR 40 -4.918 13.996 -3.714 1.00 0.00

ATOM 329 CE1 TYR 40 -6.913 14.174 -1.817 1.00 0.00

ATOM 330 CE2 TYR 40 -6.220 14.242 -4.107 1.00 0.00

ATOM 331 CZ TYR 40 -7.215 14.331 -3.155 1.00 0.00

ATOM 332 OH TYR 40 -8.513 14.576 -3.541 1.00 0.00

ATOM 333 N LEU 41 -0.490 13.859 -0.497 1.00 0.00

ATOM 334 CA LEU 41 0.863 13.390 -0.231 1.00 0.00

ATOM 335 C LEU 41 1.145 13.341 1.266 1.00 0.00

ATOM 336 O LEU 41 1.765 12.401 1.761 1.00 0.00

ATOM 337 CB LEU 41 1.885 14.289 -0.937 1.00 0.00

ATOM 338 CG LEU 41 1.899 14.184 -2.468 1.00 0.00

ATOM 339 CD1 LEU 41 2.816 15.248 -3.056 1.00 0.00

ATOM 340 CD2 LEU 41 2.356 12.792 -2.878 1.00 0.00

ATOM 341 N GLU 42 0.684 14.361 1.982 1.00 0.00

ATOM 342 CA GLU 42 0.880 14.435 3.425 1.00 0.00

ATOM 343 C GLU 42 0.251 13.240 4.129 1.00 0.00

ATOM 344 O GLU 42 0.882 12.599 4.969 1.00 0.00

ATOM 345 CB GLU 42 0.298 15.737 3.979 1.00 0.00

ATOM 346 CG GLU 42 1.079 16.987 3.598 1.00 0.00

ATOM 347 CD GLU 42 0.377 18.233 4.063 1.00 0.00

ATOM 348 OE1 GLU 42 -0.706 18.122 4.585 1.00 0.00

ATOM 349 OE2 GLU 42 0.970 19.284 3.999 1.00 0.00

ATOM 350 N GLU 43 -0.997 12.945 3.780 1.00 0.00

ATOM 351 CA GLU 43 -1.711 11.819 4.369 1.00 0.00

ATOM 352 C GLU 43 -1.028 10.499 4.036 1.00 0.00

ATOM 353 O GLU 43 -0.921 9.612 4.884 1.00 0.00

ATOM 354 CB GLU 43 -3.164 11.795 3.889 1.00 0.00

ATOM 355 CG GLU 43 -4.030 12.915 4.448 1.00 0.00

ATOM 356 CD GLU 43 -5.403 12.900 3.838 1.00 0.00

ATOM 357 OE1 GLU 43 -5.637 12.097 2.966 1.00 0.00

ATOM 358 OE2 GLU 43 -6.253 13.613 4.318 1.00 0.00

ATOM 359 N PHE 44 -0.566 10.374 2.797 1.00 0.00

ATOM 360 CA PHE 44 0.124 9.168 2.354 1.00 0.00

ATOM 361 C PHE 44 1.373 8.910 3.186 1.00 0.00

ATOM 362 O PHE 44 1.641 7.777 3.586 1.00 0.00

ATOM 363 CB PHE 44 0.491 9.275 0.873 1.00 0.00

ATOM 364 CG PHE 44 -0.693 9.457 -0.034 1.00 0.00

ATOM 365 CD1 PHE 44 -1.979 9.214 0.424 1.00 0.00

ATOM 366 CD2 PHE 44 -0.523 9.871 -1.346 1.00 0.00

ATOM 367 CE1 PHE 44 -3.069 9.381 -0.409 1.00 0.00

ATOM 368 CE2 PHE 44 -1.610 10.040 -2.181 1.00 0.00

ATOM 369 CZ PHE 44 -2.885 9.794 -1.712 1.00 0.00

ATOM 370 N ARG 45 2.135 9.967 3.445 1.00 0.00

ATOM 371 CA ARG 45 3.330 9.867 4.273 1.00 0.00

ATOM 372 C ARG 45 2.981 9.460 5.698 1.00 0.00

ATOM 373 O ARG 45 3.664 8.634 6.303 1.00 0.00

ATOM 374 CB ARG 45 4.156 11.145 4.245 1.00 0.00

ATOM 375 CG ARG 45 5.430 11.101 5.074 1.00 0.00

ATOM 376 CD ARG 45 6.264 12.326 4.979 1.00 0.00

ATOM 377 NE ARG 45 7.507 12.268 5.731 1.00 0.00

ATOM 378 CZ ARG 45 7.621 12.546 7.045 1.00 0.00

ATOM 379 NH1 ARG 45 6.580 12.937 7.747 1.00 0.00

ATOM 380 NH2 ARG 45 8.813 12.438 7.605 1.00 0.00

ATOM 381 N LYS 46 1.914 10.046 6.231 1.00 0.00

ATOM 382 CA LYS 46 1.434 9.699 7.562 1.00 0.00

ATOM 383 C LYS 46 1.131 8.210 7.669 1.00 0.00

ATOM 384 O LYS 46 1.435 7.576 8.679 1.00 0.00

ATOM 385 CB LYS 46 0.189 10.516 7.912 1.00 0.00

ATOM 386 CG LYS 46 0.456 11.995 8.159 1.00 0.00

ATOM 387 CD LYS 46 -0.825 12.737 8.509 1.00 0.00

ATOM 388 CE LYS 46 -0.562 14.219 8.738 1.00 0.00

ATOM 389 NZ LYS 46 -1.813 14.965 9.044 1.00 0.00

ATOM 390 N GLU 47 0.529 7.657 6.621 1.00 0.00

ATOM 391 CA GLU 47 0.249 6.228 6.563 1.00 0.00

ATOM 392 C GLU 47 1.538 5.416 6.529 1.00 0.00

ATOM 393 O GLU 47 1.681 4.431 7.254 1.00 0.00

ATOM 394 CB GLU 47 -0.612 5.899 5.342 1.00 0.00

ATOM 395 CG GLU 47 -2.047 6.399 5.432 1.00 0.00

ATOM 396 CD GLU 47 -2.791 6.159 4.148 1.00 0.00

ATOM 397 OE1 GLU 47 -2.187 5.693 3.212 1.00 0.00

ATOM 398 OE2 GLU 47 -3.986 6.335 4.135 1.00 0.00

ATOM 399 N SER 48 2.473 5.833 5.683 1.00 0.00

ATOM 400 CA SER 48 3.755 5.151 5.560 1.00 0.00

ATOM 401 C SER 48 4.786 6.035 4.869 1.00 0.00

ATOM 402 O SER 48 4.474 6.724 3.898 1.00 0.00

ATOM 403 CB SER 48 3.583 3.849 4.802 1.00 0.00

ATOM 404 OG SER 48 4.802 3.184 4.612 1.00 0.00

ATOM 405 N GLN 49 6.014 6.009 5.374 1.00 0.00

ATOM 406 CA GLN 49 7.092 6.812 4.809 1.00 0.00

ATOM 407 C GLN 49 7.678 6.151 3.568 1.00 0.00

ATOM 408 O GLN 49 8.283 6.816 2.726 1.00 0.00

ATOM 409 CB GLN 49 8.196 7.033 5.846 1.00 0.00

ATOM 410 CG GLN 49 7.776 7.889 7.029 1.00 0.00

ATOM 411 CD GLN 49 8.884 8.044 8.053 1.00 0.00

ATOM 412 OE1 GLN 49 9.992 7.531 7.873 1.00 0.00

ATOM 413 NE2 GLN 49 8.590 8.751 9.139 1.00 0.00

ATOM 414 N ASN 50 7.495 4.840 3.458 1.00 0.00

ATOM 415 CA ASN 50 8.005 4.086 2.320 1.00 0.00

ATOM 416 C ASN 50 6.901 3.799 1.310 1.00 0.00

ATOM 417 O ASN 50 7.093 3.030 0.368 1.00 0.00

ATOM 418 CB ASN 50 8.663 2.792 2.761 1.00 0.00

ATOM 419 CG ASN 50 9.920 2.993 3.561 1.00 0.00

ATOM 420 OD1 ASN 50 10.922 3.516 3.059 1.00 0.00

ATOM 421 ND2 ASN 50 9.900 2.509 4.776 1.00 0.00

ATOM 422 N ILE 51 5.826 4.564 1.461 1.00 0.00

ATOM 423 CA ILE 51 4.653 4.448 0.603 1.00 0.00

ATOM 424 C ILE 51 4.970 4.879 -0.823 1.00 0.00

ATOM 425 O ILE 51 5.812 5.749 -1.047 1.00 0.00

ATOM 426 CB ILE 51 3.478 5.290 1.134 1.00 0.00

ATOM 427 CG1 ILE 51 2.154 4.784 0.555 1.00 0.00

ATOM 428 CG2 ILE 51 3.683 6.759 0.800 1.00 0.00

ATOM 429 CD1 ILE 51 0.932 5.358 1.234 1.00 0.00

ATOM 430 N LYS 52 4.292 4.264 -1.787 1.00 0.00

ATOM 431 CA LYS 52 4.500 4.582 -3.194 1.00 0.00

ATOM 432 C LYS 52 3.335 5.387 -3.754 1.00 0.00

ATOM 433 O LYS 52 2.174 5.005 -3.604 1.00 0.00

ATOM 434 CB LYS 52 4.696 3.304 -4.010 1.00 0.00

ATOM 435 CG LYS 52 5.960 2.526 -3.667 1.00 0.00

ATOM 436 CD LYS 52 6.135 1.322 -4.581 1.00 0.00

ATOM 437 CE LYS 52 7.436 0.590 -4.290 1.00 0.00

ATOM 438 NZ LYS 52 8.626 1.392 -4.683 1.00 0.00

ATOM 439 N VAL 53 3.650 6.503 -4.402 1.00 0.00

ATOM 440 CA VAL 53 2.627 7.387 -4.950 1.00 0.00

ATOM 441 C VAL 53 2.979 7.825 -6.366 1.00 0.00

ATOM 442 O VAL 53 4.023 8.437 -6.594 1.00 0.00

ATOM 443 CB VAL 53 2.428 8.636 -4.071 1.00 0.00

ATOM 444 CG1 VAL 53 1.269 9.473 -4.590 1.00 0.00

ATOM 445 CG2 VAL 53 2.188 8.235 -2.623 1.00 0.00

ATOM 446 N LEU 54 2.102 7.512 -7.313 1.00 0.00

ATOM 447 CA LEU 54 2.299 7.908 -8.702 1.00 0.00

ATOM 448 C LEU 54 1.385 9.067 -9.079 1.00 0.00

ATOM 449 O LEU 54 0.165 8.979 -8.937 1.00 0.00

ATOM 450 CB LEU 54 2.058 6.713 -9.633 1.00 0.00

ATOM 451 CG LEU 54 1.977 7.055 -11.126 1.00 0.00

ATOM 452 CD1 LEU 54 3.359 7.409 -11.657 1.00 0.00

ATOM 453 CD2 LEU 54 1.393 5.874 -11.887 1.00 0.00

ATOM 454 N ILE 55 1.982 10.153 -9.560 1.00 0.00

ATOM 455 CA ILE 55 1.223 11.333 -9.955 1.00 0.00

ATOM 456 C ILE 55 1.251 11.524 -11.466 1.00 0.00

ATOM 457 O ILE 55 2.306 11.771 -12.050 1.00 0.00

ATOM 458 CB ILE 55 1.761 12.605 -9.276 1.00 0.00

ATOM 459 CG1 ILE 55 1.791 12.427 -7.756 1.00 0.00

ATOM 460 CG2 ILE 55 0.914 13.810 -9.657 1.00 0.00

ATOM 461 CD1 ILE 55 2.497 13.545 -7.024 1.00 0.00

ATOM 462 N LEU 56 0.086 11.410 -12.095 1.00 0.00

ATOM 463 CA LEU 56 -0.025 11.571 -13.539 1.00 0.00

ATOM 464 C LEU 56 -0.681 12.898 -13.897 1.00 0.00

ATOM 465 O LEU 56 -1.847 13.131 -13.578 1.00 0.00

ATOM 466 CB LEU 56 -0.817 10.405 -14.143 1.00 0.00

ATOM 467 CG LEU 56 -0.209 9.015 -13.914 1.00 0.00

ATOM 468 CD1 LEU 56 -1.118 7.944 -14.502 1.00 0.00

ATOM 469 CD2 LEU 56 1.174 8.954 -14.544 1.00 0.00

ATOM 470 N VAL 57 0.075 13.767 -14.559 1.00 0.00

ATOM 471 CA VAL 57 -0.400 15.107 -14.881 1.00 0.00

ATOM 472 C VAL 57 -0.418 15.339 -16.387 1.00 0.00

ATOM 473 O VAL 57 0.462 14.868 -17.108 1.00 0.00

ATOM 474 CB VAL 57 0.470 16.190 -14.216 1.00 0.00

ATOM 475 CG1 VAL 57 -0.114 17.572 -14.469 1.00 0.00

ATOM 476 CG2 VAL 57 0.594 15.932 -12.722 1.00 0.00

ATOM 477 N SER 58 -1.426 16.066 -16.855 1.00 0.00

ATOM 478 CA SER 58 -1.539 16.398 -18.270 1.00 0.00

ATOM 479 C SER 58 -0.984 17.788 -18.556 1.00 0.00

ATOM 480 O SER 58 -0.408 18.031 -19.617 1.00 0.00

ATOM 481 CB SER 58 -2.985 16.305 -18.714 1.00 0.00

ATOM 482 OG SER 58 -3.416 14.977 -18.830 1.00 0.00

ATOM 483 N ASN 59 -1.161 18.697 -17.603 1.00 0.00

ATOM 484 CA ASN 59 -0.690 20.068 -17.757 1.00 0.00

ATOM 485 C ASN 59 0.777 20.194 -17.367 1.00 0.00

ATOM 486 O ASN 59 1.221 19.599 -16.385 1.00 0.00

ATOM 487 CB ASN 59 -1.533 21.038 -16.949 1.00 0.00

ATOM 488 CG ASN 59 -2.928 21.215 -17.480 1.00 0.00

ATOM 489 OD1 ASN 59 -3.195 20.994 -18.667 1.00 0.00

ATOM 490 ND2 ASN 59 -3.801 21.689 -16.628 1.00 0.00

ATOM 491 N ASP 60 1.526 20.970 -18.142 1.00 0.00

ATOM 492 CA ASP 60 2.950 21.157 -17.892 1.00 0.00

ATOM 493 C ASP 60 3.187 21.887 -16.575 1.00 0.00

ATOM 494 O ASP 60 4.045 21.496 -15.785 1.00 0.00

ATOM 495 CB ASP 60 3.601 21.926 -19.044 1.00 0.00

ATOM 496 CG ASP 60 3.757 21.122 -20.328 1.00 0.00

ATOM 497 OD1 ASP 60 3.593 19.926 -20.281 1.00 0.00

ATOM 498 OD2 ASP 60 3.883 21.722 -21.369 1.00 0.00

ATOM 499 N GLU 61 2.422 22.949 -16.347 1.00 0.00

ATOM 500 CA GLU 61 2.541 23.730 -15.123 1.00 0.00

ATOM 501 C GLU 61 2.279 22.870 -13.893 1.00 0.00

ATOM 502 O GLU 61 3.078 22.846 -12.957 1.00 0.00

ATOM 503 CB GLU 61 1.577 24.918 -15.150 1.00 0.00

ATOM 504 CG GLU 61 1.658 25.822 -13.928 1.00 0.00

ATOM 505 CD GLU 61 0.698 26.974 -14.036 1.00 0.00

ATOM 506 OE1 GLU 61 0.016 27.063 -15.028 1.00 0.00

ATOM 507 OE2 GLU 61 0.564 27.701 -13.079 1.00 0.00

ATOM 508 N GLU 62 1.152 22.166 -13.901 1.00 0.00

ATOM 509 CA GLU 62 0.792 21.286 -12.795 1.00 0.00

ATOM 510 C GLU 62 1.809 20.164 -12.630 1.00 0.00

ATOM 511 O GLU 62 2.101 19.737 -11.512 1.00 0.00

ATOM 512 CB GLU 62 -0.606 20.700 -13.010 1.00 0.00

ATOM 513 CG GLU 62 -1.738 21.707 -12.867 1.00 0.00

ATOM 514 CD GLU 62 -1.737 22.341 -11.504 1.00 0.00

ATOM 515 OE1 GLU 62 -1.723 21.621 -10.535 1.00 0.00

ATOM 516 OE2 GLU 62 -1.636 23.543 -11.430 1.00 0.00

ATOM 517 N LEU 63 2.347 19.690 -13.748 1.00 0.00

ATOM 518 CA LEU 63 3.392 18.673 -13.725 1.00 0.00

ATOM 519 C LEU 63 4.599 19.141 -12.922 1.00 0.00

ATOM 520 O LEU 63 5.102 18.419 -12.061 1.00 0.00

ATOM 521 CB LEU 63 3.811 18.312 -15.156 1.00 0.00

ATOM 522 CG LEU 63 4.756 17.109 -15.276 1.00 0.00

ATOM 523 CD1 LEU 63 4.013 15.826 -14.928 1.00 0.00

ATOM 524 CD2 LEU 63 5.317 17.042 -16.688 1.00 0.00

ATOM 525 N ASP 64 5.060 20.354 -13.209 1.00 0.00

ATOM 526 CA ASP 64 6.191 20.932 -12.493 1.00 0.00

ATOM 527 C ASP 64 5.891 21.069 -11.007 1.00 0.00

ATOM 528 O ASP 64 6.743 20.794 -10.162 1.00 0.00

ATOM 529 CB ASP 64 6.558 22.296 -13.083 1.00 0.00

ATOM 530 CG ASP 64 7.238 22.230 -14.444 1.00 0.00

ATOM 531 OD1 ASP 64 7.638 21.159 -14.835 1.00 0.00

ATOM 532 OD2 ASP 64 7.213 23.213 -15.146 1.00 0.00

ATOM 533 N LYS 65 4.673 21.497 -10.691 1.00 0.00

ATOM 534 CA LYS 65 4.242 21.627 -9.305 1.00 0.00

ATOM 535 C LYS 65 4.303 20.289 -8.580 1.00 0.00

ATOM 536 O LYS 65 4.770 20.207 -7.444 1.00 0.00

ATOM 537 CB LYS 65 2.824 22.197 -9.236 1.00 0.00

ATOM 538 CG LYS 65 2.715 23.662 -9.640 1.00 0.00

ATOM 539 CD LYS 65 1.279 24.156 -9.545 1.00 0.00

ATOM 540 CE LYS 65 1.165 25.612 -9.972 1.00 0.00

ATOM 541 NZ LYS 65 -0.239 26.100 -9.917 1.00 0.00

ATOM 542 N ALA 66 3.829 19.240 -9.245 1.00 0.00

ATOM 543 CA ALA 66 3.836 17.901 -8.668 1.00 0.00

ATOM 544 C ALA 66 5.259 17.416 -8.419 1.00 0.00

ATOM 545 O ALA 66 5.548 16.818 -7.383 1.00 0.00

ATOM 546 CB ALA 66 3.092 16.930 -9.574 1.00 0.00

ATOM 547 N LYS 67 6.143 17.676 -9.376 1.00 0.00

ATOM 548 CA LYS 67 7.544 17.290 -9.250 1.00 0.00

ATOM 549 C LYS 67 8.196 17.967 -8.052 1.00 0.00

ATOM 550 O LYS 67 8.927 17.334 -7.290 1.00 0.00

ATOM 551 CB LYS 67 8.311 17.631 -10.528 1.00 0.00

ATOM 552 CG LYS 67 7.950 16.766 -11.728 1.00 0.00

ATOM 553 CD LYS 67 8.752 17.163 -12.958 1.00 0.00

ATOM 554 CE LYS 67 8.373 16.316 -14.164 1.00 0.00

ATOM 555 NZ LYS 67 9.126 16.716 -15.384 1.00 0.00

ATOM 556 N GLU 68 7.930 19.259 -7.890 1.00 0.00

ATOM 557 CA GLU 68 8.457 20.015 -6.761 1.00 0.00

ATOM 558 C GLU 68 8.022 19.403 -5.436 1.00 0.00

ATOM 559 O GLU 68 8.844 19.169 -4.549 1.00 0.00

ATOM 560 CB GLU 68 8.008 21.476 -6.839 1.00 0.00

ATOM 561 CG GLU 68 8.546 22.359 -5.722 1.00 0.00

ATOM 562 CD GLU 68 8.092 23.783 -5.885 1.00 0.00

ATOM 563 OE1 GLU 68 7.404 24.062 -6.837 1.00 0.00

ATOM 564 OE2 GLU 68 8.337 24.568 -5.000 1.00 0.00

ATOM 565 N LEU 69 6.725 19.145 -5.306 1.00 0.00

ATOM 566 CA LEU 69 6.179 18.553 -4.091 1.00 0.00

ATOM 567 C LEU 69 6.739 17.156 -3.858 1.00 0.00

ATOM 568 O LEU 69 6.987 16.758 -2.721 1.00 0.00

ATOM 569 CB LEU 69 4.647 18.508 -4.165 1.00 0.00

ATOM 570 CG LEU 69 3.950 19.873 -4.105 1.00 0.00

ATOM 571 CD1 LEU 69 2.459 19.710 -4.363 1.00 0.00

ATOM 572 CD2 LEU 69 4.195 20.510 -2.745 1.00 0.00

ATOM 573 N ALA 70 6.937 16.416 -4.944 1.00 0.00

ATOM 574 CA ALA 70 7.493 15.070 -4.863 1.00 0.00

ATOM 575 C ALA 70 8.881 15.086 -4.235 1.00 0.00

ATOM 576 O ALA 70 9.197 14.255 -3.383 1.00 0.00

ATOM 577 CB ALA 70 7.538 14.431 -6.243 1.00 0.00

ATOM 578 N GLN 71 9.708 16.035 -4.661 1.00 0.00

ATOM 579 CA GLN 71 11.062 16.164 -4.137 1.00 0.00

ATOM 580 C GLN 71 11.050 16.423 -2.636 1.00 0.00

ATOM 581 O GLN 71 11.846 15.851 -1.892 1.00 0.00

ATOM 582 CB GLN 71 11.807 17.295 -4.850 1.00 0.00

ATOM 583 CG GLN 71 12.164 16.991 -6.296 1.00 0.00

ATOM 584 CD GLN 71 12.790 18.180 -6.999 1.00 0.00

ATOM 585 OE1 GLN 71 12.886 19.273 -6.435 1.00 0.00

ATOM 586 NE2 GLN 71 13.218 17.974 -8.240 1.00 0.00

ATOM 587 N LYS 72 10.045 17.397 -2.386 1.00 0.00

ATOM 588 CA LYS 72 9.920 17.767 -0.982 1.00 0.00

ATOM 589 C LYS 72 9.551 16.564 -0.124 1.00 0.00

ATOM 590 O LYS 72 10.084 16.382 0.971 1.00 0.00

ATOM 591 CB LYS 72 8.878 18.874 -0.811 1.00 0.00

ATOM 592 CG LYS 72 9.304 20.229 -1.358 1.00 0.00

ATOM 593 CD LYS 72 8.219 21.276 -1.151 1.00 0.00

ATOM 594 CE LYS 72 8.634 22.625 -1.721 1.00 0.00

ATOM 595 NZ LYS 72 7.569 23.650 -1.554 1.00 0.00

ATOM 596 N MET 73 8.635 15.743 -0.629 1.00 0.00

ATOM 597 CA MET 73 8.165 14.575 0.106 1.00 0.00

ATOM 598 C MET 73 9.283 13.559 0.301 1.00 0.00

ATOM 599 O MET 73 10.165 13.422 -0.547 1.00 0.00

ATOM 600 CB MET 73 6.988 13.931 -0.623 1.00 0.00

ATOM 601 CG MET 73 5.732 14.789 -0.673 1.00 0.00

ATOM 602 SD MET 73 5.252 15.414 0.950 1.00 0.00

ATOM 603 CE MET 73 4.721 13.903 1.751 1.00 0.00

ATOM 604 N GLU 74 9.243 12.850 1.424 1.00 0.00

ATOM 605 CA GLU 74 10.254 11.845 1.732 1.00 0.00

ATOM 606 C GLU 74 9.797 10.455 1.307 1.00 0.00

ATOM 607 O GLU 74 10.540 9.482 1.429 1.00 0.00

ATOM 608 CB GLU 74 10.582 11.856 3.227 1.00 0.00

ATOM 609 CG GLU 74 11.245 13.136 3.715 1.00 0.00

ATOM 610 CD GLU 74 11.516 13.082 5.193 1.00 0.00

ATOM 611 OE1 GLU 74 11.172 12.099 5.805 1.00 0.00

ATOM 612 OE2 GLU 74 12.166 13.970 5.691 1.00 0.00

ATOM 613 N ILE 75 8.569 10.370 0.806 1.00 0.00

ATOM 614 CA ILE 75 8.014 9.100 0.352 1.00 0.00

ATOM 615 C ILE 75 8.327 8.857 -1.119 1.00 0.00

ATOM 616 O ILE 75 8.829 9.742 -1.811 1.00 0.00

ATOM 617 CB ILE 75 6.489 9.044 0.559 1.00 0.00

ATOM 618 CG1 ILE 75 5.796 10.115 -0.286 1.00 0.00

ATOM 619 CG2 ILE 75 6.145 9.217 2.030 1.00 0.00

ATOM 620 CD1 ILE 75 4.287 10.082 -0.196 1.00 0.00

ATOM 621 N ASP 76 8.028 7.651 -1.591 1.00 0.00

ATOM 622 CA ASP 76 8.362 7.259 -2.956 1.00 0.00

ATOM 623 C ASP 76 7.368 7.839 -3.954 1.00 0.00

ATOM 624 O ASP 76 6.454 7.149 -4.406 1.00 0.00

ATOM 625 CB ASP 76 8.404 5.734 -3.081 1.00 0.00

ATOM 626 CG ASP 76 8.903 5.226 -4.427 1.00 0.00

ATOM 627 OD1 ASP 76 9.296 6.034 -5.235 1.00 0.00

ATOM 628 OD2 ASP 76 9.036 4.035 -4.577 1.00 0.00

ATOM 629 N VAL 77 7.553 9.110 -4.295 1.00 0.00

ATOM 630 CA VAL 77 6.691 9.775 -5.264 1.00 0.00

ATOM 631 C VAL 77 7.311 9.759 -6.656 1.00 0.00

ATOM 632 O VAL 77 8.490 10.070 -6.824 1.00 0.00

ATOM 633 CB VAL 77 6.404 11.233 -4.857 1.00 0.00

ATOM 634 CG1 VAL 77 5.441 11.880 -5.841 1.00 0.00

ATOM 635 CG2 VAL 77 5.840 11.291 -3.446 1.00 0.00

ATOM 636 N ARG 78 6.510 9.392 -7.650 1.00 0.00

ATOM 637 CA ARG 78 6.949 9.426 -9.040 1.00 0.00

ATOM 638 C ARG 78 5.953 10.178 -9.914 1.00 0.00

ATOM 639 O ARG 78 4.765 9.853 -9.937 1.00 0.00

ATOM 640 CB ARG 78 7.232 8.035 -9.587 1.00 0.00

ATOM 641 CG ARG 78 8.372 7.298 -8.903 1.00 0.00

ATOM 642 CD ARG 78 9.711 7.900 -9.126 1.00 0.00

ATOM 643 NE ARG 78 10.810 7.170 -8.514 1.00 0.00

ATOM 644 CZ ARG 78 11.234 7.345 -7.247 1.00 0.00

ATOM 645 NH1 ARG 78 10.681 8.247 -6.467 1.00 0.00

ATOM 646 NH2 ARG 78 12.237 6.600 -6.816 1.00 0.00

ATOM 647 N THR 79 6.443 11.182 -10.633 1.00 0.00

ATOM 648 CA THR 79 5.584 12.033 -11.446 1.00 0.00

ATOM 649 C THR 79 5.799 11.773 -12.932 1.00 0.00

ATOM 650 O THR 79 6.932 11.754 -13.411 1.00 0.00

ATOM 651 CB THR 79 5.830 13.525 -11.155 1.00 0.00

ATOM 652 OG1 THR 79 5.543 13.800 -9.778 1.00 0.00

ATOM 653 CG2 THR 79 4.946 14.393 -12.037 1.00 0.00

ATOM 654 N ARG 80 4.703 11.574 -13.656 1.00 0.00

ATOM 655 CA ARG 80 4.770 11.311 -15.089 1.00 0.00

ATOM 656 C ARG 80 3.729 12.123 -15.848 1.00 0.00

ATOM 657 O ARG 80 2.643 12.395 -15.334 1.00 0.00

ATOM 658 CB ARG 80 4.657 9.827 -15.406 1.00 0.00

ATOM 659 CG ARG 80 5.777 8.966 -14.844 1.00 0.00

ATOM 660 CD ARG 80 7.097 9.181 -15.490 1.00 0.00

ATOM 661 NE ARG 80 8.149 8.292 -15.022 1.00 0.00

ATOM 662 CZ ARG 80 8.938 8.534 -13.957 1.00 0.00

ATOM 663 NH1 ARG 80 8.826 9.650 -13.271 1.00 0.00

ATOM 664 NH2 ARG 80 9.847 7.630 -13.635 1.00 0.00

ATOM 665 N LYS 81 4.066 12.509 -17.074 1.00 0.00

ATOM 666 CA LYS 81 3.157 13.284 -17.910 1.00 0.00

ATOM 667 C LYS 81 2.299 12.375 -18.780 1.00 0.00

ATOM 668 O LYS 81 2.803 11.440 -19.403 1.00 0.00

ATOM 669 CB LYS 81 3.939 14.265 -18.785 1.00 0.00

ATOM 670 CG LYS 81 3.070 15.146 -19.673 1.00 0.00

ATOM 671 CD LYS 81 3.914 16.121 -20.481 1.00 0.00

ATOM 672 CE LYS 81 3.048 16.992 -21.379 1.00 0.00

ATOM 673 NZ LYS 81 3.856 17.984 -22.139 1.00 0.00

ATOM 674 N VAL 82 1.000 12.655 -18.820 1.00 0.00

ATOM 675 CA VAL 82 0.069 11.861 -19.613 1.00 0.00

ATOM 676 C VAL 82 -0.767 12.744 -20.529 1.00 0.00

ATOM 677 O VAL 82 -1.223 13.815 -20.128 1.00 0.00

ATOM 678 CB VAL 82 -0.871 11.031 -18.719 1.00 0.00

ATOM 679 CG1 VAL 82 -0.072 10.050 -17.873 1.00 0.00

ATOM 680 CG2 VAL 82 -1.703 11.943 -17.830 1.00 0.00

ATOM 681 N THR 83 -0.964 12.291 -21.762 1.00 0.00

ATOM 682 CA THR 83 -1.742 13.042 -22.740 1.00 0.00

ATOM 683 C THR 83 -2.870 12.195 -23.315 1.00 0.00

ATOM 684 O THR 83 -3.621 12.648 -24.180 1.00 0.00

ATOM 685 CB THR 83 -0.859 13.553 -23.893 1.00 0.00

ATOM 686 OG1 THR 83 -0.236 12.441 -24.550 1.00 0.00

ATOM 687 CG2 THR 83 0.215 14.492 -23.366 1.00 0.00

ATOM 688 N SER 84 -2.986 10.964 -22.828 1.00 0.00

ATOM 689 CA SER 84 -4.018 10.048 -23.299 1.00 0.00

ATOM 690 C SER 84 -4.381 9.027 -22.228 1.00 0.00

ATOM 691 O SER 84 -3.509 8.517 -21.524 1.00 0.00

ATOM 692 CB SER 84 -3.555 9.346 -24.562 1.00 0.00

ATOM 693 OG SER 84 -4.503 8.428 -25.031 1.00 0.00

ATOM 694 N PRO 85 -5.671 8.733 -22.111 1.00 0.00

ATOM 695 CA PRO 85 -6.150 7.765 -21.131 1.00 0.00

ATOM 696 C PRO 85 -5.342 6.475 -21.192 1.00 0.00

ATOM 697 O PRO 85 -5.077 5.848 -20.167 1.00 0.00

ATOM 698 CB PRO 85 -7.618 7.545 -21.509 1.00 0.00

ATOM 699 CG PRO 85 -8.039 8.833 -22.130 1.00 0.00

ATOM 700 CD PRO 85 -6.857 9.289 -22.943 1.00 0.00

ATOM 701 N ASP 86 -4.954 6.082 -22.401 1.00 0.00

ATOM 702 CA ASP 86 -4.154 4.879 -22.596 1.00 0.00

ATOM 703 C ASP 86 -2.802 5.000 -21.906 1.00 0.00

ATOM 704 O ASP 86 -2.293 4.032 -21.340 1.00 0.00

ATOM 705 CB ASP 86 -3.959 4.599 -24.088 1.00 0.00

ATOM 706 CG ASP 86 -5.203 4.085 -24.799 1.00 0.00

ATOM 707 OD1 ASP 86 -6.140 3.722 -24.128 1.00 0.00

ATOM 708 OD2 ASP 86 -5.269 4.204 -25.999 1.00 0.00

ATOM 709 N GLU 87 -2.222 6.194 -21.956 1.00 0.00

ATOM 710 CA GLU 87 -0.927 6.445 -21.334 1.00 0.00

ATOM 711 C GLU 87 -1.027 6.396 -19.815 1.00 0.00

ATOM 712 O GLU 87 -0.170 5.819 -19.146 1.00 0.00

ATOM 713 CB GLU 87 -0.371 7.799 -21.781 1.00 0.00

ATOM 714 CG GLU 87 0.066 7.850 -23.238 1.00 0.00

ATOM 715 CD GLU 87 0.478 9.239 -23.637 1.00 0.00

ATOM 716 OE1 GLU 87 0.355 10.130 -22.830 1.00 0.00

ATOM 717 OE2 GLU 87 1.019 9.393 -24.706 1.00 0.00

ATOM 718 N ALA 88 -2.077 7.006 -19.275 1.00 0.00

ATOM 719 CA ALA 88 -2.315 6.994 -17.837 1.00 0.00

ATOM 720 C ALA 88 -2.496 5.573 -17.321 1.00 0.00

ATOM 721 O ALA 88 -1.954 5.207 -16.278 1.00 0.00

ATOM 722 CB ALA 88 -3.530 7.844 -17.494 1.00 0.00

ATOM 723 N LYS 89 -3.263 4.775 -18.057 1.00 0.00

ATOM 724 CA LYS 89 -3.504 3.386 -17.682 1.00 0.00

ATOM 725 C LYS 89 -2.215 2.575 -17.721 1.00 0.00

ATOM 726 O LYS 89 -1.951 1.769 -16.827 1.00 0.00

ATOM 727 CB LYS 89 -4.550 2.756 -18.603 1.00 0.00

ATOM 728 CG LYS 89 -5.969 3.265 -18.385 1.00 0.00

ATOM 729 CD LYS 89 -6.969 2.501 -19.239 1.00 0.00

ATOM 730 CE LYS 89 -6.867 2.902 -20.704 1.00 0.00

ATOM 731 NZ LYS 89 -7.889 2.217 -21.541 1.00 0.00

ATOM 732 N ARG 90 -1.417 2.790 -18.760 1.00 0.00

ATOM 733 CA ARG 90 -0.130 2.117 -18.891 1.00 0.00

ATOM 734 C ARG 90 0.769 2.408 -17.696 1.00 0.00

ATOM 735 O ARG 90 1.379 1.501 -17.130 1.00 0.00

ATOM 736 CB ARG 90 0.562 2.453 -20.203 1.00 0.00

ATOM 737 CG ARG 90 1.875 1.723 -20.439 1.00 0.00

ATOM 738 CD ARG 90 2.577 2.113 -21.689 1.00 0.00

ATOM 739 NE ARG 90 3.045 3.489 -21.712 1.00 0.00

ATOM 740 CZ ARG 90 4.151 3.932 -21.081 1.00 0.00

ATOM 741 NH1 ARG 90 4.882 3.122 -20.348 1.00 0.00

ATOM 742 NH2 ARG 90 4.468 5.210 -21.200 1.00 0.00

ATOM 743 N TRP 91 0.846 3.679 -17.317 1.00 0.00

ATOM 744 CA TRP 91 1.679 4.094 -16.194 1.00 0.00

ATOM 745 C TRP 91 1.205 3.459 -14.893 1.00 0.00

ATOM 746 O TRP 91 2.012 2.991 -14.090 1.00 0.00

ATOM 747 CB TRP 91 1.678 5.619 -16.065 1.00 0.00

ATOM 748 CG TRP 91 2.706 6.292 -16.923 1.00 0.00

ATOM 749 CD1 TRP 91 2.481 7.259 -17.857 1.00 0.00

ATOM 750 CD2 TRP 91 4.118 6.051 -16.926 1.00 0.00

ATOM 751 NE1 TRP 91 3.664 7.635 -18.443 1.00 0.00

ATOM 752 CE2 TRP 91 4.685 6.907 -17.886 1.00 0.00

ATOM 753 CE3 TRP 91 4.958 5.192 -16.206 1.00 0.00

ATOM 754 CZ2 TRP 91 6.046 6.931 -18.149 1.00 0.00

ATOM 755 CZ3 TRP 91 6.323 5.217 -16.469 1.00 0.00

ATOM 756 CH2 TRP 91 6.851 6.062 -17.412 1.00 0.00

ATOM 757 N ILE 92 -0.108 3.445 -14.691 1.00 0.00

ATOM 758 CA ILE 92 -0.692 2.847 -13.495 1.00 0.00

ATOM 759 C ILE 92 -0.393 1.355 -13.422 1.00 0.00

ATOM 760 O ILE 92 -0.095 0.824 -12.353 1.00 0.00

ATOM 761 CB ILE 92 -2.216 3.059 -13.445 1.00 0.00

ATOM 762 CG1 ILE 92 -2.544 4.541 -13.246 1.00 0.00

ATOM 763 CG2 ILE 92 -2.833 2.220 -12.336 1.00 0.00

ATOM 764 CD1 ILE 92 -4.004 4.877 -13.448 1.00 0.00

ATOM 765 N LYS 93 -0.474 0.684 -14.566 1.00 0.00

ATOM 766 CA LYS 93 -0.185 -0.743 -14.639 1.00 0.00

ATOM 767 C LYS 93 1.241 -1.039 -14.191 1.00 0.00

ATOM 768 O LYS 93 1.469 -1.912 -13.353 1.00 0.00

ATOM 769 CB LYS 93 -0.409 -1.263 -16.060 1.00 0.00

ATOM 770 CG LYS 93 -0.170 -2.758 -16.228 1.00 0.00

ATOM 771 CD LYS 93 -0.478 -3.212 -17.646 1.00 0.00

ATOM 772 CE LYS 93 -0.194 -4.696 -17.828 1.00 0.00

ATOM 773 NZ LYS 93 -0.471 -5.150 -19.218 1.00 0.00

ATOM 774 N GLU 94 2.197 -0.308 -14.754 1.00 0.00

ATOM 775 CA GLU 94 3.601 -0.480 -14.400 1.00 0.00

ATOM 776 C GLU 94 3.842 -0.151 -12.933 1.00 0.00

ATOM 777 O GLU 94 4.620 -0.824 -12.255 1.00 0.00

ATOM 778 CB GLU 94 4.489 0.393 -15.288 1.00 0.00

ATOM 779 CG GLU 94 4.575 -0.066 -16.737 1.00 0.00

ATOM 780 CD GLU 94 5.363 0.904 -17.572 1.00 0.00

ATOM 781 OE1 GLU 94 5.757 1.922 -17.053 1.00 0.00

ATOM 782 OE2 GLU 94 5.669 0.579 -18.695 1.00 0.00

ATOM 783 N PHE 95 3.173 0.888 -12.446 1.00 0.00

ATOM 784 CA PHE 95 3.275 1.278 -11.045 1.00 0.00

ATOM 785 C PHE 95 2.858 0.138 -10.124 1.00 0.00

ATOM 786 O PHE 95 3.540 -0.163 -9.144 1.00 0.00

ATOM 787 CB PHE 95 2.419 2.516 -10.771 1.00 0.00

ATOM 788 CG PHE 95 2.445 2.965 -9.338 1.00 0.00

ATOM 789 CD1 PHE 95 3.607 3.475 -8.777 1.00 0.00

ATOM 790 CD2 PHE 95 1.309 2.879 -8.547 1.00 0.00

ATOM 791 CE1 PHE 95 3.632 3.889 -7.458 1.00 0.00

ATOM 792 CE2 PHE 95 1.331 3.293 -7.229 1.00 0.00

ATOM 793 CZ PHE 95 2.495 3.798 -6.685 1.00 0.00

ATOM 794 N SER 96 1.734 -0.494 -10.445 1.00 0.00

ATOM 795 CA SER 96 1.245 -1.628 -9.670 1.00 0.00

ATOM 796 C SER 96 2.228 -2.792 -9.718 1.00 0.00

ATOM 797 O SER 96 2.484 -3.441 -8.704 1.00 0.00

ATOM 798 CB SER 96 -0.114 -2.063 -10.182 1.00 0.00

ATOM 799 OG SER 96 -0.615 -3.164 -9.475 1.00 0.00

ATOM 800 N GLU 97 2.775 -3.048 -10.900 1.00 0.00

ATOM 801 CA GLU 97 3.762 -4.108 -11.073 1.00 0.00

ATOM 802 C GLU 97 4.956 -3.905 -10.148 1.00 0.00

ATOM 803 O GLU 97 5.438 -4.851 -9.524 1.00 0.00

ATOM 804 CB GLU 97 4.230 -4.172 -12.528 1.00 0.00

ATOM 805 CG GLU 97 3.189 -4.708 -13.500 1.00 0.00

ATOM 806 CD GLU 97 3.678 -4.636 -14.920 1.00 0.00

ATOM 807 OE1 GLU 97 4.748 -4.119 -15.134 1.00 0.00

ATOM 808 OE2 GLU 97 3.038 -5.198 -15.777 1.00 0.00

ATOM 809 N GLU 98 5.429 -2.667 -10.064 1.00 0.00

ATOM 810 CA GLU 98 6.537 -2.328 -9.177 1.00 0.00

ATOM 811 C GLU 98 6.170 -2.574 -7.720 1.00 0.00

ATOM 812 O GLU 98 6.942 -3.171 -6.968 1.00 0.00

ATOM 813 CB GLU 98 6.954 -0.869 -9.375 1.00 0.00

ATOM 814 CG GLU 98 8.143 -0.435 -8.529 1.00 0.00

ATOM 815 CD GLU 98 8.519 0.993 -8.808 1.00 0.00

ATOM 816 OE1 GLU 98 7.885 1.605 -9.633 1.00 0.00

ATOM 817 OE2 GLU 98 9.367 1.509 -8.118 1.00 0.00

ATOM 818 N GLY 99 4.989 -2.113 -7.325 1.00 0.00

ATOM 819 CA GLY 99 4.515 -2.290 -5.958 1.00 0.00

ATOM 820 C GLY 99 4.580 -3.753 -5.537 1.00 0.00

ATOM 821 O GLY 99 5.011 -4.072 -4.430 1.00 0.00

ATOM 822 OXT GLY 99 4.207 -4.610 -6.289 1.00 0.00

TER

ENDMDL

**PDB ID 2LN3**

ATOM 1 N LEU A 1 0.000 0.000 0.000 1.00 0.00

ATOM 2 CA LEU A 1 1.458 0.000 0.000 1.00 0.00

ATOM 3 C LEU A 1 2.009 1.420 0.000 1.00 0.00

ATOM 4 O LEU A 1 1.387 2.337 -0.536 1.00 0.00

ATOM 5 CB LEU A 1 1.988 -0.778 -1.211 1.00 0.00

ATOM 6 CG LEU A 1 1.543 -2.244 -1.291 1.00 0.00

ATOM 7 CD1 LEU A 1 2.035 -2.869 -2.589 1.00 0.00

ATOM 8 CD2 LEU A 1 2.082 -3.005 -0.088 1.00 0.00

ATOM 9 N THR A 2 3.179 1.596 0.605 1.00 0.00

ATOM 10 CA THR A 2 3.829 2.899 0.652 1.00 0.00

ATOM 11 C THR A 2 5.312 2.789 0.326 1.00 0.00

ATOM 12 O THR A 2 6.048 2.048 0.980 1.00 0.00

ATOM 13 CB THR A 2 3.666 3.561 2.033 1.00 0.00

ATOM 14 OG1 THR A 2 2.272 3.725 2.326 1.00 0.00

ATOM 15 CG2 THR A 2 4.348 4.921 2.057 1.00 0.00

ATOM 16 N ARG A 3 5.747 3.528 -0.688 1.00 0.00

ATOM 17 CA ARG A 3 7.142 3.503 -1.113 1.00 0.00

ATOM 18 C ARG A 3 7.680 4.912 -1.321 1.00 0.00

ATOM 19 O ARG A 3 6.920 5.843 -1.588 1.00 0.00

ATOM 20 CB ARG A 3 7.351 2.638 -2.347 1.00 0.00

ATOM 21 CG ARG A 3 6.983 1.174 -2.173 1.00 0.00

ATOM 22 CD ARG A 3 7.880 0.421 -1.260 1.00 0.00

ATOM 23 NE ARG A 3 7.590 -1.001 -1.166 1.00 0.00

ATOM 24 CZ ARG A 3 6.701 -1.545 -0.312 1.00 0.00

ATOM 25 NH1 ARG A 3 6.043 -0.799 0.548 1.00 0.00

ATOM 26 NH2 ARG A 3 6.526 -2.855 -0.344 1.00 0.00

ATOM 27 N THR A 4 8.994 5.062 -1.200 1.00 0.00

ATOM 28 CA THR A 4 9.641 6.353 -1.404 1.00 0.00

ATOM 29 C THR A 4 10.858 6.222 -2.311 1.00 0.00

ATOM 30 O THR A 4 11.790 5.476 -2.010 1.00 0.00

ATOM 31 CB THR A 4 10.074 6.986 -0.069 1.00 0.00

ATOM 32 OG1 THR A 4 8.930 7.153 0.778 1.00 0.00

ATOM 33 CG2 THR A 4 10.726 8.339 -0.306 1.00 0.00

ATOM 34 N ILE A 5 10.844 6.952 -3.421 1.00 0.00

ATOM 35 CA ILE A 5 11.935 6.897 -4.387 1.00 0.00

ATOM 36 C ILE A 5 12.717 8.204 -4.408 1.00 0.00

ATOM 37 O ILE A 5 12.145 9.280 -4.587 1.00 0.00

ATOM 38 CB ILE A 5 11.419 6.595 -5.805 1.00 0.00

ATOM 39 CG1 ILE A 5 10.548 5.336 -5.799 1.00 0.00

ATOM 40 CG2 ILE A 5 12.582 6.437 -6.772 1.00 0.00

ATOM 41 CD1 ILE A 5 11.284 4.087 -5.372 1.00 0.00

ATOM 42 N THR A 6 14.029 8.105 -4.226 1.00 0.00

ATOM 43 CA THR A 6 14.896 9.278 -4.239 1.00 0.00

ATOM 44 C THR A 6 15.901 9.207 -5.382 1.00 0.00

ATOM 45 O THR A 6 16.663 8.247 -5.492 1.00 0.00

ATOM 46 CB THR A 6 15.657 9.434 -2.910 1.00 0.00

ATOM 47 OG1 THR A 6 14.721 9.540 -1.830 1.00 0.00

ATOM 48 CG2 THR A 6 16.533 10.678 -2.940 1.00 0.00

ATOM 49 N SER A 7 15.897 10.229 -6.231 1.00 0.00

ATOM 50 CA SER A 7 16.803 10.282 -7.372 1.00 0.00

ATOM 51 C SER A 7 16.649 11.589 -8.138 1.00 0.00

ATOM 52 O SER A 7 15.698 12.340 -7.919 1.00 0.00

ATOM 53 CB SER A 7 16.557 9.100 -8.288 1.00 0.00

ATOM 54 OG SER A 7 17.161 9.270 -9.541 1.00 0.00

ATOM 55 N GLN A 8 17.587 11.855 -9.040 1.00 0.00

ATOM 56 CA GLN A 8 17.534 13.052 -9.871 1.00 0.00

ATOM 57 C GLN A 8 16.842 12.769 -11.198 1.00 0.00

ATOM 58 O GLN A 8 16.626 13.677 -12.002 1.00 0.00

ATOM 59 CB GLN A 8 18.945 13.589 -10.129 1.00 0.00

ATOM 60 CG GLN A 8 19.510 14.423 -8.992 1.00 0.00

ATOM 61 CD GLN A 8 19.735 13.609 -7.732 1.00 0.00

ATOM 62 OE1 GLN A 8 20.174 12.457 -7.792 1.00 0.00

ATOM 63 NE2 GLN A 8 19.431 14.201 -6.583 1.00 0.00

ATOM 64 N ASN A 9 16.496 11.507 -11.422 1.00 0.00

ATOM 65 CA ASN A 9 15.835 11.101 -12.657 1.00 0.00

ATOM 66 C ASN A 9 14.366 11.502 -12.652 1.00 0.00

ATOM 67 O ASN A 9 13.481 10.653 -12.550 1.00 0.00

ATOM 68 CB ASN A 9 15.971 9.608 -12.895 1.00 0.00

ATOM 69 CG ASN A 9 17.377 9.169 -13.198 1.00 0.00

ATOM 70 OD1 ASN A 9 18.211 9.960 -13.654 1.00 0.00

ATOM 71 ND2 ASN A 9 17.620 7.895 -13.024 1.00 0.00

ATOM 72 N LYS A 10 14.112 12.802 -12.765 1.00 0.00

ATOM 73 CA LYS A 10 12.749 13.319 -12.767 1.00 0.00

ATOM 74 C LYS A 10 11.820 12.420 -13.573 1.00 0.00

ATOM 75 O LYS A 10 10.719 12.093 -13.129 1.00 0.00

ATOM 76 CB LYS A 10 12.717 14.742 -13.326 1.00 0.00

ATOM 77 CG LYS A 10 12.907 14.830 -14.835 1.00 0.00

ATOM 78 CD LYS A 10 12.956 16.277 -15.303 1.00 0.00

ATOM 79 CE LYS A 10 13.167 16.366 -16.807 1.00 0.00

ATOM 80 NZ LYS A 10 13.221 17.776 -17.279 1.00 0.00

ATOM 81 N GLU A 11 12.270 12.021 -14.757 1.00 0.00

ATOM 82 CA GLU A 11 11.480 11.157 -15.626 1.00 0.00

ATOM 83 C GLU A 11 11.101 9.862 -14.918 1.00 0.00

ATOM 84 O GLU A 11 9.960 9.407 -15.005 1.00 0.00

ATOM 85 CB GLU A 11 12.248 10.848 -16.913 1.00 0.00

ATOM 86 CG GLU A 11 12.376 12.028 -17.867 1.00 0.00

ATOM 87 CD GLU A 11 13.234 11.681 -19.052 1.00 0.00

ATOM 88 OE1 GLU A 11 13.764 10.596 -19.081 1.00 0.00

ATOM 89 OE2 GLU A 11 13.270 12.453 -19.980 1.00 0.00

ATOM 90 N GLU A 12 12.065 9.272 -14.219 1.00 0.00

ATOM 91 CA GLU A 12 11.842 8.012 -13.520 1.00 0.00

ATOM 92 C GLU A 12 10.865 8.188 -12.365 1.00 0.00

ATOM 93 O GLU A 12 10.005 7.339 -12.133 1.00 0.00

ATOM 94 CB GLU A 12 13.167 7.443 -13.008 1.00 0.00

ATOM 95 CG GLU A 12 13.052 6.073 -12.354 1.00 0.00

ATOM 96 CD GLU A 12 14.401 5.545 -11.953 1.00 0.00

ATOM 97 OE1 GLU A 12 15.375 6.218 -12.187 1.00 0.00

ATOM 98 OE2 GLU A 12 14.449 4.521 -11.313 1.00 0.00

ATOM 99 N LEU A 13 11.004 9.293 -11.641 1.00 0.00

ATOM 100 CA LEU A 13 10.125 9.590 -10.518 1.00 0.00

ATOM 101 C LEU A 13 8.686 9.779 -10.979 1.00 0.00

ATOM 102 O LEU A 13 7.748 9.325 -10.322 1.00 0.00

ATOM 103 CB LEU A 13 10.615 10.840 -9.776 1.00 0.00

ATOM 104 CG LEU A 13 11.950 10.679 -9.038 1.00 0.00

ATOM 105 CD1 LEU A 13 12.407 12.022 -8.486 1.00 0.00

ATOM 106 CD2 LEU A 13 11.793 9.661 -7.918 1.00 0.00

ATOM 107 N LEU A 14 8.516 10.450 -12.113 1.00 0.00

ATOM 108 CA LEU A 14 7.191 10.694 -12.670 1.00 0.00

ATOM 109 C LEU A 14 6.523 9.391 -13.091 1.00 0.00

ATOM 110 O LEU A 14 5.356 9.152 -12.781 1.00 0.00

ATOM 111 CB LEU A 14 7.284 11.656 -13.861 1.00 0.00

ATOM 112 CG LEU A 14 7.678 13.096 -13.508 1.00 0.00

ATOM 113 CD1 LEU A 14 7.984 13.879 -14.777 1.00 0.00

ATOM 114 CD2 LEU A 14 6.551 13.754 -12.726 1.00 0.00

ATOM 115 N GLU A 15 7.270 8.551 -13.800 1.00 0.00

ATOM 116 CA GLU A 15 6.747 7.277 -14.279 1.00 0.00

ATOM 117 C GLU A 15 6.336 6.381 -13.118 1.00 0.00

ATOM 118 O GLU A 15 5.255 5.792 -13.130 1.00 0.00

ATOM 119 CB GLU A 15 7.783 6.566 -15.151 1.00 0.00

ATOM 120 CG GLU A 15 8.021 7.220 -16.505 1.00 0.00

ATOM 121 CD GLU A 15 9.141 6.548 -17.248 1.00 0.00

ATOM 122 OE1 GLU A 15 9.750 5.665 -16.693 1.00 0.00

ATOM 123 OE2 GLU A 15 9.317 6.838 -18.408 1.00 0.00

ATOM 124 N ILE A 16 7.205 6.279 -12.118 1.00 0.00

ATOM 125 CA ILE A 16 6.936 5.449 -10.951 1.00 0.00

ATOM 126 C ILE A 16 5.729 5.962 -10.177 1.00 0.00

ATOM 127 O ILE A 16 4.850 5.188 -9.795 1.00 0.00

ATOM 128 CB ILE A 16 8.152 5.389 -10.008 1.00 0.00

ATOM 129 CG1 ILE A 16 9.307 4.635 -10.673 1.00 0.00

ATOM 130 CG2 ILE A 16 7.771 4.731 -8.691 1.00 0.00

ATOM 131 CD1 ILE A 16 10.621 4.756 -9.936 1.00 0.00

ATOM 132 N ALA A 17 5.693 7.270 -9.946 1.00 0.00

ATOM 133 CA ALA A 17 4.583 7.891 -9.234 1.00 0.00

ATOM 134 C ALA A 17 3.252 7.579 -9.906 1.00 0.00

ATOM 135 O ALA A 17 2.275 7.233 -9.243 1.00 0.00

ATOM 136 CB ALA A 17 4.792 9.395 -9.138 1.00 0.00

ATOM 137 N LEU A 18 3.221 7.703 -11.229 1.00 0.00

ATOM 138 CA LEU A 18 2.011 7.428 -11.996 1.00 0.00

ATOM 139 C LEU A 18 1.594 5.968 -11.860 1.00 0.00

ATOM 140 O LEU A 18 0.425 5.667 -11.620 1.00 0.00

ATOM 141 CB LEU A 18 2.226 7.787 -13.472 1.00 0.00

ATOM 142 CG LEU A 18 1.026 7.520 -14.390 1.00 0.00

ATOM 143 CD1 LEU A 18 -0.177 8.330 -13.925 1.00 0.00

ATOM 144 CD2 LEU A 18 1.393 7.875 -15.823 1.00 0.00

ATOM 145 N LYS A 19 2.557 5.066 -12.018 1.00 0.00

ATOM 146 CA LYS A 19 2.284 3.635 -11.949 1.00 0.00

ATOM 147 C LYS A 19 1.692 3.252 -10.599 1.00 0.00

ATOM 148 O LYS A 19 0.788 2.420 -10.522 1.00 0.00

ATOM 149 CB LYS A 19 3.561 2.834 -12.210 1.00 0.00

ATOM 150 CG LYS A 19 4.035 2.860 -13.656 1.00 0.00

ATOM 151 CD LYS A 19 5.335 2.087 -13.827 1.00 0.00

ATOM 152 CE LYS A 19 5.825 2.136 -15.266 1.00 0.00

ATOM 153 NZ LYS A 19 7.099 1.389 -15.446 1.00 0.00

ATOM 154 N PHE A 20 2.205 3.864 -9.537 1.00 0.00

ATOM 155 CA PHE A 20 1.753 3.560 -8.185 1.00 0.00

ATOM 156 C PHE A 20 0.343 4.083 -7.945 1.00 0.00

ATOM 157 O PHE A 20 -0.542 3.339 -7.520 1.00 0.00

ATOM 158 CB PHE A 20 2.717 4.150 -7.155 1.00 0.00

ATOM 159 CG PHE A 20 3.776 3.190 -6.695 1.00 0.00

ATOM 160 CD1 PHE A 20 4.867 2.900 -7.501 1.00 0.00

ATOM 161 CD2 PHE A 20 3.684 2.573 -5.456 1.00 0.00

ATOM 162 CE1 PHE A 20 5.842 2.016 -7.079 1.00 0.00

ATOM 163 CE2 PHE A 20 4.657 1.690 -5.031 1.00 0.00

ATOM 164 CZ PHE A 20 5.738 1.411 -5.844 1.00 0.00

ATOM 165 N ILE A 21 0.138 5.366 -8.220 1.00 0.00

ATOM 166 CA ILE A 21 -1.137 6.015 -7.944 1.00 0.00

ATOM 167 C ILE A 21 -2.259 5.399 -8.771 1.00 0.00

ATOM 168 O ILE A 21 -3.412 5.360 -8.340 1.00 0.00

ATOM 169 CB ILE A 21 -1.073 7.527 -8.227 1.00 0.00

ATOM 170 CG1 ILE A 21 -0.774 7.782 -9.707 1.00 0.00

ATOM 171 CG2 ILE A 21 -0.024 8.189 -7.348 1.00 0.00

ATOM 172 CD1 ILE A 21 -0.737 9.246 -10.079 1.00 0.00

ATOM 173 N SER A 22 -1.914 4.917 -9.960 1.00 0.00

ATOM 174 CA SER A 22 -2.888 4.282 -10.840 1.00 0.00

ATOM 175 C SER A 22 -3.484 3.037 -10.194 1.00 0.00

ATOM 176 O SER A 22 -4.549 2.568 -10.595 1.00 0.00

ATOM 177 CB SER A 22 -2.242 3.931 -12.166 1.00 0.00

ATOM 178 OG SER A 22 -1.496 2.748 -12.091 1.00 0.00

ATOM 179 N GLN A 23 -2.791 2.508 -9.192 1.00 0.00

ATOM 180 CA GLN A 23 -3.245 1.309 -8.497 1.00 0.00

ATOM 181 C GLN A 23 -3.736 1.638 -7.094 1.00 0.00

ATOM 182 O GLN A 23 -4.029 0.742 -6.301 1.00 0.00

ATOM 183 CB GLN A 23 -2.118 0.275 -8.420 1.00 0.00

ATOM 184 CG GLN A 23 -1.481 -0.053 -9.759 1.00 0.00

ATOM 185 CD GLN A 23 -2.474 -0.643 -10.743 1.00 0.00

ATOM 186 OE1 GLN A 23 -3.126 -1.652 -10.458 1.00 0.00

ATOM 187 NE2 GLN A 23 -2.593 -0.019 -11.909 1.00 0.00

ATOM 188 N GLY A 24 -3.825 2.929 -6.791 1.00 0.00

ATOM 189 CA GLY A 24 -4.286 3.378 -5.483 1.00 0.00

ATOM 190 C GLY A 24 -3.213 3.180 -4.421 1.00 0.00

ATOM 191 O GLY A 24 -3.515 3.050 -3.235 1.00 0.00

ATOM 192 N LEU A 25 -1.957 3.157 -4.855 1.00 0.00

ATOM 193 CA LEU A 25 -0.836 2.972 -3.942 1.00 0.00

ATOM 194 C LEU A 25 -0.181 4.303 -3.595 1.00 0.00

ATOM 195 O LEU A 25 -0.268 5.265 -4.358 1.00 0.00

ATOM 196 CB LEU A 25 0.194 2.014 -4.556 1.00 0.00

ATOM 197 CG LEU A 25 -0.363 0.657 -5.005 1.00 0.00

ATOM 198 CD1 LEU A 25 0.742 -0.177 -5.639 1.00 0.00

ATOM 199 CD2 LEU A 25 -0.966 -0.066 -3.810 1.00 0.00

ATOM 200 N ASP A 26 0.475 4.350 -2.441 1.00 0.00

ATOM 201 CA ASP A 26 1.144 5.565 -1.990 1.00 0.00

ATOM 202 C ASP A 26 2.588 5.610 -2.473 1.00 0.00

ATOM 203 O ASP A 26 3.362 4.685 -2.234 1.00 0.00

ATOM 204 CB ASP A 26 1.097 5.668 -0.464 1.00 0.00

ATOM 205 CG ASP A 26 1.684 6.956 0.098 1.00 0.00

ATOM 206 OD1 ASP A 26 2.191 7.739 -0.670 1.00 0.00

ATOM 207 OD2 ASP A 26 1.484 7.219 1.260 1.00 0.00

ATOM 208 N LEU A 27 2.945 6.693 -3.156 1.00 0.00

ATOM 209 CA LEU A 27 4.282 6.839 -3.719 1.00 0.00

ATOM 210 C LEU A 27 4.820 8.247 -3.501 1.00 0.00

ATOM 211 O LEU A 27 4.221 9.227 -3.943 1.00 0.00

ATOM 212 CB LEU A 27 4.267 6.498 -5.215 1.00 0.00

ATOM 213 CG LEU A 27 5.645 6.242 -5.839 1.00 0.00

ATOM 214 CD1 LEU A 27 6.354 7.564 -6.099 1.00 0.00

ATOM 215 CD2 LEU A 27 6.469 5.362 -4.911 1.00 0.00

ATOM 216 N GLU A 28 5.956 8.340 -2.817 1.00 0.00

ATOM 217 CA GLU A 28 6.612 9.623 -2.595 1.00 0.00

ATOM 218 C GLU A 28 7.873 9.752 -3.441 1.00 0.00

ATOM 219 O GLU A 28 8.673 8.820 -3.523 1.00 0.00

ATOM 220 CB GLU A 28 6.952 9.800 -1.113 1.00 0.00

ATOM 221 CG GLU A 28 7.554 11.154 -0.764 1.00 0.00

ATOM 222 CD GLU A 28 7.975 11.209 0.678 1.00 0.00

ATOM 223 OE1 GLU A 28 7.229 10.758 1.513 1.00 0.00

ATOM 224 OE2 GLU A 28 9.090 11.598 0.936 1.00 0.00

ATOM 225 N VAL A 29 8.042 10.910 -4.069 1.00 0.00

ATOM 226 CA VAL A 29 9.216 11.170 -4.894 1.00 0.00

ATOM 227 C VAL A 29 10.070 12.283 -4.303 1.00 0.00

ATOM 228 O VAL A 29 9.561 13.343 -3.938 1.00 0.00

ATOM 229 CB VAL A 29 8.821 11.549 -6.334 1.00 0.00

ATOM 230 CG1 VAL A 29 7.962 10.458 -6.956 1.00 0.00

ATOM 231 CG2 VAL A 29 8.084 12.880 -6.352 1.00 0.00

ATOM 232 N GLU A 30 11.372 12.036 -4.211 1.00 0.00

ATOM 233 CA GLU A 30 12.303 13.022 -3.672 1.00 0.00

ATOM 234 C GLU A 30 13.248 13.533 -4.752 1.00 0.00

ATOM 235 O GLU A 30 14.070 12.783 -5.278 1.00 0.00

ATOM 236 CB GLU A 30 13.103 12.427 -2.512 1.00 0.00

ATOM 237 CG GLU A 30 12.263 12.044 -1.301 1.00 0.00

ATOM 238 CD GLU A 30 13.130 11.732 -0.112 1.00 0.00

ATOM 239 OE1 GLU A 30 14.328 11.805 -0.239 1.00 0.00

ATOM 240 OE2 GLU A 30 12.594 11.530 0.952 1.00 0.00

ATOM 241 N PHE A 31 13.128 14.816 -5.078 1.00 0.00

ATOM 242 CA PHE A 31 13.953 15.424 -6.114 1.00 0.00

ATOM 243 C PHE A 31 14.883 16.480 -5.529 1.00 0.00

ATOM 244 O PHE A 31 14.455 17.587 -5.206 1.00 0.00

ATOM 245 CB PHE A 31 13.074 16.042 -7.203 1.00 0.00

ATOM 246 CG PHE A 31 13.848 16.589 -8.368 1.00 0.00

ATOM 247 CD1 PHE A 31 14.348 15.742 -9.346 1.00 0.00

ATOM 248 CD2 PHE A 31 14.077 17.951 -8.490 1.00 0.00

ATOM 249 CE1 PHE A 31 15.061 16.245 -10.419 1.00 0.00

ATOM 250 CE2 PHE A 31 14.787 18.456 -9.561 1.00 0.00

ATOM 251 CZ PHE A 31 15.280 17.601 -10.527 1.00 0.00

ATOM 252 N ASP A 32 16.158 16.129 -5.396 1.00 0.00

ATOM 253 CA ASP A 32 17.152 17.046 -4.850 1.00 0.00

ATOM 254 C ASP A 32 17.980 17.683 -5.958 1.00 0.00

ATOM 255 O ASP A 32 18.778 17.013 -6.614 1.00 0.00

ATOM 256 CB ASP A 32 18.067 16.319 -3.862 1.00 0.00

ATOM 257 CG ASP A 32 19.103 17.210 -3.191 1.00 0.00

ATOM 258 OD1 ASP A 32 19.171 18.368 -3.531 1.00 0.00

ATOM 259 OD2 ASP A 32 19.708 16.773 -2.241 1.00 0.00

ATOM 260 N SER A 33 17.785 18.982 -6.162 1.00 0.00

ATOM 261 CA SER A 33 18.509 19.711 -7.198 1.00 0.00

ATOM 262 C SER A 33 18.600 21.195 -6.863 1.00 0.00

ATOM 263 O SER A 33 17.919 21.681 -5.960 1.00 0.00

ATOM 264 CB SER A 33 17.839 19.513 -8.543 1.00 0.00

ATOM 265 OG SER A 33 18.349 20.380 -9.519 1.00 0.00

ATOM 266 N THR A 34 19.444 21.910 -7.599 1.00 0.00

ATOM 267 CA THR A 34 19.603 23.346 -7.403 1.00 0.00

ATOM 268 C THR A 34 19.016 24.129 -8.571 1.00 0.00

ATOM 269 O THR A 34 19.026 25.360 -8.573 1.00 0.00

ATOM 270 CB THR A 34 21.084 23.733 -7.231 1.00 0.00

ATOM 271 OG1 THR A 34 21.819 23.349 -8.400 1.00 0.00

ATOM 272 CG2 THR A 34 21.678 23.042 -6.013 1.00 0.00

ATOM 273 N ASP A 35 18.506 23.408 -9.563 1.00 0.00

ATOM 274 CA ASP A 35 17.907 24.033 -10.736 1.00 0.00

ATOM 275 C ASP A 35 16.444 24.373 -10.492 1.00 0.00

ATOM 276 O ASP A 35 15.594 23.485 -10.421 1.00 0.00

ATOM 277 CB ASP A 35 18.038 23.119 -11.957 1.00 0.00

ATOM 278 CG ASP A 35 17.643 23.770 -13.275 1.00 0.00

ATOM 279 OD1 ASP A 35 16.628 24.425 -13.312 1.00 0.00

ATOM 280 OD2 ASP A 35 18.433 23.741 -14.189 1.00 0.00

ATOM 281 N ASP A 36 16.154 25.665 -10.363 1.00 0.00

ATOM 282 CA ASP A 36 14.798 26.122 -10.083 1.00 0.00

ATOM 283 C ASP A 36 13.838 25.707 -11.191 1.00 0.00

ATOM 284 O ASP A 36 12.703 25.311 -10.925 1.00 0.00

ATOM 285 CB ASP A 36 14.771 27.641 -9.905 1.00 0.00

ATOM 286 CG ASP A 36 15.386 28.133 -8.602 1.00 0.00

ATOM 287 OD1 ASP A 36 15.624 27.324 -7.737 1.00 0.00

ATOM 288 OD2 ASP A 36 15.759 29.281 -8.540 1.00 0.00

ATOM 289 N LYS A 37 14.299 25.800 -12.432 1.00 0.00

ATOM 290 CA LYS A 37 13.472 25.463 -13.585 1.00 0.00

ATOM 291 C LYS A 37 13.076 23.991 -13.567 1.00 0.00

ATOM 292 O LYS A 37 11.908 23.650 -13.756 1.00 0.00

ATOM 293 CB LYS A 37 14.205 25.794 -14.886 1.00 0.00

ATOM 294 CG LYS A 37 13.401 25.517 -16.149 1.00 0.00

ATOM 295 CD LYS A 37 14.163 25.943 -17.395 1.00 0.00

ATOM 296 CE LYS A 37 13.378 25.629 -18.660 1.00 0.00

ATOM 297 NZ LYS A 37 14.125 26.013 -19.888 1.00 0.00

ATOM 298 N GLU A 38 14.055 23.123 -13.339 1.00 0.00

ATOM 299 CA GLU A 38 13.812 21.686 -13.299 1.00 0.00

ATOM 300 C GLU A 38 12.886 21.316 -12.148 1.00 0.00

ATOM 301 O GLU A 38 12.011 20.462 -12.292 1.00 0.00

ATOM 302 CB GLU A 38 15.132 20.922 -13.179 1.00 0.00

ATOM 303 CG GLU A 38 15.994 20.954 -14.433 1.00 0.00

ATOM 304 CD GLU A 38 15.263 20.378 -15.614 1.00 0.00

ATOM 305 OE1 GLU A 38 14.766 19.283 -15.504 1.00 0.00

ATOM 306 OE2 GLU A 38 15.102 21.076 -16.587 1.00 0.00

ATOM 307 N ILE A 39 13.085 21.961 -11.004 1.00 0.00

ATOM 308 CA ILE A 39 12.267 21.702 -9.824 1.00 0.00

ATOM 309 C ILE A 39 10.813 22.084 -10.069 1.00 0.00

ATOM 310 O ILE A 39 9.899 21.331 -9.733 1.00 0.00

ATOM 311 CB ILE A 39 12.790 22.468 -8.595 1.00 0.00

ATOM 312 CG1 ILE A 39 14.142 21.906 -8.151 1.00 0.00

ATOM 313 CG2 ILE A 39 11.782 22.400 -7.458 1.00 0.00

ATOM 314 CD1 ILE A 39 14.868 22.779 -7.153 1.00 0.00

ATOM 315 N GLU A 40 10.605 23.257 -10.657 1.00 0.00

ATOM 316 CA GLU A 40 9.260 23.745 -10.940 1.00 0.00

ATOM 317 C GLU A 40 8.554 22.855 -11.954 1.00 0.00

ATOM 318 O GLU A 40 7.379 22.526 -11.792 1.00 0.00

ATOM 319 CB GLU A 40 9.309 25.187 -11.449 1.00 0.00

ATOM 320 CG GLU A 40 7.945 25.807 -11.716 1.00 0.00

ATOM 321 CD GLU A 40 8.072 27.235 -12.165 1.00 0.00

ATOM 322 OE1 GLU A 40 9.177 27.715 -12.251 1.00 0.00

ATOM 323 OE2 GLU A 40 7.076 27.813 -12.531 1.00 0.00

ATOM 324 N GLU A 41 9.277 22.468 -12.999 1.00 0.00

ATOM 325 CA GLU A 41 8.724 21.606 -14.036 1.00 0.00

ATOM 326 C GLU A 41 8.336 20.245 -13.473 1.00 0.00

ATOM 327 O GLU A 41 7.274 19.711 -13.791 1.00 0.00

ATOM 328 CB GLU A 41 9.725 21.435 -15.181 1.00 0.00

ATOM 329 CG GLU A 41 9.226 20.567 -16.328 1.00 0.00

ATOM 330 CD GLU A 41 10.219 20.530 -17.456 1.00 0.00

ATOM 331 OE1 GLU A 41 11.347 20.171 -17.218 1.00 0.00

ATOM 332 OE2 GLU A 41 9.823 20.751 -18.576 1.00 0.00

ATOM 333 N PHE A 42 9.204 19.688 -12.635 1.00 0.00

ATOM 334 CA PHE A 42 8.941 18.400 -12.003 1.00 0.00

ATOM 335 C PHE A 42 7.695 18.458 -11.130 1.00 0.00

ATOM 336 O PHE A 42 6.845 17.570 -11.184 1.00 0.00

ATOM 337 CB PHE A 42 10.146 17.957 -11.172 1.00 0.00

ATOM 338 CG PHE A 42 9.952 16.638 -10.478 1.00 0.00

ATOM 339 CD1 PHE A 42 9.922 15.455 -11.202 1.00 0.00

ATOM 340 CD2 PHE A 42 9.798 16.578 -9.102 1.00 0.00

ATOM 341 CE1 PHE A 42 9.744 14.242 -10.565 1.00 0.00

ATOM 342 CE2 PHE A 42 9.620 15.366 -8.462 1.00 0.00

ATOM 343 CZ PHE A 42 9.593 14.197 -9.196 1.00 0.00

ATOM 344 N GLU A 43 7.593 19.510 -10.325 1.00 0.00

ATOM 345 CA GLU A 43 6.436 19.701 -9.457 1.00 0.00

ATOM 346 C GLU A 43 5.148 19.788 -10.267 1.00 0.00

ATOM 347 O GLU A 43 4.143 19.168 -9.919 1.00 0.00

ATOM 348 CB GLU A 43 6.608 20.961 -8.605 1.00 0.00

ATOM 349 CG GLU A 43 5.534 21.153 -7.544 1.00 0.00

ATOM 350 CD GLU A 43 5.668 22.489 -6.868 1.00 0.00

ATOM 351 OE1 GLU A 43 6.730 22.780 -6.374 1.00 0.00

ATOM 352 OE2 GLU A 43 4.744 23.265 -6.942 1.00 0.00

ATOM 353 N ARG A 44 5.185 20.560 -11.347 1.00 0.00

ATOM 354 CA ARG A 44 4.023 20.725 -12.211 1.00 0.00

ATOM 355 C ARG A 44 3.587 19.394 -12.809 1.00 0.00

ATOM 356 O ARG A 44 2.397 19.081 -12.852 1.00 0.00

ATOM 357 CB ARG A 44 4.254 21.771 -13.291 1.00 0.00

ATOM 358 CG ARG A 44 4.343 23.203 -12.786 1.00 0.00

ATOM 359 CD ARG A 44 4.550 24.216 -13.853 1.00 0.00

ATOM 360 NE ARG A 44 4.734 25.574 -13.366 1.00 0.00

ATOM 361 CZ ARG A 44 3.734 26.393 -12.986 1.00 0.00

ATOM 362 NH1 ARG A 44 2.478 26.012 -13.070 1.00 0.00

ATOM 363 NH2 ARG A 44 4.047 27.600 -12.549 1.00 0.00

ATOM 364 N ASP A 45 4.557 18.613 -13.271 1.00 0.00

ATOM 365 CA ASP A 45 4.277 17.307 -13.856 1.00 0.00

ATOM 366 C ASP A 45 3.640 16.372 -12.836 1.00 0.00

ATOM 367 O ASP A 45 2.714 15.627 -13.156 1.00 0.00

ATOM 368 CB ASP A 45 5.559 16.683 -14.413 1.00 0.00

ATOM 369 CG ASP A 45 6.069 17.330 -15.694 1.00 0.00

ATOM 370 OD1 ASP A 45 5.335 18.085 -16.287 1.00 0.00

ATOM 371 OD2 ASP A 45 7.233 17.191 -15.983 1.00 0.00

ATOM 372 N MET A 46 4.148 16.394 -11.628 1.00 0.00

ATOM 373 CA MET A 46 3.620 15.561 -10.554 1.00 0.00

ATOM 374 C MET A 46 2.169 15.910 -10.248 1.00 0.00

ATOM 375 O MET A 46 1.323 15.027 -10.111 1.00 0.00

ATOM 376 CB MET A 46 4.477 15.714 -9.298 1.00 0.00

ATOM 377 CG MET A 46 5.809 14.980 -9.351 1.00 0.00

ATOM 378 SD MET A 46 5.615 13.187 -9.385 1.00 0.00

ATOM 379 CE MET A 46 4.848 12.900 -7.793 1.00 0.00

ATOM 380 N GLU A 47 1.887 17.205 -10.143 1.00 0.00

ATOM 381 CA GLU A 47 0.537 17.675 -9.857 1.00 0.00

ATOM 382 C GLU A 47 -0.425 17.297 -10.976 1.00 0.00

ATOM 383 O GLU A 47 -1.552 16.869 -10.722 1.00 0.00

ATOM 384 CB GLU A 47 0.529 19.191 -9.647 1.00 0.00

ATOM 385 CG GLU A 47 -0.821 19.762 -9.238 1.00 0.00

ATOM 386 CD GLU A 47 -0.713 21.219 -8.884 1.00 0.00

ATOM 387 OE1 GLU A 47 0.379 21.734 -8.894 1.00 0.00

ATOM 388 OE2 GLU A 47 -1.732 21.845 -8.712 1.00 0.00

ATOM 389 N ASP A 48 0.025 17.457 -12.216 1.00 0.00

ATOM 390 CA ASP A 48 -0.791 17.119 -13.376 1.00 0.00

ATOM 391 C ASP A 48 -1.159 15.641 -13.380 1.00 0.00

ATOM 392 O ASP A 48 -2.313 15.279 -13.604 1.00 0.00

ATOM 393 CB ASP A 48 -0.059 17.481 -14.670 1.00 0.00

ATOM 394 CG ASP A 48 0.001 18.975 -14.962 1.00 0.00

ATOM 395 OD1 ASP A 48 -0.707 19.714 -14.321 1.00 0.00

ATOM 396 OD2 ASP A 48 0.861 19.380 -15.708 1.00 0.00

ATOM 397 N LEU A 49 -0.169 14.789 -13.129 1.00 0.00

ATOM 398 CA LEU A 49 -0.389 13.349 -13.098 1.00 0.00

ATOM 399 C LEU A 49 -1.360 12.962 -11.990 1.00 0.00

ATOM 400 O LEU A 49 -2.242 12.127 -12.187 1.00 0.00

ATOM 401 CB LEU A 49 0.946 12.614 -12.919 1.00 0.00

ATOM 402 CG LEU A 49 1.887 12.665 -14.129 1.00 0.00

ATOM 403 CD1 LEU A 49 3.234 12.053 -13.768 1.00 0.00

ATOM 404 CD2 LEU A 49 1.254 11.926 -15.298 1.00 0.00

ATOM 405 N ALA A 50 -1.192 13.576 -10.824 1.00 0.00

ATOM 406 CA ALA A 50 -2.057 13.302 -9.682 1.00 0.00

ATOM 407 C ALA A 50 -3.507 13.650 -9.995 1.00 0.00

ATOM 408 O ALA A 50 -4.419 12.885 -9.680 1.00 0.00

ATOM 409 CB ALA A 50 -1.577 14.068 -8.458 1.00 0.00

ATOM 410 N LYS A 51 -3.713 14.806 -10.615 1.00 0.00

ATOM 411 CA LYS A 51 -5.054 15.264 -10.957 1.00 0.00

ATOM 412 C LYS A 51 -5.682 14.379 -12.025 1.00 0.00

ATOM 413 O LYS A 51 -6.868 14.053 -11.957 1.00 0.00

ATOM 414 CB LYS A 51 -5.019 16.717 -11.432 1.00 0.00

ATOM 415 CG LYS A 51 -4.766 17.735 -10.327 1.00 0.00

ATOM 416 CD LYS A 51 -4.720 19.152 -10.880 1.00 0.00

ATOM 417 CE LYS A 51 -4.531 20.174 -9.769 1.00 0.00

ATOM 418 NZ LYS A 51 -4.396 21.557 -10.302 1.00 0.00

ATOM 419 N LYS A 52 -4.881 13.991 -13.012 1.00 0.00

ATOM 420 CA LYS A 52 -5.360 13.147 -14.101 1.00 0.00

ATOM 421 C LYS A 52 -5.814 11.788 -13.585 1.00 0.00

ATOM 422 O LYS A 52 -6.852 11.271 -14.001 1.00 0.00

ATOM 423 CB LYS A 52 -4.270 12.971 -15.160 1.00 0.00

ATOM 424 CG LYS A 52 -4.018 14.206 -16.014 1.00 0.00

ATOM 425 CD LYS A 52 -2.877 13.977 -16.994 1.00 0.00

ATOM 426 CE LYS A 52 -2.599 15.223 -17.823 1.00 0.00

ATOM 427 NZ LYS A 52 -1.485 15.013 -18.787 1.00 0.00

ATOM 428 N THR A 53 -5.032 11.213 -12.678 1.00 0.00

ATOM 429 CA THR A 53 -5.355 9.912 -12.103 1.00 0.00

ATOM 430 C THR A 53 -6.563 10.002 -11.179 1.00 0.00

ATOM 431 O THR A 53 -7.449 9.149 -11.215 1.00 0.00

ATOM 432 CB THR A 53 -4.165 9.329 -11.319 1.00 0.00

ATOM 433 OG1 THR A 53 -3.058 9.125 -12.207 1.00 0.00

ATOM 434 CG2 THR A 53 -4.546 8.005 -10.676 1.00 0.00

ATOM 435 N GLY A 54 -6.593 11.042 -10.352 1.00 0.00

ATOM 436 CA GLY A 54 -7.691 11.244 -9.415 1.00 0.00

ATOM 437 C GLY A 54 -7.255 10.959 -7.984 1.00 0.00

ATOM 438 O GLY A 54 -8.024 10.428 -7.184 1.00 0.00

ATOM 439 N VAL A 55 -6.013 11.312 -7.668 1.00 0.00

ATOM 440 CA VAL A 55 -5.467 11.084 -6.336 1.00 0.00

ATOM 441 C VAL A 55 -5.067 12.396 -5.672 1.00 0.00

ATOM 442 O VAL A 55 -4.957 13.429 -6.333 1.00 0.00

ATOM 443 CB VAL A 55 -4.245 10.148 -6.379 1.00 0.00

ATOM 444 CG1 VAL A 55 -4.635 8.788 -6.937 1.00 0.00

ATOM 445 CG2 VAL A 55 -3.131 10.765 -7.211 1.00 0.00

ATOM 446 N GLN A 56 -4.851 12.348 -4.362 1.00 0.00

ATOM 447 CA GLN A 56 -4.472 13.535 -3.604 1.00 0.00

ATOM 448 C GLN A 56 -2.963 13.740 -3.621 1.00 0.00

ATOM 449 O GLN A 56 -2.196 12.797 -3.428 1.00 0.00

ATOM 450 CB GLN A 56 -4.963 13.427 -2.158 1.00 0.00

ATOM 451 CG GLN A 56 -4.503 14.560 -1.258 1.00 0.00

ATOM 452 CD GLN A 56 -5.130 15.889 -1.634 1.00 0.00

ATOM 453 OE1 GLN A 56 -6.357 16.020 -1.685 1.00 0.00

ATOM 454 NE2 GLN A 56 -4.292 16.883 -1.903 1.00 0.00

ATOM 455 N ILE A 57 -2.542 14.979 -3.852 1.00 0.00

ATOM 456 CA ILE A 57 -1.123 15.314 -3.876 1.00 0.00

ATOM 457 C ILE A 57 -0.727 16.121 -2.647 1.00 0.00

ATOM 458 O ILE A 57 -1.460 17.009 -2.212 1.00 0.00

ATOM 459 CB ILE A 57 -0.751 16.109 -5.141 1.00 0.00

ATOM 460 CG1 ILE A 57 0.685 16.630 -5.041 1.00 0.00

ATOM 461 CG2 ILE A 57 -1.725 17.258 -5.353 1.00 0.00

ATOM 462 CD1 ILE A 57 1.229 17.172 -6.343 1.00 0.00

ATOM 463 N GLN A 58 0.438 15.808 -2.090 1.00 0.00

ATOM 464 CA GLN A 58 0.964 16.545 -0.947 1.00 0.00

ATOM 465 C GLN A 58 2.369 17.063 -1.225 1.00 0.00

ATOM 466 O GLN A 58 3.316 16.284 -1.343 1.00 0.00

ATOM 467 CB GLN A 58 0.977 15.659 0.302 1.00 0.00

ATOM 468 CG GLN A 58 1.689 16.275 1.494 1.00 0.00

ATOM 469 CD GLN A 58 0.975 17.504 2.023 1.00 0.00

ATOM 470 OE1 GLN A 58 -0.159 17.421 2.503 1.00 0.00

ATOM 471 NE2 GLN A 58 1.633 18.654 1.935 1.00 0.00

ATOM 472 N LYS A 59 2.500 18.381 -1.330 1.00 0.00

ATOM 473 CA LYS A 59 3.788 19.004 -1.613 1.00 0.00

ATOM 474 C LYS A 59 4.553 19.292 -0.328 1.00 0.00

ATOM 475 O LYS A 59 3.973 19.721 0.669 1.00 0.00

ATOM 476 CB LYS A 59 3.595 20.294 -2.411 1.00 0.00

ATOM 477 CG LYS A 59 2.886 20.109 -3.746 1.00 0.00

ATOM 478 CD LYS A 59 2.769 21.426 -4.498 1.00 0.00

ATOM 479 CE LYS A 59 2.016 21.251 -5.808 1.00 0.00

ATOM 480 NZ LYS A 59 1.960 22.515 -6.591 1.00 0.00

ATOM 481 N GLN A 60 5.861 19.056 -0.359 1.00 0.00

ATOM 482 CA GLN A 60 6.715 19.322 0.792 1.00 0.00

ATOM 483 C GLN A 60 8.079 19.843 0.358 1.00 0.00

ATOM 484 O GLN A 60 8.568 19.506 -0.721 1.00 0.00

ATOM 485 CB GLN A 60 6.889 18.055 1.633 1.00 0.00

ATOM 486 CG GLN A 60 5.616 17.578 2.311 1.00 0.00

ATOM 487 CD GLN A 60 5.861 16.406 3.243 1.00 0.00

ATOM 488 OE1 GLN A 60 7.007 16.016 3.485 1.00 0.00

ATOM 489 NE2 GLN A 60 4.784 15.834 3.769 1.00 0.00

ATOM 490 N TRP A 61 8.689 20.667 1.203 1.00 0.00

ATOM 491 CA TRP A 61 9.990 21.252 0.899 1.00 0.00

ATOM 492 C TRP A 61 10.979 21.014 2.034 1.00 0.00

ATOM 493 O TRP A 61 10.673 21.265 3.199 1.00 0.00

ATOM 494 CB TRP A 61 9.850 22.751 0.629 1.00 0.00

ATOM 495 CG TRP A 61 11.116 23.392 0.146 1.00 0.00

ATOM 496 CD1 TRP A 61 11.972 24.159 0.879 1.00 0.00

ATOM 497 CD2 TRP A 61 11.668 23.322 -1.174 1.00 0.00

ATOM 498 NE1 TRP A 61 13.022 24.573 0.098 1.00 0.00

ATOM 499 CE2 TRP A 61 12.859 24.070 -1.168 1.00 0.00

ATOM 500 CE3 TRP A 61 11.269 22.697 -2.362 1.00 0.00

ATOM 501 CZ2 TRP A 61 13.652 24.213 -2.295 1.00 0.00

ATOM 502 CZ3 TRP A 61 12.065 22.840 -3.492 1.00 0.00

ATOM 503 CH2 TRP A 61 13.223 23.576 -3.460 1.00 0.00

ATOM 504 N GLN A 62 12.165 20.531 1.685 1.00 0.00

ATOM 505 CA GLN A 62 13.204 20.264 2.673 1.00 0.00

ATOM 506 C GLN A 62 14.538 20.860 2.244 1.00 0.00

ATOM 507 O GLN A 62 15.344 20.197 1.590 1.00 0.00

ATOM 508 CB GLN A 62 13.360 18.757 2.894 1.00 0.00

ATOM 509 CG GLN A 62 12.125 18.081 3.465 1.00 0.00

ATOM 510 CD GLN A 62 11.841 18.507 4.893 1.00 0.00

ATOM 511 OE1 GLN A 62 12.733 18.504 5.746 1.00 0.00

ATOM 512 NE2 GLN A 62 10.593 18.873 5.163 1.00 0.00

ATOM 513 N GLY A 63 14.766 22.115 2.615 1.00 0.00

ATOM 514 CA GLY A 63 16.007 22.802 2.275 1.00 0.00

ATOM 515 C GLY A 63 16.065 23.130 0.788 1.00 0.00

ATOM 516 O GLY A 63 15.521 24.140 0.344 1.00 0.00

ATOM 517 N ASN A 64 16.729 22.268 0.023 1.00 0.00

ATOM 518 CA ASN A 64 16.811 22.435 -1.422 1.00 0.00

ATOM 519 C ASN A 64 16.206 21.240 -2.149 1.00 0.00

ATOM 520 O ASN A 64 16.288 21.139 -3.373 1.00 0.00

ATOM 521 CB ASN A 64 18.242 22.656 -1.876 1.00 0.00

ATOM 522 CG ASN A 64 18.852 23.929 -1.357 1.00 0.00

ATOM 523 OD1 ASN A 64 18.460 25.034 -1.749 1.00 0.00

ATOM 524 ND2 ASN A 64 19.863 23.778 -0.540 1.00 0.00

ATOM 525 N LYS A 65 15.600 20.336 -1.388 1.00 0.00

ATOM 526 CA LYS A 65 15.000 19.134 -1.955 1.00 0.00

ATOM 527 C LYS A 65 13.481 19.240 -1.990 1.00 0.00

ATOM 528 O LYS A 65 12.856 19.666 -1.019 1.00 0.00

ATOM 529 CB LYS A 65 15.423 17.898 -1.159 1.00 0.00

ATOM 530 CG LYS A 65 14.834 16.590 -1.669 1.00 0.00

ATOM 531 CD LYS A 65 15.475 15.391 -0.986 1.00 0.00

ATOM 532 CE LYS A 65 14.998 15.253 0.453 1.00 0.00

ATOM 533 NZ LYS A 65 15.562 14.045 1.113 1.00 0.00

ATOM 534 N LEU A 66 12.892 18.853 -3.116 1.00 0.00

ATOM 535 CA LEU A 66 11.441 18.852 -3.262 1.00 0.00

ATOM 536 C LEU A 66 10.860 17.470 -2.992 1.00 0.00

ATOM 537 O LEU A 66 11.323 16.473 -3.547 1.00 0.00

ATOM 538 CB LEU A 66 11.050 19.331 -4.666 1.00 0.00

ATOM 539 CG LEU A 66 9.545 19.330 -4.960 1.00 0.00

ATOM 540 CD1 LEU A 66 8.849 20.394 -4.123 1.00 0.00

ATOM 541 CD2 LEU A 66 9.316 19.577 -6.444 1.00 0.00

ATOM 542 N ARG A 67 9.844 17.417 -2.139 1.00 0.00

ATOM 543 CA ARG A 67 9.207 16.154 -1.783 1.00 0.00

ATOM 544 C ARG A 67 7.731 16.157 -2.156 1.00 0.00

ATOM 545 O ARG A 67 6.935 16.894 -1.575 1.00 0.00

ATOM 546 CB ARG A 67 9.410 15.803 -0.316 1.00 0.00

ATOM 547 CG ARG A 67 10.845 15.490 0.074 1.00 0.00

ATOM 548 CD ARG A 67 11.040 15.211 1.520 1.00 0.00

ATOM 549 NE ARG A 67 10.473 13.951 1.973 1.00 0.00

ATOM 550 CZ ARG A 67 10.459 13.534 3.254 1.00 0.00

ATOM 551 NH1 ARG A 67 11.013 14.253 4.206 1.00 0.00

ATOM 552 NH2 ARG A 67 9.896 12.370 3.526 1.00 0.00

ATOM 553 N ILE A 68 7.371 15.328 -3.130 1.00 0.00

ATOM 554 CA ILE A 68 5.986 15.220 -3.573 1.00 0.00

ATOM 555 C ILE A 68 5.422 13.834 -3.286 1.00 0.00

ATOM 556 O ILE A 68 5.885 12.838 -3.843 1.00 0.00

ATOM 557 CB ILE A 68 5.848 15.519 -5.077 1.00 0.00

ATOM 558 CG1 ILE A 68 6.499 16.862 -5.416 1.00 0.00

ATOM 559 CG2 ILE A 68 4.383 15.515 -5.488 1.00 0.00

ATOM 560 CD1 ILE A 68 5.850 18.045 -4.735 1.00 0.00

ATOM 561 N ARG A 69 4.420 13.777 -2.416 1.00 0.00

ATOM 562 CA ARG A 69 3.800 12.511 -2.046 1.00 0.00

ATOM 563 C ARG A 69 2.397 12.393 -2.628 1.00 0.00

ATOM 564 O ARG A 69 1.545 13.251 -2.398 1.00 0.00

ATOM 565 CB ARG A 69 3.797 12.291 -0.540 1.00 0.00

ATOM 566 CG ARG A 69 3.208 10.965 -0.088 1.00 0.00

ATOM 567 CD ARG A 69 3.123 10.803 1.386 1.00 0.00

ATOM 568 NE ARG A 69 4.402 10.877 2.075 1.00 0.00

ATOM 569 CZ ARG A 69 4.546 11.007 3.408 1.00 0.00

ATOM 570 NH1 ARG A 69 3.498 11.116 4.195 1.00 0.00

ATOM 571 NH2 ARG A 69 5.771 11.047 3.903 1.00 0.00

ATOM 572 N LEU A 70 2.164 11.326 -3.383 1.00 0.00

ATOM 573 CA LEU A 70 0.852 11.072 -3.967 1.00 0.00

ATOM 574 C LEU A 70 0.160 9.900 -3.283 1.00 0.00

ATOM 575 O LEU A 70 0.791 8.888 -2.976 1.00 0.00

ATOM 576 CB LEU A 70 0.983 10.809 -5.472 1.00 0.00

ATOM 577 CG LEU A 70 1.673 11.921 -6.272 1.00 0.00

ATOM 578 CD1 LEU A 70 1.616 11.606 -7.760 1.00 0.00

ATOM 579 CD2 LEU A 70 1.000 13.253 -5.976 1.00 0.00

ATOM 580 N LYS A 71 -1.138 10.044 -3.043 1.00 0.00

ATOM 581 CA LYS A 71 -1.916 9.001 -2.383 1.00 0.00

ATOM 582 C LYS A 71 -3.374 9.038 -2.825 1.00 0.00

ATOM 583 O LYS A 71 -3.984 10.105 -2.894 1.00 0.00

ATOM 584 CB LYS A 71 -1.824 9.144 -0.863 1.00 0.00

ATOM 585 CG LYS A 71 -2.486 8.016 -0.084 1.00 0.00

ATOM 586 CD LYS A 71 -2.270 8.177 1.413 1.00 0.00

ATOM 587 CE LYS A 71 -2.895 7.028 2.190 1.00 0.00

ATOM 588 NZ LYS A 71 -2.683 7.167 3.657 1.00 0.00

ATOM 589 N GLY A 72 -3.926 7.867 -3.122 1.00 0.00

ATOM 590 CA GLY A 72 -5.315 7.763 -3.555 1.00 0.00

ATOM 591 C GLY A 72 -6.208 7.269 -2.424 1.00 0.00

ATOM 592 O GLY A 72 -5.765 6.519 -1.553 1.00 0.00

ATOM 593 OXT GLY A 72 -7.358 7.610 -2.375 1.00 0.00

TER

ENDMDL

**PDB ID 2LVB**

ATOM 1 N GLY A 1 0.000 0.000 0.000 1.00 0.00

ATOM 2 CA GLY A 1 1.458 0.000 0.000 1.00 0.00

ATOM 3 C GLY A 1 2.009 1.420 0.000 1.00 0.00

ATOM 4 O GLY A 1 2.550 1.886 -1.003 1.00 0.00

ATOM 5 N LYS A 2 1.868 2.104 1.130 1.00 0.00

ATOM 6 CA LYS A 2 2.311 3.488 1.249 1.00 0.00

ATOM 7 C LYS A 2 2.767 3.800 2.668 1.00 0.00

ATOM 8 O LYS A 2 1.992 3.691 3.618 1.00 0.00

ATOM 9 CB LYS A 2 1.193 4.446 0.833 1.00 0.00

ATOM 10 CG LYS A 2 1.638 5.892 0.656 1.00 0.00

ATOM 11 CD LYS A 2 0.500 6.762 0.143 1.00 0.00

ATOM 12 CE LYS A 2 0.248 6.527 -1.339 1.00 0.00

ATOM 13 NZ LYS A 2 -0.788 7.449 -1.880 1.00 0.00

ATOM 14 N VAL A 3 4.030 4.190 2.807 1.00 0.00

ATOM 15 CA VAL A 3 4.597 4.502 4.113 1.00 0.00

ATOM 16 C VAL A 3 5.011 5.966 4.199 1.00 0.00

ATOM 17 O VAL A 3 5.599 6.510 3.265 1.00 0.00

ATOM 18 CB VAL A 3 5.816 3.615 4.426 1.00 0.00

ATOM 19 CG1 VAL A 3 6.889 3.789 3.362 1.00 0.00

ATOM 20 CG2 VAL A 3 6.375 3.944 5.802 1.00 0.00

ATOM 21 N LEU A 4 4.699 6.598 5.325 1.00 0.00

ATOM 22 CA LEU A 4 5.040 8.000 5.536 1.00 0.00

ATOM 23 C LEU A 4 6.090 8.153 6.629 1.00 0.00

ATOM 24 O LEU A 4 5.886 7.722 7.764 1.00 0.00

ATOM 25 CB LEU A 4 3.782 8.804 5.887 1.00 0.00

ATOM 26 CG LEU A 4 4.036 10.247 6.344 1.00 0.00

ATOM 27 CD1 LEU A 4 4.635 11.058 5.203 1.00 0.00

ATOM 28 CD2 LEU A 4 2.731 10.867 6.819 1.00 0.00

ATOM 29 N LEU A 5 7.214 8.770 6.280 1.00 0.00

ATOM 30 CA LEU A 5 8.238 9.107 7.261 1.00 0.00

ATOM 31 C LEU A 5 7.990 10.483 7.866 1.00 0.00

ATOM 32 O LEU A 5 8.103 11.501 7.184 1.00 0.00

ATOM 33 CB LEU A 5 9.629 9.051 6.616 1.00 0.00

ATOM 34 CG LEU A 5 10.793 9.431 7.540 1.00 0.00

ATOM 35 CD1 LEU A 5 10.897 8.435 8.687 1.00 0.00

ATOM 36 CD2 LEU A 5 12.086 9.469 6.739 1.00 0.00

ATOM 37 N VAL A 6 7.652 10.507 9.151 1.00 0.00

ATOM 38 CA VAL A 6 7.434 11.761 9.862 1.00 0.00

ATOM 39 C VAL A 6 8.628 12.111 10.740 1.00 0.00

ATOM 40 O VAL A 6 8.945 11.394 11.690 1.00 0.00

ATOM 41 CB VAL A 6 6.167 11.701 10.737 1.00 0.00

ATOM 42 CG1 VAL A 6 5.986 13.004 11.501 1.00 0.00

ATOM 43 CG2 VAL A 6 4.942 11.412 9.882 1.00 0.00

ATOM 44 N ILE A 7 9.289 13.217 10.417 1.00 0.00

ATOM 45 CA ILE A 7 10.509 13.612 11.112 1.00 0.00

ATOM 46 C ILE A 7 10.277 14.845 11.975 1.00 0.00

ATOM 47 O ILE A 7 9.746 15.852 11.506 1.00 0.00

ATOM 48 CB ILE A 7 11.655 13.897 10.124 1.00 0.00

ATOM 49 CG1 ILE A 7 11.889 12.688 9.214 1.00 0.00

ATOM 50 CG2 ILE A 7 12.928 14.255 10.876 1.00 0.00

ATOM 51 CD1 ILE A 7 12.837 12.960 8.069 1.00 0.00

ATOM 52 N SER A 8 10.677 14.760 13.239 1.00 0.00

ATOM 53 CA SER A 8 10.626 15.907 14.138 1.00 0.00

ATOM 54 C SER A 8 11.822 15.923 15.080 1.00 0.00

ATOM 55 O SER A 8 12.786 15.182 14.887 1.00 0.00

ATOM 56 CB SER A 8 9.331 15.894 14.927 1.00 0.00

ATOM 57 OG SER A 8 9.072 17.130 15.534 1.00 0.00

ATOM 58 N THR A 9 11.754 16.771 16.101 1.00 0.00

ATOM 59 CA THR A 9 12.844 16.906 17.059 1.00 0.00

ATOM 60 C THR A 9 12.368 16.630 18.479 1.00 0.00

ATOM 61 O THR A 9 13.166 16.319 19.362 1.00 0.00

ATOM 62 CB THR A 9 13.474 18.311 17.004 1.00 0.00

ATOM 63 OG1 THR A 9 12.493 19.292 17.365 1.00 0.00

ATOM 64 CG2 THR A 9 13.993 18.609 15.606 1.00 0.00

ATOM 65 N ASP A 10 11.061 16.745 18.692 1.00 0.00

ATOM 66 CA ASP A 10 10.482 16.560 20.017 1.00 0.00

ATOM 67 C ASP A 10 9.655 15.282 20.083 1.00 0.00

ATOM 68 O ASP A 10 8.678 15.125 19.351 1.00 0.00

ATOM 69 CB ASP A 10 9.619 17.765 20.398 1.00 0.00

ATOM 70 CG ASP A 10 9.080 17.728 21.822 1.00 0.00

ATOM 71 OD1 ASP A 10 9.350 16.775 22.514 1.00 0.00

ATOM 72 OD2 ASP A 10 8.539 18.716 22.257 1.00 0.00

ATOM 73 N THR A 11 10.052 14.371 20.965 1.00 0.00

ATOM 74 CA THR A 11 9.325 13.122 21.156 1.00 0.00

ATOM 75 C THR A 11 7.891 13.381 21.597 1.00 0.00

ATOM 76 O THR A 11 6.984 12.613 21.276 1.00 0.00

ATOM 77 CB THR A 11 10.016 12.219 22.195 1.00 0.00

ATOM 78 OG1 THR A 11 11.325 11.865 21.729 1.00 0.00

ATOM 79 CG2 THR A 11 9.205 10.954 22.425 1.00 0.00

ATOM 80 N ASN A 12 7.691 14.468 22.335 1.00 0.00

ATOM 81 CA ASN A 12 6.361 14.847 22.796 1.00 0.00

ATOM 82 C ASN A 12 5.456 15.212 21.626 1.00 0.00

ATOM 83 O ASN A 12 4.270 14.883 21.621 1.00 0.00

ATOM 84 CB ASN A 12 6.424 15.994 23.788 1.00 0.00

ATOM 85 CG ASN A 12 6.952 15.597 25.139 1.00 0.00

ATOM 86 OD1 ASN A 12 6.928 14.420 25.516 1.00 0.00

ATOM 87 ND2 ASN A 12 7.352 16.583 25.901 1.00 0.00

ATOM 88 N ILE A 13 6.022 15.894 20.637 1.00 0.00

ATOM 89 CA ILE A 13 5.300 16.204 19.409 1.00 0.00

ATOM 90 C ILE A 13 4.976 14.939 18.625 1.00 0.00

ATOM 91 O ILE A 13 3.881 14.797 18.081 1.00 0.00

ATOM 92 CB ILE A 13 6.101 17.163 18.509 1.00 0.00

ATOM 93 CG1 ILE A 13 6.161 18.558 19.136 1.00 0.00

ATOM 94 CG2 ILE A 13 5.486 17.227 17.119 1.00 0.00

ATOM 95 CD1 ILE A 13 7.106 19.504 18.431 1.00 0.00

ATOM 96 N ILE A 14 5.935 14.020 18.572 1.00 0.00

ATOM 97 CA ILE A 14 5.724 12.729 17.929 1.00 0.00

ATOM 98 C ILE A 14 4.553 11.985 18.558 1.00 0.00

ATOM 99 O ILE A 14 3.676 11.480 17.857 1.00 0.00

ATOM 100 CB ILE A 14 6.983 11.847 18.006 1.00 0.00

ATOM 101 CG1 ILE A 14 8.079 12.399 17.092 1.00 0.00

ATOM 102 CG2 ILE A 14 6.649 10.410 17.634 1.00 0.00

ATOM 103 CD1 ILE A 14 9.427 11.744 17.291 1.00 0.00

ATOM 104 N SER A 15 4.545 11.920 19.885 1.00 0.00

ATOM 105 CA SER A 15 3.483 11.234 20.612 1.00 0.00

ATOM 106 C SER A 15 2.135 11.904 20.379 1.00 0.00

ATOM 107 O SER A 15 1.116 11.232 20.220 1.00 0.00

ATOM 108 CB SER A 15 3.805 11.196 22.094 1.00 0.00

ATOM 109 OG SER A 15 4.932 10.410 22.367 1.00 0.00

ATOM 110 N SER A 16 2.136 13.233 20.361 1.00 0.00

ATOM 111 CA SER A 16 0.923 13.996 20.090 1.00 0.00

ATOM 112 C SER A 16 0.300 13.588 18.762 1.00 0.00

ATOM 113 O SER A 16 -0.889 13.278 18.692 1.00 0.00

ATOM 114 CB SER A 16 1.228 15.481 20.095 1.00 0.00

ATOM 115 OG SER A 16 1.597 15.938 21.368 1.00 0.00

ATOM 116 N VAL A 17 1.110 13.589 17.709 1.00 0.00

ATOM 117 CA VAL A 17 0.619 13.316 16.363 1.00 0.00

ATOM 118 C VAL A 17 0.220 11.855 16.209 1.00 0.00

ATOM 119 O VAL A 17 -0.723 11.532 15.486 1.00 0.00

ATOM 120 CB VAL A 17 1.672 13.667 15.295 1.00 0.00

ATOM 121 CG1 VAL A 17 1.185 13.257 13.913 1.00 0.00

ATOM 122 CG2 VAL A 17 1.990 15.154 15.328 1.00 0.00

ATOM 123 N GLN A 18 0.943 10.973 16.891 1.00 0.00

ATOM 124 CA GLN A 18 0.589 9.559 16.932 1.00 0.00

ATOM 125 C GLN A 18 -0.809 9.357 17.501 1.00 0.00

ATOM 126 O GLN A 18 -1.596 8.570 16.975 1.00 0.00

ATOM 127 CB GLN A 18 1.606 8.777 17.767 1.00 0.00

ATOM 128 CG GLN A 18 2.900 8.460 17.037 1.00 0.00

ATOM 129 CD GLN A 18 3.922 7.790 17.936 1.00 0.00

ATOM 130 OE1 GLN A 18 3.716 7.661 19.146 1.00 0.00

ATOM 131 NE2 GLN A 18 5.035 7.363 17.349 1.00 0.00

ATOM 132 N GLU A 19 -1.112 10.072 18.579 1.00 0.00

ATOM 133 CA GLU A 19 -2.425 9.990 19.208 1.00 0.00

ATOM 134 C GLU A 19 -3.514 10.514 18.282 1.00 0.00

ATOM 135 O GLU A 19 -4.591 9.926 18.178 1.00 0.00

ATOM 136 CB GLU A 19 -2.437 10.767 20.527 1.00 0.00

ATOM 137 CG GLU A 19 -1.658 10.104 21.654 1.00 0.00

ATOM 138 CD GLU A 19 -2.202 8.737 21.963 1.00 0.00

ATOM 139 OE1 GLU A 19 -3.384 8.624 22.181 1.00 0.00

ATOM 140 OE2 GLU A 19 -1.458 7.789 21.870 1.00 0.00

ATOM 141 N ARG A 20 -3.228 11.624 17.610 1.00 0.00

ATOM 142 CA ARG A 20 -4.168 12.209 16.660 1.00 0.00

ATOM 143 C ARG A 20 -4.445 11.258 15.503 1.00 0.00

ATOM 144 O ARG A 20 -5.594 11.070 15.104 1.00 0.00

ATOM 145 CB ARG A 20 -3.708 13.572 16.163 1.00 0.00

ATOM 146 CG ARG A 20 -3.678 14.662 17.223 1.00 0.00

ATOM 147 CD ARG A 20 -5.016 15.065 17.725 1.00 0.00

ATOM 148 NE ARG A 20 -5.030 16.319 18.461 1.00 0.00

ATOM 149 CZ ARG A 20 -6.060 16.753 19.214 1.00 0.00

ATOM 150 NH1 ARG A 20 -7.172 16.059 19.304 1.00 0.00

ATOM 151 NH2 ARG A 20 -5.931 17.908 19.842 1.00 0.00

ATOM 152 N ALA A 21 -3.386 10.660 14.969 1.00 0.00

ATOM 153 CA ALA A 21 -3.510 9.752 13.835 1.00 0.00

ATOM 154 C ALA A 21 -4.272 8.490 14.220 1.00 0.00

ATOM 155 O ALA A 21 -5.093 7.990 13.451 1.00 0.00

ATOM 156 CB ALA A 21 -2.135 9.398 13.286 1.00 0.00

ATOM 157 N LYS A 22 -3.995 7.980 15.415 1.00 0.00

ATOM 158 CA LYS A 22 -4.660 6.779 15.908 1.00 0.00

ATOM 159 C LYS A 22 -6.151 7.018 16.107 1.00 0.00

ATOM 160 O LYS A 22 -6.977 6.182 15.742 1.00 0.00

ATOM 161 CB LYS A 22 -4.022 6.313 17.218 1.00 0.00

ATOM 162 CG LYS A 22 -4.633 5.045 17.798 1.00 0.00

ATOM 163 CD LYS A 22 -3.904 4.608 19.060 1.00 0.00

ATOM 164 CE LYS A 22 -4.536 3.361 19.662 1.00 0.00

ATOM 165 NZ LYS A 22 -3.849 2.936 20.911 1.00 0.00

ATOM 166 N HIS A 23 -6.489 8.164 16.689 1.00 0.00

ATOM 167 CA HIS A 23 -7.876 8.484 17.004 1.00 0.00

ATOM 168 C HIS A 23 -8.684 8.737 15.737 1.00 0.00

ATOM 169 O HIS A 23 -9.833 8.310 15.627 1.00 0.00

ATOM 170 CB HIS A 23 -7.954 9.704 17.927 1.00 0.00

ATOM 171 CG HIS A 23 -7.399 9.459 19.295 1.00 0.00

ATOM 172 ND1 HIS A 23 -7.141 10.480 20.187 1.00 0.00

ATOM 173 CD2 HIS A 23 -7.051 8.312 19.925 1.00 0.00

ATOM 174 CE1 HIS A 23 -6.659 9.969 21.307 1.00 0.00

ATOM 175 NE2 HIS A 23 -6.594 8.658 21.173 1.00 0.00

ATOM 176 N ASN A 24 -8.076 9.432 14.783 1.00 0.00

ATOM 177 CA ASN A 24 -8.752 9.779 13.538 1.00 0.00

ATOM 178 C ASN A 24 -8.872 8.570 12.621 1.00 0.00

ATOM 179 O ASN A 24 -9.906 8.359 11.988 1.00 0.00

ATOM 180 CB ASN A 24 -8.045 10.915 12.820 1.00 0.00

ATOM 181 CG ASN A 24 -8.225 12.253 13.481 1.00 0.00

ATOM 182 OD1 ASN A 24 -9.175 12.470 14.242 1.00 0.00

ATOM 183 ND2 ASN A 24 -7.366 13.176 13.130 1.00 0.00

ATOM 184 N TYR A 25 -7.807 7.777 12.553 1.00 0.00

ATOM 185 CA TYR A 25 -7.763 6.628 11.656 1.00 0.00

ATOM 186 C TYR A 25 -7.175 5.408 12.353 1.00 0.00

ATOM 187 O TYR A 25 -6.024 5.041 12.114 1.00 0.00

ATOM 188 CB TYR A 25 -6.951 6.958 10.402 1.00 0.00

ATOM 189 CG TYR A 25 -7.389 8.226 9.704 1.00 0.00

ATOM 190 CD1 TYR A 25 -6.702 9.417 9.891 1.00 0.00

ATOM 191 CD2 TYR A 25 -8.488 8.228 8.857 1.00 0.00

ATOM 192 CE1 TYR A 25 -7.099 10.578 9.256 1.00 0.00

ATOM 193 CE2 TYR A 25 -8.893 9.383 8.217 1.00 0.00

ATOM 194 CZ TYR A 25 -8.196 10.557 8.419 1.00 0.00

ATOM 195 OH TYR A 25 -8.595 11.710 7.782 1.00 0.00

ATOM 196 N PRO A 26 -7.970 4.783 13.214 1.00 0.00

ATOM 197 CA PRO A 26 -7.518 3.622 13.971 1.00 0.00

ATOM 198 C PRO A 26 -7.039 2.513 13.043 1.00 0.00

ATOM 199 O PRO A 26 -6.158 1.730 13.399 1.00 0.00

ATOM 200 CB PRO A 26 -8.748 3.203 14.783 1.00 0.00

ATOM 201 CG PRO A 26 -9.550 4.452 14.913 1.00 0.00

ATOM 202 CD PRO A 26 -9.349 5.193 13.618 1.00 0.00

ATOM 203 N GLY A 27 -7.623 2.451 11.851 1.00 0.00

ATOM 204 CA GLY A 27 -7.343 1.368 10.916 1.00 0.00

ATOM 205 C GLY A 27 -5.915 1.447 10.394 1.00 0.00

ATOM 206 O GLY A 27 -5.324 0.435 10.018 1.00 0.00

ATOM 207 N ARG A 28 -5.364 2.657 10.372 1.00 0.00

ATOM 208 CA ARG A 28 -4.033 2.882 9.822 1.00 0.00

ATOM 209 C ARG A 28 -2.951 2.497 10.823 1.00 0.00

ATOM 210 O ARG A 28 -3.151 2.587 12.034 1.00 0.00

ATOM 211 CB ARG A 28 -3.851 4.310 9.327 1.00 0.00

ATOM 212 CG ARG A 28 -5.036 4.879 8.565 1.00 0.00

ATOM 213 CD ARG A 28 -5.040 4.561 7.113 1.00 0.00

ATOM 214 NE ARG A 28 -6.011 5.313 6.335 1.00 0.00

ATOM 215 CZ ARG A 28 -7.267 4.900 6.073 1.00 0.00

ATOM 216 NH1 ARG A 28 -7.696 3.730 6.492 1.00 0.00

ATOM 217 NH2 ARG A 28 -8.049 5.693 5.362 1.00 0.00

ATOM 218 N GLU A 29 -1.803 2.067 10.309 1.00 0.00

ATOM 219 CA GLU A 29 -0.724 1.569 11.154 1.00 0.00

ATOM 220 C GLU A 29 0.202 2.699 11.586 1.00 0.00

ATOM 221 O GLU A 29 0.490 3.609 10.809 1.00 0.00

ATOM 222 CB GLU A 29 0.073 0.486 10.423 1.00 0.00

ATOM 223 CG GLU A 29 -0.699 -0.802 10.174 1.00 0.00

ATOM 224 CD GLU A 29 -1.091 -1.463 11.465 1.00 0.00

ATOM 225 OE1 GLU A 29 -0.230 -1.686 12.282 1.00 0.00

ATOM 226 OE2 GLU A 29 -2.266 -1.643 11.684 1.00 0.00

ATOM 227 N ILE A 30 0.667 2.634 12.829 1.00 0.00

ATOM 228 CA ILE A 30 1.611 3.617 13.346 1.00 0.00

ATOM 229 C ILE A 30 2.825 2.939 13.970 1.00 0.00

ATOM 230 O ILE A 30 2.688 2.015 14.771 1.00 0.00

ATOM 231 CB ILE A 30 0.955 4.536 14.392 1.00 0.00

ATOM 232 CG1 ILE A 30 -0.200 5.320 13.763 1.00 0.00

ATOM 233 CG2 ILE A 30 1.984 5.484 14.988 1.00 0.00

ATOM 234 CD1 ILE A 30 -1.028 6.095 14.763 1.00 0.00

ATOM 235 N ARG A 31 4.012 3.404 13.597 1.00 0.00

ATOM 236 CA ARG A 31 5.254 2.807 14.075 1.00 0.00

ATOM 237 C ARG A 31 6.268 3.876 14.458 1.00 0.00

ATOM 238 O ARG A 31 6.122 5.043 14.094 1.00 0.00

ATOM 239 CB ARG A 31 5.837 1.818 13.076 1.00 0.00

ATOM 240 CG ARG A 31 4.954 0.618 12.774 1.00 0.00

ATOM 241 CD ARG A 31 4.805 -0.333 13.906 1.00 0.00

ATOM 242 NE ARG A 31 4.076 -1.548 13.580 1.00 0.00

ATOM 243 CZ ARG A 31 2.735 -1.672 13.629 1.00 0.00

ATOM 244 NH1 ARG A 31 1.977 -0.673 14.024 1.00 0.00

ATOM 245 NH2 ARG A 31 2.202 -2.833 13.291 1.00 0.00

ATOM 246 N THR A 32 7.297 3.472 15.194 1.00 0.00

ATOM 247 CA THR A 32 8.364 4.385 15.587 1.00 0.00

ATOM 248 C THR A 32 9.735 3.770 15.335 1.00 0.00

ATOM 249 O THR A 32 10.001 2.637 15.736 1.00 0.00

ATOM 250 CB THR A 32 8.254 4.778 17.072 1.00 0.00

ATOM 251 OG1 THR A 32 6.967 5.358 17.321 1.00 0.00

ATOM 252 CG2 THR A 32 9.338 5.780 17.440 1.00 0.00

ATOM 253 N ALA A 33 10.603 4.524 14.669 1.00 0.00

ATOM 254 CA ALA A 33 11.937 4.042 14.333 1.00 0.00

ATOM 255 C ALA A 33 13.013 4.889 15.000 1.00 0.00

ATOM 256 O ALA A 33 12.863 6.103 15.141 1.00 0.00

ATOM 257 CB ALA A 33 12.131 4.026 12.824 1.00 0.00

ATOM 258 N THR A 34 14.099 4.242 15.410 1.00 0.00

ATOM 259 CA THR A 34 15.239 4.946 15.986 1.00 0.00

ATOM 260 C THR A 34 16.511 4.677 15.193 1.00 0.00

ATOM 261 O THR A 34 17.586 5.165 15.541 1.00 0.00

ATOM 262 CB THR A 34 15.467 4.545 17.455 1.00 0.00

ATOM 263 OG1 THR A 34 15.728 3.138 17.534 1.00 0.00

ATOM 264 CG2 THR A 34 14.244 4.878 18.296 1.00 0.00

ATOM 265 N SER A 35 16.382 3.896 14.125 1.00 0.00

ATOM 266 CA SER A 35 17.519 3.569 13.274 1.00 0.00

ATOM 267 C SER A 35 17.077 3.302 11.841 1.00 0.00

ATOM 268 O SER A 35 15.883 3.197 11.559 1.00 0.00

ATOM 269 CB SER A 35 18.259 2.367 13.830 1.00 0.00

ATOM 270 OG SER A 35 17.519 1.185 13.694 1.00 0.00

ATOM 271 N SER A 36 18.045 3.194 10.938 1.00 0.00

ATOM 272 CA SER A 36 17.763 2.860 9.547 1.00 0.00

ATOM 273 C SER A 36 17.260 1.428 9.415 1.00 0.00

ATOM 274 O SER A 36 16.482 1.114 8.515 1.00 0.00

ATOM 275 CB SER A 36 19.002 3.066 8.699 1.00 0.00

ATOM 276 OG SER A 36 19.986 2.103 8.962 1.00 0.00

ATOM 277 N GLN A 37 17.710 0.563 10.318 1.00 0.00

ATOM 278 CA GLN A 37 17.243 -0.818 10.354 1.00 0.00

ATOM 279 C GLN A 37 15.794 -0.897 10.817 1.00 0.00

ATOM 280 O GLN A 37 15.007 -1.683 10.290 1.00 0.00

ATOM 281 CB GLN A 37 18.128 -1.658 11.280 1.00 0.00

ATOM 282 CG GLN A 37 17.811 -3.143 11.264 1.00 0.00

ATOM 283 CD GLN A 37 18.056 -3.773 9.905 1.00 0.00

ATOM 284 OE1 GLN A 37 19.143 -3.646 9.334 1.00 0.00

ATOM 285 NE2 GLN A 37 17.045 -4.453 9.378 1.00 0.00

ATOM 286 N ASP A 38 15.448 -0.079 11.804 1.00 0.00

ATOM 287 CA ASP A 38 14.060 0.066 12.228 1.00 0.00

ATOM 288 C ASP A 38 13.174 0.507 11.071 1.00 0.00

ATOM 289 O ASP A 38 12.104 -0.059 10.845 1.00 0.00

ATOM 290 CB ASP A 38 13.953 1.066 13.382 1.00 0.00

ATOM 291 CG ASP A 38 14.491 0.552 14.711 1.00 0.00

ATOM 292 OD1 ASP A 38 14.757 -0.622 14.808 1.00 0.00

ATOM 293 OD2 ASP A 38 14.778 1.358 15.564 1.00 0.00

ATOM 294 N ILE A 39 13.625 1.521 10.339 1.00 0.00

ATOM 295 CA ILE A 39 12.881 2.029 9.193 1.00 0.00

ATOM 296 C ILE A 39 12.695 0.951 8.134 1.00 0.00

ATOM 297 O ILE A 39 11.598 0.769 7.605 1.00 0.00

ATOM 298 CB ILE A 39 13.582 3.244 8.558 1.00 0.00

ATOM 299 CG1 ILE A 39 13.519 4.450 9.499 1.00 0.00

ATOM 300 CG2 ILE A 39 12.953 3.579 7.215 1.00 0.00

ATOM 301 CD1 ILE A 39 14.395 5.605 9.071 1.00 0.00

ATOM 302 N ARG A 40 13.773 0.236 7.829 1.00 0.00

ATOM 303 CA ARG A 40 13.732 -0.822 6.826 1.00 0.00

ATOM 304 C ARG A 40 12.730 -1.904 7.207 1.00 0.00

ATOM 305 O ARG A 40 11.934 -2.346 6.379 1.00 0.00

ATOM 306 CB ARG A 40 15.109 -1.408 6.554 1.00 0.00

ATOM 307 CG ARG A 40 16.033 -0.520 5.736 1.00 0.00

ATOM 308 CD ARG A 40 17.307 -1.167 5.333 1.00 0.00

ATOM 309 NE ARG A 40 18.175 -1.530 6.442 1.00 0.00

ATOM 310 CZ ARG A 40 19.110 -0.721 6.978 1.00 0.00

ATOM 311 NH1 ARG A 40 19.329 0.479 6.488 1.00 0.00

ATOM 312 NH2 ARG A 40 19.821 -1.177 7.995 1.00 0.00

ATOM 313 N ASP A 41 12.775 -2.327 8.466 1.00 0.00

ATOM 314 CA ASP A 41 11.886 -3.376 8.953 1.00 0.00

ATOM 315 C ASP A 41 10.432 -2.924 8.923 1.00 0.00

ATOM 316 O ASP A 41 9.537 -3.701 8.589 1.00 0.00

ATOM 317 CB ASP A 41 12.277 -3.793 10.373 1.00 0.00

ATOM 318 CG ASP A 41 13.582 -4.574 10.462 1.00 0.00

ATOM 319 OD1 ASP A 41 14.091 -4.962 9.438 1.00 0.00

ATOM 320 OD2 ASP A 41 14.139 -4.640 11.532 1.00 0.00

ATOM 321 N ILE A 42 10.202 -1.663 9.274 1.00 0.00

ATOM 322 CA ILE A 42 8.852 -1.114 9.319 1.00 0.00

ATOM 323 C ILE A 42 8.266 -0.979 7.919 1.00 0.00

ATOM 324 O ILE A 42 7.073 -1.203 7.711 1.00 0.00

ATOM 325 CB ILE A 42 8.823 0.260 10.012 1.00 0.00

ATOM 326 CG1 ILE A 42 9.084 0.106 11.513 1.00 0.00

ATOM 327 CG2 ILE A 42 7.491 0.951 9.768 1.00 0.00

ATOM 328 CD1 ILE A 42 9.365 1.412 12.221 1.00 0.00

ATOM 329 N ILE A 43 9.111 -0.612 6.962 1.00 0.00

ATOM 330 CA ILE A 43 8.706 -0.555 5.563 1.00 0.00

ATOM 331 C ILE A 43 8.293 -1.930 5.052 1.00 0.00

ATOM 332 O ILE A 43 7.297 -2.065 4.341 1.00 0.00

ATOM 333 CB ILE A 43 9.832 -0.003 4.670 1.00 0.00

ATOM 334 CG1 ILE A 43 10.056 1.485 4.951 1.00 0.00

ATOM 335 CG2 ILE A 43 9.506 -0.228 3.202 1.00 0.00

ATOM 336 CD1 ILE A 43 11.311 2.043 4.321 1.00 0.00

ATOM 337 N LYS A 44 9.064 -2.948 5.418 1.00 0.00

ATOM 338 CA LYS A 44 8.739 -4.323 5.059 1.00 0.00

ATOM 339 C LYS A 44 7.427 -4.763 5.694 1.00 0.00

ATOM 340 O LYS A 44 6.612 -5.432 5.059 1.00 0.00

ATOM 341 CB LYS A 44 9.868 -5.266 5.477 1.00 0.00

ATOM 342 CG LYS A 44 11.147 -5.117 4.663 1.00 0.00

ATOM 343 CD LYS A 44 12.231 -6.064 5.156 1.00 0.00

ATOM 344 CE LYS A 44 13.525 -5.878 4.379 1.00 0.00

ATOM 345 NZ LYS A 44 14.592 -6.806 4.844 1.00 0.00

ATOM 346 N SER A 45 7.227 -4.383 6.952 1.00 0.00

ATOM 347 CA SER A 45 5.997 -4.707 7.664 1.00 0.00

ATOM 348 C SER A 45 4.796 -4.016 7.030 1.00 0.00

ATOM 349 O SER A 45 3.715 -4.598 6.930 1.00 0.00

ATOM 350 CB SER A 45 6.121 -4.317 9.124 1.00 0.00

ATOM 351 OG SER A 45 7.078 -5.088 9.795 1.00 0.00

ATOM 352 N MET A 46 4.991 -2.773 6.604 1.00 0.00

ATOM 353 CA MET A 46 3.947 -2.028 5.911 1.00 0.00

ATOM 354 C MET A 46 3.506 -2.748 4.643 1.00 0.00

ATOM 355 O MET A 46 2.314 -2.956 4.419 1.00 0.00

ATOM 356 CB MET A 46 4.437 -0.621 5.576 1.00 0.00

ATOM 357 CG MET A 46 3.421 0.242 4.840 1.00 0.00

ATOM 358 SD MET A 46 3.323 -0.148 3.082 1.00 0.00

ATOM 359 CE MET A 46 4.897 0.481 2.507 1.00 0.00

ATOM 360 N LYS A 47 4.475 -3.127 3.816 1.00 0.00

ATOM 361 CA LYS A 47 4.188 -3.823 2.568 1.00 0.00

ATOM 362 C LYS A 47 3.493 -5.154 2.827 1.00 0.00

ATOM 363 O LYS A 47 2.549 -5.519 2.127 1.00 0.00

ATOM 364 CB LYS A 47 5.475 -4.048 1.772 1.00 0.00

ATOM 365 CG LYS A 47 5.289 -4.847 0.489 1.00 0.00

ATOM 366 CD LYS A 47 6.569 -4.878 -0.334 1.00 0.00

ATOM 367 CE LYS A 47 6.501 -5.936 -1.425 1.00 0.00

ATOM 368 NZ LYS A 47 7.751 -5.986 -2.230 1.00 0.00

ATOM 369 N ASP A 48 3.967 -5.876 3.837 1.00 0.00

ATOM 370 CA ASP A 48 3.389 -7.167 4.193 1.00 0.00

ATOM 371 C ASP A 48 1.945 -7.015 4.653 1.00 0.00

ATOM 372 O ASP A 48 1.096 -7.855 4.353 1.00 0.00

ATOM 373 CB ASP A 48 4.221 -7.844 5.285 1.00 0.00

ATOM 374 CG ASP A 48 5.554 -8.402 4.805 1.00 0.00

ATOM 375 OD1 ASP A 48 5.745 -8.489 3.615 1.00 0.00

ATOM 376 OD2 ASP A 48 6.424 -8.590 5.620 1.00 0.00

ATOM 377 N ASN A 49 1.672 -5.940 5.384 1.00 0.00

ATOM 378 CA ASN A 49 0.318 -5.645 5.837 1.00 0.00

ATOM 379 C ASN A 49 -0.560 -5.179 4.683 1.00 0.00

ATOM 380 O ASN A 49 -1.762 -5.442 4.658 1.00 0.00

ATOM 381 CB ASN A 49 0.317 -4.611 6.948 1.00 0.00

ATOM 382 CG ASN A 49 0.836 -5.131 8.260 1.00 0.00

ATOM 383 OD1 ASN A 49 0.789 -6.336 8.535 1.00 0.00

ATOM 384 ND2 ASN A 49 1.256 -4.222 9.101 1.00 0.00

ATOM 385 N GLY A 50 0.048 -4.484 3.727 1.00 0.00

ATOM 386 CA GLY A 50 -0.668 -4.016 2.546 1.00 0.00

ATOM 387 C GLY A 50 -1.558 -2.825 2.878 1.00 0.00

ATOM 388 O GLY A 50 -2.550 -2.571 2.196 1.00 0.00

ATOM 389 N LYS A 51 -1.196 -2.097 3.929 1.00 0.00

ATOM 390 CA LYS A 51 -1.999 -0.973 4.395 1.00 0.00

ATOM 391 C LYS A 51 -1.122 0.223 4.744 1.00 0.00

ATOM 392 O LYS A 51 0.061 0.070 5.048 1.00 0.00

ATOM 393 CB LYS A 51 -2.840 -1.380 5.606 1.00 0.00

ATOM 394 CG LYS A 51 -3.943 -2.383 5.296 1.00 0.00

ATOM 395 CD LYS A 51 -4.800 -2.659 6.523 1.00 0.00

ATOM 396 CE LYS A 51 -5.919 -3.640 6.208 1.00 0.00

ATOM 397 NZ LYS A 51 -6.772 -3.912 7.397 1.00 0.00

ATOM 398 N PRO A 52 -1.709 1.414 4.700 1.00 0.00

ATOM 399 CA PRO A 52 -0.973 2.642 4.977 1.00 0.00

ATOM 400 C PRO A 52 -0.336 2.602 6.360 1.00 0.00

ATOM 401 O PRO A 52 -0.969 2.195 7.334 1.00 0.00

ATOM 402 CB PRO A 52 -2.029 3.746 4.860 1.00 0.00

ATOM 403 CG PRO A 52 -3.064 3.175 3.952 1.00 0.00

ATOM 404 CD PRO A 52 -3.112 1.705 4.274 1.00 0.00

ATOM 405 N LEU A 53 0.920 3.027 6.440 1.00 0.00

ATOM 406 CA LEU A 53 1.651 3.025 7.702 1.00 0.00

ATOM 407 C LEU A 53 2.471 4.298 7.865 1.00 0.00

ATOM 408 O LEU A 53 3.211 4.692 6.964 1.00 0.00

ATOM 409 CB LEU A 53 2.558 1.791 7.786 1.00 0.00

ATOM 410 CG LEU A 53 3.219 1.559 9.151 1.00 0.00

ATOM 411 CD1 LEU A 53 3.484 0.073 9.356 1.00 0.00

ATOM 412 CD2 LEU A 53 4.513 2.354 9.232 1.00 0.00

ATOM 413 N VAL A 54 2.335 4.938 9.022 1.00 0.00

ATOM 414 CA VAL A 54 3.107 6.136 9.329 1.00 0.00

ATOM 415 C VAL A 54 4.168 5.852 10.384 1.00 0.00

ATOM 416 O VAL A 54 3.856 5.403 11.487 1.00 0.00

ATOM 417 CB VAL A 54 2.202 7.281 9.821 1.00 0.00

ATOM 418 CG1 VAL A 54 3.028 8.525 10.114 1.00 0.00

ATOM 419 CG2 VAL A 54 1.125 7.589 8.792 1.00 0.00

ATOM 420 N VAL A 55 5.424 6.117 10.040 1.00 0.00

ATOM 421 CA VAL A 55 6.536 5.867 10.949 1.00 0.00

ATOM 422 C VAL A 55 7.178 7.171 11.405 1.00 0.00

ATOM 423 O VAL A 55 7.511 8.029 10.588 1.00 0.00

ATOM 424 CB VAL A 55 7.611 4.978 10.295 1.00 0.00

ATOM 425 CG1 VAL A 55 8.094 5.597 8.991 1.00 0.00

ATOM 426 CG2 VAL A 55 8.779 4.766 11.247 1.00 0.00

ATOM 427 N PHE A 56 7.350 7.313 12.715 1.00 0.00

ATOM 428 CA PHE A 56 7.883 8.543 13.288 1.00 0.00

ATOM 429 C PHE A 56 9.339 8.372 13.701 1.00 0.00

ATOM 430 O PHE A 56 9.722 7.337 14.247 1.00 0.00

ATOM 431 CB PHE A 56 7.043 8.980 14.490 1.00 0.00

ATOM 432 CG PHE A 56 5.637 9.372 14.136 1.00 0.00

ATOM 433 CD1 PHE A 56 4.683 8.407 13.851 1.00 0.00

ATOM 434 CD2 PHE A 56 5.265 10.707 14.087 1.00 0.00

ATOM 435 CE1 PHE A 56 3.389 8.767 13.525 1.00 0.00

ATOM 436 CE2 PHE A 56 3.973 11.070 13.763 1.00 0.00

ATOM 437 CZ PHE A 56 3.034 10.098 13.481 1.00 0.00

ATOM 438 N VAL A 57 10.147 9.393 13.438 1.00 0.00

ATOM 439 CA VAL A 57 11.548 9.386 13.845 1.00 0.00

ATOM 440 C VAL A 57 11.948 10.720 14.463 1.00 0.00

ATOM 441 O VAL A 57 11.696 11.781 13.890 1.00 0.00

ATOM 442 CB VAL A 57 12.480 9.082 12.657 1.00 0.00

ATOM 443 CG1 VAL A 57 13.931 9.048 13.114 1.00 0.00

ATOM 444 CG2 VAL A 57 12.098 7.762 12.005 1.00 0.00

ATOM 445 N ASN A 58 12.572 10.660 15.634 1.00 0.00

ATOM 446 CA ASN A 58 13.111 11.853 16.276 1.00 0.00

ATOM 447 C ASN A 58 14.552 12.104 15.850 1.00 0.00

ATOM 448 O ASN A 58 15.459 11.360 16.223 1.00 0.00

ATOM 449 CB ASN A 58 13.021 11.758 17.788 1.00 0.00

ATOM 450 CG ASN A 58 13.493 12.994 18.502 1.00 0.00

ATOM 451 OD1 ASN A 58 14.301 13.768 17.976 1.00 0.00

ATOM 452 ND2 ASN A 58 13.057 13.140 19.727 1.00 0.00

ATOM 453 N GLY A 59 14.756 13.157 15.066 1.00 0.00

ATOM 454 CA GLY A 59 16.094 13.538 14.628 1.00 0.00

ATOM 455 C GLY A 59 16.600 12.608 13.533 1.00 0.00

ATOM 456 O GLY A 59 17.754 12.181 13.553 1.00 0.00

ATOM 457 N ALA A 60 15.730 12.299 12.577 1.00 0.00

ATOM 458 CA ALA A 60 16.103 11.462 11.443 1.00 0.00

ATOM 459 C ALA A 60 17.265 12.071 10.668 1.00 0.00

ATOM 460 O ALA A 60 17.173 13.195 10.175 1.00 0.00

ATOM 461 CB ALA A 60 14.906 11.246 10.528 1.00 0.00

ATOM 462 N SER A 61 18.357 11.322 10.564 1.00 0.00

ATOM 463 CA SER A 61 19.525 11.771 9.816 1.00 0.00

ATOM 464 C SER A 61 19.336 11.558 8.319 1.00 0.00

ATOM 465 O SER A 61 18.395 10.889 7.892 1.00 0.00

ATOM 466 CB SER A 61 20.766 11.046 10.300 1.00 0.00

ATOM 467 OG SER A 61 20.755 9.690 9.946 1.00 0.00

ATOM 468 N GLN A 62 20.236 12.131 7.527 1.00 0.00

ATOM 469 CA GLN A 62 20.182 11.986 6.077 1.00 0.00

ATOM 470 C GLN A 62 20.304 10.525 5.664 1.00 0.00

ATOM 471 O GLN A 62 19.724 10.103 4.664 1.00 0.00

ATOM 472 CB GLN A 62 21.293 12.805 5.415 1.00 0.00

ATOM 473 CG GLN A 62 21.091 14.309 5.496 1.00 0.00

ATOM 474 CD GLN A 62 22.257 15.082 4.909 1.00 0.00

ATOM 475 OE1 GLN A 62 23.254 14.496 4.477 1.00 0.00

ATOM 476 NE2 GLN A 62 22.140 16.405 4.893 1.00 0.00

ATOM 477 N ASN A 63 21.062 9.758 6.440 1.00 0.00

ATOM 478 CA ASN A 63 21.224 8.332 6.182 1.00 0.00

ATOM 479 C ASN A 63 19.911 7.584 6.375 1.00 0.00

ATOM 480 O ASN A 63 19.616 6.632 5.653 1.00 0.00

ATOM 481 CB ASN A 63 22.305 7.729 7.061 1.00 0.00

ATOM 482 CG ASN A 63 23.700 8.119 6.658 1.00 0.00

ATOM 483 OD1 ASN A 63 23.947 8.527 5.518 1.00 0.00

ATOM 484 ND2 ASN A 63 24.625 7.919 7.562 1.00 0.00

ATOM 485 N ASP A 64 19.125 8.022 7.352 1.00 0.00

ATOM 486 CA ASP A 64 17.854 7.377 7.660 1.00 0.00

ATOM 487 C ASP A 64 16.800 7.708 6.611 1.00 0.00

ATOM 488 O ASP A 64 15.973 6.866 6.261 1.00 0.00

ATOM 489 CB ASP A 64 17.363 7.794 9.048 1.00 0.00

ATOM 490 CG ASP A 64 18.332 7.478 10.180 1.00 0.00

ATOM 491 OD1 ASP A 64 18.789 6.361 10.249 1.00 0.00

ATOM 492 OD2 ASP A 64 18.727 8.388 10.868 1.00 0.00

ATOM 493 N VAL A 65 16.835 8.939 6.112 1.00 0.00

ATOM 494 CA VAL A 65 15.929 9.362 5.052 1.00 0.00

ATOM 495 C VAL A 65 16.268 8.680 3.733 1.00 0.00

ATOM 496 O VAL A 65 15.378 8.268 2.989 1.00 0.00

ATOM 497 CB VAL A 65 15.966 10.889 4.852 1.00 0.00

ATOM 498 CG1 VAL A 65 15.156 11.286 3.627 1.00 0.00

ATOM 499 CG2 VAL A 65 15.440 11.601 6.089 1.00 0.00

ATOM 500 N ASN A 66 17.561 8.562 3.448 1.00 0.00

ATOM 501 CA ASN A 66 18.021 7.903 2.232 1.00 0.00

ATOM 502 C ASN A 66 17.665 6.422 2.240 1.00 0.00

ATOM 503 O ASN A 66 17.224 5.874 1.230 1.00 0.00

ATOM 504 CB ASN A 66 19.515 8.083 2.034 1.00 0.00

ATOM 505 CG ASN A 66 19.904 9.466 1.591 1.00 0.00

ATOM 506 OD1 ASN A 66 19.085 10.222 1.056 1.00 0.00

ATOM 507 ND2 ASN A 66 21.169 9.770 1.737 1.00 0.00

ATOM 508 N GLU A 67 17.860 5.778 3.386 1.00 0.00

ATOM 509 CA GLU A 67 17.517 4.370 3.542 1.00 0.00

ATOM 510 C GLU A 67 16.010 4.159 3.467 1.00 0.00

ATOM 511 O GLU A 67 15.539 3.166 2.911 1.00 0.00

ATOM 512 CB GLU A 67 18.058 3.830 4.868 1.00 0.00

ATOM 513 CG GLU A 67 19.556 3.561 4.873 1.00 0.00

ATOM 514 CD GLU A 67 19.926 2.513 3.860 1.00 0.00

ATOM 515 OE1 GLU A 67 19.338 1.458 3.882 1.00 0.00

ATOM 516 OE2 GLU A 67 20.716 2.806 2.993 1.00 0.00

ATOM 517 N PHE A 68 15.257 5.098 4.029 1.00 0.00

ATOM 518 CA PHE A 68 13.803 5.082 3.920 1.00 0.00

ATOM 519 C PHE A 68 13.361 5.031 2.463 1.00 0.00

ATOM 520 O PHE A 68 12.606 4.145 2.064 1.00 0.00

ATOM 521 CB PHE A 68 13.202 6.309 4.609 1.00 0.00

ATOM 522 CG PHE A 68 11.731 6.483 4.360 1.00 0.00

ATOM 523 CD1 PHE A 68 10.801 5.724 5.055 1.00 0.00

ATOM 524 CD2 PHE A 68 11.274 7.406 3.432 1.00 0.00

ATOM 525 CE1 PHE A 68 9.447 5.883 4.827 1.00 0.00

ATOM 526 CE2 PHE A 68 9.921 7.568 3.203 1.00 0.00

ATOM 527 CZ PHE A 68 9.007 6.805 3.902 1.00 0.00

ATOM 528 N GLN A 69 13.837 5.987 1.671 1.00 0.00

ATOM 529 CA GLN A 69 13.433 6.096 0.274 1.00 0.00

ATOM 530 C GLN A 69 13.927 4.905 -0.536 1.00 0.00

ATOM 531 O GLN A 69 13.187 4.340 -1.342 1.00 0.00

ATOM 532 CB GLN A 69 13.967 7.394 -0.338 1.00 0.00

ATOM 533 CG GLN A 69 13.329 8.654 0.223 1.00 0.00

ATOM 534 CD GLN A 69 13.963 9.917 -0.328 1.00 0.00

ATOM 535 OE1 GLN A 69 14.969 9.864 -1.042 1.00 0.00

ATOM 536 NE2 GLN A 69 13.380 11.064 0.004 1.00 0.00

ATOM 537 N ASN A 70 15.183 4.527 -0.318 1.00 0.00

ATOM 538 CA ASN A 70 15.810 3.466 -1.098 1.00 0.00

ATOM 539 C ASN A 70 15.130 2.126 -0.853 1.00 0.00

ATOM 540 O ASN A 70 14.894 1.358 -1.785 1.00 0.00

ATOM 541 CB ASN A 70 17.295 3.364 -0.802 1.00 0.00

ATOM 542 CG ASN A 70 18.110 4.475 -1.403 1.00 0.00

ATOM 543 OD1 ASN A 70 17.686 5.139 -2.355 1.00 0.00

ATOM 544 ND2 ASN A 70 19.311 4.627 -0.904 1.00 0.00

ATOM 545 N GLU A 71 14.816 1.850 0.409 1.00 0.00

ATOM 546 CA GLU A 71 14.170 0.597 0.781 1.00 0.00

ATOM 547 C GLU A 71 12.774 0.497 0.179 1.00 0.00

ATOM 548 O GLU A 71 12.363 -0.566 -0.286 1.00 0.00

ATOM 549 CB GLU A 71 14.096 0.463 2.304 1.00 0.00

ATOM 550 CG GLU A 71 13.497 -0.848 2.792 1.00 0.00

ATOM 551 CD GLU A 71 14.390 -2.012 2.464 1.00 0.00

ATOM 552 OE1 GLU A 71 15.516 -1.786 2.092 1.00 0.00

ATOM 553 OE2 GLU A 71 13.986 -3.128 2.692 1.00 0.00

ATOM 554 N ALA A 72 12.049 1.610 0.190 1.00 0.00

ATOM 555 CA ALA A 72 10.719 1.664 -0.404 1.00 0.00

ATOM 556 C ALA A 72 10.781 1.455 -1.912 1.00 0.00

ATOM 557 O ALA A 72 9.905 0.816 -2.496 1.00 0.00

ATOM 558 CB ALA A 72 10.046 2.989 -0.077 1.00 0.00

ATOM 559 N LYS A 73 11.820 1.997 -2.537 1.00 0.00

ATOM 560 CA LYS A 73 12.046 1.797 -3.964 1.00 0.00

ATOM 561 C LYS A 73 12.369 0.340 -4.271 1.00 0.00

ATOM 562 O LYS A 73 11.919 -0.207 -5.277 1.00 0.00

ATOM 563 CB LYS A 73 13.174 2.702 -4.460 1.00 0.00

ATOM 564 CG LYS A 73 12.806 4.178 -4.543 1.00 0.00

ATOM 565 CD LYS A 73 13.983 5.016 -5.019 1.00 0.00

ATOM 566 CE LYS A 73 13.630 6.495 -5.061 1.00 0.00

ATOM 567 NZ LYS A 73 14.755 7.322 -5.575 1.00 0.00

ATOM 568 N LYS A 74 13.153 -0.283 -3.398 1.00 0.00

ATOM 569 CA LYS A 74 13.524 -1.683 -3.564 1.00 0.00

ATOM 570 C LYS A 74 12.310 -2.594 -3.434 1.00 0.00

ATOM 571 O LYS A 74 12.197 -3.598 -4.136 1.00 0.00

ATOM 572 CB LYS A 74 14.591 -2.080 -2.542 1.00 0.00

ATOM 573 CG LYS A 74 15.970 -1.492 -2.811 1.00 0.00

ATOM 574 CD LYS A 74 16.947 -1.840 -1.699 1.00 0.00

ATOM 575 CE LYS A 74 18.307 -1.201 -1.935 1.00 0.00

ATOM 576 NZ LYS A 74 19.267 -1.516 -0.843 1.00 0.00

ATOM 577 N GLU A 75 11.403 -2.236 -2.531 1.00 0.00

ATOM 578 CA GLU A 75 10.187 -3.012 -2.319 1.00 0.00

ATOM 579 C GLU A 75 9.139 -2.696 -3.379 1.00 0.00

ATOM 580 O GLU A 75 8.277 -3.522 -3.678 1.00 0.00

ATOM 581 CB GLU A 75 9.619 -2.746 -0.923 1.00 0.00

ATOM 582 CG GLU A 75 10.451 -3.320 0.215 1.00 0.00

ATOM 583 CD GLU A 75 10.707 -4.789 0.017 1.00 0.00

ATOM 584 OE1 GLU A 75 9.763 -5.515 -0.184 1.00 0.00

ATOM 585 OE2 GLU A 75 11.852 -5.169 -0.045 1.00 0.00

ATOM 586 N GLY A 76 9.220 -1.497 -3.944 1.00 0.00

ATOM 587 CA GLY A 76 8.300 -1.082 -4.997 1.00 0.00

ATOM 588 C GLY A 76 7.008 -0.526 -4.411 1.00 0.00

ATOM 589 O GLY A 76 5.918 -0.813 -4.906 1.00 0.00

ATOM 590 N VAL A 77 7.138 0.271 -3.356 1.00 0.00

ATOM 591 CA VAL A 77 5.978 0.818 -2.663 1.00 0.00

ATOM 592 C VAL A 77 6.036 2.339 -2.607 1.00 0.00

ATOM 593 O VAL A 77 7.076 2.940 -2.877 1.00 0.00

ATOM 594 CB VAL A 77 5.864 0.266 -1.229 1.00 0.00

ATOM 595 CG1 VAL A 77 5.663 -1.242 -1.253 1.00 0.00

ATOM 596 CG2 VAL A 77 7.102 0.624 -0.420 1.00 0.00

ATOM 597 N SER A 78 4.913 2.957 -2.257 1.00 0.00

ATOM 598 CA SER A 78 4.844 4.408 -2.131 1.00 0.00

ATOM 599 C SER A 78 5.493 4.880 -0.836 1.00 0.00

ATOM 600 O SER A 78 5.394 4.217 0.197 1.00 0.00

ATOM 601 CB SER A 78 3.401 4.870 -2.198 1.00 0.00

ATOM 602 OG SER A 78 2.821 4.604 -3.445 1.00 0.00

ATOM 603 N TYR A 79 6.157 6.029 -0.897 1.00 0.00

ATOM 604 CA TYR A 79 6.863 6.568 0.259 1.00 0.00

ATOM 605 C TYR A 79 6.852 8.092 0.249 1.00 0.00

ATOM 606 O TYR A 79 6.985 8.716 -0.803 1.00 0.00

ATOM 607 CB TYR A 79 8.304 6.053 0.294 1.00 0.00

ATOM 608 CG TYR A 79 9.146 6.507 -0.878 1.00 0.00

ATOM 609 CD1 TYR A 79 9.889 7.676 -0.810 1.00 0.00

ATOM 610 CD2 TYR A 79 9.196 5.764 -2.047 1.00 0.00

ATOM 611 CE1 TYR A 79 10.660 8.096 -1.877 1.00 0.00

ATOM 612 CE2 TYR A 79 9.963 6.173 -3.121 1.00 0.00

ATOM 613 CZ TYR A 79 10.694 7.341 -3.032 1.00 0.00

ATOM 614 OH TYR A 79 11.461 7.752 -4.098 1.00 0.00

ATOM 615 N ASP A 80 6.693 8.685 1.428 1.00 0.00

ATOM 616 CA ASP A 80 6.742 10.135 1.568 1.00 0.00

ATOM 617 C ASP A 80 7.609 10.545 2.752 1.00 0.00

ATOM 618 O ASP A 80 7.733 9.804 3.728 1.00 0.00

ATOM 619 CB ASP A 80 5.331 10.706 1.726 1.00 0.00

ATOM 620 CG ASP A 80 5.224 12.201 1.457 1.00 0.00

ATOM 621 OD1 ASP A 80 6.190 12.776 1.016 1.00 0.00

ATOM 622 OD2 ASP A 80 4.138 12.724 1.545 1.00 0.00

ATOM 623 N VAL A 81 8.206 11.728 2.660 1.00 0.00

ATOM 624 CA VAL A 81 9.012 12.266 3.750 1.00 0.00

ATOM 625 C VAL A 81 8.550 13.665 4.137 1.00 0.00

ATOM 626 O VAL A 81 8.636 14.600 3.340 1.00 0.00

ATOM 627 CB VAL A 81 10.506 12.313 3.378 1.00 0.00

ATOM 628 CG1 VAL A 81 11.320 12.904 4.520 1.00 0.00

ATOM 629 CG2 VAL A 81 11.012 10.922 3.029 1.00 0.00

ATOM 630 N LEU A 82 8.061 13.803 5.364 1.00 0.00

ATOM 631 CA LEU A 82 7.636 15.099 5.879 1.00 0.00

ATOM 632 C LEU A 82 8.381 15.455 7.159 1.00 0.00

ATOM 633 O LEU A 82 8.614 14.599 8.011 1.00 0.00

ATOM 634 CB LEU A 82 6.122 15.102 6.124 1.00 0.00

ATOM 635 CG LEU A 82 5.259 14.842 4.883 1.00 0.00

ATOM 636 CD1 LEU A 82 3.798 14.692 5.285 1.00 0.00

ATOM 637 CD2 LEU A 82 5.432 15.985 3.894 1.00 0.00

ATOM 638 N LYS A 83 8.752 16.725 7.288 1.00 0.00

ATOM 639 CA LYS A 83 9.536 17.181 8.429 1.00 0.00

ATOM 640 C LYS A 83 8.982 18.484 8.991 1.00 0.00

ATOM 641 O LYS A 83 8.655 19.405 8.243 1.00 0.00

ATOM 642 CB LYS A 83 11.003 17.360 8.034 1.00 0.00

ATOM 643 CG LYS A 83 11.923 17.736 9.188 1.00 0.00

ATOM 644 CD LYS A 83 13.372 17.825 8.733 1.00 0.00

ATOM 645 CE LYS A 83 14.290 18.223 9.880 1.00 0.00

ATOM 646 NZ LYS A 83 15.715 18.285 9.456 1.00 0.00

ATOM 647 N SER A 84 8.878 18.556 10.314 1.00 0.00

ATOM 648 CA SER A 84 8.415 19.766 10.982 1.00 0.00

ATOM 649 C SER A 84 8.560 19.650 12.494 1.00 0.00

ATOM 650 O SER A 84 8.637 18.548 13.037 1.00 0.00

ATOM 651 CB SER A 84 6.973 20.049 10.609 1.00 0.00

ATOM 652 OG SER A 84 6.431 21.100 11.361 1.00 0.00

ATOM 653 N THR A 85 8.595 20.794 13.170 1.00 0.00

ATOM 654 CA THR A 85 8.618 20.822 14.627 1.00 0.00

ATOM 655 C THR A 85 7.322 21.393 15.187 1.00 0.00

ATOM 656 O THR A 85 7.241 21.736 16.367 1.00 0.00

ATOM 657 CB THR A 85 9.803 21.650 15.158 1.00 0.00

ATOM 658 OG1 THR A 85 9.722 22.987 14.646 1.00 0.00

ATOM 659 CG2 THR A 85 11.123 21.027 14.731 1.00 0.00

ATOM 660 N ASP A 86 6.308 21.494 14.334 1.00 0.00

ATOM 661 CA ASP A 86 4.982 21.921 14.766 1.00 0.00

ATOM 662 C ASP A 86 4.024 20.740 14.854 1.00 0.00

ATOM 663 O ASP A 86 3.759 20.068 13.857 1.00 0.00

ATOM 664 CB ASP A 86 4.424 22.981 13.815 1.00 0.00

ATOM 665 CG ASP A 86 3.072 23.548 14.228 1.00 0.00

ATOM 666 OD1 ASP A 86 2.535 23.095 15.211 1.00 0.00

ATOM 667 OD2 ASP A 86 2.661 24.529 13.655 1.00 0.00

ATOM 668 N PRO A 87 3.508 20.491 16.053 1.00 0.00

ATOM 669 CA PRO A 87 2.632 19.349 16.287 1.00 0.00

ATOM 670 C PRO A 87 1.316 19.502 15.536 1.00 0.00

ATOM 671 O PRO A 87 0.688 18.513 15.156 1.00 0.00

ATOM 672 CB PRO A 87 2.430 19.338 17.806 1.00 0.00

ATOM 673 CG PRO A 87 2.728 20.735 18.231 1.00 0.00

ATOM 674 CD PRO A 87 3.822 21.208 17.310 1.00 0.00

ATOM 675 N GLU A 88 0.902 20.747 15.323 1.00 0.00

ATOM 676 CA GLU A 88 -0.336 21.032 14.609 1.00 0.00

ATOM 677 C GLU A 88 -0.173 20.806 13.111 1.00 0.00

ATOM 678 O GLU A 88 -1.079 20.302 12.447 1.00 0.00

ATOM 679 CB GLU A 88 -0.792 22.468 14.878 1.00 0.00

ATOM 680 CG GLU A 88 -1.209 22.736 16.317 1.00 0.00

ATOM 681 CD GLU A 88 -2.376 21.876 16.716 1.00 0.00

ATOM 682 OE1 GLU A 88 -3.365 21.890 16.023 1.00 0.00

ATOM 683 OE2 GLU A 88 -2.239 21.121 17.650 1.00 0.00

ATOM 684 N GLU A 89 0.987 21.182 12.585 1.00 0.00

ATOM 685 CA GLU A 89 1.296 20.964 11.176 1.00 0.00

ATOM 686 C GLU A 89 1.389 19.478 10.856 1.00 0.00

ATOM 687 O GLU A 89 0.779 19.000 9.899 1.00 0.00

ATOM 688 CB GLU A 89 2.602 21.666 10.799 1.00 0.00

ATOM 689 CG GLU A 89 3.051 21.431 9.364 1.00 0.00

ATOM 690 CD GLU A 89 2.105 22.068 8.385 1.00 0.00

ATOM 691 OE1 GLU A 89 1.269 22.832 8.806 1.00 0.00

ATOM 692 OE2 GLU A 89 2.287 21.881 7.205 1.00 0.00

ATOM 693 N LEU A 90 2.155 18.751 11.662 1.00 0.00

ATOM 694 CA LEU A 90 2.344 17.320 11.455 1.00 0.00

ATOM 695 C LEU A 90 1.041 16.558 11.655 1.00 0.00

ATOM 696 O LEU A 90 0.777 15.570 10.970 1.00 0.00

ATOM 697 CB LEU A 90 3.426 16.786 12.402 1.00 0.00

ATOM 698 CG LEU A 90 4.856 17.237 12.080 1.00 0.00

ATOM 699 CD1 LEU A 90 5.819 16.717 13.140 1.00 0.00

ATOM 700 CD2 LEU A 90 5.250 16.733 10.700 1.00 0.00

ATOM 701 N THR A 91 0.228 17.022 12.598 1.00 0.00

ATOM 702 CA THR A 91 -1.109 16.476 12.795 1.00 0.00

ATOM 703 C THR A 91 -1.918 16.522 11.506 1.00 0.00

ATOM 704 O THR A 91 -2.456 15.508 11.062 1.00 0.00

ATOM 705 CB THR A 91 -1.873 17.235 13.896 1.00 0.00

ATOM 706 OG1 THR A 91 -1.270 16.970 15.169 1.00 0.00

ATOM 707 CG2 THR A 91 -3.331 16.801 13.929 1.00 0.00

ATOM 708 N GLN A 92 -2.002 17.706 10.908 1.00 0.00

ATOM 709 CA GLN A 92 -2.785 17.897 9.693 1.00 0.00

ATOM 710 C GLN A 92 -2.165 17.156 8.516 1.00 0.00

ATOM 711 O GLN A 92 -2.873 16.649 7.646 1.00 0.00

ATOM 712 CB GLN A 92 -2.904 19.387 9.361 1.00 0.00

ATOM 713 CG GLN A 92 -3.701 20.189 10.375 1.00 0.00

ATOM 714 CD GLN A 92 -5.116 19.668 10.541 1.00 0.00

ATOM 715 OE1 GLN A 92 -5.780 19.312 9.564 1.00 0.00

ATOM 716 NE2 GLN A 92 -5.588 19.625 11.782 1.00 0.00

ATOM 717 N ARG A 93 -0.838 17.096 8.494 1.00 0.00

ATOM 718 CA ARG A 93 -0.120 16.385 7.443 1.00 0.00

ATOM 719 C ARG A 93 -0.481 14.905 7.434 1.00 0.00

ATOM 720 O ARG A 93 -0.827 14.346 6.393 1.00 0.00

ATOM 721 CB ARG A 93 1.385 16.590 7.534 1.00 0.00

ATOM 722 CG ARG A 93 1.875 17.946 7.051 1.00 0.00

ATOM 723 CD ARG A 93 3.344 18.141 7.162 1.00 0.00

ATOM 724 NE ARG A 93 3.796 19.492 6.870 1.00 0.00

ATOM 725 CZ ARG A 93 5.083 19.889 6.891 1.00 0.00

ATOM 726 NH1 ARG A 93 6.044 19.055 7.224 1.00 0.00

ATOM 727 NH2 ARG A 93 5.354 21.147 6.589 1.00 0.00

ATOM 728 N VAL A 94 -0.399 14.274 8.601 1.00 0.00

ATOM 729 CA VAL A 94 -0.690 12.851 8.724 1.00 0.00

ATOM 730 C VAL A 94 -2.179 12.577 8.556 1.00 0.00

ATOM 731 O VAL A 94 -2.573 11.568 7.971 1.00 0.00

ATOM 732 CB VAL A 94 -0.226 12.295 10.084 1.00 0.00

ATOM 733 CG1 VAL A 94 -0.701 10.861 10.263 1.00 0.00

ATOM 734 CG2 VAL A 94 1.288 12.372 10.202 1.00 0.00

ATOM 735 N ARG A 95 -3.004 13.481 9.073 1.00 0.00

ATOM 736 CA ARG A 95 -4.450 13.372 8.926 1.00 0.00

ATOM 737 C ARG A 95 -4.851 13.295 7.459 1.00 0.00

ATOM 738 O ARG A 95 -5.596 12.403 7.054 1.00 0.00

ATOM 739 CB ARG A 95 -5.187 14.492 9.647 1.00 0.00

ATOM 740 CG ARG A 95 -6.704 14.401 9.588 1.00 0.00

ATOM 741 CD ARG A 95 -7.410 15.578 10.155 1.00 0.00

ATOM 742 NE ARG A 95 -7.236 16.807 9.397 1.00 0.00

ATOM 743 CZ ARG A 95 -7.880 17.096 8.250 1.00 0.00

ATOM 744 NH1 ARG A 95 -8.711 16.236 7.704 1.00 0.00

ATOM 745 NH2 ARG A 95 -7.636 18.260 7.673 1.00 0.00

ATOM 746 N GLU A 96 -4.351 14.236 6.664 1.00 0.00

ATOM 747 CA GLU A 96 -4.685 14.298 5.246 1.00 0.00

ATOM 748 C GLU A 96 -4.066 13.134 4.484 1.00 0.00

ATOM 749 O GLU A 96 -4.666 12.603 3.548 1.00 0.00

ATOM 750 CB GLU A 96 -4.221 15.627 4.645 1.00 0.00

ATOM 751 CG GLU A 96 -5.024 16.837 5.100 1.00 0.00

ATOM 752 CD GLU A 96 -4.446 18.113 4.553 1.00 0.00

ATOM 753 OE1 GLU A 96 -3.425 18.053 3.910 1.00 0.00

ATOM 754 OE2 GLU A 96 -5.082 19.132 4.681 1.00 0.00

ATOM 755 N PHE A 97 -2.863 12.740 4.889 1.00 0.00

ATOM 756 CA PHE A 97 -2.202 11.578 4.308 1.00 0.00

ATOM 757 C PHE A 97 -3.055 10.325 4.460 1.00 0.00

ATOM 758 O PHE A 97 -3.273 9.590 3.497 1.00 0.00

ATOM 759 CB PHE A 97 -0.832 11.364 4.954 1.00 0.00

ATOM 760 CG PHE A 97 -0.161 10.085 4.543 1.00 0.00

ATOM 761 CD1 PHE A 97 0.468 9.980 3.311 1.00 0.00

ATOM 762 CD2 PHE A 97 -0.157 8.983 5.385 1.00 0.00

ATOM 763 CE1 PHE A 97 1.086 8.803 2.931 1.00 0.00

ATOM 764 CE2 PHE A 97 0.460 7.807 5.009 1.00 0.00

ATOM 765 CZ PHE A 97 1.082 7.717 3.780 1.00 0.00

ATOM 766 N LEU A 98 -3.535 10.087 5.676 1.00 0.00

ATOM 767 CA LEU A 98 -4.306 8.885 5.974 1.00 0.00

ATOM 768 C LEU A 98 -5.734 9.005 5.458 1.00 0.00

ATOM 769 O LEU A 98 -6.392 8.001 5.185 1.00 0.00

ATOM 770 CB LEU A 98 -4.304 8.615 7.483 1.00 0.00

ATOM 771 CG LEU A 98 -2.927 8.325 8.093 1.00 0.00

ATOM 772 CD1 LEU A 98 -3.055 8.117 9.596 1.00 0.00

ATOM 773 CD2 LEU A 98 -2.325 7.096 7.429 1.00 0.00

ATOM 774 N LYS A 99 -6.208 10.239 5.326 1.00 0.00

ATOM 775 CA LYS A 99 -7.530 10.496 4.768 1.00 0.00

ATOM 776 C LYS A 99 -7.590 10.111 3.295 1.00 0.00

ATOM 777 O LYS A 99 -8.501 9.404 2.865 1.00 0.00

ATOM 778 CB LYS A 99 -7.909 11.968 4.942 1.00 0.00

ATOM 779 CG LYS A 99 -9.299 12.325 4.434 1.00 0.00

ATOM 780 CD LYS A 99 -9.630 13.784 4.705 1.00 0.00

ATOM 781 CE LYS A 99 -11.000 14.154 4.154 1.00 0.00

ATOM 782 NZ LYS A 99 -11.315 15.593 4.368 1.00 0.00

ATOM 783 N THR A 100 -6.614 10.582 2.526 1.00 0.00

ATOM 784 CA THR A 100 -6.532 10.255 1.107 1.00 0.00

ATOM 785 C THR A 100 -6.160 8.792 0.899 1.00 0.00

ATOM 786 O THR A 100 -6.757 8.101 0.074 1.00 0.00

ATOM 787 CB THR A 100 -5.505 11.143 0.381 1.00 0.00

ATOM 788 OG1 THR A 100 -5.912 12.515 0.465 1.00 0.00

ATOM 789 CG2 THR A 100 -5.390 10.741 -1.081 1.00 0.00

ATOM 790 N ALA A 101 -5.170 8.326 1.653 1.00 0.00

ATOM 791 CA ALA A 101 -4.762 6.927 1.602 1.00 0.00

ATOM 792 C ALA A 101 -5.384 6.130 2.742 1.00 0.00

ATOM 793 O ALA A 101 -6.519 5.751 2.659 1.00 0.00

ATOM 794 OXT ALA A 101 -4.738 5.881 3.722 1.00 0.00

ATOM 795 CB ALA A 101 -3.245 6.816 1.639 1.00 0.00

TER

ENDMDL

**PDB ID 2LTA**

ATOM 1 N SER A 1 0.000 0.000 0.000 1.00 0.00

ATOM 2 CA SER A 1 1.458 0.000 0.000 1.00 0.00

ATOM 3 C SER A 1 2.009 1.420 0.000 1.00 0.00

ATOM 4 O SER A 1 1.297 2.372 0.326 1.00 0.00

ATOM 5 CB SER A 1 1.980 -0.769 -1.198 1.00 0.00

ATOM 6 OG SER A 1 3.355 -1.018 -1.105 1.00 0.00

ATOM 7 N LYS A 2 3.279 1.559 -0.368 1.00 0.00

ATOM 8 CA LYS A 2 3.948 2.853 -0.347 1.00 0.00

ATOM 9 C LYS A 2 4.421 3.249 -1.739 1.00 0.00

ATOM 10 O LYS A 2 4.763 2.396 -2.555 1.00 0.00

ATOM 11 CB LYS A 2 5.129 2.830 0.625 1.00 0.00

ATOM 12 CG LYS A 2 4.789 2.310 2.016 1.00 0.00

ATOM 13 CD LYS A 2 3.820 3.238 2.733 1.00 0.00

ATOM 14 CE LYS A 2 3.586 2.793 4.169 1.00 0.00

ATOM 15 NZ LYS A 2 2.638 1.649 4.249 1.00 0.00

ATOM 16 N ILE A 3 4.440 4.552 -2.001 1.00 0.00

ATOM 17 CA ILE A 3 4.879 5.062 -3.298 1.00 0.00

ATOM 18 C ILE A 3 5.927 6.155 -3.129 1.00 0.00

ATOM 19 O ILE A 3 5.694 7.154 -2.446 1.00 0.00

ATOM 20 CB ILE A 3 3.699 5.615 -4.118 1.00 0.00

ATOM 21 CG1 ILE A 3 2.728 4.490 -4.483 1.00 0.00

ATOM 22 CG2 ILE A 3 4.204 6.315 -5.370 1.00 0.00

ATOM 23 CD1 ILE A 3 1.473 4.968 -5.177 1.00 0.00

ATOM 24 N ILE A 4 7.082 5.959 -3.757 1.00 0.00

ATOM 25 CA ILE A 4 8.136 6.970 -3.757 1.00 0.00

ATOM 26 C ILE A 4 8.299 7.592 -5.137 1.00 0.00

ATOM 27 O ILE A 4 8.685 6.920 -6.093 1.00 0.00

ATOM 28 CB ILE A 4 9.486 6.381 -3.308 1.00 0.00

ATOM 29 CG1 ILE A 4 9.371 5.798 -1.897 1.00 0.00

ATOM 30 CG2 ILE A 4 10.574 7.442 -3.362 1.00 0.00

ATOM 31 CD1 ILE A 4 9.000 4.333 -1.869 1.00 0.00

ATOM 32 N VAL A 5 8.004 8.885 -5.237 1.00 0.00

ATOM 33 CA VAL A 5 8.067 9.588 -6.513 1.00 0.00

ATOM 34 C VAL A 5 9.299 10.479 -6.588 1.00 0.00

ATOM 35 O VAL A 5 9.360 11.525 -5.947 1.00 0.00

ATOM 36 CB VAL A 5 6.809 10.446 -6.749 1.00 0.00

ATOM 37 CG1 VAL A 5 6.892 11.153 -8.093 1.00 0.00

ATOM 38 CG2 VAL A 5 5.557 9.586 -6.677 1.00 0.00

ATOM 39 N ILE A 6 10.279 10.055 -7.378 1.00 0.00

ATOM 40 CA ILE A 6 11.586 10.704 -7.392 1.00 0.00

ATOM 41 C ILE A 6 11.748 11.597 -8.615 1.00 0.00

ATOM 42 O ILE A 6 11.552 11.155 -9.747 1.00 0.00

ATOM 43 CB ILE A 6 12.729 9.673 -7.367 1.00 0.00

ATOM 44 CG1 ILE A 6 12.629 8.796 -6.116 1.00 0.00

ATOM 45 CG2 ILE A 6 14.078 10.373 -7.423 1.00 0.00

ATOM 46 CD1 ILE A 6 13.600 7.638 -6.103 1.00 0.00

ATOM 47 N ILE A 7 12.102 12.855 -8.380 1.00 0.00

ATOM 48 CA ILE A 7 12.341 13.800 -9.463 1.00 0.00

ATOM 49 C ILE A 7 13.819 14.146 -9.577 1.00 0.00

ATOM 50 O ILE A 7 14.449 14.551 -8.598 1.00 0.00

ATOM 51 CB ILE A 7 11.533 15.096 -9.270 1.00 0.00

ATOM 52 CG1 ILE A 7 10.033 14.791 -9.243 1.00 0.00

ATOM 53 CG2 ILE A 7 11.856 16.094 -10.372 1.00 0.00

ATOM 54 CD1 ILE A 7 9.465 14.644 -7.850 1.00 0.00

ATOM 55 N SER A 8 14.371 13.985 -10.775 1.00 0.00

ATOM 56 CA SER A 8 15.761 14.347 -11.034 1.00 0.00

ATOM 57 C SER A 8 16.026 14.476 -12.530 1.00 0.00

ATOM 58 O SER A 8 15.101 14.420 -13.341 1.00 0.00

ATOM 59 CB SER A 8 16.691 13.320 -10.419 1.00 0.00

ATOM 60 OG SER A 8 18.016 13.774 -10.369 1.00 0.00

ATOM 61 N SER A 9 17.293 14.648 -12.887 1.00 0.00

ATOM 62 CA SER A 9 17.687 14.767 -14.285 1.00 0.00

ATOM 63 C SER A 9 18.699 13.696 -14.667 1.00 0.00

ATOM 64 O SER A 9 18.985 13.489 -15.848 1.00 0.00

ATOM 65 CB SER A 9 18.253 16.148 -14.552 1.00 0.00

ATOM 66 OG SER A 9 17.303 17.156 -14.339 1.00 0.00

ATOM 67 N ASP A 10 19.244 13.018 -13.661 1.00 0.00

ATOM 68 CA ASP A 10 20.212 11.953 -13.891 1.00 0.00

ATOM 69 C ASP A 10 19.528 10.596 -13.981 1.00 0.00

ATOM 70 O ASP A 10 19.026 10.078 -12.981 1.00 0.00

ATOM 71 CB ASP A 10 21.266 11.937 -12.781 1.00 0.00

ATOM 72 CG ASP A 10 22.356 10.890 -12.963 1.00 0.00

ATOM 73 OD1 ASP A 10 22.369 10.245 -13.985 1.00 0.00

ATOM 74 OD2 ASP A 10 23.254 10.849 -12.156 1.00 0.00

ATOM 75 N ASP A 11 19.511 10.023 -15.178 1.00 0.00

ATOM 76 CA ASP A 11 18.838 8.752 -15.413 1.00 0.00

ATOM 77 C ASP A 11 19.538 7.617 -14.674 1.00 0.00

ATOM 78 O ASP A 11 18.925 6.597 -14.360 1.00 0.00

ATOM 79 CB ASP A 11 18.775 8.447 -16.911 1.00 0.00

ATOM 80 CG ASP A 11 20.135 8.307 -17.583 1.00 0.00

ATOM 81 OD1 ASP A 11 20.897 9.244 -17.537 1.00 0.00

ATOM 82 OD2 ASP A 11 20.462 7.221 -17.996 1.00 0.00

ATOM 83 N THR A 12 20.825 7.798 -14.401 1.00 0.00

ATOM 84 CA THR A 12 21.614 6.785 -13.708 1.00 0.00

ATOM 85 C THR A 12 21.234 6.705 -12.236 1.00 0.00

ATOM 86 O THR A 12 20.979 5.625 -11.707 1.00 0.00

ATOM 87 CB THR A 12 23.124 7.067 -13.823 1.00 0.00

ATOM 88 OG1 THR A 12 23.513 7.045 -15.203 1.00 0.00

ATOM 89 CG2 THR A 12 23.921 6.022 -13.059 1.00 0.00

ATOM 90 N THR A 13 21.202 7.860 -11.574 1.00 0.00

ATOM 91 CA THR A 13 20.787 7.931 -10.176 1.00 0.00

ATOM 92 C THR A 13 19.330 7.525 -10.012 1.00 0.00

ATOM 93 O THR A 13 18.972 6.836 -9.056 1.00 0.00

ATOM 94 CB THR A 13 20.982 9.345 -9.597 1.00 0.00

ATOM 95 OG1 THR A 13 22.370 9.699 -9.652 1.00 0.00

ATOM 96 CG2 THR A 13 20.504 9.401 -8.155 1.00 0.00

ATOM 97 N LEU A 14 18.491 7.953 -10.951 1.00 0.00

ATOM 98 CA LEU A 14 17.077 7.604 -10.929 1.00 0.00

ATOM 99 C LEU A 14 16.878 6.097 -11.019 1.00 0.00

ATOM 100 O LEU A 14 16.062 5.523 -10.299 1.00 0.00

ATOM 101 CB LEU A 14 16.341 8.309 -12.075 1.00 0.00

ATOM 102 CG LEU A 14 16.120 9.815 -11.880 1.00 0.00

ATOM 103 CD1 LEU A 14 15.673 10.451 -13.190 1.00 0.00

ATOM 104 CD2 LEU A 14 15.084 10.041 -10.789 1.00 0.00

ATOM 105 N GLU A 15 17.587 5.478 -11.917 1.00 0.00

ATOM 106 CA GLU A 15 17.508 4.031 -12.088 1.00 0.00

ATOM 107 C GLU A 15 17.949 3.305 -10.824 1.00 0.00

ATOM 108 O GLU A 15 17.269 2.396 -10.347 1.00 0.00

ATOM 109 CB GLU A 15 18.362 3.584 -13.276 1.00 0.00

ATOM 110 CG GLU A 15 18.357 2.082 -13.524 1.00 0.00

ATOM 111 CD GLU A 15 19.322 1.705 -14.614 1.00 0.00

ATOM 112 OE1 GLU A 15 19.885 2.588 -15.215 1.00 0.00

ATOM 113 OE2 GLU A 15 19.581 0.535 -14.771 1.00 0.00

ATOM 114 N GLU A 16 19.098 3.705 -10.290 1.00 0.00

ATOM 115 CA GLU A 16 19.670 3.046 -9.118 1.00 0.00

ATOM 116 C GLU A 16 18.724 3.125 -7.927 1.00 0.00

ATOM 117 O GLU A 16 18.523 2.141 -7.215 1.00 0.00

ATOM 118 CB GLU A 16 21.021 3.669 -8.758 1.00 0.00

ATOM 119 CG GLU A 16 22.150 3.321 -9.718 1.00 0.00

ATOM 120 CD GLU A 16 23.392 4.109 -9.408 1.00 0.00

ATOM 121 OE1 GLU A 16 23.342 4.942 -8.536 1.00 0.00

ATOM 122 OE2 GLU A 16 24.420 3.801 -9.964 1.00 0.00

ATOM 123 N LEU A 17 18.144 4.299 -7.713 1.00 0.00

ATOM 124 CA LEU A 17 17.205 4.504 -6.618 1.00 0.00

ATOM 125 C LEU A 17 15.946 3.668 -6.812 1.00 0.00

ATOM 126 O LEU A 17 15.411 3.102 -5.857 1.00 0.00

ATOM 127 CB LEU A 17 16.847 5.991 -6.498 1.00 0.00

ATOM 128 CG LEU A 17 17.975 6.895 -5.985 1.00 0.00

ATOM 129 CD1 LEU A 17 17.567 8.357 -6.102 1.00 0.00

ATOM 130 CD2 LEU A 17 18.296 6.538 -4.541 1.00 0.00

ATOM 131 N ALA A 18 15.473 3.598 -8.050 1.00 0.00

ATOM 132 CA ALA A 18 14.298 2.797 -8.378 1.00 0.00

ATOM 133 C ALA A 18 14.550 1.317 -8.124 1.00 0.00

ATOM 134 O ALA A 18 13.669 0.600 -7.652 1.00 0.00

ATOM 135 CB ALA A 18 13.888 3.025 -9.825 1.00 0.00

ATOM 136 N ARG A 19 15.759 0.866 -8.438 1.00 0.00

ATOM 137 CA ARG A 19 16.144 -0.522 -8.206 1.00 0.00

ATOM 138 C ARG A 19 16.166 -0.846 -6.718 1.00 0.00

ATOM 139 O ARG A 19 15.722 -1.915 -6.298 1.00 0.00

ATOM 140 CB ARG A 19 17.467 -0.871 -8.872 1.00 0.00

ATOM 141 CG ARG A 19 17.426 -0.920 -10.391 1.00 0.00

ATOM 142 CD ARG A 19 18.723 -1.265 -11.028 1.00 0.00

ATOM 143 NE ARG A 19 18.714 -1.209 -12.481 1.00 0.00

ATOM 144 CZ ARG A 19 18.190 -2.159 -13.280 1.00 0.00

ATOM 145 NH1 ARG A 19 17.667 -3.255 -12.776 1.00 0.00

ATOM 146 NH2 ARG A 19 18.239 -1.972 -14.587 1.00 0.00

ATOM 147 N LYS A 20 16.688 0.082 -5.923 1.00 0.00

ATOM 148 CA LYS A 20 16.744 -0.091 -4.477 1.00 0.00

ATOM 149 C LYS A 20 15.349 -0.140 -3.871 1.00 0.00

ATOM 150 O LYS A 20 15.077 -0.940 -2.976 1.00 0.00

ATOM 151 CB LYS A 20 17.556 1.036 -3.836 1.00 0.00

ATOM 152 CG LYS A 20 19.048 0.987 -4.140 1.00 0.00

ATOM 153 CD LYS A 20 19.771 2.193 -3.556 1.00 0.00

ATOM 154 CE LYS A 20 21.244 2.192 -3.936 1.00 0.00

ATOM 155 NZ LYS A 20 21.955 3.388 -3.407 1.00 0.00

ATOM 156 N ILE A 21 14.464 0.721 -4.368 1.00 0.00

ATOM 157 CA ILE A 21 13.080 0.748 -3.906 1.00 0.00

ATOM 158 C ILE A 21 12.355 -0.544 -4.262 1.00 0.00

ATOM 159 O ILE A 21 11.573 -1.068 -3.465 1.00 0.00

ATOM 160 CB ILE A 21 12.308 1.940 -4.503 1.00 0.00

ATOM 161 CG1 ILE A 21 12.866 3.260 -3.965 1.00 0.00

ATOM 162 CG2 ILE A 21 10.824 1.820 -4.195 1.00 0.00

ATOM 163 CD1 ILE A 21 12.400 4.477 -4.731 1.00 0.00

ATOM 164 N LYS A 22 12.618 -1.056 -5.459 1.00 0.00

ATOM 165 CA LYS A 22 12.065 -2.335 -5.883 1.00 0.00

ATOM 166 C LYS A 22 12.566 -3.471 -5.001 1.00 0.00

ATOM 167 O LYS A 22 11.807 -4.374 -4.647 1.00 0.00

ATOM 168 CB LYS A 22 12.416 -2.611 -7.346 1.00 0.00

ATOM 169 CG LYS A 22 11.870 -3.927 -7.886 1.00 0.00

ATOM 170 CD LYS A 22 12.235 -4.117 -9.351 1.00 0.00

ATOM 171 CE LYS A 22 11.747 -5.460 -9.874 1.00 0.00

ATOM 172 NZ LYS A 22 12.158 -5.691 -11.285 1.00 0.00

ATOM 173 N ASP A 23 13.846 -3.419 -4.649 1.00 0.00

ATOM 174 CA ASP A 23 14.438 -4.420 -3.768 1.00 0.00

ATOM 175 C ASP A 23 13.756 -4.427 -2.405 1.00 0.00

ATOM 176 O ASP A 23 13.544 -5.484 -1.811 1.00 0.00

ATOM 177 CB ASP A 23 15.939 -4.171 -3.603 1.00 0.00

ATOM 178 CG ASP A 23 16.775 -4.533 -4.823 1.00 0.00

ATOM 179 OD1 ASP A 23 16.260 -5.188 -5.698 1.00 0.00

ATOM 180 OD2 ASP A 23 17.863 -4.024 -4.945 1.00 0.00

ATOM 181 N GLU A 24 13.412 -3.242 -1.916 1.00 0.00

ATOM 182 CA GLU A 24 12.657 -3.115 -0.674 1.00 0.00

ATOM 183 C GLU A 24 11.236 -3.633 -0.840 1.00 0.00

ATOM 184 O GLU A 24 10.662 -4.202 0.088 1.00 0.00

ATOM 185 CB GLU A 24 12.634 -1.658 -0.206 1.00 0.00

ATOM 186 CG GLU A 24 13.981 -1.126 0.262 1.00 0.00

ATOM 187 CD GLU A 24 14.559 -1.985 1.351 1.00 0.00

ATOM 188 OE1 GLU A 24 13.875 -2.229 2.316 1.00 0.00

ATOM 189 OE2 GLU A 24 15.641 -2.492 1.170 1.00 0.00

ATOM 190 N GLY A 25 10.673 -3.437 -2.028 1.00 0.00

ATOM 191 CA GLY A 25 9.384 -4.025 -2.372 1.00 0.00

ATOM 192 C GLY A 25 8.289 -2.969 -2.409 1.00 0.00

ATOM 193 O GLY A 25 7.107 -3.283 -2.270 1.00 0.00

ATOM 194 N LEU A 26 8.688 -1.716 -2.599 1.00 0.00

ATOM 195 CA LEU A 26 7.738 -0.613 -2.669 1.00 0.00

ATOM 196 C LEU A 26 7.649 -0.050 -4.082 1.00 0.00

ATOM 197 O LEU A 26 8.457 -0.390 -4.946 1.00 0.00

ATOM 198 CB LEU A 26 8.134 0.490 -1.679 1.00 0.00

ATOM 199 CG LEU A 26 8.286 0.036 -0.222 1.00 0.00

ATOM 200 CD1 LEU A 26 8.718 1.208 0.649 1.00 0.00

ATOM 201 CD2 LEU A 26 6.969 -0.546 0.271 1.00 0.00

ATOM 202 N GLU A 27 6.663 0.812 -4.310 1.00 0.00

ATOM 203 CA GLU A 27 6.436 1.384 -5.632 1.00 0.00

ATOM 204 C GLU A 27 7.314 2.608 -5.861 1.00 0.00

ATOM 205 O GLU A 27 7.576 3.376 -4.935 1.00 0.00

ATOM 206 CB GLU A 27 4.962 1.754 -5.811 1.00 0.00

ATOM 207 CG GLU A 27 4.011 0.566 -5.808 1.00 0.00

ATOM 208 CD GLU A 27 4.290 -0.360 -6.960 1.00 0.00

ATOM 209 OE1 GLU A 27 4.290 0.096 -8.078 1.00 0.00

ATOM 210 OE2 GLU A 27 4.615 -1.498 -6.715 1.00 0.00

ATOM 211 N VAL A 28 7.763 2.787 -7.097 1.00 0.00

ATOM 212 CA VAL A 28 8.610 3.918 -7.452 1.00 0.00

ATOM 213 C VAL A 28 8.174 4.537 -8.774 1.00 0.00

ATOM 214 O VAL A 28 7.824 3.831 -9.716 1.00 0.00

ATOM 215 CB VAL A 28 10.091 3.507 -7.551 1.00 0.00

ATOM 216 CG1 VAL A 28 10.267 2.392 -8.570 1.00 0.00

ATOM 217 CG2 VAL A 28 10.953 4.705 -7.920 1.00 0.00

ATOM 218 N TYR A 29 8.204 5.865 -8.837 1.00 0.00

ATOM 219 CA TYR A 29 7.978 6.578 -10.088 1.00 0.00

ATOM 220 C TYR A 29 9.165 7.466 -10.440 1.00 0.00

ATOM 221 O TYR A 29 9.674 8.203 -9.591 1.00 0.00

ATOM 222 CB TYR A 29 6.702 7.417 -10.002 1.00 0.00

ATOM 223 CG TYR A 29 5.430 6.599 -9.980 1.00 0.00

ATOM 224 CD1 TYR A 29 4.934 6.084 -8.791 1.00 0.00

ATOM 225 CD2 TYR A 29 4.727 6.346 -11.148 1.00 0.00

ATOM 226 CE1 TYR A 29 3.774 5.335 -8.765 1.00 0.00

ATOM 227 CE2 TYR A 29 3.565 5.599 -11.134 1.00 0.00

ATOM 228 CZ TYR A 29 3.091 5.095 -9.940 1.00 0.00

ATOM 229 OH TYR A 29 1.933 4.352 -9.920 1.00 0.00

ATOM 230 N ILE A 30 9.605 7.392 -11.690 1.00 0.00

ATOM 231 CA ILE A 30 10.771 8.141 -12.140 1.00 0.00

ATOM 232 C ILE A 30 10.358 9.357 -12.961 1.00 0.00

ATOM 233 O ILE A 30 10.001 9.236 -14.133 1.00 0.00

ATOM 234 CB ILE A 30 11.718 7.263 -12.978 1.00 0.00

ATOM 235 CG1 ILE A 30 12.220 6.078 -12.150 1.00 0.00

ATOM 236 CG2 ILE A 30 12.886 8.087 -13.498 1.00 0.00

ATOM 237 CD1 ILE A 30 12.991 5.055 -12.952 1.00 0.00

ATOM 238 N LEU A 31 10.413 10.532 -12.339 1.00 0.00

ATOM 239 CA LEU A 31 10.101 11.779 -13.027 1.00 0.00

ATOM 240 C LEU A 31 11.362 12.453 -13.546 1.00 0.00

ATOM 241 O LEU A 31 12.140 13.014 -12.776 1.00 0.00

ATOM 242 CB LEU A 31 9.338 12.724 -12.091 1.00 0.00

ATOM 243 CG LEU A 31 7.812 12.558 -12.098 1.00 0.00

ATOM 244 CD1 LEU A 31 7.442 11.123 -11.750 1.00 0.00

ATOM 245 CD2 LEU A 31 7.191 13.531 -11.107 1.00 0.00

ATOM 246 N LEU A 32 11.561 12.394 -14.860 1.00 0.00

ATOM 247 CA LEU A 32 12.781 12.906 -15.473 1.00 0.00

ATOM 248 C LEU A 32 12.572 14.317 -16.013 1.00 0.00

ATOM 249 O LEU A 32 11.825 14.523 -16.969 1.00 0.00

ATOM 250 CB LEU A 32 13.246 11.968 -16.593 1.00 0.00

ATOM 251 CG LEU A 32 14.552 12.376 -17.287 1.00 0.00

ATOM 252 CD1 LEU A 32 15.704 12.343 -16.292 1.00 0.00

ATOM 253 CD2 LEU A 32 14.822 11.440 -18.456 1.00 0.00

ATOM 254 N LYS A 33 13.239 15.287 -15.395 1.00 0.00

ATOM 255 CA LYS A 33 13.109 16.680 -15.794 1.00 0.00

ATOM 256 C LYS A 33 14.232 17.097 -16.732 1.00 0.00

ATOM 257 O LYS A 33 15.409 16.867 -16.448 1.00 0.00

ATOM 258 CB LYS A 33 13.088 17.588 -14.563 1.00 0.00

ATOM 259 CG LYS A 33 12.895 19.066 -14.875 1.00 0.00

ATOM 260 CD LYS A 33 12.763 19.888 -13.601 1.00 0.00

ATOM 261 CE LYS A 33 12.616 21.370 -13.911 1.00 0.00

ATOM 262 NZ LYS A 33 12.529 22.191 -12.673 1.00 0.00

ATOM 263 N ASP A 34 13.865 17.710 -17.852 1.00 0.00

ATOM 264 CA ASP A 34 14.843 18.175 -18.829 1.00 0.00

ATOM 265 C ASP A 34 14.242 19.220 -19.757 1.00 0.00

ATOM 266 O ASP A 34 13.019 19.353 -19.849 1.00 0.00

ATOM 267 CB ASP A 34 15.387 16.999 -19.645 1.00 0.00

ATOM 268 CG ASP A 34 16.735 17.261 -20.302 1.00 0.00

ATOM 269 OD1 ASP A 34 17.272 18.325 -20.109 1.00 0.00

ATOM 270 OD2 ASP A 34 17.289 16.343 -20.860 1.00 0.00

ATOM 271 N LYS A 35 15.103 19.959 -20.445 1.00 0.00

ATOM 272 CA LYS A 35 14.657 20.971 -21.396 1.00 0.00

ATOM 273 C LYS A 35 14.868 20.513 -22.832 1.00 0.00

ATOM 274 O LYS A 35 14.124 20.898 -23.733 1.00 0.00

ATOM 275 CB LYS A 35 15.388 22.292 -21.153 1.00 0.00

ATOM 276 CG LYS A 35 15.189 22.875 -19.760 1.00 0.00

ATOM 277 CD LYS A 35 13.736 23.257 -19.522 1.00 0.00

ATOM 278 CE LYS A 35 13.555 23.931 -18.170 1.00 0.00

ATOM 279 NZ LYS A 35 12.132 24.283 -17.910 1.00 0.00

ATOM 280 N ASP A 36 15.888 19.689 -23.040 1.00 0.00

ATOM 281 CA ASP A 36 16.131 19.082 -24.344 1.00 0.00

ATOM 282 C ASP A 36 15.181 17.917 -24.592 1.00 0.00

ATOM 283 O ASP A 36 15.259 16.889 -23.924 1.00 0.00

ATOM 284 CB ASP A 36 17.582 18.609 -24.455 1.00 0.00

ATOM 285 CG ASP A 36 17.984 18.133 -25.844 1.00 0.00

ATOM 286 OD1 ASP A 36 17.265 17.346 -26.413 1.00 0.00

ATOM 287 OD2 ASP A 36 18.917 18.675 -26.389 1.00 0.00

ATOM 288 N GLU A 37 14.286 18.088 -25.561 1.00 0.00

ATOM 289 CA GLU A 37 13.226 17.120 -25.801 1.00 0.00

ATOM 290 C GLU A 37 13.790 15.796 -26.306 1.00 0.00

ATOM 291 O GLU A 37 13.302 14.722 -25.949 1.00 0.00

ATOM 292 CB GLU A 37 12.212 17.676 -26.804 1.00 0.00

ATOM 293 CG GLU A 37 11.349 18.806 -26.262 1.00 0.00

ATOM 294 CD GLU A 37 10.460 19.375 -27.333 1.00 0.00

ATOM 295 OE1 GLU A 37 10.576 18.957 -28.460 1.00 0.00

ATOM 296 OE2 GLU A 37 9.587 20.145 -27.006 1.00 0.00

ATOM 297 N LYS A 38 14.821 15.882 -27.141 1.00 0.00

ATOM 298 CA LYS A 38 15.458 14.692 -27.693 1.00 0.00

ATOM 299 C LYS A 38 16.134 13.872 -26.602 1.00 0.00

ATOM 300 O LYS A 38 15.847 12.689 -26.430 1.00 0.00

ATOM 301 CB LYS A 38 16.476 15.079 -28.767 1.00 0.00

ATOM 302 CG LYS A 38 17.196 13.899 -29.405 1.00 0.00

ATOM 303 CD LYS A 38 18.178 14.361 -30.472 1.00 0.00

ATOM 304 CE LYS A 38 18.927 13.185 -31.082 1.00 0.00

ATOM 305 NZ LYS A 38 19.933 13.627 -32.085 1.00 0.00

ATOM 306 N ARG A 39 17.040 14.510 -25.864 1.00 0.00

ATOM 307 CA ARG A 39 17.724 13.859 -24.754 1.00 0.00

ATOM 308 C ARG A 39 16.726 13.353 -23.717 1.00 0.00

ATOM 309 O ARG A 39 16.883 12.258 -23.177 1.00 0.00

ATOM 310 CB ARG A 39 18.779 14.756 -24.124 1.00 0.00

ATOM 311 CG ARG A 39 19.564 14.119 -22.988 1.00 0.00

ATOM 312 CD ARG A 39 20.583 15.008 -22.375 1.00 0.00

ATOM 313 NE ARG A 39 20.038 16.182 -21.712 1.00 0.00

ATOM 314 CZ ARG A 39 20.751 17.280 -21.393 1.00 0.00

ATOM 315 NH1 ARG A 39 22.041 17.346 -21.638 1.00 0.00

ATOM 316 NH2 ARG A 39 20.122 18.282 -20.803 1.00 0.00

ATOM 317 N LEU A 40 15.703 14.154 -23.444 1.00 0.00

ATOM 318 CA LEU A 40 14.694 13.800 -22.455 1.00 0.00

ATOM 319 C LEU A 40 14.008 12.488 -22.812 1.00 0.00

ATOM 320 O LEU A 40 14.020 11.536 -22.033 1.00 0.00

ATOM 321 CB LEU A 40 13.659 14.924 -22.327 1.00 0.00

ATOM 322 CG LEU A 40 12.484 14.627 -21.387 1.00 0.00

ATOM 323 CD1 LEU A 40 13.003 14.236 -20.010 1.00 0.00

ATOM 324 CD2 LEU A 40 11.583 15.850 -21.296 1.00 0.00

ATOM 325 N GLU A 41 13.411 12.444 -24.000 1.00 0.00

ATOM 326 CA GLU A 41 12.657 11.273 -24.434 1.00 0.00

ATOM 327 C GLU A 41 13.566 10.064 -24.599 1.00 0.00

ATOM 328 O GLU A 41 13.166 8.932 -24.326 1.00 0.00

ATOM 329 CB GLU A 41 11.925 11.563 -25.746 1.00 0.00

ATOM 330 CG GLU A 41 10.737 12.505 -25.609 1.00 0.00

ATOM 331 CD GLU A 41 10.077 12.748 -26.938 1.00 0.00

ATOM 332 OE1 GLU A 41 10.591 12.288 -27.929 1.00 0.00

ATOM 333 OE2 GLU A 41 9.000 13.297 -26.952 1.00 0.00

ATOM 334 N GLU A 42 14.794 10.307 -25.047 1.00 0.00

ATOM 335 CA GLU A 42 15.789 9.249 -25.175 1.00 0.00

ATOM 336 C GLU A 42 16.020 8.549 -23.843 1.00 0.00

ATOM 337 O GLU A 42 15.984 7.322 -23.762 1.00 0.00

ATOM 338 CB GLU A 42 17.108 9.814 -25.708 1.00 0.00

ATOM 339 CG GLU A 42 18.218 8.784 -25.860 1.00 0.00

ATOM 340 CD GLU A 42 19.480 9.413 -26.380 1.00 0.00

ATOM 341 OE1 GLU A 42 19.471 10.593 -26.637 1.00 0.00

ATOM 342 OE2 GLU A 42 20.484 8.742 -26.415 1.00 0.00

ATOM 343 N LYS A 43 16.256 9.337 -22.800 1.00 0.00

ATOM 344 CA LYS A 43 16.539 8.791 -21.476 1.00 0.00

ATOM 345 C LYS A 43 15.293 8.159 -20.864 1.00 0.00

ATOM 346 O LYS A 43 15.384 7.187 -20.117 1.00 0.00

ATOM 347 CB LYS A 43 17.085 9.881 -20.552 1.00 0.00

ATOM 348 CG LYS A 43 18.514 10.309 -20.860 1.00 0.00

ATOM 349 CD LYS A 43 19.010 11.341 -19.858 1.00 0.00

ATOM 350 CE LYS A 43 20.445 11.752 -20.151 1.00 0.00

ATOM 351 NZ LYS A 43 21.388 10.607 -20.030 1.00 0.00

ATOM 352 N ILE A 44 14.133 8.721 -21.188 1.00 0.00

ATOM 353 CA ILE A 44 12.863 8.160 -20.744 1.00 0.00

ATOM 354 C ILE A 44 12.677 6.737 -21.256 1.00 0.00

ATOM 355 O ILE A 44 12.270 5.847 -20.510 1.00 0.00

ATOM 356 CB ILE A 44 11.673 9.022 -21.206 1.00 0.00

ATOM 357 CG1 ILE A 44 11.628 10.335 -20.421 1.00 0.00

ATOM 358 CG2 ILE A 44 10.368 8.256 -21.046 1.00 0.00

ATOM 359 CD1 ILE A 44 10.663 11.352 -20.988 1.00 0.00

ATOM 360 N GLN A 45 12.984 6.531 -22.533 1.00 0.00

ATOM 361 CA GLN A 45 12.900 5.204 -23.135 1.00 0.00

ATOM 362 C GLN A 45 13.910 4.252 -22.505 1.00 0.00

ATOM 363 O GLN A 45 13.599 3.090 -22.242 1.00 0.00

ATOM 364 CB GLN A 45 13.137 5.283 -24.646 1.00 0.00

ATOM 365 CG GLN A 45 12.001 5.928 -25.421 1.00 0.00

ATOM 366 CD GLN A 45 12.345 6.140 -26.883 1.00 0.00

ATOM 367 OE1 GLN A 45 13.471 5.878 -27.314 1.00 0.00

ATOM 368 NE2 GLN A 45 11.376 6.621 -27.654 1.00 0.00

ATOM 369 N LYS A 46 15.117 4.748 -22.267 1.00 0.00

ATOM 370 CA LYS A 46 16.161 3.954 -21.627 1.00 0.00

ATOM 371 C LYS A 46 15.765 3.560 -20.210 1.00 0.00

ATOM 372 O LYS A 46 16.084 2.464 -19.749 1.00 0.00

ATOM 373 CB LYS A 46 17.482 4.724 -21.609 1.00 0.00

ATOM 374 CG LYS A 46 18.143 4.873 -22.973 1.00 0.00

ATOM 375 CD LYS A 46 19.415 5.703 -22.884 1.00 0.00

ATOM 376 CE LYS A 46 20.100 5.814 -24.238 1.00 0.00

ATOM 377 NZ LYS A 46 21.311 6.676 -24.180 1.00 0.00

ATOM 378 N LEU A 47 15.067 4.458 -19.526 1.00 0.00

ATOM 379 CA LEU A 47 14.604 4.199 -18.168 1.00 0.00

ATOM 380 C LEU A 47 13.450 3.206 -18.163 1.00 0.00

ATOM 381 O LEU A 47 13.389 2.311 -17.319 1.00 0.00

ATOM 382 CB LEU A 47 14.184 5.510 -17.491 1.00 0.00

ATOM 383 CG LEU A 47 15.338 6.443 -17.102 1.00 0.00

ATOM 384 CD1 LEU A 47 14.803 7.834 -16.792 1.00 0.00

ATOM 385 CD2 LEU A 47 16.073 5.868 -15.900 1.00 0.00

ATOM 386 N LYS A 48 12.532 3.368 -19.112 1.00 0.00

ATOM 387 CA LYS A 48 11.361 2.499 -19.203 1.00 0.00

ATOM 388 C LYS A 48 11.767 1.066 -19.525 1.00 0.00

ATOM 389 O LYS A 48 11.122 0.115 -19.085 1.00 0.00

ATOM 390 CB LYS A 48 10.387 3.020 -20.261 1.00 0.00

ATOM 391 CG LYS A 48 9.572 4.230 -19.824 1.00 0.00

ATOM 392 CD LYS A 48 8.612 4.674 -20.917 1.00 0.00

ATOM 393 CE LYS A 48 7.675 5.766 -20.421 1.00 0.00

ATOM 394 NZ LYS A 48 6.736 6.219 -21.484 1.00 0.00

ATOM 395 N SER A 49 12.838 0.915 -20.295 1.00 0.00

ATOM 396 CA SER A 49 13.328 -0.402 -20.680 1.00 0.00

ATOM 397 C SER A 49 13.872 -1.161 -19.478 1.00 0.00

ATOM 398 O SER A 49 14.096 -2.370 -19.543 1.00 0.00

ATOM 399 CB SER A 49 14.396 -0.270 -21.748 1.00 0.00

ATOM 400 OG SER A 49 15.609 0.199 -21.225 1.00 0.00

ATOM 401 N GLN A 50 14.085 -0.444 -18.380 1.00 0.00

ATOM 402 CA GLN A 50 14.583 -1.053 -17.150 1.00 0.00

ATOM 403 C GLN A 50 13.460 -1.745 -16.385 1.00 0.00

ATOM 404 O GLN A 50 13.711 -2.595 -15.531 1.00 0.00

ATOM 405 CB GLN A 50 15.241 0.002 -16.257 1.00 0.00

ATOM 406 CG GLN A 50 16.375 0.762 -16.924 1.00 0.00

ATOM 407 CD GLN A 50 17.337 -0.155 -17.654 1.00 0.00

ATOM 408 OE1 GLN A 50 17.799 -1.158 -17.102 1.00 0.00

ATOM 409 NE2 GLN A 50 17.650 0.185 -18.899 1.00 0.00

ATOM 410 N GLY A 51 12.225 -1.377 -16.698 1.00 0.00

ATOM 411 CA GLY A 51 11.061 -2.003 -16.083 1.00 0.00

ATOM 412 C GLY A 51 10.496 -1.137 -14.964 1.00 0.00

ATOM 413 O GLY A 51 9.939 -1.648 -13.993 1.00 0.00

ATOM 414 N PHE A 52 10.645 0.176 -15.107 1.00 0.00

ATOM 415 CA PHE A 52 10.115 1.115 -14.126 1.00 0.00

ATOM 416 C PHE A 52 9.199 2.139 -14.784 1.00 0.00

ATOM 417 O PHE A 52 9.292 2.384 -15.988 1.00 0.00

ATOM 418 CB PHE A 52 11.257 1.823 -13.394 1.00 0.00

ATOM 419 CG PHE A 52 12.158 0.893 -12.633 1.00 0.00

ATOM 420 CD1 PHE A 52 13.418 0.575 -13.117 1.00 0.00

ATOM 421 CD2 PHE A 52 11.747 0.333 -11.433 1.00 0.00

ATOM 422 CE1 PHE A 52 14.248 -0.281 -12.417 1.00 0.00

ATOM 423 CE2 PHE A 52 12.575 -0.522 -10.731 1.00 0.00

ATOM 424 CZ PHE A 52 13.827 -0.829 -11.225 1.00 0.00

ATOM 425 N GLU A 53 8.311 2.730 -13.990 1.00 0.00

ATOM 426 CA GLU A 53 7.429 3.784 -14.478 1.00 0.00

ATOM 427 C GLU A 53 8.176 5.102 -14.621 1.00 0.00

ATOM 428 O GLU A 53 8.845 5.554 -13.692 1.00 0.00

ATOM 429 CB GLU A 53 6.231 3.958 -13.542 1.00 0.00

ATOM 430 CG GLU A 53 5.175 2.869 -13.663 1.00 0.00

ATOM 431 CD GLU A 53 4.556 2.858 -15.034 1.00 0.00

ATOM 432 OE1 GLU A 53 4.083 3.884 -15.460 1.00 0.00

ATOM 433 OE2 GLU A 53 4.656 1.855 -15.701 1.00 0.00

ATOM 434 N VAL A 54 8.056 5.719 -15.792 1.00 0.00

ATOM 435 CA VAL A 54 8.790 6.941 -16.093 1.00 0.00

ATOM 436 C VAL A 54 7.854 8.034 -16.605 1.00 0.00

ATOM 437 O VAL A 54 6.929 7.764 -17.367 1.00 0.00

ATOM 438 CB VAL A 54 9.895 6.693 -17.136 1.00 0.00

ATOM 439 CG1 VAL A 54 10.713 7.957 -17.357 1.00 0.00

ATOM 440 CG2 VAL A 54 10.796 5.548 -16.696 1.00 0.00

ATOM 441 N ARG A 55 8.108 9.268 -16.178 1.00 0.00

ATOM 442 CA ARG A 55 7.254 10.392 -16.546 1.00 0.00

ATOM 443 C ARG A 55 8.068 11.518 -17.165 1.00 0.00

ATOM 444 O ARG A 55 9.210 11.764 -16.773 1.00 0.00

ATOM 445 CB ARG A 55 6.420 10.888 -15.374 1.00 0.00

ATOM 446 CG ARG A 55 5.479 9.853 -14.776 1.00 0.00

ATOM 447 CD ARG A 55 4.397 9.409 -15.691 1.00 0.00

ATOM 448 NE ARG A 55 3.506 8.406 -15.129 1.00 0.00

ATOM 449 CZ ARG A 55 3.692 7.076 -15.231 1.00 0.00

ATOM 450 NH1 ARG A 55 4.709 6.582 -15.903 1.00 0.00

ATOM 451 NH2 ARG A 55 2.809 6.276 -14.658 1.00 0.00

ATOM 452 N LYS A 56 7.473 12.208 -18.135 1.00 0.00

ATOM 453 CA LYS A 56 8.179 13.240 -18.885 1.00 0.00

ATOM 454 C LYS A 56 7.950 14.616 -18.277 1.00 0.00

ATOM 455 O LYS A 56 6.836 15.139 -18.301 1.00 0.00

ATOM 456 CB LYS A 56 7.739 13.233 -20.350 1.00 0.00

ATOM 457 CG LYS A 56 8.493 14.214 -21.238 1.00 0.00

ATOM 458 CD LYS A 56 7.997 14.154 -22.675 1.00 0.00

ATOM 459 CE LYS A 56 8.773 15.110 -23.569 1.00 0.00

ATOM 460 NZ LYS A 56 8.230 15.139 -24.955 1.00 0.00

ATOM 461 N VAL A 57 9.012 15.201 -17.730 1.00 0.00

ATOM 462 CA VAL A 57 8.916 16.491 -17.061 1.00 0.00

ATOM 463 C VAL A 57 9.667 17.570 -17.830 1.00 0.00

ATOM 464 O VAL A 57 10.856 17.426 -18.119 1.00 0.00

ATOM 465 CB VAL A 57 9.465 16.423 -15.623 1.00 0.00

ATOM 466 CG1 VAL A 57 9.432 17.799 -14.975 1.00 0.00

ATOM 467 CG2 VAL A 57 8.668 15.427 -14.795 1.00 0.00

ATOM 468 N LYS A 58 8.968 18.650 -18.162 1.00 0.00

ATOM 469 CA LYS A 58 9.603 19.821 -18.754 1.00 0.00

ATOM 470 C LYS A 58 9.695 20.964 -17.750 1.00 0.00

ATOM 471 O LYS A 58 10.548 21.843 -17.872 1.00 0.00

ATOM 472 CB LYS A 58 8.836 20.274 -19.997 1.00 0.00

ATOM 473 CG LYS A 58 8.844 19.269 -21.142 1.00 0.00

ATOM 474 CD LYS A 58 8.176 19.840 -22.384 1.00 0.00

ATOM 475 CE LYS A 58 8.183 18.836 -23.528 1.00 0.00

ATOM 476 NZ LYS A 58 7.514 19.373 -24.743 1.00 0.00

ATOM 477 N ASP A 59 8.809 20.948 -16.759 1.00 0.00

ATOM 478 CA ASP A 59 8.754 22.011 -15.767 1.00 0.00

ATOM 479 C ASP A 59 8.123 21.522 -14.467 1.00 0.00

ATOM 480 O ASP A 59 7.585 20.419 -14.406 1.00 0.00

ATOM 481 CB ASP A 59 7.975 23.211 -16.313 1.00 0.00

ATOM 482 CG ASP A 59 8.340 24.543 -15.672 1.00 0.00

ATOM 483 OD1 ASP A 59 9.189 24.553 -14.812 1.00 0.00

ATOM 484 OD2 ASP A 59 7.887 25.554 -16.151 1.00 0.00

ATOM 485 N ASP A 60 8.195 22.350 -13.433 1.00 0.00

ATOM 486 CA ASP A 60 7.662 21.989 -12.124 1.00 0.00

ATOM 487 C ASP A 60 6.156 21.760 -12.187 1.00 0.00

ATOM 488 O ASP A 60 5.604 20.985 -11.408 1.00 0.00

ATOM 489 CB ASP A 60 7.985 23.075 -11.094 1.00 0.00

ATOM 490 CG ASP A 60 9.449 23.130 -10.678 1.00 0.00

ATOM 491 OD1 ASP A 60 10.173 22.218 -11.001 1.00 0.00

ATOM 492 OD2 ASP A 60 9.866 24.148 -10.180 1.00 0.00

ATOM 493 N ASP A 61 5.498 22.438 -13.123 1.00 0.00

ATOM 494 CA ASP A 61 4.084 22.208 -13.379 1.00 0.00

ATOM 495 C ASP A 61 3.817 20.752 -13.741 1.00 0.00

ATOM 496 O ASP A 61 2.843 20.153 -13.280 1.00 0.00

ATOM 497 CB ASP A 61 3.587 23.128 -14.497 1.00 0.00

ATOM 498 CG ASP A 61 3.440 24.588 -14.091 1.00 0.00

ATOM 499 OD1 ASP A 61 3.492 24.866 -12.917 1.00 0.00

ATOM 500 OD2 ASP A 61 3.435 25.427 -14.960 1.00 0.00

ATOM 501 N ASP A 62 4.689 20.186 -14.571 1.00 0.00

ATOM 502 CA ASP A 62 4.568 18.789 -14.970 1.00 0.00

ATOM 503 C ASP A 62 4.757 17.860 -13.779 1.00 0.00

ATOM 504 O ASP A 62 4.053 16.857 -13.643 1.00 0.00

ATOM 505 CB ASP A 62 5.583 18.452 -16.065 1.00 0.00

ATOM 506 CG ASP A 62 5.261 19.052 -17.427 1.00 0.00

ATOM 507 OD1 ASP A 62 4.161 19.519 -17.604 1.00 0.00

ATOM 508 OD2 ASP A 62 6.159 19.181 -18.224 1.00 0.00

ATOM 509 N ILE A 63 5.708 18.198 -12.914 1.00 0.00

ATOM 510 CA ILE A 63 5.957 17.421 -11.707 1.00 0.00

ATOM 511 C ILE A 63 4.701 17.312 -10.851 1.00 0.00

ATOM 512 O ILE A 63 4.334 16.226 -10.405 1.00 0.00

ATOM 513 CB ILE A 63 7.091 18.034 -10.865 1.00 0.00

ATOM 514 CG1 ILE A 63 8.425 17.929 -11.608 1.00 0.00

ATOM 515 CG2 ILE A 63 7.177 17.349 -9.510 1.00 0.00

ATOM 516 CD1 ILE A 63 9.540 18.732 -10.977 1.00 0.00

ATOM 517 N ASP A 64 4.046 18.447 -10.625 1.00 0.00

ATOM 518 CA ASP A 64 2.834 18.485 -9.816 1.00 0.00

ATOM 519 C ASP A 64 1.711 17.691 -10.468 1.00 0.00

ATOM 520 O ASP A 64 0.957 16.991 -9.792 1.00 0.00

ATOM 521 CB ASP A 64 2.389 19.930 -9.583 1.00 0.00

ATOM 522 CG ASP A 64 3.267 20.711 -8.614 1.00 0.00

ATOM 523 OD1 ASP A 64 4.069 20.101 -7.946 1.00 0.00

ATOM 524 OD2 ASP A 64 3.243 21.917 -8.661 1.00 0.00

ATOM 525 N LYS A 65 1.602 17.805 -11.788 1.00 0.00

ATOM 526 CA LYS A 65 0.573 17.096 -12.536 1.00 0.00

ATOM 527 C LYS A 65 0.771 15.588 -12.449 1.00 0.00

ATOM 528 O LYS A 65 -0.197 14.834 -12.319 1.00 0.00

ATOM 529 CB LYS A 65 0.570 17.543 -13.999 1.00 0.00

ATOM 530 CG LYS A 65 0.027 18.948 -14.227 1.00 0.00

ATOM 531 CD LYS A 65 0.139 19.354 -15.688 1.00 0.00

ATOM 532 CE LYS A 65 -0.346 20.780 -15.906 1.00 0.00

ATOM 533 NZ LYS A 65 -0.234 21.195 -17.331 1.00 0.00

ATOM 534 N TRP A 66 2.024 15.154 -12.518 1.00 0.00

ATOM 535 CA TRP A 66 2.344 13.730 -12.477 1.00 0.00

ATOM 536 C TRP A 66 2.081 13.146 -11.094 1.00 0.00

ATOM 537 O TRP A 66 1.529 12.055 -10.966 1.00 0.00

ATOM 538 CB TRP A 66 3.803 13.499 -12.874 1.00 0.00

ATOM 539 CG TRP A 66 4.061 13.689 -14.338 1.00 0.00

ATOM 540 CD1 TRP A 66 5.078 14.401 -14.901 1.00 0.00

ATOM 541 CD2 TRP A 66 3.293 13.157 -15.425 1.00 0.00

ATOM 542 NE1 TRP A 66 4.990 14.349 -16.270 1.00 0.00

ATOM 543 CE2 TRP A 66 3.902 13.589 -16.617 1.00 0.00

ATOM 544 CE3 TRP A 66 2.147 12.356 -15.503 1.00 0.00

ATOM 545 CZ2 TRP A 66 3.408 13.252 -17.867 1.00 0.00

ATOM 546 CZ3 TRP A 66 1.653 12.017 -16.758 1.00 0.00

ATOM 547 CH2 TRP A 66 2.266 12.452 -17.906 1.00 0.00

ATOM 548 N ILE A 67 2.481 13.882 -10.061 1.00 0.00

ATOM 549 CA ILE A 67 2.286 13.440 -8.687 1.00 0.00

ATOM 550 C ILE A 67 0.806 13.340 -8.344 1.00 0.00

ATOM 551 O ILE A 67 0.374 12.391 -7.690 1.00 0.00

ATOM 552 CB ILE A 67 2.972 14.389 -7.687 1.00 0.00

ATOM 553 CG1 ILE A 67 4.494 14.296 -7.821 1.00 0.00

ATOM 554 CG2 ILE A 67 2.539 14.067 -6.265 1.00 0.00

ATOM 555 CD1 ILE A 67 5.240 15.385 -7.083 1.00 0.00

ATOM 556 N ASP A 68 0.033 14.325 -8.786 1.00 0.00

ATOM 557 CA ASP A 68 -1.413 14.305 -8.607 1.00 0.00

ATOM 558 C ASP A 68 -2.037 13.099 -9.299 1.00 0.00

ATOM 559 O ASP A 68 -2.871 12.404 -8.718 1.00 0.00

ATOM 560 CB ASP A 68 -2.036 15.598 -9.139 1.00 0.00

ATOM 561 CG ASP A 68 -1.794 16.820 -8.262 1.00 0.00

ATOM 562 OD1 ASP A 68 -1.360 16.650 -7.148 1.00 0.00

ATOM 563 OD2 ASP A 68 -1.895 17.914 -8.763 1.00 0.00

ATOM 564 N LYS A 69 -1.628 12.855 -10.538 1.00 0.00

ATOM 565 CA LYS A 69 -2.112 11.708 -11.295 1.00 0.00

ATOM 566 C LYS A 69 -1.802 10.401 -10.572 1.00 0.00

ATOM 567 O LYS A 69 -2.637 9.498 -10.515 1.00 0.00

ATOM 568 CB LYS A 69 -1.499 11.693 -12.696 1.00 0.00

ATOM 569 CG LYS A 69 -1.985 10.555 -13.583 1.00 0.00

ATOM 570 CD LYS A 69 -1.409 10.664 -14.988 1.00 0.00

ATOM 571 CE LYS A 69 -1.891 9.525 -15.874 1.00 0.00

ATOM 572 NZ LYS A 69 -1.346 9.626 -17.255 1.00 0.00

ATOM 573 N ILE A 70 -0.595 10.307 -10.028 1.00 0.00

ATOM 574 CA ILE A 70 -0.182 9.125 -9.280 1.00 0.00

ATOM 575 C ILE A 70 -1.069 8.903 -8.063 1.00 0.00

ATOM 576 O ILE A 70 -1.470 7.776 -7.772 1.00 0.00

ATOM 577 CB ILE A 70 1.284 9.229 -8.821 1.00 0.00

ATOM 578 CG1 ILE A 70 2.227 9.154 -10.024 1.00 0.00

ATOM 579 CG2 ILE A 70 1.607 8.132 -7.818 1.00 0.00

ATOM 580 CD1 ILE A 70 3.642 9.591 -9.720 1.00 0.00

ATOM 581 N LYS A 71 -1.372 9.985 -7.352 1.00 0.00

ATOM 582 CA LYS A 71 -2.251 9.917 -6.195 1.00 0.00

ATOM 583 C LYS A 71 -3.668 9.530 -6.597 1.00 0.00

ATOM 584 O LYS A 71 -4.369 8.844 -5.854 1.00 0.00

ATOM 585 CB LYS A 71 -2.262 11.256 -5.455 1.00 0.00

ATOM 586 CG LYS A 71 -0.975 11.569 -4.703 1.00 0.00

ATOM 587 CD LYS A 71 -1.052 12.925 -4.016 1.00 0.00

ATOM 588 CE LYS A 71 0.208 13.211 -3.213 1.00 0.00

ATOM 589 NZ LYS A 71 0.155 14.542 -2.550 1.00 0.00

ATOM 590 N LYS A 72 -4.085 9.972 -7.778 1.00 0.00

ATOM 591 CA LYS A 72 -5.402 9.635 -8.300 1.00 0.00

ATOM 592 C LYS A 72 -5.477 8.168 -8.702 1.00 0.00

ATOM 593 O LYS A 72 -6.515 7.525 -8.547 1.00 0.00

ATOM 594 CB LYS A 72 -5.749 10.526 -9.495 1.00 0.00

ATOM 595 CG LYS A 72 -6.050 11.974 -9.132 1.00 0.00

ATOM 596 CD LYS A 72 -6.300 12.815 -10.376 1.00 0.00

ATOM 597 CE LYS A 72 -6.554 14.272 -10.018 1.00 0.00

ATOM 598 NZ LYS A 72 -6.806 15.106 -11.224 1.00 0.00

ATOM 599 N GLU A 73 -4.372 7.646 -9.218 1.00 0.00

ATOM 600 CA GLU A 73 -4.298 6.241 -9.604 1.00 0.00

ATOM 601 C GLU A 73 -4.210 5.338 -8.380 1.00 0.00

ATOM 602 O GLU A 73 -4.754 4.234 -8.372 1.00 0.00

ATOM 603 CB GLU A 73 -3.099 6.000 -10.524 1.00 0.00

ATOM 604 CG GLU A 73 -3.254 6.578 -11.924 1.00 0.00

ATOM 605 CD GLU A 73 -2.003 6.385 -12.736 1.00 0.00

ATOM 606 OE1 GLU A 73 -1.039 5.896 -12.199 1.00 0.00

ATOM 607 OE2 GLU A 73 -2.046 6.621 -13.920 1.00 0.00

ATOM 608 N ARG A 74 -3.524 5.814 -7.346 1.00 0.00

ATOM 609 CA ARG A 74 -3.339 5.039 -6.127 1.00 0.00

ATOM 610 C ARG A 74 -3.587 5.893 -4.889 1.00 0.00

ATOM 611 O ARG A 74 -2.651 6.258 -4.179 1.00 0.00

ATOM 612 CB ARG A 74 -1.975 4.366 -6.076 1.00 0.00

ATOM 613 CG ARG A 74 -1.734 3.328 -7.161 1.00 0.00

ATOM 614 CD ARG A 74 -2.568 2.106 -7.036 1.00 0.00

ATOM 615 NE ARG A 74 -2.269 1.070 -8.011 1.00 0.00

ATOM 616 CZ ARG A 74 -2.807 1.001 -9.244 1.00 0.00

ATOM 617 NH1 ARG A 74 -3.698 1.881 -9.645 1.00 0.00

ATOM 618 NH2 ARG A 74 -2.433 0.010 -10.035 1.00 0.00

ATOM 619 N PRO A 75 -4.855 6.205 -4.635 1.00 0.00

ATOM 620 CA PRO A 75 -5.220 7.072 -3.525 1.00 0.00

ATOM 621 C PRO A 75 -5.107 6.337 -2.193 1.00 0.00

ATOM 622 O PRO A 75 -5.108 6.958 -1.129 1.00 0.00

ATOM 623 CB PRO A 75 -6.661 7.488 -3.836 1.00 0.00

ATOM 624 CG PRO A 75 -7.205 6.365 -4.652 1.00 0.00

ATOM 625 CD PRO A 75 -6.045 5.874 -5.477 1.00 0.00

ATOM 626 N GLN A 76 -5.016 5.012 -2.258 1.00 0.00

ATOM 627 CA GLN A 76 -4.921 4.190 -1.058 1.00 0.00

ATOM 628 C GLN A 76 -3.469 3.927 -0.684 1.00 0.00

ATOM 629 O GLN A 76 -3.186 3.326 0.354 1.00 0.00

ATOM 630 CB GLN A 76 -5.651 2.860 -1.262 1.00 0.00

ATOM 631 CG GLN A 76 -7.128 3.003 -1.586 1.00 0.00

ATOM 632 CD GLN A 76 -7.894 3.719 -0.489 1.00 0.00

ATOM 633 OE1 GLN A 76 -7.753 3.403 0.694 1.00 0.00

ATOM 634 NE2 GLN A 76 -8.715 4.689 -0.879 1.00 0.00

ATOM 635 N LEU A 77 -2.553 4.378 -1.530 1.00 0.00

ATOM 636 CA LEU A 77 -1.127 4.180 -1.296 1.00 0.00

ATOM 637 C LEU A 77 -0.459 5.471 -0.840 1.00 0.00

ATOM 638 O LEU A 77 -0.830 6.560 -1.278 1.00 0.00

ATOM 639 CB LEU A 77 -0.450 3.649 -2.566 1.00 0.00

ATOM 640 CG LEU A 77 -0.543 2.131 -2.769 1.00 0.00

ATOM 641 CD1 LEU A 77 -1.986 1.729 -3.042 1.00 0.00

ATOM 642 CD2 LEU A 77 0.360 1.715 -3.921 1.00 0.00

ATOM 643 N GLU A 78 0.529 5.341 0.039 1.00 0.00

ATOM 644 CA GLU A 78 1.203 6.500 0.608 1.00 0.00

ATOM 645 C GLU A 78 2.205 7.094 -0.376 1.00 0.00

ATOM 646 O GLU A 78 3.350 6.655 -0.447 1.00 0.00

ATOM 647 CB GLU A 78 1.908 6.120 1.912 1.00 0.00

ATOM 648 CG GLU A 78 0.969 5.713 3.039 1.00 0.00

ATOM 649 CD GLU A 78 0.259 6.905 3.617 1.00 0.00

ATOM 650 OE1 GLU A 78 0.579 8.005 3.236 1.00 0.00

ATOM 651 OE2 GLU A 78 -0.679 6.713 4.355 1.00 0.00

ATOM 652 N VAL A 79 1.765 8.095 -1.127 1.00 0.00

ATOM 653 CA VAL A 79 2.631 8.774 -2.087 1.00 0.00

ATOM 654 C VAL A 79 3.502 9.815 -1.396 1.00 0.00

ATOM 655 O VAL A 79 3.006 10.646 -0.637 1.00 0.00

ATOM 656 CB VAL A 79 1.815 9.458 -3.200 1.00 0.00

ATOM 657 CG1 VAL A 79 2.736 10.213 -4.147 1.00 0.00

ATOM 658 CG2 VAL A 79 0.993 8.433 -3.964 1.00 0.00

ATOM 659 N ARG A 80 4.802 9.763 -1.667 1.00 0.00

ATOM 660 CA ARG A 80 5.734 10.752 -1.137 1.00 0.00

ATOM 661 C ARG A 80 6.560 11.384 -2.251 1.00 0.00

ATOM 662 O ARG A 80 6.937 10.718 -3.215 1.00 0.00

ATOM 663 CB ARG A 80 6.622 10.177 -0.044 1.00 0.00

ATOM 664 CG ARG A 80 5.881 9.681 1.188 1.00 0.00

ATOM 665 CD ARG A 80 5.203 10.750 1.965 1.00 0.00

ATOM 666 NE ARG A 80 4.514 10.281 3.157 1.00 0.00

ATOM 667 CZ ARG A 80 3.259 9.791 3.172 1.00 0.00

ATOM 668 NH1 ARG A 80 2.539 9.738 2.074 1.00 0.00

ATOM 669 NH2 ARG A 80 2.760 9.388 4.328 1.00 0.00

ATOM 670 N LYS A 81 6.834 12.677 -2.116 1.00 0.00

ATOM 671 CA LYS A 81 7.609 13.408 -3.112 1.00 0.00

ATOM 672 C LYS A 81 9.087 13.440 -2.751 1.00 0.00

ATOM 673 O LYS A 81 9.454 13.786 -1.628 1.00 0.00

ATOM 674 CB LYS A 81 7.075 14.833 -3.264 1.00 0.00

ATOM 675 CG LYS A 81 7.782 15.659 -4.331 1.00 0.00

ATOM 676 CD LYS A 81 7.184 17.054 -4.437 1.00 0.00

ATOM 677 CE LYS A 81 7.873 17.871 -5.520 1.00 0.00

ATOM 678 NZ LYS A 81 7.278 19.228 -5.656 1.00 0.00

ATOM 679 N VAL A 82 9.936 13.079 -3.710 1.00 0.00

ATOM 680 CA VAL A 82 11.376 13.076 -3.497 1.00 0.00

ATOM 681 C VAL A 82 12.088 13.928 -4.537 1.00 0.00

ATOM 682 O VAL A 82 11.847 13.789 -5.737 1.00 0.00

ATOM 683 CB VAL A 82 11.950 11.647 -3.537 1.00 0.00

ATOM 684 CG1 VAL A 82 13.470 11.683 -3.481 1.00 0.00

ATOM 685 CG2 VAL A 82 11.395 10.817 -2.390 1.00 0.00

ATOM 686 N THR A 83 12.966 14.810 -4.072 1.00 0.00

ATOM 687 CA THR A 83 13.687 15.714 -4.959 1.00 0.00

ATOM 688 C THR A 83 15.190 15.640 -4.719 1.00 0.00

ATOM 689 O THR A 83 15.930 16.561 -5.064 1.00 0.00

ATOM 690 CB THR A 83 13.221 17.171 -4.782 1.00 0.00

ATOM 691 OG1 THR A 83 13.400 17.570 -3.418 1.00 0.00

ATOM 692 CG2 THR A 83 11.754 17.310 -5.159 1.00 0.00

ATOM 693 N ASP A 84 15.635 14.537 -4.125 1.00 0.00

ATOM 694 CA ASP A 84 17.053 14.333 -3.852 1.00 0.00

ATOM 695 C ASP A 84 17.356 12.865 -3.583 1.00 0.00

ATOM 696 O ASP A 84 16.462 12.087 -3.251 1.00 0.00

ATOM 697 CB ASP A 84 17.498 15.188 -2.662 1.00 0.00

ATOM 698 CG ASP A 84 18.994 15.468 -2.614 1.00 0.00

ATOM 699 OD1 ASP A 84 19.693 15.011 -3.488 1.00 0.00

ATOM 700 OD2 ASP A 84 19.404 16.262 -1.801 1.00 0.00

ATOM 701 N GLU A 85 18.623 12.493 -3.728 1.00 0.00

ATOM 702 CA GLU A 85 19.051 11.120 -3.485 1.00 0.00

ATOM 703 C GLU A 85 18.989 10.778 -2.001 1.00 0.00

ATOM 704 O GLU A 85 18.506 9.712 -1.620 1.00 0.00

ATOM 705 CB GLU A 85 20.468 10.898 -4.017 1.00 0.00

ATOM 706 CG GLU A 85 20.982 9.474 -3.861 1.00 0.00

ATOM 707 CD GLU A 85 22.373 9.332 -4.415 1.00 0.00

ATOM 708 OE1 GLU A 85 22.895 10.298 -4.916 1.00 0.00

ATOM 709 OE2 GLU A 85 22.958 8.289 -4.237 1.00 0.00

ATOM 710 N ASP A 86 19.480 11.690 -1.168 1.00 0.00

ATOM 711 CA ASP A 86 19.407 11.523 0.279 1.00 0.00

ATOM 712 C ASP A 86 17.961 11.508 0.758 1.00 0.00

ATOM 713 O ASP A 86 17.619 10.792 1.699 1.00 0.00

ATOM 714 CB ASP A 86 20.184 12.636 0.986 1.00 0.00

ATOM 715 CG ASP A 86 21.698 12.504 0.892 1.00 0.00

ATOM 716 OD1 ASP A 86 22.161 11.460 0.497 1.00 0.00

ATOM 717 OD2 ASP A 86 22.372 13.492 1.063 1.00 0.00

ATOM 718 N GLN A 87 17.118 12.299 0.106 1.00 0.00

ATOM 719 CA GLN A 87 15.691 12.312 0.406 1.00 0.00

ATOM 720 C GLN A 87 15.031 11.002 -0.003 1.00 0.00

ATOM 721 O GLN A 87 14.130 10.511 0.677 1.00 0.00

ATOM 722 CB GLN A 87 15.003 13.480 -0.305 1.00 0.00

ATOM 723 CG GLN A 87 13.534 13.646 0.046 1.00 0.00

ATOM 724 CD GLN A 87 12.943 14.919 -0.527 1.00 0.00

ATOM 725 OE1 GLN A 87 13.553 15.580 -1.371 1.00 0.00

ATOM 726 NE2 GLN A 87 11.748 15.274 -0.067 1.00 0.00

ATOM 727 N ALA A 88 15.486 10.439 -1.118 1.00 0.00

ATOM 728 CA ALA A 88 15.028 9.126 -1.555 1.00 0.00

ATOM 729 C ALA A 88 15.333 8.060 -0.509 1.00 0.00

ATOM 730 O ALA A 88 14.496 7.208 -0.215 1.00 0.00

ATOM 731 CB ALA A 88 15.662 8.760 -2.889 1.00 0.00

ATOM 732 N LYS A 89 16.538 8.115 0.049 1.00 0.00

ATOM 733 CA LYS A 89 16.946 7.172 1.084 1.00 0.00

ATOM 734 C LYS A 89 16.092 7.323 2.337 1.00 0.00

ATOM 735 O LYS A 89 15.631 6.337 2.909 1.00 0.00

ATOM 736 CB LYS A 89 18.424 7.363 1.430 1.00 0.00

ATOM 737 CG LYS A 89 19.386 6.965 0.318 1.00 0.00

ATOM 738 CD LYS A 89 20.828 7.258 0.704 1.00 0.00

ATOM 739 CE LYS A 89 21.782 6.944 -0.440 1.00 0.00

ATOM 740 NZ LYS A 89 23.193 7.259 -0.089 1.00 0.00

ATOM 741 N GLN A 90 15.887 8.567 2.758 1.00 0.00

ATOM 742 CA GLN A 90 15.103 8.849 3.955 1.00 0.00

ATOM 743 C GLN A 90 13.662 8.386 3.793 1.00 0.00

ATOM 744 O GLN A 90 13.112 7.717 4.669 1.00 0.00

ATOM 745 CB GLN A 90 15.132 10.346 4.273 1.00 0.00

ATOM 746 CG GLN A 90 14.391 10.728 5.543 1.00 0.00

ATOM 747 CD GLN A 90 15.045 10.155 6.787 1.00 0.00

ATOM 748 OE1 GLN A 90 16.268 10.215 6.947 1.00 0.00

ATOM 749 NE2 GLN A 90 14.233 9.593 7.675 1.00 0.00

ATOM 750 N ILE A 91 13.054 8.744 2.667 1.00 0.00

ATOM 751 CA ILE A 91 11.656 8.414 2.412 1.00 0.00

ATOM 752 C ILE A 91 11.463 6.910 2.262 1.00 0.00

ATOM 753 O ILE A 91 10.509 6.342 2.791 1.00 0.00

ATOM 754 CB ILE A 91 11.132 9.119 1.148 1.00 0.00

ATOM 755 CG1 ILE A 91 11.057 10.632 1.371 1.00 0.00

ATOM 756 CG2 ILE A 91 9.769 8.568 0.758 1.00 0.00

ATOM 757 CD1 ILE A 91 10.110 11.039 2.477 1.00 0.00

ATOM 758 N LEU A 92 12.376 6.273 1.536 1.00 0.00

ATOM 759 CA LEU A 92 12.307 4.833 1.314 1.00 0.00

ATOM 760 C LEU A 92 12.363 4.068 2.631 1.00 0.00

ATOM 761 O LEU A 92 11.556 3.172 2.876 1.00 0.00

ATOM 762 CB LEU A 92 13.445 4.385 0.388 1.00 0.00

ATOM 763 CG LEU A 92 13.586 2.868 0.214 1.00 0.00

ATOM 764 CD1 LEU A 92 12.272 2.273 -0.274 1.00 0.00

ATOM 765 CD2 LEU A 92 14.710 2.570 -0.767 1.00 0.00

ATOM 766 N GLU A 93 13.323 4.429 3.478 1.00 0.00

ATOM 767 CA GLU A 93 13.518 3.745 4.750 1.00 0.00

ATOM 768 C GLU A 93 12.319 3.936 5.668 1.00 0.00

ATOM 769 O GLU A 93 11.867 2.995 6.320 1.00 0.00

ATOM 770 CB GLU A 93 14.791 4.246 5.437 1.00 0.00

ATOM 771 CG GLU A 93 16.083 3.774 4.786 1.00 0.00

ATOM 772 CD GLU A 93 17.282 4.422 5.421 1.00 0.00

ATOM 773 OE1 GLU A 93 17.101 5.257 6.274 1.00 0.00

ATOM 774 OE2 GLU A 93 18.380 4.004 5.135 1.00 0.00

ATOM 775 N ASP A 94 11.805 5.161 5.715 1.00 0.00

ATOM 776 CA ASP A 94 10.652 5.476 6.549 1.00 0.00

ATOM 777 C ASP A 94 9.416 4.708 6.096 1.00 0.00

ATOM 778 O ASP A 94 8.691 4.141 6.914 1.00 0.00

ATOM 779 CB ASP A 94 10.372 6.980 6.530 1.00 0.00

ATOM 780 CG ASP A 94 11.351 7.813 7.346 1.00 0.00

ATOM 781 OD1 ASP A 94 12.076 7.245 8.128 1.00 0.00

ATOM 782 OD2 ASP A 94 11.478 8.982 7.071 1.00 0.00

ATOM 783 N LEU A 95 9.182 4.691 4.788 1.00 0.00

ATOM 784 CA LEU A 95 8.018 4.016 4.228 1.00 0.00

ATOM 785 C LEU A 95 8.123 2.506 4.395 1.00 0.00

ATOM 786 O LEU A 95 7.118 1.823 4.588 1.00 0.00

ATOM 787 CB LEU A 95 7.858 4.380 2.746 1.00 0.00

ATOM 788 CG LEU A 95 6.931 5.569 2.462 1.00 0.00

ATOM 789 CD1 LEU A 95 7.197 6.687 3.461 1.00 0.00

ATOM 790 CD2 LEU A 95 7.150 6.056 1.038 1.00 0.00

ATOM 791 N LYS A 96 9.345 1.991 4.320 1.00 0.00

ATOM 792 CA LYS A 96 9.595 0.573 4.554 1.00 0.00

ATOM 793 C LYS A 96 9.286 0.192 5.996 1.00 0.00

ATOM 794 O LYS A 96 8.692 -0.854 6.258 1.00 0.00

ATOM 795 CB LYS A 96 11.045 0.224 4.218 1.00 0.00

ATOM 796 CG LYS A 96 11.421 -1.228 4.487 1.00 0.00

ATOM 797 CD LYS A 96 10.650 -2.177 3.581 1.00 0.00

ATOM 798 CE LYS A 96 11.080 -3.620 3.797 1.00 0.00

ATOM 799 NZ LYS A 96 10.308 -4.564 2.942 1.00 0.00

ATOM 800 N LYS A 97 9.691 1.046 6.929 1.00 0.00

ATOM 801 CA LYS A 97 9.416 0.823 8.344 1.00 0.00

ATOM 802 C LYS A 97 7.924 0.912 8.636 1.00 0.00

ATOM 803 O LYS A 97 7.401 0.179 9.475 1.00 0.00

ATOM 804 CB LYS A 97 10.181 1.830 9.204 1.00 0.00

ATOM 805 CG LYS A 97 11.685 1.600 9.254 1.00 0.00

ATOM 806 CD LYS A 97 12.383 2.680 10.067 1.00 0.00

ATOM 807 CE LYS A 97 13.891 2.478 10.078 1.00 0.00

ATOM 808 NZ LYS A 97 14.589 3.537 10.856 1.00 0.00

ATOM 809 N LYS A 98 7.243 1.816 7.939 1.00 0.00

ATOM 810 CA LYS A 98 5.799 1.961 8.078 1.00 0.00

ATOM 811 C LYS A 98 5.067 0.749 7.514 1.00 0.00

ATOM 812 O LYS A 98 4.063 0.305 8.071 1.00 0.00

ATOM 813 CB LYS A 98 5.320 3.236 7.382 1.00 0.00

ATOM 814 CG LYS A 98 5.703 4.524 8.098 1.00 0.00

ATOM 815 CD LYS A 98 5.241 5.748 7.320 1.00 0.00

ATOM 816 CE LYS A 98 5.650 7.036 8.018 1.00 0.00

ATOM 817 NZ LYS A 98 5.165 8.241 7.292 1.00 0.00

ATOM 818 N GLY A 99 5.575 0.219 6.407 1.00 0.00

ATOM 819 CA GLY A 99 4.996 -0.968 5.791 1.00 0.00

ATOM 820 C GLY A 99 5.195 -2.197 6.670 1.00 0.00

ATOM 821 O GLY A 99 6.173 -2.290 7.412 1.00 0.00

ATOM 822 OXT GLY A 99 4.393 -3.088 6.643 1.00 0.00

TER

ENDMDL
